# Optimizing Read Mapping to Reference Genomes to Determine Composition and Species Prevalence in Microbial Communities

**DOI:** 10.1371/journal.pone.0036427

**Published:** 2012-06-13

**Authors:** John Martin, Sean Sykes, Sarah Young, Karthik Kota, Ravi Sanka, Nihar Sheth, Joshua Orvis, Erica Sodergren, Zhengyuan Wang, George M. Weinstock, Makedonka Mitreva

**Affiliations:** 1 The Genome Institute, Washington University School of Medicine, St. Louis, Missouri, United States of America; 2 The Broad Institute of MIT and Harvard, Cambridge, Massachusetts, United States of America; 3 J. Craig Venter Institute, Rockville, Maryland, United States of America; 4 Virginia Commonwealth University, Center for Study of Biological Complexity, Richmond, Virginia, United States of America; 5 Insitute for Genome Sciences, University of Maryland School of Medicine, Baltimore, Maryland, United States of America; 6 Department of Genetics, Washington University School of Medicine, St. Louis, Missouri, United States of America; Albert Einstein College of Medicine, United States of America

## Abstract

The Human Microbiome Project (HMP) aims to characterize the microbial communities of 18 body sites from healthy individuals. To accomplish this, the HMP generated two types of shotgun data: reference shotgun sequences isolated from different anatomical sites on the human body and shotgun metagenomic sequences from the microbial communities of each site. The alignment strategy for characterizing these metagenomic communities using available reference sequence is important to the success of HMP data analysis. Six next-generation aligners were used to align a community of known composition against a database comprising reference organisms known to be present in that community. All aligners report nearly complete genome coverage (>97%) for strains with over 6X depth of coverage, however they differ in speed, memory requirement and ease of use issues such as database size limitations and supported mapping strategies. The selected aligner was tested across a range of parameters to maximize sensitivity while maintaining a low false positive rate. We found that constraining alignment length had more impact on sensitivity than does constraining similarity in all cases tested. However, when reference species were replaced with phylogenetic neighbors, similarity begins to play a larger role in detection. We also show that choosing the top hit randomly when multiple, equally strong mappings are available increases overall sensitivity at the expense of taxonomic resolution. The results of this study identified a strategy that was used to map over 3 tera-bases of microbial sequence against a database of more than 5,000 reference genomes in just over a month.

## Introduction

A key goal of the Human Microbiome Project (HMP) is the characterization of the microbial communities present in different body habitats [1]. An important part of this characterization is determining the presence and abundance of organisms within each habitat. The HMP generated three types of sequence data, sequences of the genes coding 16S rRNA and two types of shotgun data: reference sequences isolated from different anatomical sites on the human body and shotgun metagenomic sequences from the microbial communities of each site. The most widely used approach to report on species abundance in metagenomic collections is by surveying the bacterial 16S rRNA genes (e.g. *see* [2]–[3]). For metagenomic samples where there has been deep sequencing of 16S RNA, alignments are generated using a number of tools (such as ARB [4] and the NAST [5]), and profiles of species presence and abundance from different sources are displayed individually or together in a plot. The 16S rRNA sequences are explored in phylogenetic or phylogeny-independent space [6]. However, while well defined and frequently used, 16S rRNA based community profiling has its limitations, such as the use of degenerate primers for 16S amplification that do not capture all community members, variable copy numbers of 16S rRNA genes in different species, the fact that PCR amplification is involved, the use of incomplete 16S rRNA databases and the inability to capture viruses and eukaryotes.

An alternative method to characterize the structure of microbial communities is to generate shotgun metagenomic sequence, which provides advantages such as the exclusion of biases introduced by using 16S marker gene for community profiling. Shotgun sequencing bias is introduced mainly from the sequencing platform used and thus provides a better absolute measurement of species abundances than do 16S rRNA measurements assuming adequate coverage is generated. Hence, aligning the shotgun metagenomic sequences generated from samples originating from the different body habitats against microbial reference genomes can generate abundance tables that contain information for comparative metagenomics that are free of typical 16s biases. The best method for generating comprehensive abundance tables is to align the metagenomic shotgun reads against a collection of reference genomes comprising the whole genome sequences of all available microorganisms (including the four major superkingdoms, Archaea, Bacteria, Eukaryota and Viruses). To accomplish this in a timely and robust manner for the HMP, which generated over 7 tera-bases of sequence data, effort was invested in the exploration of available tools and methods.

A wide variety of short read alignment software has been developed in recent years [7], presenting the HMP with many potential tools capable of performing the analysis. We chose to limit this comparison to aligners with which members of the HMP Data Processing Group had experience. Since many short read aligners were designed for human re-sequencing which has limited sequence diversity, we relied on a prior knowledge of the variables each parameter represents in reaching our goal of choosing and optimizing an aligner for mapping shotgun metagenomic sequences to a database of reference genomes. We evaluated the performance of six aligners with regards to the identification of microbial sequences in shotgun metagenomic samples, and their correctness in taxonomic assignment and estimation of prevalence with the goal of ensuring that this analysis be both robust and timely. The aligners were evaluated on i) Accuracy, ii) Sensitivity, iii) Speed & Scalability and iv) Convenience of use. The selected aligner was further evaluated, optimized alignment parameters were identified and the effect of mapping strategy on the ability to resolve hits at different taxonomic levels was investigated. Finally, taking into consideration that in many cases metagenomic reads originate from unculturable organisms or organisms not having a reference genome, we investigated the behavior of the aligner when the species known to be present in a community were removed, leaving only neighboring species from the same genus to be mapped against. This paper discusses these aligner optimizations in detail, describes the creation of the reference database and outlines the HMP’s read mapping Standard Operating Procedure.

## Results

### Reference Database Creation

**Figure 1:**
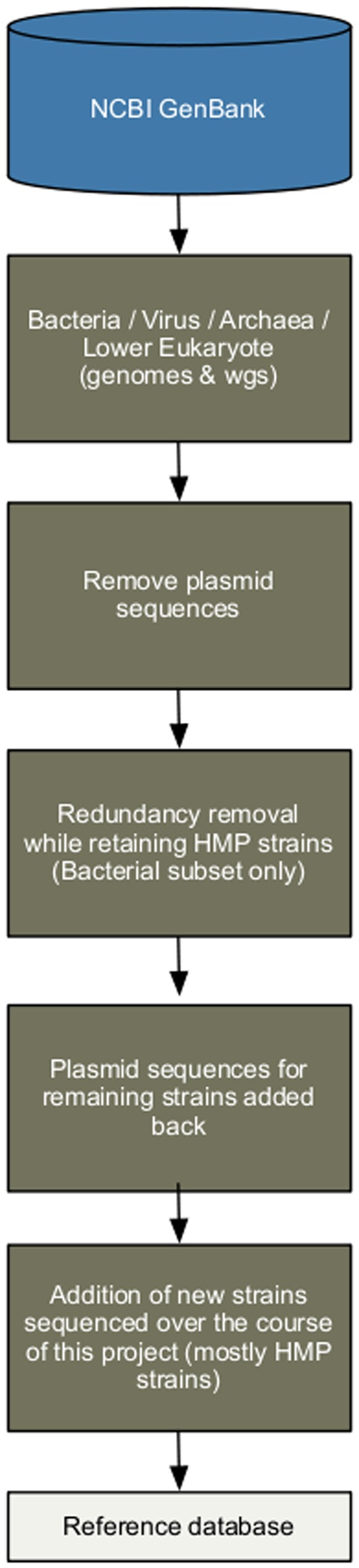
Reference Genome Database creation. An overview of the process of creating our Reference Genome Database (RGD). Complete and WGS genomes were downloaded from GenBank, plasmid sequences were removed to simplify redundancy screening, and then the Mauve genome assembly tool was used to identify redundant strains that were subsequently removed (except for HMP stains which were always kept). For strains remaining after redundancy removal, their corresponding plasmids were restored into the database. This database was periodically updated as new strains became available over the course of the project.

The final Reference Genome Database (RGD)(Figure 1) that was used in the ‘Mapping resolution’ analysis contained 1,751 bacterial genomes spread over 1,253 species. The other components of the database covered: i) Archaea: 131 genomes over 97 species, ii) Lower eukaryotes: 326 genomes over 326 species and iii) Viruses: 3,683 genomes over 1,420 species. The process of removing highly redundant bacterial strains (*see* Methods) resulted in the elimination of 2,265 complete and draft bacterial genomes and corresponding plasmid sequences, resulting in 5891 remaining genomes across the four superkingdoms.

The Mock Metagenomic Database (MMD) that was used for aligner comparisons and parameter optimization comprised 20 bacterial genomes from 17 genera and one archaeal strain (*see* Methods). These 21 organisms are represented by 51 sequences in a fasta database about 82 Mb in size (Text S1).

### Aligner Comparison

The percentage of the 22,735,802 mock community reads that mapped to the Mock Metagenomic Database (MMD) ranged from 63% to 92%, with the two extremes being from SMALT and SOAP (Table 1). All the aligners correctly show near-complete coverage of most genomes in the MMD with relatively similar abundances (Table 2). Differences observed for the MAP and SMALT aligners may result from their inability to report alignments in which the query maps equally well to multiple locations. The MAP aligner was set to report hits using its topN setting of 5, and for SMALT, only the reads mapped uniquely were reported. The SOAP aligner showed uniformly less coverage across all genomes (Table 3), and a statistically significant difference in depth of coverage was detected (at p = 0.05, Chi-square test). On average across all aligners, 82% of mock reads were mapped back to the MMD. When the detected strain abundances was compared to the actual mock community concentrations using Spearman’s rank test the correlation coefficients were between 0.7-0.8 (Table 4).

**Table 1:** Aligner metrics, requirements and performance.

Aligners	Default Parameters	Mapping Style	Memory Footprint (Gb)	Databasesize limit limit	Paired and fragment reads map together	Time of run (minutes)	Reads mapped	
							(#)	% of total
SOAP	M = 4 r = 1 m = 150 x = 600	top random	<4	4 Gb	no	84	14,376,440	63.23
MAP	w = 16 a = 3–legacy-cigar	topN = 5	14	no limit	no	24	19,491,796	85.73
SMALT	f = samsoft	unique only	<4	varies*	no	165	20,874,489	91.81
BWA	<default>	top random	<4	4 Gb	no	120	18,446,241	81.13
CLC	q p fb ss 180 250	top random	∼2	no limit	yes	15	20,184,374	88.78
NOVOALIGN	-F STDFQ -r Random -o SAM -I PE 215,50	top random	<1	20 Gb**	no	206	19,330,244	85.02

* varies depending on search window size.

** 4 Gb x step size limit of aligner (max value 5).

**Table 2:** Sensitivity and specificity comparison.

			Aligner and coverage											
			BWA		MAP		CLC		SMALT		SOAP		NOVOALIGN	
Species			Breadth	Depth	Breadth	Depth	Breadth	Depth	Breadth	Depth	Breadth	Depth	Breadth	Depth
*Deinococcus radiodurans R1*			99.98	237.75	98.79	252.83	99.99	295.15	99.46	292.42	99.87	165.49	99.97	245.59
*Acinetobacter baumannii ATCC 17978*			99.98	73.02	99.99	76.73	99.99	76.25	99.52	76.06	99.90	68.53	99.98	73.62
*Staphylococcus* *epidermidis ATCC 12228*			99.99	40.39	99.55	37.28	99.99	42.22	98.66	41.99	99.97	38.94	99.99	40.70
*Helicobacter pylori 26695*			99.96	38.76	99.98	41.64	99.97	40.43	98.10	39.45	99.97	36.66	99.95	39.04
*Bacteroides vulgatus* *ATCC 8482*			100.00	36.95	99.51	39.47	100.00	38.75	96.98	37.53	100.00	34.62	100.00	37.24
*Propionibacterium acnes KPA171202*			99.98	34.96	99.99	37.75	99.98	39.28	99.52	39.41	99.98	28.20	99.98	35.53
*Streptococcus* *pneumoniae TI GR4*			100.00	34.48	99.38	36.70	100.00	35.88	97.85	34.97	99.99	32.79	100.00	34.74
*Streptococcus mutans UA159*			100.00	22.15	99.85	23.74	100.00	23.02	98.24	22.78	100.00	21.11	100.00	22.29
*Neisseria meningitidis MC58*			99.99	21.60	99.85	24.94	100.00	23.97	95.00	22.62	99.89	18.25	99.99	21.85
*Staphylococcus aureus USA300_TCH1516*			91.46	20.90	92.03	22.64	92.56	22.91	92.09	22.75	88.25	20.73	91.57	21.52
*Actinomyces* *odontolyticus ATCC 17982*			99.90	18.88	99.94	21.15	99.94	22.58	99.37	22.87	99.51	13.91	99.91	19.44
*Listeria monocytogenes EGD-e*			100.00	15.52	99.32	15.85	100.00	16.12	99.07	16.15	99.99	14.79	99.99	15.62
*Rhodobacter sphaeroides 2.4.1*			99.05	12.87	99.23	15.12	99.60	16.99	99.41	17.50	95.12	8.74	98.92	13.39
*Enterococcus faecalis OG1RF*			99.96	11.09	99.97	11.55	99.97	11.55	99.26	11.61	99.92	10.55	99.95	11.16
*Clostridium beijerinckii NCIMB 8052*			99.90	10.25	98.99	10.44	99.91	10.64	98.65	10.64	99.88	9.92	99.90	10.32
*Escherichia coli K12*			99.48	7.40	98.95	7.76	99.62	7.96	98.49	8.02	98.72	6.63	99.40	7.46
*Methanobrevibacter* *smithii ATCC 35061*			97.91	6.54	97.35	6.72	98.19	6.79	97.01	6.87	97.70	6.46	97.90	6.59
*Bacillus cereus ATCC* *10987*			89.60	3.18	89.28	3.26	90.17	3.30	89.72	3.38	88.70	3.44	89.58	3.21
*Pseudomonas aeruginosa PAO1*			80.50	2.24	82.50	2.59	86.18	2.84	89.41	3.16	65.73	2.31	79.80	2.32
*Streptococcus agalactiae 2603V/R*			47.94	0.91	47.76	0.92	49.00	0.96	51.63	1.03	46.94	1.88	48.02	0.92
*Lactobacillus gasseri* *ATCC 33323*			20.05	0.31	20.98	0.33	20.73	0.32	25.98	0.42	19.64	1.53	20.11	0.31

A detection cutoff of 1% breadth and 0.01x depth of coverage was used allowing the detection of low abundance species (such as *Escherichia coli* in the gut, e.g. [8]) while reducing the incidence of only spurious alignments being reported. All programs identified the most abundant species present in the proper order of prevalence, and in fact were able to detect all 21 bacterial species present in the mock community mixture. Some key aberrations include the observation that the SOAP aligner found a notably smaller depth of coverage for the most abundant organism (*Deinococcus radiodurans R1*) and also found a noticeably lower breadth of coverage for one of the less abundant species (*Pseudomonas aeruginosa PAO1*). The alignment softwares were also benchmarked for their performance and other limitations such as the maximum size of the database that can be searched against. The database size for BWA and SOAP is limited to 4 Gb (Table 1), and while SMALT claims to allow larger database sizes as the default search window size is increased, we had difficulties getting anything larger than a 6 Gb database to work reliably on available hardware. Novoalign supports searches against databases of up to 4 Gb x the ‘step size limit’ of the aligner, which has a maximum value of 5, resulting in a limit of 20 Gb. Using default settings for Novoalign we were able to handle the 7.3 Gb RGD, however it was the slowest aligner tested (Table 1). Both MAP and CLC are limited only by how much memory can be made available on the machine running the alignment software although it appears that the CLC aligner is somewhat more memory efficient in comparison to MAP. CLC also proved to be the only aligner capable of mapping both paired end reads and fragment reads from a sample in a single execution while taking advantage of pairing information. In summary, the CLC aligner displayed the best speed and a small memory footprint, is able to handle the 7.3 Gb RGD in a single alignment on our current hardware and it has the ability to map both paired end reads and fragment reads in a single execution while taking advantage of pairing information (Table 1). Therefore, the CLC aligner was chosen for further analyses reported in this paper.

### Parameter Optimization

**Figure 2:**
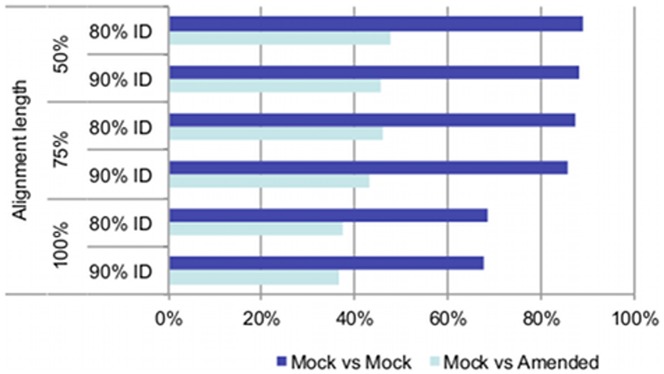
Parameter effects on mapping against the MMD and the amended MMD. This plot shows the percent of total mock queries able to be mapped to the mock database at each given CLC parameter combination. The Mock vs. Mock data (dark blue) uses the original MMD, which contains all strains present in the mock community. The Mock vs. Amended data (light blue) shows the same results when the mock query is mapped to an amended MMD where several strains were removed and other strains from the same genus were included in their place.

We looked first at the total number of reads mapped at each parameter combination. The Illumina GAIIx reads from the mock community (22,735,802 reads) were aligned to the MMD, which contained genome sequences for all organisms in the mock community. We found that the minimum length of alignment required (in terms of query length) has more of an effect on mapping sensitivity than does varying the percent identity required within the length of the alignment (Figure 2, Mock vs. Mock data). The two less-stringent length settings perform similarly well, while the 100% length requirement results in a significant decrease in hits detected. In all cases, decreasing the percent identity requirement causes a minor increase in the number of hits detected, but this change is trivial compared to reducing the length constraint below 100%.

The different parameter combinations were also evaluated in regards to their ability to identify each genus independently by looking at the effects on the breadth and depth of coverage for all the genomes present in the mock community. Figure S2A shows the parameter effects on breadth of coverage, and Figure S2B shows the effect on depth of coverage at the genus level. In both breadth and depth of coverage, only the 100% length requirement seems to have an impact on the ability to detect organisms at the genus level. That most stringent length criteria fails to identify almost 15% of the *P. aeruginosa PAO1* reference sequence that can be picked up by the less conservative cutoffs (Figure S2A). Looking at the detected depth of coverage also shows a significant loss of sensitivity when using the 100% length cutoff. In this case the most obvious effect can be seen in the most abundant genus *Deinococcus*, reducing the depth of coverage by 100 fold when subjected to the more stringent length requirement. Similar but smaller effects can be seen in most of the other genera (Figure S2B).

**Table 3:** Chi-square comparison of detected abundances*.

	SOAP	MAP	SMALT	BWA	CLC	NOVOALIGN
SOAP		0.28385	0.0217	0.49387	0.01281	0.37475
MAP			0.99972	1	0.99992	1
SMALT				0.9992	1	0.99991
BWA					0.99989	1
CLC						0.99997
NOVOALIGN						

* Based on depth of coverage per genome. Values > = 0.05 are considered significantly similar.

**Table 4:** Spearman's rank correlation with true MMD concentration.

Aligner	Correlation coefficient
SOAP	0.6960784
MAP	0.7720588
SMALT	0.7941176
BWA	0.7573529
CLC	0.7916667
NOVOALIGN	0.7720588

**Figure 3:**
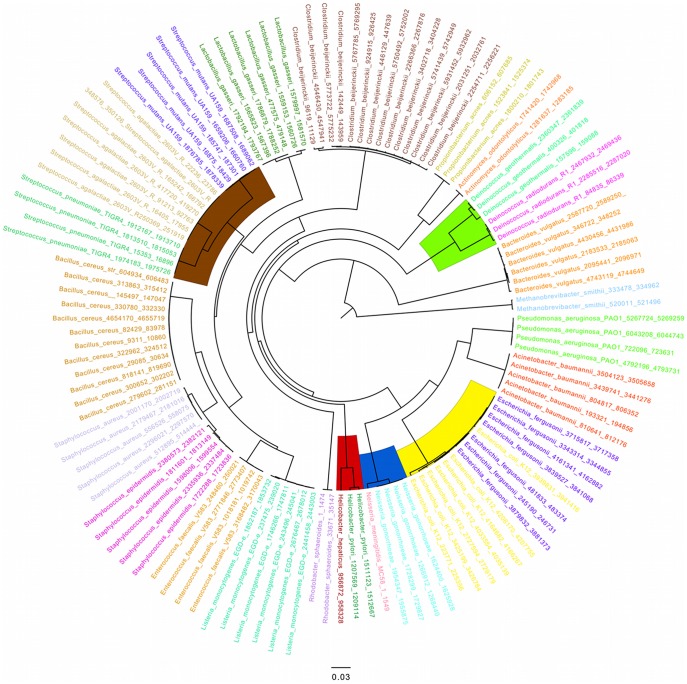
Phylogenetic tree view of MMD strains and amended MMD strains based on 16S genes. This image displays a phylogenetic tree based on 16S data for all 21 strains in the MMD, and also the 4 strains used as replacements in the amended MMD (*D. geothermalis*, *Helicobacter hepaticus*, *Neisseria gonorrheae* and *Escherichia fergusonii*). The shaded regions indicate the genera containing the amended strains (yellow: *Escherichia*, blue: *Neisseria*, red: *Helicobacter*, brown: *Streptococcus* and green: *Deinococcus*).

Often the genome of the exact strain present in a microbial community is not represented in the RGD. Therefore, we tested the parameters under low identity conditions, when the exact query strain is not present in the reference, but a taxonomically related organism from the same genus is (Table 5). The same set of alignment parameters was used, but the MMD was amended by replacing several of the strains present with other organisms of the same genus (Table 5 describes the amended MMD strains and their similarity to the original strain that they replaced and Figure 3 displays a 16S rRNA tree showing the phylogeny of the original 21 organisms and the 4 new amended ones). When recording the total numbers of reads mapped at each cutoff combination, the major effect was still the same, i.e., varying the length constraint has the largest effect on alignment sensitivity. But this time we observed that the less stringent 80% identity cutoff can map ∼5% more reads than the more stringent 90% cutoff (Figure 2). These additional identifications are likely due to conservation between phylogenetically related species (Figure 2, Mock vs. Amended data). The number of reads hitting the modified MMD drops from ∼80% down to ∼45% overall because one of the species swapped out was the most abundant organism in the mock community, *D. radiodurans R1*. A large number of possible mappings for the mock query set were lost when *Deinococcus geothermalis,* a species only ∼46% similar at the genome level, was swapped in for *D. radiodurans R1*.

**Figure 4:**
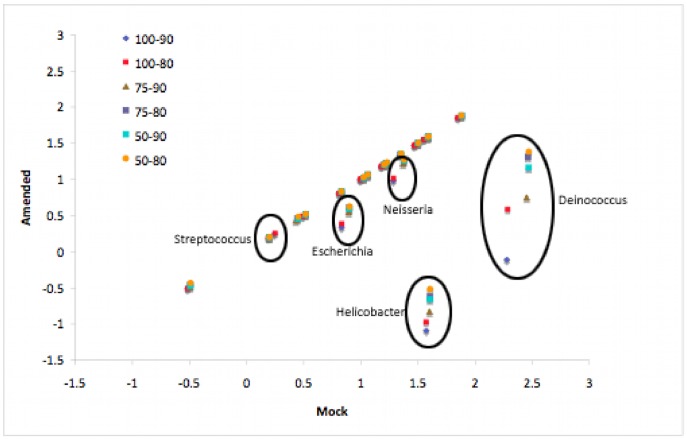
A comparison of mock and amended MMD depth of coverage. This plot shows the log transformed depth values for the mock query versus the amended MMD on the y-axis, and the mock query versus the original MMD on the x-axis. Unaffected genera should lie along the diagonal, while those showing a change in depth of coverage will fall off the diagonal. The amended genera are indicated, and the 4 that were swapped do stand off the diagonal. The genus *Streptococcus* was represented by 3 strains in the mock community, and was amended by removing two of the three strains leaving only *S. mutans UA159* in the amended MMD. The depth value of this multi-strain genus was the read normalized average value of the 3 member strains, and after being pruned down to a single strain, the single strain depth remained similar to the original, averaged value.

Looking into the coverage of the amended MMD (Figure S3), we observed that the strains that were replaced all suffer a loss of both depth and breadth of coverage, but this time a more pronounced effect was seen from the percent similarity requirement on these alignments. Also, while the depth and breadth covered across all 5 amended genera did drop, they did not drop to zero, suggesting retained coverage of the conserved regions among the original and replaced genomes. Figure 4 gives an overview of these effects, showing that the 4 genera with amended strains fall off the diagonal when plotting log transformed depth values between mappings to the original MMD and mappings to the amended MMD. The genus *Streptococcus* was also amended, but this genus was one of two genera in the mock community represented by more than a single species (the other being *Staphylococcus*). Rather than replacing all 3 *Streptococcus* species, in this case 2 of the 3 original species were removed, leaving only *Streptococcus mutans UA159*. Depth values for these multi-species genera were calculated as the mean of the member species and the depth of coverage for *S. mutans UA159* alone is very close to the mean depth of all 3 *Streptococcus* species. Thus it falls on the diagonal along with the other, un-amended genera.

Basing the decision on these observations, the suggested cutoff for community profiling using shotgun metagenomic sequences is 80% identity over 75% of the length of the query. This setting represents a good balance between sensitivity and accuracy, even in an environment where not all strains in the community will be represented in the reference database.

**Table 5:** Amended strain similarity to original MMD strains.

Genus	Original strain in mock community	Replacement strain	Genome wide similarity
*Deinococcus*	*Deinococcus radiodurans R1*	*Deinococcus geothermalis*	∼46%
*Helicobacter*	*Helicobacter pylori*	*Helicobacter hepaticus*	∼15%
*Neisseria*	*Neisseria meningitidis*	*Neisseria gonorrheae*	∼81%
*Escherichia*	*Escherichia coli K12*	*Escherichia fergusonii*	∼78%
	*Streptococcus agalactiae 2603V/R*		
*Streptococcus*	*Streptococcus mutans UA159*	*Streptococcus mutans UA159* *	na
	*Streptococcus pneumoniae TIGR4*		

* For *Streptococcus*, 2 of the 3 strains were removed, leaving only S.mutans UA159.

### Mapping Resolution Analysis

**Figure 5:**
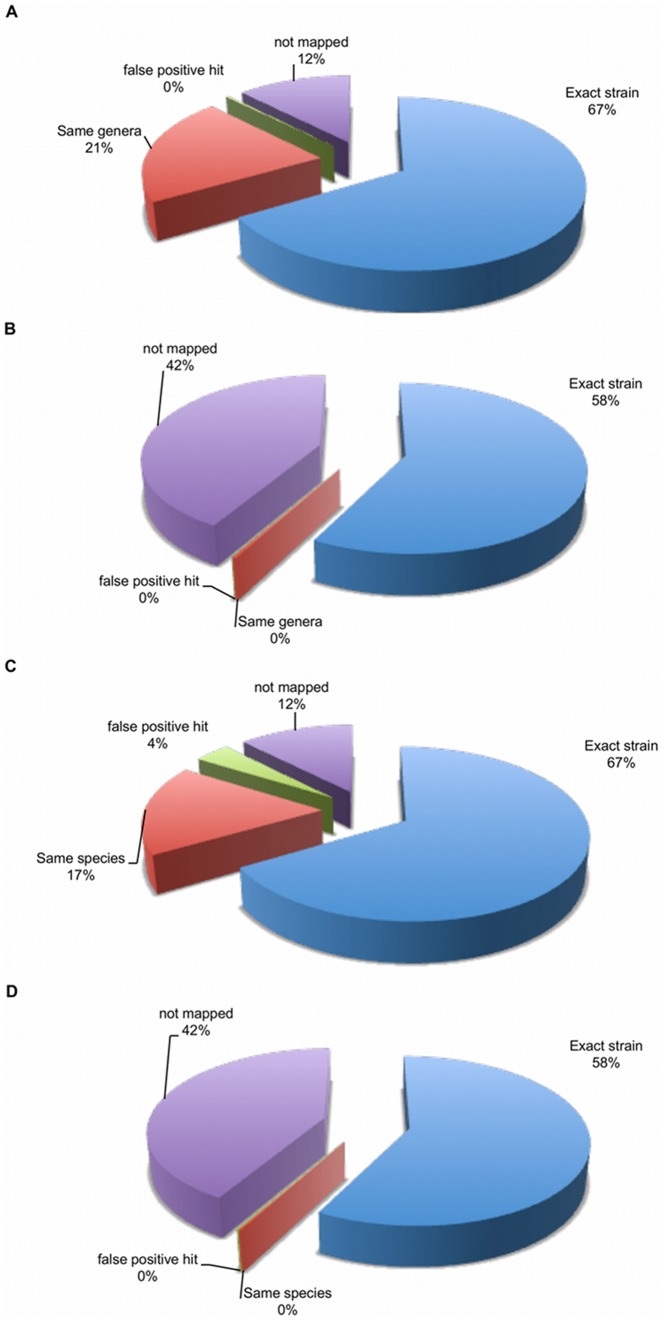
Mapping Resolution. (**A**). **Using a top random mapping strategy to classify at the strain or genus level.** This image shows the mapping fate of all 22,735,802 mock queries when mapped to the RGD under a top random mapping strategy, falling back to genus level annotations when the strain cannot be identified. (**B**). **Using a unique only mapping strategy to classify at the strain or genus level.** This image shows the mapping fate of all 22,735,802 mock queries when mapped to the RGD under a unique only mapping strategy, falling back to genus level annotations when the strain cannot be identified. (**C**). **Using a top random mapping strategy to classify at the strain or species level.** This image shows the mapping fate of all 22,735,802 mock queries when mapped to the RGD under a top random mapping strategy, falling back to species level annotations when the strain cannot be identified. (**D**). **Using a unique only mapping strategy to classify at the strain or species level.** This image shows the mapping fate of all 22,735,802 mock queries when mapped to the RGD under a unique only mapping strategy, falling back to species level annotations when the strain cannot be identified.

We next mapped the reads from the mock community against the RGD. When using the ‘top random’ mapping strategy (when the aligner randomly reports one hit in the case of multiple equally high scoring top hits) with 80% identity and a 75% fraction of length cutoff, 67% of all mappings are to the correct strains present in the mock community, 21% map to non-mock community strains but within the correct genus, 12% of reads don’t map at all and close to 0% of reads (63,321 out of 22,735,802) map to an organism of the wrong genus (Figure 5A). When using the same alignments re-parsed under ‘unique placement only’ rules (query aligning equally well to more than a single location in the reference is not reported as a hit), only 58% of the reads are mapping to the correct strain, with the majority of the remainder not able to map uniquely (Figure 5B).

Considering these results at the species rather than the genus level, we find that under the top random mapping strategy, about 4% of the reads that had previously been classified to the correct genus were not able to be assigned to the correct species (Figure 5C). This 4% false positive rate is not seen when using the unique only alignment strategy. Under unique only rules, reads that do map can be annotated at the species level with almost the same confidence as they can be at the genus level.

**Figure 6:**
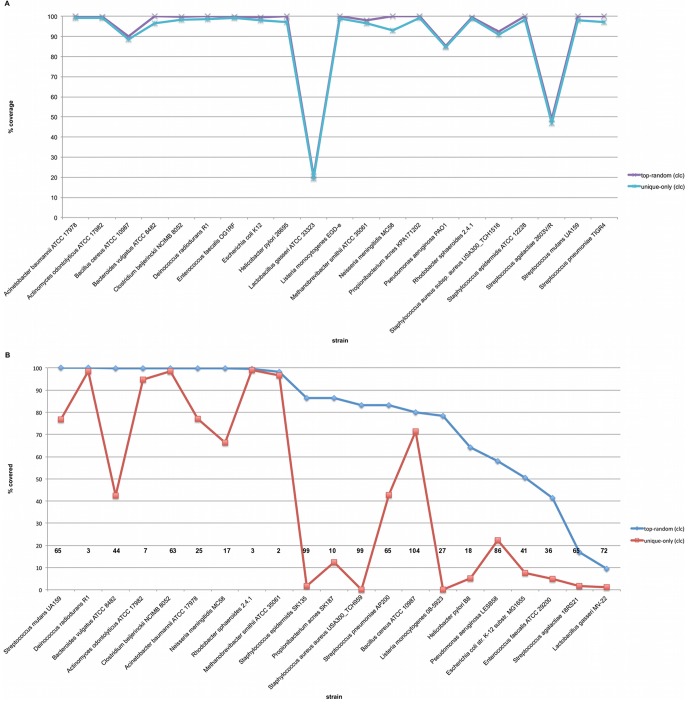
Mock strain coverage. (**A**). **Coverage of mock strains in the MMD.** This image shows the results of mapping the mock community query against the MMD under both top random and unique only mapping strategies. (**B**). **Coverage of mock strains in the RGD.** This image shows the results of mapping the mock community query against the RGD under both top random and unique only mapping strategies. The numbers printed in the plot reflect the number of strains present in the RGD for the genus in which the displayed strain belongs.

We also plotted the detected coverage of mock strains when the 22,735,802 mock query sequences were aligned against both the MMD and RGD under both top random and unique placement only mapping strategies. When mapped against the MMD, both strategies displayed very similar coverage for all strains (Figure 6A). However, results against the RGD show that the number of strains represented in the database within each genus has a significant effect on the coverage of the specific organism in the mock community (Figure 6B). Mock community organisms with many similar strains available within the reference database show good breadth of coverage under top random rules, but poor coverage under unique placement only rules (e.g. *Bacteroides vulgatus ATCC 8482*, the *Streptococcus* strains and the *Staphylococcus* strains). While organisms with very few related genomes show similar detected coverage under both rule sets (e.g. *D. radiodurans R1*, *Rhodobacter sphaeroides 2.4.1* and *Methanobrevibacter smithii ATCC 35061*).

**Figure 7:**
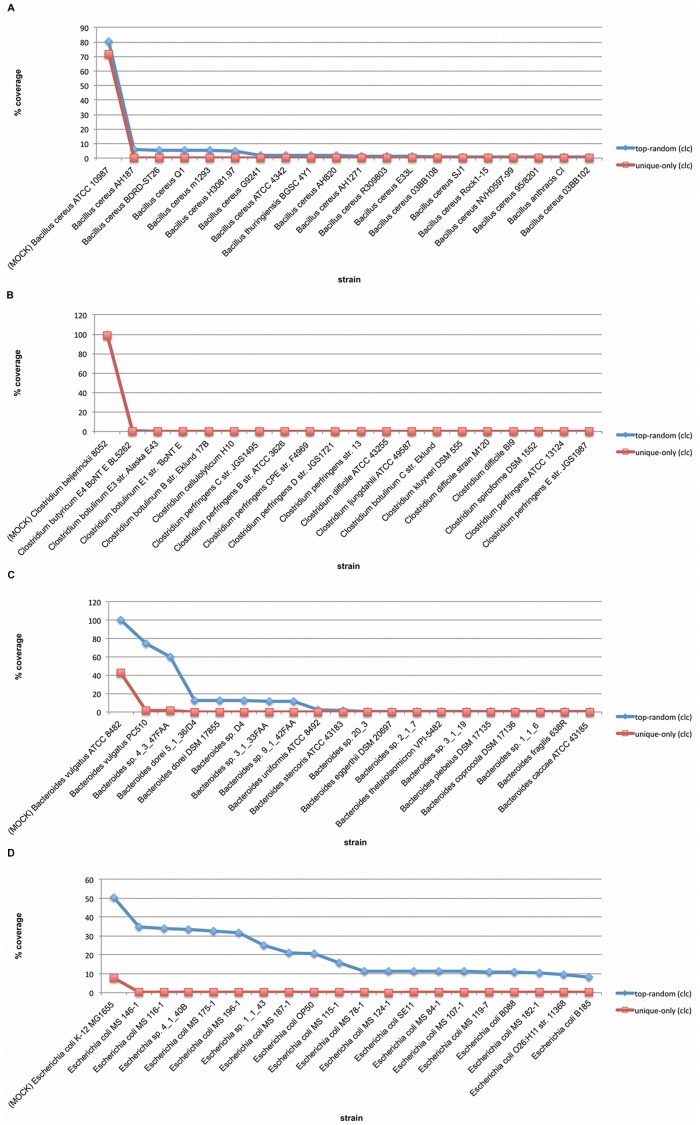
The effect of strain representation within the RGD and mapping strategy on mapping resolution. (**A**). **Top 20 most covered strains from the genus** ***Bacillus*** **found in mock vs. RGD mapping.** This figure shows the detected coverage using both mapping strategies when aligning the mock community queries against the full RGD for *Bacillus* strains. Note that the strain present in the mock community (*B. cereus ATCC 10987*) is indicated in the image. (**B**). **Top 20 most covered strains from the genus** ***Clostridium*** **found in mock vs. RGD mapping.** This figure shows the detected coverage using both mapping strategies when aligning the mock community queries against the full RGD for *Clostridium* strains. Note that the strain present in the mock community (*C. beijerinckii NCIMB 8052*) is indicated in the image. (**C**). **Top 20 most covered strains from the genus** ***Bacteroides*** **found in mock vs. RGD mapping.** This figure shows the detected coverage using both mapping strategies when aligning the mock community queries against the full RGD for *Bacteroides* strains. Note that the strain present in the mock community (*B. vulgatus ATCC 8482*) is indicated in the image. (**D**). **Top 20 most covered strains from the genus** ***Escherichia*** **found in mock vs. RGD mapping.** This figure shows the detected coverage using both mapping strategies when aligning the mock community queries against the full RGD for *Escherichia* strains. Note that the strain present in the mock community (*E. coli K-12 MG1655*) is indicated in the image.

We found two cases for which this observation did not hold true. The mock strains *Bacillus cereus ATCC 10987* and *Clostridium beijerinckii NCIMB 8052* have 104 and 63 strains available within their genera respectively, yet demonstrated similar coverage results under both top random and unique only strategies. To investigate this effect we examined the coverage of all *Bacillus* strains and all *Clostridium* strains available in the RGD independently under both mapping strategies. Figures 7A and 7B show the top 20 most covered strains in each case under both alignment strategies. In both experiments it can be seen that the only strain with significant coverage under either strategy is the strain represented in the mock community. Conversely, we performed a similar experiment using two strains that behaved more as expected, *B. vulgatus ATCC 8482* and *E. coli K-12 MG1655*. Figures 7C and 7D show that in both cases there is significant bleed over coverage into neighboring, non-mock community strains that prevents the unique only mapping strategy from detecting significant coverage (since few reads are uniquely mappable in these strains), while top random coverage is divided amongst a number of conserved strains.

## Discussion

The accuracy was similar for most of the tested aligners, therefore primarily convenience issues, such as which tool has the smallest memory footprint and which tool benchmarks the fastest, drove the choice of aligner. Additional major determining factors were, i) which aligner could handle the size of our reference database, and ii) which aligner could map both paired end reads and fragment reads in a single execution. The number of reference genomes is increasing with a rapid rate. For example, only the HMP project is committed to sequencing 3,000 bacterial genomes over its course, resulting in an ever-increasing size of the RGD (presently 7.3 Gb). Many available next generation aligners impose a 4 Gb database size limitation, which is a technical hurdle for mapping algorithms that use the Burrows-Wheeler transform in their implementation (e.g. [9], [10]). Additionally, steps within the read mapping process (see Text S2) prior to alignment result in a fraction of the paired end reads being orphaned during low complexity screening, resulting in a sample having both a set of paired end reads, and a fragment read file that both need to be aligned. These issues together would have been a computational hurdle because they would have required us to run four alignments per sample to scan the full breadth of the RGD.

The SOAP aligner was a statistically significant outlier, detecting fewer hits to all strains in the MMD as compared to the other aligners. BWA’s primary weakness was its inability to handle a database larger than 4 Gb in size. The SMALT aligner, while claiming to be able support larger databases if the user increases search window size, was unable to handle a database larger than 6 Gb in our hands. In addition, the loss of sensitivity prompted by an increased window size (data not shown) was of concern. Novoalign displayed the smallest memory footprint of all aligners tested during our benchmarking. Its limitation proved to be speed, clocking in as the slowest aligner tested (over 10 fold slower than the frontrunner). MAP performed similarly to CLC, and was able to support the large database size we required, but the version tested was limited in that the only available mapping strategy revolved around their topN setting, which will only report hits with that number or fewer identical top hits (i.e. topN = 5 tells MAP not to report a query that aligns equally well to >5 spots in the reference). Drastically increasing the topN value to ensure we are not missing hits caused a significant increase in the amount of memory needed to complete the alignment. Note that parameter modifications have since been made in MAP to address this issue (Brian Hilbush, RealTime Genomics, personal communication), but only after this evaluation had been completed. Finally, only the CLC aligner was able to map both fragment and paired end reads in a single execution while still considering read paring information. While several aligners achieved similar levels of sensitivity and accuracy, the overall feature set that CLC offered tipped the balance and so it was selected for the optimization related analysis in our study.

None of the aligners compared were able to map 100% of the 22,735,802 mock community reads back to the MMD. Depending on the aligner, only 63% to 92% of the mock community queries could be aligned (Table 1). This is attributed in part to the fact that the mock query data had not been screened for low complexity. The DUST application [11] was used to mask low complexity sequence and subsequently remove it from the query set. This filtering accounted for 3-4% of the unmappable queries (data not shown). The inability to map the remainder of the reads is likely due to the fact that: i) of the 21 genomes included in the MMD, 2 are based on draft assemblies (*Actinomyces odontolyticus ATCC 17982* and *Enterococcus faecalis OG1RF*) therefore may not be complete representations of their respective genomes and ii) not all plasmid sequences associated with each strain were available in GenBank when the MMD was created.

The CLC parameters were tested to achieve maximum sensitivity while minimizing false positives on a gross level. Due to limitations in the availability of bacterial organisms for inclusion in the reference database, no amount of parameter tweaking will be able to completely overcome problems with false positives detection, but by considering the problem at a higher taxonomic level (the genus level), where we do have good representation across the phylogeny, we were able to arrive at a parameter combination that could provide a relatively good profiling of a microbial community.

Based on the results, in the ideal case when all organisms in the query pool are represented in the database (as in the case of aligning the mock query data against the MMD), it is apparent that the length constraint has a much stronger impact on sensitivity than did the various similarity settings tested. And it was also apparent that only the most stringent length requirements hampered sensitivity. But when we attempted to model the state of live data by replacing several strains with other organisms from within the same genus, we began to see a difference in community structure reflecting changes of required percent identity. This is expected when sequences are mapped to more divergent strains. Furthermore, there is a significant overall decrease in detection caused by the substitution of *D. geothermalis* [12] for *D. radiodurans R1*. *D. radiodurans R1* [13] is by far the most abundant strain present in the mock community, and its replacement (*D. geothermalis*) is only ∼46% similar at a genome wide level, so this was an expected result. Somewhat surprising is the fact that when the genus *Streptococcus* was modified by removing two of the three mock species present [14], [15], [16], the depth found for the remaining genome alone was approximately equivalent to the average depth found across all three *Streptococcus* species in the original MMD. One could have expected that under a top random mapping strategy the remaining species would have captured the reads that had originally mapped to the now missing species, increasing its reported depth of coverage. But instead we observed the same depth of coverage for the remaining species as was originally seen. A possible explanation for this is that the two removed species were diverse enough from *S. mutans UA159* to prevent any kind of cross mapping. Consistent with this, genome-wide pair-wise alignments between *S. mutans UA159* and the other two genomes shows a ∼51% and ∼35% similarity to *Streptococcus agalactiae 2603V/R* and *Streptococcus pneumoniae TIGR4* respectively.

The experiment investigating the effects of mapping strategy on taxonomic resolution (i.e. the ability to correctly identify an organism at a given taxonomic level) showed a clear trade-off between the fraction of the reads representing a sample that can be characterized and the accuracy of that classification. As shown in Figure 5, 88% of all reads can find a hit under the top random mapping strategy, but 21% of those alignments are incorrect at the strain level. Thus, using this strategy, we can only confidently classify reads to higher order taxonomies (the genus level in this figure). Under the unique placement only strategy we are able to annotate only 58% of reads, but in this case the characterization is accurate at the strain level. This ability to classify data to the strain level represents a key advantage that shotgun metagenomic sequences have over 16s rRNA characterizations made with the commonly-used RDP algorithm [17] which typically makes identifications (at 0.8 level of confidence) at the genus level.

Looking into the effect of strain representation within the reference database on mapping resolution, we found a relationship between the number of strains available in the RGD under a given genus and our ability to resolve down to the strain level. Mappings performed against the MMD, where only the mock strains known to be present were available, showed that mapping strategy played little role in the ability to detect coverage. In such a perfect scenario almost any hit will be to the correct strain because there are no phylogenetically closely related neighbors to compete for alignment within conserved regions that could preclude its detection under unique placement only rules. But when a query metagenomic shotgun sequence is mapped to the RGD, strains with many similar neighbors available in the database preclude accurate mappings to finer grained taxonomic levels. In summary, genera with many strains available in the reference database, such as *Bacteroides*, *Lactobacillus*, *Staphylococcus* and *Streptococcus* will present a challenge when trying to resolve to the strain level using the unique only mapping strategy. Genera with few strains available will offer strong resolving power using either mapping strategy. In the two examples where this did not hold true, the mock community strains were considerably divergent from other organisms within their genus (Figure 7) [18].

Furthermore, based on alignments of the mock queries against the RGD under the top random alignment strategy, we found that the number of false positive identifications at the species level is higher than what is seen when taxonomic assignments are made at the genus level. Approximately 4% of these classifications are incorrect at the species level, but all of those reads can be mapped to the correct genus. When using the unique only mapping protocol, we did find a few more false positive classifications at the species level, but the overall misclassification rate was not significantly inflated (0.3% false positive rate). This is expected because the only time a misclassification can happen under unique only rules is when the sequence’s strain of origin is missing from the RGD, but the read happens to fall into a region that is divergent from other close neighbors within the same species, but conserved in some other organism represented in the database. Based on our results, this is a very rare event.

In future studies, the more advanced approach would be to generate a pan-genome (e.g. [19]) reference database in which only the unique portions of genomes are represented. By maintaining only single instances of highly conserved regions, and by tracking which genomes share these unique conserved regions, one could confidently classify the shotgun metagenomic sequences to a lower taxonomic resolution more confidently. Using a pan-genome reference would allow either mapping strategy, top random and unique only, to see conserved sequence and offers solid annotation for all genomes sharing that region. This will allow the user to fine tune the taxonomic resolution based on available information, sometimes allowing annotation down to the species level where previously one could only assign a genus level classification.

In conclusion, we compared six short read aligners for the purpose of identifying an aligner and parameters that will enable accurate profiling of metagenomic communities for any project that uses large NGS datasets and aims at completing the analysis within a reasonable timeframe. We used a mock community sample of known composition and aligned it against the MMD, which comprises genome sequences of all organisms in the community. Five of the six aligners perform similarly well, with the notable exception of the SOAP aligner, which seemed to detect less coverage in general. The selection of CLC aligner was prompted by several practical factors: i) the ability to handle large databases, ii) its ability to map both paired end and fragment sequence data in a single operation and iii) its speed and small memory footprint. The MAP aligner held a respectable second place, but its lack of support for traditional top random & unique only mapping strategies (at the time of this evaluation) and its inability to map both paired end and fragment reads simultaneously kept it from taking the lead.

Once the best performing aligner was chosen, we focused on identifying appropriate parameters for mapping shotgun metagenomic data. When the database provided the exact strain targets for all reads in the query, we found that that length of alignment constraint had the strongest effect on mapping sensitivity, with the percent identity (considering only two fairly stringent settings) having only a minimal effect. But when swapping out several MMD strains with other organisms from the same genus, the percent similarity setting becomes more important. When the genome of an exact strain present in the metagenomic community is not sequenced (therefore absent from the reference database) but the genome of a close relative is sequenced, having a slightly more lenient similarity cutoff can improve sensitivity at the species or genus level. The suggested parameter settings for profiling microbial community structure using metagenomic shotgun sequences are 80% similarity over 75% length of the query being required to align.

We further explored the issue of mapping resolution and the effects of taxonomic density (i.e. the number of closely related strains available under a species or genus) within the RGD. We considered the cost of the top random mapping strategy in loss of resolution at the strain level to the benefit of being able to map a larger fraction of samples to the genus level. While identifying a larger percentage of samples at a lower resolution might have more immediate value for some applications than correctly identifying a smaller portion of the samples at a finer grained taxonomic level, its of importance to note that by using the unique placement only alignment strategy the capability to map to a greater degree of taxonomic clarity exists. We also showed that the number of conserved strains or species present within a genus both increases the likelihood of correctly identifying the genus of a read, while lessening the likelihood of correctly identifying the exact strain (or species) under the top random mapping strategy. The final Read Mapping Standard Operating Procedure is described in Text S2.

## Methods

### Mock Database Creation

For a number of the analyses described in this paper we used a mock community comprising 20 bacterial and 1 archaeal species, mixed together at different concentrations per strain [20] and sequenced on Illumina GAIIx (100 bp paired-end reads). The Illumina sequences are available in GenBank under Accession: PRJNA48475, ID: 48475. These 20 bacterial and 1 archaeal strains have genome sequence available in GenBank [21]. The fasta sequence of these 21 strains is what is referred to as the ‘Mock Metagenomic Database’ (MMD)(Text S1).

### Reference Database Creation

For the ‘Mapping resolution’ analysis we generated a database comprising archaeal, bacterial, lower eukaryotic and viral organisms available in GenBank, referred to as the ‘Reference Genome Database’ (RGD). These sequences were downloaded via keyword search from the NCBI’s GenBank database on 11/10/2009. The bacterial component underwent special processing as described below, but for the other three superkingdoms, we used the keywords “Archaea[ORGN]”, “Virus[ORGN]” and “Eukaryota[ORGN] NOT Bilateria[ORGN] NOT Streptophyta[ORGN]” (for Archaea, Virus, and lower Eukaryotes respectively), along with the descriptor “complete” and/or “WGS”. All archaeal, viral and lower eukaryotic strains found in that manner were included in the RGD. For the bacterial component of the RGD, a similar keyword search was used, “Bacteria[ORGN] and complete” and “Bacteria[ORGN] and WGS”, followed by removing highly redundant strains that were not part of the HMP. For this redundancy removal step, all sequences from a given genome were first tagged with a prefix unique to that strain. This allows a hit to any component of a draft genome to be easily related back to its parent genome, and was a required step to enable the creation of abundance metrics per genome. The complete and draft genomes were categorized on per species level, resulting in categories including anywhere from single strains to those including many strains per species (e.g. *E. coli* and *Bacillus anthracis*, 57 and 11 strains respectively at the time of the original construction). For selecting representatives among multiple strains within a species, the mauveAligner module of Mauve [22] was wrapped into custom-built PERL scripts to automate most of the process (Figure S1 shows an example mauve alignment). The criterion for exclusion was a similarity of over 90% on a genome-wide level (pair-wise comparisons) and the genome that is longer and provides the most unique sequence was kept. While this process worked well for cases with a small number of strains per species, the challenge grew as the number of sequences increased and the homology decreased among greater numbers of genomic pieces. In some cases many pair-wise alignments were done and the sequences were eliminated progressively. In the case of a large numbers of strains, a slightly relaxed homology (as low as 83%) was used. Bacterial strains that were collected from humans as part of the HMP were retained without being subject to redundancy removal because these strains were deemed informative to the project. Finally plasmids corresponding to the non-redundant genomes that were selected through the above analysis were also incorporated in the database. Figure 1 shows an overview of this process. The RGD fasta database is provided as Text S3, and an index describing the strain-prefix relationship is provided as Text S4.

### Aligner Comparison

Six aligners were tested, BWA [9], CLC [23], MAP [24], SMALT [25], SOAP [10] and Novoalign (www.novocraft.com, unpublished), using roughly default parameters for each program (see Table 1) by aligning 22,735,802 reads generated from the Microbial mock community and sequenced on the Illumina GAIIx against the MMD described above.

Alignments from each aligner were collected using a random top-hit strategy for all programs that supported it (BWA, CLC, SOAP, Novoalign), and the default mapping strategy of the aligner for the others (MAP, SMALT). The top random mapping strategy involves reporting only a single, best hit per query, and in the case of a query having multiple, equally strong best hits (i.e. mapping quality 0 [26]), one of those hits is chosen at random. The MAP aligner supports a novel approach for its mapping strategy where the user sets a ‘topN’ value that sets the maximum number of equally scoring best hits that will be reported. In cases where a query has that many equally scoring best hits or fewer, all hits are reported. If more than those numbers of equal placements are found, no hit is reported. The SMALT aligner only supports 2 mapping strategies. The first reports all hits regardless of the number of tied, best hits, and the default mode is unique only placement, where only queries with a single, best placement are reported.

The breadth (defined as the percentage of covered bases over the length of the reference genome) and depth (defined as the sum of the depths of each covered base divided by the length of the genome) of coverage were calculated based on all alignments of each genome represented in the MMD using a software package called RefCov (http://gmt.genome.wustl.edu/gmt-refcov) and results were compared.

### Parameter Optimization

Parameter optimization was performed only for the aligner that best fulfilled all the required criteria (CLC bio’s CLC Assembly Cell) by varying the minimum similarity setting (-similarity) and the minimum length of alignment setting (-lengthfraction) across 6 different combinations. The tested combinations include: i) 50% length +80% identity (default), ii) 50% length +90% identity, iii) 75% length +80% identity, iv) 75% length +90% identity, v) 100% length +80% identity and vi) 100% length +90% identity. The 64bit version of the CLC Assembly Cell long read alignment program, clc_ref_assemble_long, was used with the parameters “–l <% length> -s <% identity> -p fb ss 180 250” where the –l & -s values were varied as described above. The castosam program was used to extract a sam format file [27] from the cas format output of clc_ref_assemble_long program, and analysis was performed on the sam files. A top random mapping strategy was applied for the parameter tuning analysis, which in the case of multiple, equally strong best mappings, will randomly report one of those mappings as the hit.

### Mapping Resolution Analysis

The effects on mapping accuracy and sensitivity resulting from changing mapping strategies was tested by aligning 22,735,802 illumina GAIIx reads prepared from the mock community against both the RGD and the MMD. CLC Assembly Cell alignments were run using the 64bit version of the program clc_ref_assembly_long with the parameters “–l <% length> -s <% identity> -p fb ss 180 250” where the <% length> + <% identity> settings varied across: i) 50% length +80% identity (default), ii) 50% length +90% identity, iii) 75% length +80% identity, iv) 75% length +90% identity, v) 100% length +80% identity and vi) 100% length +90% identity, and then sam files were produced from alignment outputs as described in the mapping resolution analysis section above, We report the number of hits to the exact mock strains, to the genera represented by those mock strains, to the species represented by those mock strains, false positive organism assignments and those with no hits at all. The hits were reported using both the top random and unique only mapping strategies against both the RGD and the MMD. We also report how many strains were present in the RGD per genera represented in the mock community.

## Supporting Information

Text S1 **Mock Metagenomic community database**
(DOCX)

Text S2 **Read mapping Standard Operating Procedure**
(DOCX)

Text S3 **Reference Genome database**
(DOCX)

Text S4 **Prefix-Strain index for Reference Genome database**
(DOCX)

Figure S1 **Example Mauve alignment.** This picture shows a screenshot of an example Mauve alignment of 8 similar strains of *B. cereus.* Colored blocks show regions of homology between organisms with the amplitude shown within each box showing the strength of the similarity. Lines between strains show smaller regions of homology between sequences.
(TIF)

Figure S2 **Alignment parameter effects on breadth and depth of coverage of original MMD strains. (A). Parameter effects on genome coverage of the MMD at the genus level.** This chart shows the effect of varying the parameters on the coverage of the mock genomes on the genus level. For the genera *Streptococcus* and *Staphylococcus*, which are represented by more than a single strain in the mock community pool, the values are averaged across each member strain. The genus *Pseudomonas*, represented in the mock community by the strain *P. aeruginosa PAO1*, displays a marked decrease in coverage when using the stringent 100% length cutoff. **(B). Parameter effects on genome depth of coverage of the MMD at the genus level.** This chart shows the effect of varying the parameters on the depth of coverage found for the mock genomes on the genus level. For the genera *Streptococcus* and *Staphylococcus*, which are represented by more than a single strain in our mock community pool, the values are averaged across each member strain. The genus *Deinococcus*, which in the mock community is represented by the strain *D. radiodurans* R1, shows a marked decrease in estimated depth of coverage for parameter combinations that require 100% length to align.
(TIF)

Figure S3 **Alignment parameter effects on breadth and depth of coverage of amended MMD strains. (A). Parameter effects on genome coverage of the amended MMD at the genus level.** This chart shows the effect of varying the parameters on the breadth of coverage of the mock genomes on the genus level. The most affected genera are the ones where the strain membership was modified before running the alignments. For *Deinococcus, Escherichia, Helicobacter* and *Neisseria*, the member strain was removed, and a non-mock strain from the same genus was put in its place, and for *Streptococcus*, two of the three strains were removed, leaving the original *S.mutans UA159* strain intact. This figure illustrates that for those genera not having strains present in the mock community, the similarity value begins to have more of an effect on the numbers able to align. **(B). Parameter effects on genome depth of coverage of the amended MMD at the genus level.** This chart shows the effect of varying the parameters on the depth of coverage of the mock genomes on the genus level. The most affected genera are the ones where the strain membership was modified before running the alignments. For *Deinococcus, Escherichia, Helicobacter* and *Neisseria*, the member strain was removed, and a non-mock strain from the same genus was put in its place, and for *Streptococcus*, two of the three strains were removed, leaving the original *S.mutans UA159* strain intact. This figure illustrates that for those genera not having strains present in the mock community, the similarity value begins to have more of an effect on the numbers able to align.
(TIF)
