# Supplementary material for: Optimizing Read Mapping to Reference Genomes to Determine Composition and Species Prevalence in Microbial Communities
Source: PLoS One. 2012 Jun 13;7(6):e36427. doi: 10.1371/journal.pone.0036427 (PMC3374613; doi:10.1371/journal.pone.0036427)
Supplement: Text S1 — Mock Metagenomic community database (DOCX) [file pone.0036427.s001.docx]

**Text S1**. Mock Metagenomic community database

MMD database: The GenBank accession number of the 21 genomes in the MMD are listed below. Several genes are not finished, i.e. are in more multiple pieces, therefore there are more than 21 gi numbers.

gi|126640115

gi|126640097

gi|126640109

gi|148337902

gi|148337903

gi|42740913

gi|44004339

gi|150002608

gi|150014892

gi|15805042

gi|10957530

gi|15807672

gi|10957398

gi|194268102

gi|49175990

gi|15644634

gi|116628683

gi|16802048

gi|148642060

gi|77358697

gi|50841496

gi|110645304

gi|77461965

gi|77464988

gi|125654605

gi|77404592

gi|77404693

gi|77404776

gi|125654693

gi|161508266

gi|161510924

gi|225631039

gi|27466918

gi|32470520

gi|32470532

gi|32470555

gi|32470572

gi|32470581

gi|32470588

gi|22536185

gi|24378532

gi|194172857
