# Supplementary material for: Optimizing Read Mapping to Reference Genomes to Determine Composition and Species Prevalence in Microbial Communities
Source: PLoS One. 2012 Jun 13;7(6):e36427. doi: 10.1371/journal.pone.0036427 (PMC3374613; doi:10.1371/journal.pone.0036427)

**Text S2**. Read mapping Standard Operating Procedure

*Description of the HMP WGS read mapping process*

The process of read mapping to reference genomes for HMP WGS data began with the download of processed data from the DACC FTP site. We used the Aspera high speed file transfer client for this job (The Aspera download client is freely available at "http://www.asperasoft.com/en/products/client_software_2"). Each sample download was comprised of 2 mated pair fastq files, and 1 fragment fastq file. Before further use, each of these 3 files was confirmed valid via md5 fingerprinting.

Fasta was then extracted from each file, and subject to a low-complexity screen using the 'dust' program (distributed with NCBI blast). Dust masks out all bases that have low compositional complexity, and "can eliminate statistically significant but biologically uninteresting reports from the output" (NCBI website blurb @ http://www.ncbi.nlm.nih.gov/BLAST/blastcgihelp.shtml#filter). We considered any read with fewer than 60 non-masked, bases (not necessarily consecutive) to be of low complexity, and thus discarded it from the final set. In cases where one end of a paired end set of mates was found to be of low complexity, and the other end was not, the orphaned (but good quality) read was removed from the paired end file, and moved into the fragment read file.

The processed reads not marked as low-complexity were then aligned to our reference genomes database using the aligner clc_ref_assemble_long (part of the CLC Assembly Cell package available from CLC bio @ <http://www.clcbio.com/>) with the parameters "-lengthfraction 0.75 -similarity 0.8 -p fb ss 180 250" (note: -p fb ss 180 250 sets paired end information, 'fb' indicates that the first read is in the 'f'orward orientation, and the second is in the 'b'ackward orientation (i.e. facing each other), the 'ss 180 250' part informs the program to expect the 's'tart to 's'tart (i.e. the far ends, since they are facing each other) distance between the reads to be from 180-250bp in length). Both the paired end set and the fragment file were aligned in a single execution of the software. The immediate results from the aligner were in CAS format, and were converted into BAM format using the script 'castosam' for downstream analysis (the 'castosam' program is included in the CLC bio aligner ("CLC Assembly Cell") distribution).

The reference database used was comprised of all archaeal, bacterial, lower eukaryote and viral organisms available in GenBank. These sequences were downloaded via keyword search from the NCBI's GenBank on 11/10/2009, and were periodically updated over the course of the project. The archaeal, lower eukaryote and viral components were taken 'as-is' from the keyword searches "Archaea[ORGN]", "Virus[ORGN]" and "Eukaryota[ORGN] NOT Bilateria[ORGN] NOT Streptophyta[ORGN]" respectively. But the bacterial component was subject to special processing to remove highly redundant strains that were not part of the HMP. We started with a similar keyword search, "Bacteria[ORGN] and complete" and "Bacteria[ORGN] and WGS", and then we subjected them to a process in which we try to remove highly redundant strains that were not part of the HMP. Contigs were grouped into their respective genomes based on their GenBank ID ranges in random order. To assist with downstream identifications, all sequences from a given genome were tagged with a prefix id unique to that strain. This allows a hit to any contig in a draft genome to be easily related back to its parent genome, and was a required step to enable the creation of abundance metrics per genome. The complete and draft genomes were categorized on per species level, resulting in categories including single strain up to over 50 strains per species (e.g. E. coli and B. anthracis). Redundancy removal was implemented to exclude strains with nearly identical sequences. For selecting representatives among multiple strains within a species, we used the Mauve program (Darling, Aaron C.E., Mau, Bob, Blattner, Frederick R., and Perna, Nicole T. "Mauve: Multiple Alignment of Conserved Genomic Sequence With Rearrangements", Genome Research 2004, 14:1394-1403). The mauveAligner module of Mauve program was wrapped into custom-built PERL scripts to automate most of the process (Figure 1 shows an example mauve alignment). Our criteria was simple, if there was more than 90% similarity between two genomes, we would pick the longer one. Mauve worked well for the smaller number of genomes that were in one or very few sequences, however the challenges grew when the number of sequences increased and as the homology decreased among greater numbers of genomic pieces. In some of cases many pair-wise alignments were done and the sequences were eliminated progressively. In cases of large numbers of strains, a slightly relaxed homology (as low as 82-83%) was used. Another filter that was also added was to check if a given strain was a human originated strain (i.e. part of the HMP project). Because our focus is on the human microbiome, human originated references were excepted from the removal process. Finally, plasmids corresponding to the non-redundant genomes that were selected through the above analysis were added in. (Figure 2 shows a flow-diagram of the process of creating the reference database). The final reference database that was used in the analysis for this paper contained 1751 bacterial strains spread over 1253 species. The other components of the database covered: i) Archaea: 131 strains over 97 species, ii) Lower eukaryotes: 326 strains over 326 species and iii) Virus: 3683 strains over 1420 species. The process of removing highly redundant bacterial strains resulted in the elimination of 2265 complete genomes, draft genomes, and plasmid sequences.

After mapping to the described reference genomes database, minimal metrics were collected using samtools flagstats. We harvested the total number of reads passing our low-complexity filter and the total number of reads mapping to the database. In total we attempted to map 38,415,726,108 high quality microbial reads, known to be free of low-complexity sequence, spread over 754 samples, against our reference database. And we found 22,113,712,465 hits (~57.6%) using our alignment cutoff of 80% identity over 75% of the length of the query. Additionally we built strain abundance tables on a per-sample basis, showing the depth and breadth of coverage of each strain in our database. We reported all strains meeting a coverage cutoff of 0.01x depth across 1% breadth of each strain's reference genome. Initial coverage statistics were generated using the Ref-Cov program (internal, TGI software by Todd Wylie & Jason Walker), and the coverage per each sequence record was collapsed such that we had a cumulative depth and breadth of coverage for each strain in the reference database (many draft strains are comprised of multiple contigs that needed to be collapsed & considered as a single organismal entity for the final report). Figure 3 shows an overview of the entire read mapping process.

The mapping bam files, minimal metrics files and sample abundance tables (along with md5 fingerprints for download validation) are all available on the DACC FTP site under the directory:

/WholeMetagenomic/05-Analysis/ReadMappingToReference/SRS*

**Figures**

**Figure 1.**


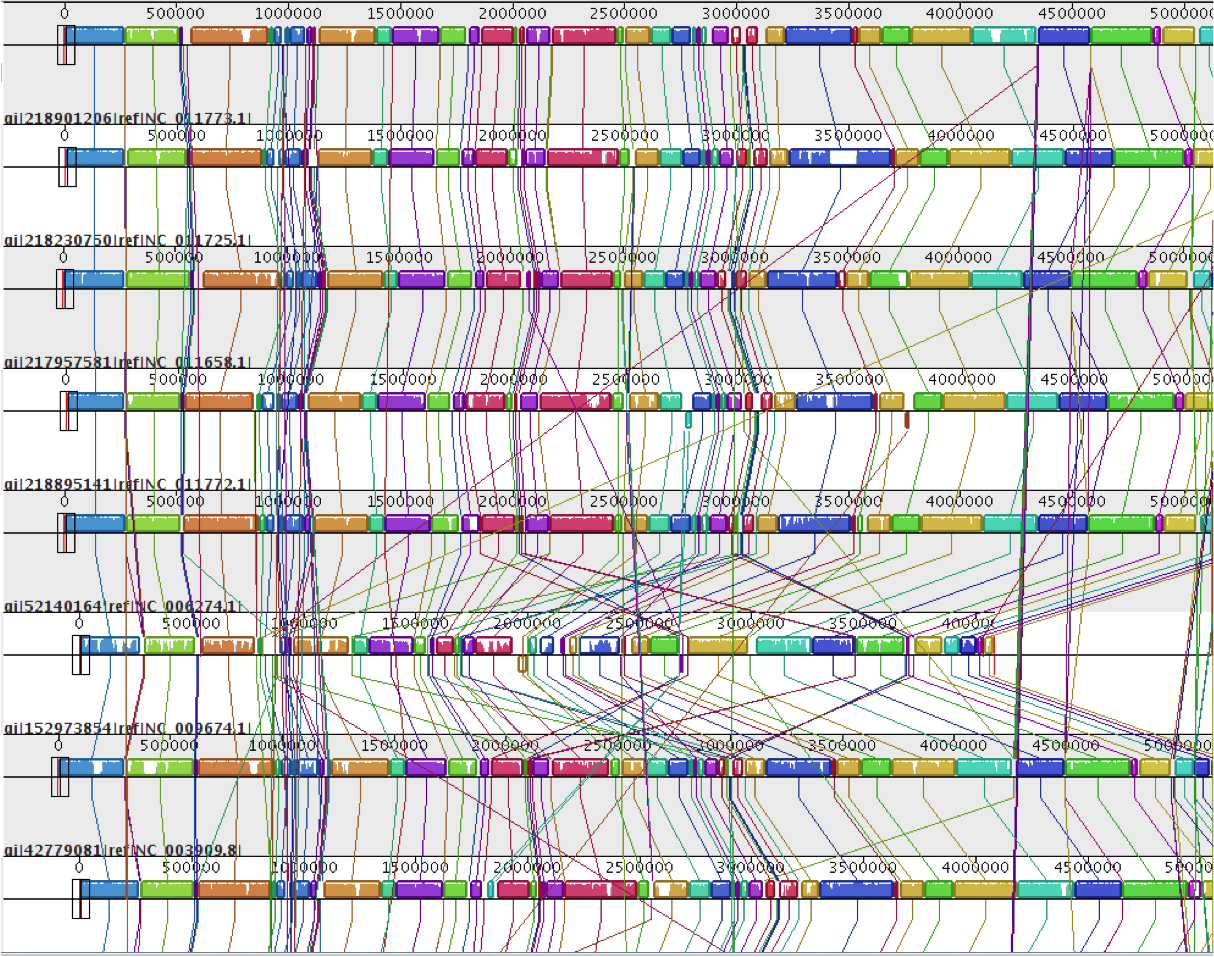


**Figure 2.**


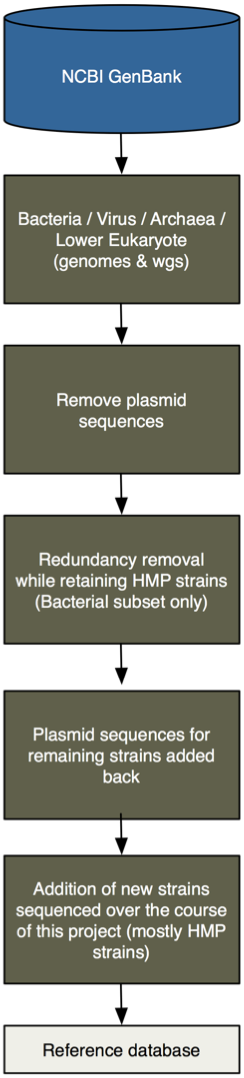


**Figure 3.**


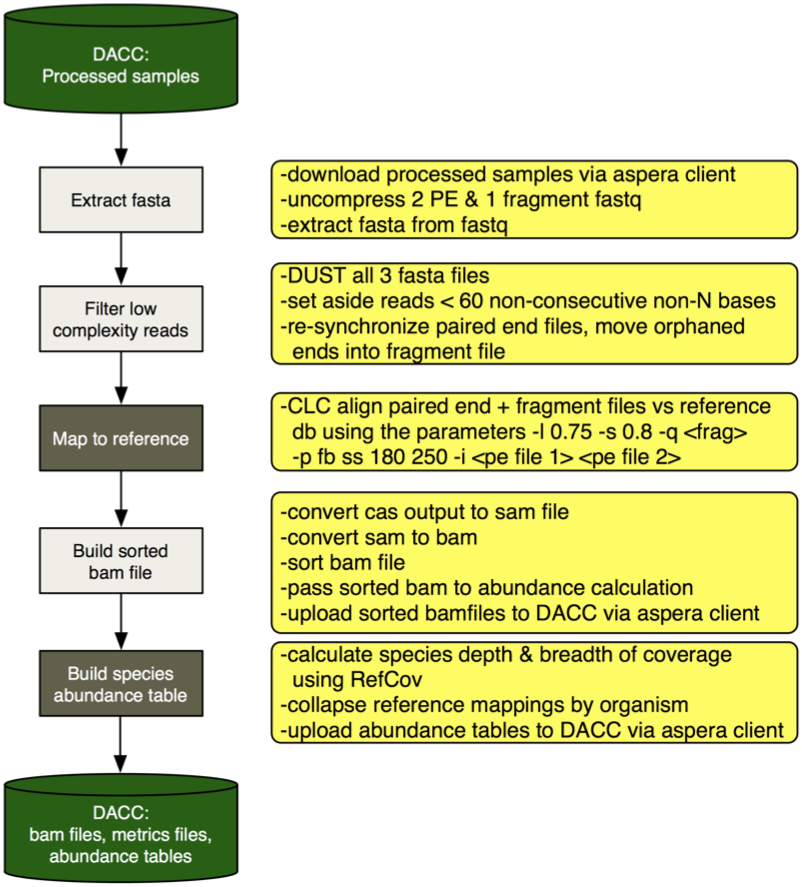

Supplement: Text S2 — Read mapping Standard Operating Procedure (DOCX) [file pone.0036427.s002.docx]
