# Supplementary material for: Optimizing Read Mapping to Reference Genomes to Determine Composition and Species Prevalence in Microbial Communities
Source: PLoS One. 2012 Jun 13;7(6):e36427. doi: 10.1371/journal.pone.0036427 (PMC3374613; doi:10.1371/journal.pone.0036427)
Supplement: Text S3 — Reference Genome database (DOCX) [file pone.0036427.s003.docx]

**Text S3**. Reference Genome Database (RGD)

The 7.3Gb RGD is now available on the Washington University Genome Institute FTP site at:

<ftp://genome.wustl.edu/pub/supplemental/jmartin_plos_one_2011/Martin_etal_TextS3_13Dec2011.fasta>
