# Supplementary material for: Optimizing Read Mapping to Reference Genomes to Determine Composition and Species Prevalence in Microbial Communities
Source: PLoS One. 2012 Jun 13;7(6):e36427. doi: 10.1371/journal.pone.0036427 (PMC3374613; doi:10.1371/journal.pone.0036427)
Supplement: Text S4 — Prefix-Strain index for Reference Genome database (DOCX) [file pone.0036427.s004.docx]

**Text S4**. Prefix-Strain index for Reference Genome database

Species_id Species_name

BACT_628 Helicobacter hepaticus ATCC 51449

BACT_699 Leptothrix cholodnii SP-6

BACT_1513 Streptococcus oralis ATCC 35037

BACT_220 Bifidobacterium longum subsp. infantis CCUG 52486

BACT_1292 Acidovorax avenae subsp. avenae ATCC 19860

BACT_800 Natranaerobius thermophilus JW/NM-WN-LF

BACT_15 Acidothermus cellulolyticus 11B

BACT_1622 Rhodobacter capsulatus SB 1003

BACT_749 Methylobacterium extorquens AM1

BACT_1283 Zymomonas mobilis subsp. mobilis NCIB 11163

BACT_1106 Streptococcus agalactiae H36B

BACT_669 Lactobacillus gasseri 202-4

BACT_1394 Frankia sp. EuI1c

BACT_1347 Edwardsiella tarda ATCC 23685

BACT_40 Actinomyces urogenitalis DSM 15434

BACT_1253 Wolbachia endosymbiont of Muscidifurax uniraptor

BACT_815 Nitrobacter hamburgensis X14

BACT_268 Burkholderia cenocepacia MC0-3

BACT_664 Lactobacillus coleohominis 101-4-CHN

BACT_1033 Salmonella enterica subsp. enterica serovar Agona str. SL483

BACT_1169 Teredinibacter turnerae T7901

BACT_1486 Staphylococcus aureus A10102

BACT_676 Lactobacillus jensenii 27-2-CHN

BACT_534 Escherichia sp. 4_1_40B

BACT_532 Escherichia fergusonii ATCC 35469

BACT_608 Gluconacetobacter xylinus NBRC 3288

BACT_877 Photobacterium profundum SS9

BACT_85 Arthrospira maxima CS-328

BACT_1362 Escherichia coli B088

BACT_331 Carnobacterium sp. AT7

BACT_1332 Candidatus Poribacteria sp. WGA-A3

BACT_1146 Sulfurovum sp. NBC37-1

BACT_1176 Thermoanaerobacter mathranii subsp. mathranii str. A3

BACT_505 Elusimicrobium minutum Pei191

BACT_1546 Arcobacter nitrofigilis DSM 7299

BACT_184 Bacteroides sp. 1_1_6

BACT_116 Bacillus cereus BDRD-ST196

BACT_652 Kingella oralis ATCC 51147

BACT_1337 Clostridium difficile NAP08

BACT_1466 Pseudomonas savastanoi pv. savastanoi NCPPB 3335

BACT_547 Fervidobacterium nodosum Rt17-B1

BACT_1571 Coraliomargarita akajimensis DSM 45221

BACT_283 Caldicellulosiruptor saccharolyticus DSM 8903

BACT_132 Bacillus cereus Rock1-3

BACT_1072 Shuttleworthia satelles DSM 14600

BACT_898 Prevotella veroralis F0319

BACT_245 Bradyrhizobium sp. ORS278

BACT_645 Janibacter sp. HTCC2649

BACT_23 Acinetobacter baumannii AYE

BACT_1445 Parachlamydia acanthamoebae str. Hall's coccus

BACT_1584 Erwinia amylovora CFBP1430

BACT_1142 Sulfurihydrogenibium sp. YO3AOP1

BACT_1413 Lactobacillus gasseri 224-1

BACT_1516 Streptococcus pneumoniae str. Canada MDR_19F

BACT_688 Lactococcus lactis subsp. lactis Il1403

BACT_167 Bacillus thuringiensis serovar tochigiensis BGSC 4Y1

BACT_625 Helicobacter bilis ATCC 43879

BACT_719 Loktanella vestfoldensis SKA53

BACT_922 Pseudomonas entomophila L48

BACT_913 Proteus penneri ATCC 35198

BACT_552 Flavobacteria bacterium MS024-3C

BACT_160 Bacillus thuringiensis serovar kurstaki str. T03a001

BACT_936 Pseudomonas syringae pv. syringae B728a

BACT_780 Mycobacterium smegmatis str. MC2 155

BACT_1496 Staphylococcus aureus subsp. aureus C101

BACT_89 Atopobium parvulum DSM 20469

BACT_1494 Staphylococcus aureus subsp. aureus ATCC 51811

BACT_1231 Vibrio harveyi ATCC BAA-1116

BACT_520 Erwinia carotovora subsp. atroseptica SCRI1043

BACT_1518 Streptococcus suis 05HAS68

BACT_1519 Streptomyces clavuligerus ATCC 27064

BACT_685 Lactobacillus ultunensis DSM 16047

BACT_1162 Synechococcus sp. WH 7805

BACT_728 Mannheimia succiniciproducens MBEL55E

BACT_704 Limnobacter sp. MED105

BACT_661 Lactobacillus antri DSM 16041

BACT_395 Clostridium leptum DSM 753

BACT_1021 Ruminococcus gnavus ATCC 29149

BACT_725 Magnetospirillum magneticum AMB-1

BACT_216 Bifidobacterium gallicum DSM 20093

BACT_743 Mesorhizobium opportunistum WSM2075

BACT_269 Burkholderia dolosa AUO158

BACT_1291 Acidobacterium sp. MP5ACTX9

BACT_312 Candidatus Desulforudis audaxviator MP104C

BACT_333 Catenulispora acidiphila DSM 44928

BACT_1535 Xanthomonas fuscans subsp. aurantifolii str. ICPB 11122

BACT_1424 Lysinibacillus fusiformis ZC1

BACT_1158 Synechococcus sp. RS9916

BACT_558 Francisella tularensis subsp. novicida U112

BACT_1591 Herbaspirillum seropedicae SmR1

BACT_807 Neisseria meningitidis MC58

BACT_365 Citrobacter koseri ATCC BAA-895

BACT_1260 Xanthomonas campestris pv. musacearum NCPPB4381

BACT_1544 Anaplasma centrale str. Israel

BACT_1035 Salmonella enterica subsp. enterica serovar Dublin str. CT_02021853

BACT_982 Rhodopseudomonas palustris HaA2

BACT_1320 Bacteroides vulgatus PC510

BACT_1363 Escherichia coli B185

BACT_891 Porphyromonas uenonis 60-3

BACT_349 Chlorobaculum parvum NCIB 8327

BACT_1154 Synechococcus sp. JA-3-3Ab

BACT_944 Psychrobacter sp. PRwf-1

BACT_1271 Yersinia intermedia ATCC 29909

BACT_1218 Vibrio alginolyticus 12G01

BACT_787 Mycoplasma arthritidis 158L3-1

BACT_195 Bacteroides thetaiotaomicron VPI-5482

BACT_940 Pseudomonas syringae pv. tomato T1

BACT_17 Acidovorax delafieldii 2AN

BACT_638 Hoeflea phototrophica DFL-43

BACT_1617 Prevotella ruminicola 23

BACT_1128 Streptomyces griseus subsp. griseus NBRC 13350

BACT_1373 Escherichia coli MS 175-1

BACT_1188 Thermosipho africanus TCF52B

BACT_193 Bacteroides dorei 5_1_36/D4

BACT_1004 Roseiflexus sp. RS-1

BACT_1463 Propionibacterium acnes J165

BACT_2 Burkholderia pseudomallei 305

BACT_1343 Cylindrospermopsis raciborskii CS-505

BACT_909 Prochlorococcus marinus subsp. pastoris str. CCMP1986

BACT_83 Arthrobacter chlorophenolicus A6

BACT_757 Methylophaga thiooxidans DMS010

BACT_738 Mariprofundus ferrooxydans PV-1

BACT_513 Enterococcus faecalis HH22

BACT_1582 Enterobacter cloacae subsp. cloacae ATCC 13047

BACT_34 Actinobacillus pleuropneumoniae serovar 1 str. 4074

BACT_1258 Xanthomonas axonopodis pv. citri str. 306

BACT_1209 Uncultured Termite group 1 bacterium phylotype Rs-D17

BACT_1259 Xanthomonas campestris pv. campestris str. 8004

BACT_370 Clostridium acetobutylicum ATCC 824

BACT_1217 Verrucomicrobium spinosum DSM 4136

BACT_618 Haemophilus influenzae R2866

BACT_3 Hydrogenivirga sp. 128-5-R1-1

BACT_736 Marinobacter aquaeolei VT8

BACT_867 Pelobacter propionicus DSM 2379

BACT_1206 Trichodesmium erythraeum IMS101

BACT_612 Granulicatella adiacens ATCC 49175

BACT_957 Rhizobium etli 8C-3

BACT_794 Mycoplasma penetrans HF-2

BACT_660 Labrenzia alexandrii DFL-11

BACT_1334 Chlamydia trachomatis L2tet1

BACT_1078 Slackia heliotrinireducens DSM 20476

BACT_1493 Staphylococcus aureus subsp. aureus A017934/97

BACT_659 Labrenzia aggregata IAM 12614

BACT_298 Campylobacter upsaliensis RM3195

BACT_905 Prochlorococcus marinus str. MIT 9313

BACT_347 Chlamydophila felis Fe/C-56

BACT_845 Orientia tsutsugamushi str. Ikeda

BACT_1615 Pantoea ananatis LMG 20103

BACT_943 Psychrobacter cryohalolentis K5

BACT_1639 Streptococcus mutans NN2025

BACT_729 Maricaulis maris MCS10

BACT_178 Bacteroides finegoldii DSM 17565

BACT_1560 Candidatus Nitrospira defluvii

BACT_833 Oceanicola granulosus HTCC2516

BACT_1618 Propionibacterium acnes SK137

BACT_510 Enterobacter sp. 638

BACT_155 Bacillus thuringiensis IBL 4222

BACT_92 Aurantimonas manganoxydans SI85-9A1

BACT_1558 Brevundimonas subvibrioides ATCC 15264

BACT_1553 Bacillus tusciae DSM 2912

BACT_402 Clostridium perfringens C str. JGS1495

BACT_1572 Corynebacterium pseudotuberculosis FRC41

BACT_1372 Escherichia coli MS 146-1

BACT_173 Bacteroides cellulosilyticus DSM 14838

BACT_1491 Staphylococcus aureus A9765

BACT_487 Dialister invisus DSM 15470

BACT_418 Collinsella aerofaciens ATCC 25986

BACT_1652 Yersinia pestis Z176003

BACT_1029 Salinibacter ruber DSM 13855

BACT_105 Bacillus cereus AH187

BACT_239 Borrelia valaisiana VS116

BACT_1442 Neisseria sp. oral taxon 014 str. F0314

BACT_465 Desulfatibacillum alkenivorans AK-01

BACT_310 Candidatus Cloacamonas acidaminovorans

BACT_468 Desulfohalobium retbaense DSM 5692

BACT_1353 Enterococcus faecium D344SRF

BACT_191 Bacteroides sp. 4_3_47FAA

BACT_745 Methylacidiphilum infernorum V4

BACT_727 Mannheimia haemolytica serotype A2 str. OVINE

BACT_947 Psychromonas sp. CNPT3

BACT_1081 Sorangium cellulosum 'So ce 56'

BACT_986 Rickettsia africae ESF-5

BACT_951 Ralstonia pickettii 12J

BACT_1404 Gluconacetobacter hansenii ATCC 23769

BACT_998 Rickettsia typhi str. Wilmington

BACT_1427 Methylosinus trichosporium OB3b

BACT_1126 Streptomyces ghanaensis ATCC 14672

BACT_564 Fusobacterium mortiferum ATCC 9817

BACT_1642 Thermincola sp. JR

BACT_147 Bacillus pseudomycoides DSM 12442

BACT_616 Haemophilus influenzae 22.4-21

BACT_479 Desulfovibrio vulgaris RCH1

BACT_1550 Bacillus megaterium QM B1551

BACT_231 Borrelia burgdorferi 80a

BACT_859 Pasteuria nishizawae str. North American

BACT_376 Clostridium botulinum B str. Eklund 17B

BACT_720 Lutiella nitroferrum 2002

BACT_717 Listeria monocytogenes str. 4b H7858

BACT_551 Flavobacteria bacterium MS024-2A

BACT_624 Helicobacter acinonychis str. Sheeba

BACT_1135 Streptomyces sp. SPB74

BACT_765 Microscilla marina ATCC 23134

BACT_1448 Peptoniphilus lacrimalis 315-B

BACT_1046 Salmonella enterica subsp. enterica serovar Typhimurium str. LT2

BACT_215 Bifidobacterium dentium ATCC 27678

BACT_967 Rhodobacter sp. SW2

BACT_98 Bacillus cereus 03BB102

BACT_824 Nocardioides sp. JS614

BACT_1191 Thermotoga lettingae TMO

BACT_1160 Synechococcus sp. WH 5701

BACT_927 Pseudomonas putida F1

BACT_252 Brucella ceti str. Cudo

BACT_1415 Lactobacillus jensenii 208-1

BACT_299 Campylobacterales bacterium GD 1

BACT_1457 Prevotella melaninogenica D18

BACT_364 Citreicella sp. SE45

BACT_1501 Staphylococcus aureus subsp. aureus H19

BACT_747 Methylobacillus flagellatus KT

BACT_503 Ehrlichia ruminantium str. Welgevonden

BACT_480 Desulfovibrio vulgaris str. 'Miyazaki F'

BACT_164 Bacillus thuringiensis serovar pulsiensis BGSC 4CC1

BACT_1623 Rhodothermus marinus DSM 4252

BACT_916 Providencia rustigianii DSM 4541

BACT_270 Burkholderia graminis C4D1M

BACT_662 Lactobacillus buchneri ATCC 11577

BACT_1314 Bacteroides sp. 20_3

BACT_853 Parabacteroides sp. D13

BACT_278 Burkholderia thailandensis MSMB43

BACT_590 Geobacillus sp. WCH70

BACT_1083 Sphingobacterium spiritivorum ATCC 33300

BACT_52 Algoriphagus sp. PR1

BACT_1489 Staphylococcus aureus A8819

BACT_649 Jonquetella anthropi E3_33 E1

BACT_444 Cronobacter turicensis

BACT_1032 Salmonella enterica subsp. arizonae serovar 62:z4,z23:--

BACT_541 Eubacterium siraeum DSM 15702

BACT_1164 Synechococcus sp. WH 8109

BACT_62 Anabaena variabilis ATCC 29413

BACT_488 Acidovorax ebreus TPSY

BACT_1299 Acinetobacter sp. 6014059

BACT_427 Corynebacterium accolens ATCC 49725

BACT_362 Chryseobacterium gleum ATCC 35910

BACT_1214 Veillonella parvula DSM 2008

BACT_315 Candidatus Koribacter versatilis Ellin345

BACT_1495 Staphylococcus aureus subsp. aureus Btn1260

BACT_542 Eubacterium ventriosum ATCC 27560

BACT_1052 Serratia proteamaculans 568

BACT_241 Brachyspira hyodysenteriae WA1

BACT_1268 Yersinia bercovieri ATCC 43970

BACT_760 Methylovorus sp. SIP3-4

BACT_605 Glaciecola sp. HTCC2999

BACT_566 Fusobacterium nucleatum subsp. nucleatum ATCC 25586

BACT_1000 Robiginitalea biformata HTCC2501

BACT_499 Eggerthella lenta DSM 2243

BACT_1414 Lactobacillus iners AB-1

BACT_693 Legionella pneumophila str. Corby

BACT_1593 Klebsiella variicola At-22

BACT_572 Fusobacterium sp. 3_1_5R

BACT_1194 Thermotoga neapolitana DSM 4359

BACT_665 Lactobacillus crispatus MV-3A-US

BACT_960 Rhizobium etli CIAT 894

BACT_881 Planctomyces limnophilus DSM 3776

BACT_679 Lactobacillus plantarum WCFS1

BACT_1235 Vibrio orientalis CIP 102891

BACT_1168 Syntrophus aciditrophicus SB

BACT_176 Bacteroides dorei DSM 17855

BACT_452 Cyanothece sp. PCC 7822

BACT_1594 Lactobacillus casei str. Zhang

BACT_1658 Hydrogenobaculum sp. SN

BACT_1010 Roseobacter sp. MED193

BACT_1460 Prevotella sp. oral taxon 317 str. F0108

BACT_1265 Xylanimonas cellulosilytica DSM 15894

BACT_1105 Streptococcus agalactiae COH1

BACT_565 Fusobacterium nucleatum subsp. polymorphum ATCC 10953

BACT_902 Prochlorococcus marinus str. MIT 9301

BACT_764 Micromonospora sp. ATCC 39149

BACT_43 Aeromonas hydrophila subsp. hydrophila ATCC 7966

BACT_1208 Tsukamurella paurometabola DSM 20162

BACT_545 Faecalibacterium prausnitzii A2-165

BACT_511 Enterococcus casseliflavus EC10

BACT_598 Geobacter metallireducens GS-15

BACT_1384 Escherichia coli MS 84-1

BACT_1371 Escherichia coli MS 124-1

BACT_1429 Micromonospora sp. L5

BACT_850 Parabacteroides distasonis ATCC 8503

BACT_1329 Burkholderia sp. Ch1-1

BACT_973 Rhodobacterales bacterium HTCC2654

BACT_619 Haemophilus somnus 2336

BACT_600 Geobacter sp. M18

BACT_1071 Shigella sp. D9

BACT_460 Deinococcus deserti VCD115

BACT_146 Bacillus mycoides Rock1-4

BACT_1047 Sanguibacter keddieii DSM 10542

BACT_72 Anaeromyxobacter sp. Fw109-5

BACT_539 Eubacterium rectale ATCC 33656

BACT_346 Chlamydophila caviae GPIC

BACT_901 Prochlorococcus marinus str. MIT 9215

BACT_1161 Synechococcus sp. WH 7803

BACT_533 Escherichia sp. 3_2_53FAA

BACT_340 Chlamydia muridarum Nigg

BACT_1091 Staphylococcus capitis SK14

BACT_143 Bacillus halodurans C-125

BACT_121 Bacillus cereus F65185

BACT_309 Candidatus Carsonella ruddii PV

BACT_762 Microcoleus chthonoplastes PCC 7420

BACT_1409 Lactobacillus amylolyticus DSM 11664

BACT_1204 Treponema pallidum subsp. pallidum SS14

BACT_177 Bacteroides eggerthii DSM 20697

BACT_1183 Thermobifida fusca YX

BACT_360 Chromobacterium violaceum ATCC 12472

BACT_1382 Escherichia coli MS 69-1

BACT_244 Bradyrhizobium sp. BTAi1

BACT_311 Candidatus Desulfococcus oleovorans Hxd3

BACT_46 Aggregatibacter aphrophilus NJ8700

BACT_1068 Shigella boydii CDC 3083-94

BACT_1118 Streptococcus sp. 2_1_36FAA

BACT_304 Candidatus Accumulibacter phosphatis clade IIA str. UW-1

BACT_1002 Roseburia inulinivorans DSM 16841

BACT_172 Bacteroides capillosus ATCC 29799

BACT_144 Bacillus licheniformis ATCC 14580

BACT_1359 Enterococcus faecium E980

BACT_288 Campylobacter gracilis RM3268

BACT_1113 Streptococcus mutans UA159

BACT_445 Cryptobacterium curtum DSM 15641

BACT_1511 Staphylococcus epidermidis M23864:W2(grey)

BACT_1309 Bacillus cereus SJ1

BACT_1318 Bacteroides sp. D22

BACT_956 Renibacterium salmoninarum ATCC 33209

BACT_928 Pseudomonas putida GB-1

BACT_819 Nitrosomonas europaea ATCC 19718

BACT_470 Desulfonatronospira thiodismutans ASO3-1

BACT_413 Clostridium sp. SS2/1

BACT_1182 Thermobaculum terrenum ATCC BAA-798

BACT_237 Borrelia recurrentis A1

BACT_1266 Xylella fastidiosa Dixon

BACT_151 Bacillus sp. SG-1

BACT_682 Lactobacillus rhamnosus LMS2-1

BACT_734 Marinitoga piezophila KA3

BACT_1278 Yersinia pestis Pestoides A

BACT_99 Bacillus cereus 03BB108

BACT_1175 Thermoanaerobacter italicus Ab9

BACT_157 Bacillus thuringiensis serovar berliner ATCC 10792

BACT_684 Lactobacillus sakei subsp. sakei 23K

BACT_345 Chlamydophila abortus S26/3

BACT_1007 Roseobacter sp. AzwK-3b

BACT_1155 Synechococcus sp. PCC 7002

BACT_1300 Acinetobacter sp. SH024

BACT_818 Nitrococcus mobilis Nb-231

BACT_379 Clostridium botulinum Bf

BACT_77 Anaplasma phagocytophilum HZ

BACT_1446 Parascardovia denticolens F0305

BACT_78 Anoxybacillus flavithermus WK1

BACT_642 Hyphomonas neptunium ATCC 15444

BACT_634 Heliobacterium modesticaldum Ice1

BACT_253 Brucella melitensis ATCC 23457

BACT_1103 Streptococcus agalactiae 515

BACT_1523 Streptomyces sp. e14

BACT_1195 Thermotoga petrophila RKU-1

BACT_857 Pasteurella dagmatis ATCC 43325

BACT_410 Clostridium sp. 7_2_43FAA

BACT_972 Rhodobacterales bacterium HTCC2255

BACT_507 Enhydrobacter aerosaccus SK60

BACT_407 Clostridium phytofermentans ISDg

BACT_1438 Mycoplasma alligatoris A21JP2

BACT_10 Acidimicrobium ferrooxidans DSM 10331

BACT_1338 Clostridium lentocellum DSM 5427

BACT_213 Bifidobacterium breve DSM 20213

BACT_1326 Bulleidia extructa W1219

BACT_1143 Sulfurihydrogenibium yellowstonense SS-5

BACT_1203 Treponema denticola ATCC 35405

BACT_504 Eikenella corrodens ATCC 23834

BACT_1619 Propionibacterium freudenreichii subsp. shermanii CIRM-BIA1

BACT_1498 Staphylococcus aureus subsp. aureus C427

BACT_1335 Citromicrobium bathyomarinum JL354

BACT_175 Bacteroides coprophilus DSM 18228

BACT_517 Enterococcus faecium TX1330

BACT_1564 Caulobacter segnis ATCC 21756

BACT_1428 Micrococcus luteus SK58

BACT_14 Acidobacterium capsulatum ATCC 51196

BACT_1243 Vibrio splendidus LGP32

BACT_1396 Frankia symbiont of Datisca glomerata

BACT_1441 Neisseria polysaccharea ATCC 43768

BACT_1102 Streptococcus agalactiae 18RS21

BACT_870 Pelotomaculum thermopropionicum SI

BACT_148 Bacillus selenitireducens MLS10

BACT_723 Macrococcus caseolyticus JCSC5402

BACT_1013 Roseovarius sp. 217

BACT_653 Klebsiella pneumoniae subsp. rhinoscleromatis ATCC 13884

BACT_1245 Vibrionales bacterium SWAT-3

BACT_1199 Thiobacillus denitrificans ATCC 25259

BACT_1407 Klebsiella sp. 1_1_55

BACT_130 Bacillus cereus R309803

BACT_264 Burkholderia ambifaria IOP40-10

BACT_467 Desulfobacterium autotrophicum HRM2

BACT_1149 Synechococcus sp. BL107

BACT_548 Fibrobacter succinogenes subsp. succinogenes S85

BACT_1541 Aminobacterium colombiense DSM 12261

BACT_767 Mobiluncus curtisii ATCC 43063

BACT_1229 Vibrio fischeri MJ11

BACT_68 Anaerococcus tetradius ATCC 35098

BACT_214 Bifidobacterium catenulatum DSM 16992

BACT_1608 Moraxella catarrhalis RH4

BACT_458 Dehalococcoides sp. CBDB1

BACT_38 Actinomyces odontolyticus ATCC 17982

BACT_1093 Staphylococcus epidermidis RP62A

BACT_1144 Sulfurimonas denitrificans DSM 1251

BACT_578 Fusobacterium varium ATCC 27725

BACT_528 Escherichia coli 83972

BACT_713 Listeria monocytogenes FSL R2-503

BACT_826 Nodularia spumigena CCY9414

BACT_434 Corynebacterium lipophiloflavum DSM 44291

BACT_1123 Streptomyces avermitilis MA-4680

BACT_69 Anaerococcus vaginalis ATCC 51170

BACT_945 Psychroflexus torquis ATCC 700755

BACT_140 Bacillus clausii KSM-K16

BACT_1181 Thermoanaerobacterium thermosaccharolyticum DSM 571

BACT_630 Helicobacter pylori 98-10

BACT_482 Desulfovibrio vulgaris subsp. vulgaris str. Hildenborough

BACT_1324 Brevibacterium mcbrellneri ATCC 49030

BACT_1184 Thermodesulfovibrio yellowstonii DSM 11347

BACT_1500 Staphylococcus aureus subsp. aureus EMRSA16

BACT_593 Geobacillus sp. Y412MC52

BACT_1174 Thermoanaerobacter ethanolicus CCSD1

BACT_615 Haemophilus ducreyi 35000HP

BACT_921 Pseudomonas aeruginosa LESB58

BACT_481 Desulfovibrio vulgaris subsp. vulgaris DP4

BACT_991 Rickettsia endosymbiont of Ixodes scapularis

BACT_1242 Vibrio splendidus 12B01

BACT_849 Pantoea sp. At-9b

BACT_56 Alkaliphilus oremlandii OhILAs

BACT_1577 Dehalogenimonas lykanthroporepellens BL-DC-9

BACT_1008 Roseobacter sp. CCS2

BACT_1509 Staphylococcus aureus subsp. aureus WBG10049

BACT_657 Kribbella flavida DSM 17836

BACT_136 Bacillus cereus Rock4-18

BACT_810 Neisseria subflava NJ9703

BACT_524 Erythrobacter litoralis HTCC2594

BACT_1610 Mycoplasma crocodyli MP145

BACT_400 Clostridium perfringens ATCC 13124

BACT_359 Chloroherpeton thalassium ATCC 35110

BACT_1062 Shewanella piezotolerans WP3

BACT_834 Oceanobacillus iheyensis HTE831

BACT_343 Chlamydia muridarum Weiss

BACT_76 Anaplasma marginale str. Florida

BACT_1247 Weissella paramesenteroides ATCC 33313

BACT_373 Clostridium beijerinckii NCIMB 8052

BACT_1281 Yersinia ruckeri ATCC 29473

BACT_848 Paenibacillus larvae subsp. larvae BRL-230010

BACT_1443 Oenococcus oeni AWRIB429

BACT_802 Neisseria cinerea ATCC 14685

BACT_768 Mobiluncus mulieris ATCC 35243

BACT_306 Candidatus Azobacteroides pseudotrichonymphae genomovar. CFP2

BACT_225 Blautia hydrogenotrophica DSM 10507

BACT_255 Brucella neotomae 5K33

BACT_16 Acidovorax avenae subsp. citrulli AAC00-1

BACT_822 Nitrosospira multiformis ATCC 25196

BACT_1272 Yersinia kristensenii ATCC 33638

BACT_1651 Xenorhabdus nematophila ATCC 19061

BACT_251 Brucella ceti M644/93/1

BACT_1411 Lactobacillus crispatus 214-1

BACT_206 Beijerinckia indica subsp. indica ATCC 9039

BACT_457 Dehalococcoides ethenogenes 195

BACT_1084 Sphingomonas sp. SKA58

BACT_203 Bdellovibrio bacteriovorus HD100

BACT_1317 Bacteroides sp. D20

BACT_1219 Vibrio cholera CIRS 101

BACT_1628 Shewanella violacea DSS12

BACT_104 Bacillus cereus AH1273

BACT_843 Oribacterium sp. oral taxon 078 str. F0262

BACT_483 Desulfurivibrio alkaliphilus AHT2

BACT_437 Corynebacterium striatum ATCC 6940

BACT_280 Burkholderia vietnamiensis G4

BACT_984 Rhodospirillum centenum SW

BACT_756 Methylococcus capsulatus str. Bath

BACT_112 Bacillus cereus ATCC 14579

BACT_1316 Bacteroides sp. 3_1_23

BACT_512 Enterococcus faecalis ATCC 29200

BACT_804 Neisseria flavescens SK114

BACT_436 Corynebacterium pseudogenitalium ATCC 33035

BACT_964 Rhizobium leguminosarum bv. trifolii WSM1325

BACT_1583 Erwinia amylovora ATCC 49946

BACT_1100 Stigmatella aurantiaca DW4/3-1

BACT_1226 Vibrio cholerae NCTC 8457

BACT_234 Borrelia garinii PBi

BACT_1186 Thermomonospora curvata DSM 43183

BACT_702 Leptotrichia hofstadii F0254

BACT_204 Beggiatoa sp. PS

BACT_19 Acinetobacter baumannii AB0057

BACT_895 Prevotella oris F0302

BACT_908 Prochlorococcus marinus subsp. marinus str. CCMP1375

BACT_799 Nakamurella multipartita DSM 44233

BACT_198 Bartonella grahamii as4aup

BACT_412 Clostridium sp. M62/1

BACT_1020 Ruminococcus flavefaciens FD-1

BACT_1444 Oscillatoria sp. PCC 6506

BACT_823 Nocardia farcinica IFM 10152

BACT_637 Hirschia baltica ATCC 49814

BACT_584 Gardnerella vaginalis ATCC 14019

BACT_575 Fusobacterium sp. D11

BACT_917 Providencia stuartii ATCC 25827

BACT_21 Acinetobacter baumannii ATCC 17978

BACT_1405 Haemophilus influenzae HK1212

BACT_1350 Enterococcus faecalis R712

BACT_1529 Vibrio alginolyticus 40B

BACT_1178 Thermoanaerobacter sp. X514

BACT_1626 Salinibacter ruber

BACT_1522 Streptomyces sp. ACTE

BACT_135 Bacillus cereus Rock3-44

BACT_275 Burkholderia sp. 383

BACT_44 Aeromonas salmonicida subsp. salmonicida A449

BACT_946 Psychromonas ingrahamii 37

BACT_1469 Pseudomonas syringae pv. tomato Max13

BACT_777 Mycobacterium kansasii ATCC 12478

BACT_687 Lactococcus lactis subsp. cremoris MG1363

BACT_335 Caulobacter crescentus NA1000

BACT_1374 Escherichia coli MS 182-1

BACT_1015 Roseovarius sp. TM1035

BACT_492 Dictyoglomus turgidum DSM 6724

BACT_1510 Staphylococcus aureus subsp. aureus WW2703/97

BACT_1585 Erwinia billingiae Eb661

BACT_409 Clostridium scindens ATCC 35704

BACT_1172 Thermanaerovibrio acidaminovorans DSM 6589

BACT_339 Chitinophaga pinensis DSM 2588

BACT_639 Holdemania filiformis DSM 12042

BACT_1038 Salmonella enterica subsp. enterica serovar Hadar str. RI_05P066

BACT_1257 Xanthobacter autotrophicus Py2

BACT_1056 Shewanella denitrificans OS217

BACT_18 Acidovorax sp. JS42

BACT_1575 Deferribacter desulfuricans SSM1

BACT_1003 Roseiflexus castenholzii DSM 13941

BACT_816 Nitrobacter sp. Nb-311A

BACT_411 Clostridium sp. L2-50

BACT_1472 Raphidiopsis brookii D9

BACT_354 Chlorobium phaeobacteroides DSM 266

BACT_1579 Desulfarculus baarsii DSM 2075

BACT_535 Eubacterium biforme DSM 3989

BACT_87 Aster yellows witches'-broom phytoplasma AYWB

BACT_1042 Salmonella enterica subsp. enterica serovar Saintpaul str. SARA29

BACT_495 Dorea formicigenerans ATCC 27755

BACT_82 Arthrobacter aurescens TC1

BACT_417 Clostridium thermocellum DSM 2360

BACT_141 Bacillus coagulans 36D1

BACT_778 Mycobacterium leprae TN

BACT_194 Bacteroides stercoris ATCC 43183

BACT_1368 Escherichia coli MS 115-1

BACT_842 Oribacterium sinus F0268

BACT_443 Crocosphaera watsonii WH 8501

BACT_1454 Prevotella bryantii B14

BACT_1267 Yersinia aldovae ATCC 35236

BACT_348 Chlamydophila pneumoniae CWL029

BACT_259 Bryantella formatexigens DSM 14469

BACT_793 Mycoplasma mycoides subsp. mycoides SC str. PG1

BACT_844 Orientia tsutsugamushi Boryong

BACT_1222 Vibrio cholerae B33

BACT_864 Pedobacter heparinus DSM 2366

BACT_1313 Bacteroides sp. 1_1_14

BACT_1016 Rothia mucilaginosa ATCC 25296

BACT_1120 Streptococcus thermophilus LMD-9

BACT_1515 Streptococcus pneumoniae str. Canada MDR_19A

BACT_1476 Roseomonas cervicalis ATCC 49957

BACT_1345 Desulfovibrio aespoeensis Aspo-2

BACT_1616 Pirellula staleyi DSM 6068

BACT_1275 Yersinia pestis biovar Mediaevalis str. K1973002

BACT_821 Nitrosomonas sp. AL212

BACT_911 Prosthecochloris aestuarii DSM 271

BACT_1325 Brucella sp. NVSL 07-0026

BACT_975 Rhodococcus jostii RHA1

BACT_529 Escherichia coli O157:H7 str. EC4024

BACT_1306 Arthrospira sp. PCC 8005

BACT_1069 Shigella flexneri 2a str. 301

BACT_375 Clostridium botulinum A2 str. Kyoto

BACT_168 Bacillus thuringiensis str. Al Hakam

BACT_1506 Staphylococcus aureus subsp. aureus MR1

BACT_527 Escherichia albertii TW07627

BACT_1152 Synechococcus sp. CC9902

BACT_788 Mycoplasma capricolum subsp. capricolum ATCC 27343

BACT_486 Dethiosulfovibrio peptidovorans DSM 11002

BACT_236 Borrelia hermsii DAH

BACT_1367 Escherichia coli MS 107-1

BACT_319 Candidatus Phytoplasma mali

BACT_1525 Turicibacter sp. PC909

BACT_1333 Chlamydia muridarum MopnTet14

BACT_142 Bacillus coahuilensis m4-4

BACT_31 Acinetobacter sp. ATCC 27244

BACT_646 Jannaschia sp. CCS1

BACT_438 Corynebacterium tuberculostearicum SK141

BACT_274 Burkholderia pseudomallei S13

BACT_1234 Vibrio mimicus VM573

BACT_977 Rhodoferax ferrireducens T118

BACT_783 Mycobacterium tuberculosis str. Haarlem

BACT_1012 Roseovarius nubinhibens ISM

BACT_1433 Mycobacterium tuberculosis 210

BACT_812 Neorickettsia sennetsu str. Miyayama

BACT_94 Azorhizobium caulinodans ORS 571

BACT_966 Rhizobium sp. NGR234

BACT_724 Magnetococcus sp. MC-1

BACT_914 Providencia alcalifaciens DSM 30120

BACT_759 Methylotenera mobilis JLW8

BACT_952 Ralstonia solanacearum GMI1000

BACT_326 Capnocytophaga ochracea DSM 7271

BACT_993 Rickettsia massiliae MTU5

BACT_1621 Ralstonia solanacearum PSI07

BACT_1559 Burkholderia sp. CCGE1002

BACT_703 Leuconostoc citreum KM20

BACT_1653 Zunongwangia profunda SM-A87

BACT_209 Beutenbergia cavernae DSM 12333

BACT_286 Campylobacter concisus 13826

BACT_1552 Bacillus thuringiensis BMB171

BACT_1517 Streptococcus sp. M143

BACT_744 Mesorhizobium sp. BNC1

BACT_279 Burkholderia ubonensis Bu

BACT_404 Clostridium perfringens D str. JGS1721

BACT_516 Enterococcus faecium DO

BACT_1228 Vibrio coralliilyticus ATCC BAA-450

BACT_950 Ralstonia eutropha JMP134

BACT_883 Plesiocystis pacifica SIR-1

BACT_919 Pseudoalteromonas tunicata D2

BACT_235 Borrelia garinii PBr

BACT_1030 Salinispora arenicola CNS-205

BACT_1263 Xanthomonas oryzae pv. oryzae PXO99A

BACT_134 Bacillus cereus Rock3-42

BACT_103 Bacillus cereus AH1271

BACT_1187 Thermosinus carboxydivorans Nor1

BACT_1076 Sinorhizobium meliloti 1021

BACT_1248 Wigglesworthia glossinidia endosymbiont of Glossina brevipalpis

BACT_1545 Arcanobacterium haemolyticum DSM 20595

BACT_1057 Shewanella frigidimarina NCIMB 400

BACT_1386 Ethanoligenens harbinense YUAN-3

BACT_60 Alteromonadales bacterium TW-7

BACT_399 Clostridium papyrosolvens DSM 2782

BACT_1634 Staphylococcus aureus subsp. aureus ED98

BACT_70 Anaerofustis stercorihominis DSM 17244

BACT_308 Candidatus Blochmannia pennsylvanicus str. BPEN

BACT_963 Rhizobium etli Kim 5

BACT_932 Pseudomonas stutzeri A1501

BACT_892 Prevotella bergensis DSM 17361

BACT_1534 Xanthomonas fuscans subsp. aurantifolii str. ICPB 10535

BACT_1140 Sulfitobacter sp. NAS-14.1

BACT_1455 Prevotella buccae D17

BACT_232 Borrelia duttonii Ly

BACT_1221 Vibrio cholerae 623-39

BACT_526 Erythrobacter sp. SD-21

BACT_64 Anaerocellum thermophilum DSM 6725

BACT_841 Opitutus terrae PB90-1

BACT_686 Lactobacillus vaginalis ATCC 49540

BACT_192 Bacteroides sp. D2

BACT_357 Chloroflexus aurantiacus J-10-fl

BACT_1286 Streptomyces sp. Mg1

BACT_119 Bacillus cereus BGSC 6E1

BACT_1321 Bacteroides xylanisolvens SD CC 1b

BACT_422 Comamonas testosteroni KF-1

BACT_988 Rickettsia bellii OSU 85-389

BACT_1280 Yersinia rohdei ATCC 43380

BACT_1121 Streptococcus uberis 0140J

BACT_1250 Wolbachia endosymbiont of Drosophila melanogaster

BACT_983 Rhodopseudomonas palustris TIE-1

BACT_827 Nostoc punctiforme PCC 73102

BACT_574 Fusobacterium sp. 7_1

BACT_1408 Ktedonobacter racemifer DSM 44963

BACT_941 Pseudovibrio sp. JE062

BACT_432 Corynebacterium glutamicum R

BACT_1026 Saccharomonospora viridis DSM 43017

BACT_1420 Lactobacillus salivarius ACS-116-V-Col5a

BACT_1151 Synechococcus sp. CC9605

BACT_1255 Wolbachia sp. wRi

BACT_971 Rhodobacterales bacterium HTCC2150

BACT_858 Pasteurella multocida subsp. multocida str. Pm70

BACT_577 Fusobacterium ulcerans ATCC 49185

BACT_440 Coxiella burnetii Dugway 5J108-111

BACT_171 Bacteroides caccae ATCC 43185

BACT_74 Anaerostipes caccae DSM 14662

BACT_53 Alistipes putredinis DSM 17216

BACT_1079 Sodalis glossinidius str. 'morsitans'

BACT_473 Desulfotomaculum reducens MI-1

BACT_701 Leptotrichia goodfellowii F0264

BACT_131 Bacillus cereus Rock1-15

BACT_39 Actinomyces sp. oral taxon 848 str. F0332

BACT_95 Azotobacter vinelandii DJ

BACT_879 Photorhabdus asymbiotica

BACT_835 Ochrobactrum intermedium LMG 3301

BACT_985 Rhodospirillum rubrum ATCC 11170

BACT_1095 Staphylococcus hominis SK119

BACT_271 Burkholderia mallei ATCC 10399

BACT_29 Acinetobacter radioresistens SK82

BACT_897 Prevotella tannerae ATCC 51259

BACT_493 Dinoroseobacter shibae DFL 12

BACT_451 Cyanothece sp. PCC 7425

BACT_1225 Vibrio cholerae MZO-3

BACT_1358 Enterococcus faecium E1679

BACT_1614 Olsenella uli DSM 7084

BACT_801 Nautilia profundicola AmH

BACT_314 Candidatus Hodgkinia cicadicola Dsem

BACT_1055 Shewanella benthica KT99

BACT_1094 Staphylococcus haemolyticus JCSC1435

BACT_846 Oxalobacter formigenes HOxBLS

BACT_397 Clostridium nexile DSM 1787

BACT_224 Blautia hansenii DSM 20583

BACT_774 Mycobacterium bovis BCG str. Pasteur 1173P2

BACT_586 Gemmata obscuriglobus UQM 2246

BACT_13 Acidithiobacillus ferrooxidans ATCC 23270

BACT_162 Bacillus thuringiensis serovar pakistani str. T13001

BACT_491 Dictyoglomus thermophilum H-6-12

BACT_295 Campylobacter lari RM2100

BACT_24 Acinetobacter baumannii SDF

BACT_635 Herminiimonas arsenicoxydans

BACT_1059 Shewanella loihica PV-4

BACT_775 Mycobacterium gilvum PYR-GCK

BACT_1227 Vibrio cholerae RC385

BACT_1403 Geobacillus thermoglucosidasius C56-YS93

BACT_817 Nitrobacter winogradskyi Nb-255

BACT_1439 Neisseria elongata subsp. glycolytica ATCC 29315

BACT_293 Campylobacter jejuni subsp. jejuni HB93-13

BACT_1606 Methylotenera sp. 301

BACT_715 Listeria monocytogenes HPB2262

BACT_6 Acaryochloris marina MBIC11017

BACT_556 Flavobacterium psychrophilum JIP02/86

BACT_292 Campylobacter jejuni subsp. jejuni BH-01-0142

BACT_1054 Shewanella baltica OS195

BACT_11 Acidiphilium cryptum JF-5

BACT_851 Parabacteroides johnsonii DSM 18315

BACT_1145 Sulfurospirillum deleyianum DSM 6946

BACT_1130 Streptomyces lividans TK24

BACT_351 Chlorobium ferrooxidans DSM 13031

BACT_1655 Brucella abortus bv. 5 str. B3196

BACT_543 Exiguobacterium sibiricum 255-15

BACT_707 Listeria monocytogenes FSL F2-515

BACT_519 Epulopiscium sp. 'N.t. morphotype B'

BACT_1092 Staphylococcus carnosus subsp. carnosus TM300

BACT_387 Clostridium carboxidivorans P7

BACT_735 Marinobacter algicola DG893

BACT_631 Helicobacter pylori B128

BACT_711 Listeria monocytogenes FSL J2-064

BACT_694 Leifsonia xyli subsp. xyli str. CTCB07

BACT_1189 Thermosipho melanesiensis BI429

BACT_257 Brucella sp. F5/99

BACT_970 Rhodobacterales bacterium HTCC2083

BACT_180 Bacteroides intestinalis DSM 17393

BACT_405 Clostridium perfringens E str. JGS1987

BACT_796 Mycoplasma pulmonis UAB CTIP

BACT_1381 Escherichia coli MS 45-1

BACT_282 Butyrivibrio crossotus DSM 2876

BACT_316 Candidatus Liberibacter asiaticus str. psy62

BACT_273 Burkholderia oklahomensis EO147

BACT_1163 Synechococcus sp. WH 8102

BACT_291 Campylobacter jejuni subsp. doylei 269.97

BACT_1562 Candidatus Riesia pediculicola USDA

BACT_113 Bacillus cereus ATCC 4342

BACT_614 Grimontia hollisae CIP 101886

BACT_33 Actinobacillus minor NM305

BACT_670 Lactobacillus gasseri JV-V03

BACT_403 Clostridium perfringens CPE str. F4969

BACT_431 Corynebacterium glucuronolyticum ATCC 51866

BACT_786 Mycoplasma agalactiae PG2

BACT_449 Cyanothece sp. CCY0110

BACT_954 Ralstonia solanacearum UW551

BACT_894 Prevotella melaninogenica ATCC 25845

BACT_1512 Staphylococcus epidermidis SK135

BACT_1440 Neisseria gonorrhoeae F62

BACT_371 Clostridium asparagiforme DSM 15981

BACT_1549 Bacillus megaterium DSM319

BACT_741 Mesoplasma florum L1

BACT_854 Parvibaculum lavamentivorans DS-1

BACT_592 Geobacillus sp. Y412MC10

BACT_1595 Lactobacillus crispatus ST1

BACT_872 Petrotoga mobilis SJ95

BACT_752 Methylobacterium populi BJ001

BACT_776 Mycobacterium intracellulare ATCC 13950

BACT_689 Laribacter hongkongensis HLHK9

BACT_1249 Wolbachia endosymbiont of Drosophila ananassae

BACT_1060 Shewanella oneidensis MR-1

BACT_1547 Azospirillum sp. B510

BACT_588 Geobacillus kaustophilus HTA426

BACT_1641 Syntrophothermus lipocalidus DSM 12680

BACT_372 Clostridium bartlettii DSM 16795

BACT_825 Nocardiopsis dassonvillei subsp. dassonvillei DSM 43111

BACT_165 Bacillus thuringiensis serovar sotto str. T04001

BACT_1604 Listeria seeligeri serovar 1/2b str. SLCC3954

BACT_753 Methylobacterium radiotolerans JCM 2831

BACT_754 Methylobacterium sp. 4-46

BACT_199 Bartonella henselae str. Houston-1

BACT_1357 Enterococcus faecium E1636

BACT_9 Acidaminococcus sp. D21

BACT_878 Photobacterium sp. SKA34

BACT_893 Prevotella copri DSM 18205

BACT_1077 Slackia exigua ATCC 700122

BACT_219 Bifidobacterium longum subsp. infantis ATCC 15697

BACT_272 Burkholderia multivorans ATCC 17616

BACT_1185 Thermomicrobium roseum DSM 5159

BACT_1458 Prevotella oris C735

BACT_969 Rhodobacteraceae bacterium KLH11

BACT_1470 Pseudomonas syringae pv. tomato NCPPB 1108

BACT_289 Campylobacter hominis ATCC BAA-381

BACT_368 Clavibacter michiganensis subsp. michiganensis NCPPB 382

BACT_861 Pectobacterium carotovorum subsp. carotovorum PC1

BACT_1141 Sulfurihydrogenibium azorense Az-Fu1

BACT_981 Rhodopseudomonas palustris BisB5

BACT_1216 Verrucomicrobiae bacterium DG1235

BACT_61 Ammonifex degensii KC4

BACT_920 Pseudomonas aeruginosa 2192

BACT_781 Mycobacterium sp. JLS

BACT_1220 Vibrio cholerae 1587

BACT_876 Photobacterium profundum 3TCK

BACT_714 Listeria monocytogenes HCC23

BACT_498 Edwardsiella ictaluri 93-146

BACT_1402 Gardnerella vaginalis AMD

BACT_1588 Geobacillus sp. C56-T3

BACT_1207 Tropheryma whipplei str. Twist

BACT_433 Corynebacterium kroppenstedtii DSM 44385

BACT_839 Onion yellows phytoplasma OY-M

BACT_1426 Megasphaera genomosp. type_1 str. 28L

BACT_276 Burkholderia sp. H160

BACT_555 Flavobacterium johnsoniae UW101

BACT_477 Desulfovibrio piger ATCC 29098

BACT_674 Lactobacillus jensenii 1153

BACT_643 Idiomarina baltica OS145

BACT_426 Coprothermobacter proteolyticus DSM 5265

BACT_831 Oceanicaulis alexandrii HTCC2633

BACT_1236 Vibrio shilonii AK1

BACT_126 Bacillus cereus m1550

BACT_1481 Serratia odorifera 4Rx13

BACT_726 Magnetospirillum magnetotacticum MS-1

BACT_297 Campylobacter showae RM3277

BACT_1037 Salmonella enterica subsp. enterica serovar Gallinarum str. 287/91

BACT_1197 Thermus aquaticus Y51MC23

BACT_885 Polaribacter sp. MED152

BACT_1340 Comamonas testosteroni S44

BACT_1150 Synechococcus sp. CC9311

BACT_1073 Sideroxydans lithotrophicus ES-1

BACT_1190 Thermosynechococcus elongatus BP-1

BACT_1282 Zymomonas mobilis subsp. mobilis ATCC 10988

BACT_201 Bartonella tribocorum CIP 105476

BACT_396 Clostridium methylpentosum DSM 5476

BACT_570 Fusobacterium sp. 3_1_33

BACT_992 Rickettsia felis URRWXCal2

BACT_710 Listeria monocytogenes FSL J1-208

BACT_538 Eubacterium hallii DSM 3353

BACT_655 Kordia algicida OT-1

BACT_961 Rhizobium etli GR56

BACT_942 Psychrobacter arcticus 273-4

BACT_1548 Bacillus anthracis CI

BACT_1289 Achromobacter piechaudii ATCC 43553

BACT_856 Parvularcula bermudensis HTCC2503

BACT_1503 Staphylococcus aureus subsp. aureus M809

BACT_1573 Cupriavidus metallidurans CH34

BACT_401 Clostridium perfringens B str. ATCC 3626

BACT_66 Anaerococcus lactolyticus ATCC 51172

BACT_1237 Vibrio sp. AND4

BACT_934 Pseudomonas syringae pv. oryzae str. 1_6

BACT_496 Dorea longicatena DSM 13814

BACT_1171 Thauera sp. MZ1T

BACT_200 Bartonella quintana str. Toulouse

BACT_398 Clostridium novyi NT

BACT_1365 Escherichia coli FVEC1302

BACT_904 Prochlorococcus marinus str. MIT 9312

BACT_26 Acinetobacter johnsonii SH046

BACT_889 Porphyromonas endodontalis ATCC 35406

BACT_1053 Shewanella amazonensis SB2B

BACT_1109 Streptococcus equi subsp. equi 4047

BACT_1473 Rhodococcus equi ATCC 33707

BACT_797 Mycoplasma synoviae 53

BACT_1567 Clostridiales genomosp. BVAB3 str. UPII9-5

BACT_1139 Subdoligranulum variabile DSM 15176

BACT_773 Mycobacterium avium

BACT_1193 Thermotoga naphthophila RKU-10

BACT_152 Bacillus subtilis subsp. subtilis str. NCIB 3610

BACT_1198 Thermus thermophilus HB27

BACT_332 Catenibacterium mitsuokai DSM 15897

BACT_1097 Staphylococcus warneri L37603

BACT_117 Bacillus cereus BDRD-ST24

BACT_1138 Streptosporangium roseum DSM 43021

BACT_1298 Acinetobacter sp. 6013150

BACT_393 Clostridium hylemonae DSM 15053

BACT_924 Pseudomonas fluorescens Pf0-1

BACT_537 Eubacterium eligens ATCC 27750

BACT_1526 Veillonella parvula ATCC 17745

BACT_212 Bifidobacterium bifidum NCIMB 41171

BACT_1006 Roseobacter litoralis Och 149

BACT_246 Brevibacillus brevis NBRC 100599

BACT_994 Rickettsia peacockii str. Rustic

BACT_962 Rhizobium etli IE4771

BACT_1051 Selenomonas sputigena ATCC 35185

BACT_828 Nostoc sp. PCC 7120

BACT_1132 Streptomyces sp. AA4

BACT_1437 Mycobacterium tuberculosis KZN V2475

BACT_1107 Streptococcus agalactiae NEM316

BACT_1375 Escherichia coli MS 185-1

BACT_976 Rhodococcus opacus B4

BACT_1133 Streptomyces sp. C

BACT_441 Coxiella burnetii RSA 334

BACT_980 Rhodopseudomonas palustris BisB18

BACT_673 Lactobacillus iners DSM 13335

BACT_1452 Photobacterium damselae subsp. damselae CIP 102761

BACT_594 Geobacillus sp. Y412MC61

BACT_1211 Ureaplasma parvum serovar 6 str. ATCC 27818

BACT_1601 Leuconostoc kimchii IMSNU 11154

BACT_1497 Staphylococcus aureus subsp. aureus C160

BACT_485 Dethiobacter alkaliphilus AHT 1

BACT_837 Octadecabacter antarcticus 307

BACT_1274 Yersinia pestis biovar Antiqua str. E1979001

BACT_419 Collinsella intestinalis DSM 13280

BACT_108 Bacillus cereus AH676

BACT_654 Kocuria rhizophila DC2201

BACT_394 Clostridium kluyveri DSM 555

BACT_1561 Candidatus Puniceispirillum marinum IMCC1322

BACT_554 Flavobacteriales bacterium HTCC2170

BACT_633 Helicobacter winghamensis ATCC BAA-430

BACT_599 Geobacter sp. FRC-32

BACT_1342 Corynebacterium resistens DSM 45100

BACT_887 Polaromonas sp. JS666

BACT_1018 Ruegeria pomeroyi DSS-3

BACT_1269 Yersinia enterocolitica subsp. enterocolitica 8081

BACT_124 Bacillus cereus H3081.97

BACT_1461 Prevotella timonensis CRIS 5C-B1

BACT_1581 Edwardsiella tarda EIB202

BACT_721 Lyngbya sp. PCC 8106

BACT_871 Persephonella marina EX-H1

BACT_690 Lawsonia intracellularis PHE/MN1-00

BACT_873 Phaeobacter gallaeciensis BS107

BACT_154 Bacillus thuringiensis IBL 200

BACT_163 Bacillus thuringiensis serovar pondicheriensis BGSC 4BA1

BACT_442 Croceibacter atlanticus HTCC2559

BACT_1625 Ruegeria sp. TM1040

BACT_1173 Thermoanaerobacter brockii subsp. finnii Ako-1

BACT_1304 Aggregatibacter actinomycetemcomitans D7S-1

BACT_1592 Hydrogenobacter thermophilus TK-6

BACT_1646 Thioalkalivibrio sp. K90mix

BACT_1370 Escherichia coli MS 119-7

BACT_388 Clostridium cellulolyticum H10

BACT_380 Clostridium botulinum C str. Eklund

BACT_71 Anaeromyxobacter dehalogenans 2CP-1

BACT_5 Abiotrophia defectiva ATCC 49176

BACT_1080 Solibacter usitatus Ellin6076

BACT_784 Mycobacterium ulcerans Agy99

BACT_677 Lactobacillus jensenii SJ-7A-US

BACT_1349 Enterococcus faecalis PC1.1

BACT_1378 Escherichia coli MS 198-1

BACT_923 Pseudomonas fluorescens Pf-5

BACT_1387 Filifactor alocis ATCC 35896

BACT_324 Candidatus Vesicomyosocius okutanii HA

BACT_1385 Escherichia coli OP50

BACT_425 Coprococcus eutactus ATCC 27759

BACT_758 Methylophilales bacterium HTCC2181

BACT_1075 Sinorhizobium medicae WSM419

BACT_381 Clostridium botulinum D str. 1873

BACT_1474 Rhodomicrobium vannielii ATCC 17100

BACT_1270 Yersinia frederiksenii ATCC 33641

BACT_1058 Shewanella halifaxensis HAW-EB4

BACT_1296 Acinetobacter haemolyticus ATCC 19194

BACT_648 Jonesia denitrificans DSM 20603

BACT_185 Bacteroides sp. 2_1_16

BACT_111 Bacillus cereus ATCC 10987

BACT_1273 Yersinia mollaretii ATCC 43969

BACT_391 Clostridium hathewayi DSM 13479

BACT_838 Oligotropha carboxidovorans OM5

BACT_1644 Thermocrinis albus DSM 14484

BACT_1563 Candidatus Sulcia muelleri DMIN

BACT_258 Brucella suis bv. 4 str. 40

BACT_1019 Ruegeria sp. R11

BACT_266 Burkholderia ambifaria MEX-5

BACT_1049 Selenomonas flueggei ATCC 43531

BACT_930 Pseudomonas putida W619

BACT_352 Chlorobium limicola DSM 245

BACT_109 Bacillus cereus AH820

BACT_145 Bacillus mycoides DSM 2048

BACT_337 Cellulomonas flavigena DSM 20109

BACT_1657 Saccharopolyspora erythraea NRRL 2338

BACT_453 Cyanothece sp. PCC 8801

BACT_761 Micrococcus luteus NCTC 2665

BACT_430 Corynebacterium genitalium ATCC 33030

BACT_462 Delftia acidovorans SPH-1

BACT_1101 Streptobacillus moniliformis DSM 12112

BACT_1377 Escherichia coli MS 196-1

BACT_1613 Nitrosococcus watsoni C-113

BACT_808 Neisseria mucosa ATCC 25996

BACT_471 Desulfotalea psychrophila LSv54

BACT_377 Clostridium botulinum B1 str. Okra

BACT_27 Acinetobacter junii SH205

BACT_106 Bacillus cereus AH603

BACT_1635 Staphylococcus lugdunensis HKU09-01

BACT_869 Pelodictyon phaeoclathratiforme BU-1

BACT_918 Pseudoalteromonas atlantica T6c

BACT_1129 Streptomyces hygroscopicus ATCC 53653

BACT_1011 Roseobacter sp. SK209-2-6

BACT_1293 Acinetobacter baumannii AB056

BACT_1360 Enterococcus faecium PC4.1

BACT_525 Erythrobacter sp. NAP1

BACT_1383 Escherichia coli MS 78-1

BACT_1482 Serratia odorifera DSM 4582

BACT_358 Chloroflexus sp. Y-400-fl

BACT_367 Citrobacter youngae ATCC 29220

BACT_742 Mesorhizobium loti MAFF303099

BACT_383 Clostridium botulinum E3 str. Alaska E43

BACT_12 Acidithiobacillus caldus ATCC 51756

BACT_414 Clostridium spiroforme DSM 1552

BACT_1484 Simonsiella muelleri ATCC 29453

BACT_1224 Vibrio cholerae MJ-1236

BACT_196 Bacteroides vulgatus ATCC 8482

BACT_886 Polaromonas naphthalenivorans CJ2

BACT_561 Frankia sp. EAN1pec

BACT_307 Candidatus Blochmannia floridanus

BACT_459 Dehalococcoides sp. VS

BACT_30 Acinetobacter sp. ADP1

BACT_549 Flavobacteria bacterium BAL38

BACT_1311 Bacteroides ovatus SD CC 2a

BACT_1346 Desulfovibrio sp. FW1012B

BACT_523 Erysipelothrix rhusiopathiae ATCC 19414

BACT_122 Bacillus cereus G9241

BACT_226 Bordetella avium 197N

BACT_862 Pectobacterium wasabiae WPP163

BACT_55 Alkaliphilus metalliredigens QYMF

BACT_1014 Roseovarius sp. HTCC2601

BACT_1479 Ruminococcus albus 8

BACT_187 Bacteroides sp. 2_1_33B

BACT_596 Geobacter bemidjiensis Bem

BACT_284 Caminibacter mediatlanticus TB-2

BACT_1650 Xenorhabdus bovienii SS-2004

BACT_1279 Yersinia pseudotuberculosis IP 32953

BACT_1596 Lactobacillus johnsonii FI9785

BACT_890 Porphyromonas gingivalis ATCC 33277

BACT_1276 Yersinia pestis biovar Microtus str. 91001

BACT_446 Cyanobium sp. PCC 7001

BACT_93 Azoarcus sp. BH72

BACT_559 Frankia alni ACN14a

BACT_118 Bacillus cereus BDRD-ST26

BACT_1244 Vibrio vulnificus YJ016

BACT_1401 Gardnerella vaginalis 5-1

BACT_1074 Silicibacter lacuscaerulensis ITI-1157

BACT_1017 Rubrobacter xylanophilus DSM 9941

BACT_1586 Escherichia coli O55:H7 str. CB9615

BACT_1031 Salinispora tropica CNB-440

BACT_158 Bacillus thuringiensis serovar huazhongensis BGSC 4BD1

BACT_49 Akkermansia muciniphila ATCC BAA-835

BACT_1521 Streptomyces sp. ACT-1

BACT_1645 Thermosediminibacter oceani DSM 16646

BACT_501 Ehrlichia chaffeensis str. Arkansas

BACT_243 Bradyrhizobium japonicum USDA 110

BACT_211 Bifidobacterium animalis subsp. lactis Bl-04

BACT_1609 Mycoplasma agalactiae

BACT_1240 Vibrio sp. RC341

BACT_107 Bacillus cereus AH621

BACT_1555 Bifidobacterium longum subsp. longum JDM301

BACT_1488 Staphylococcus aureus A8796

BACT_1262 Xanthomonas campestris pv. vesicatoria str. 85-10

BACT_1379 Escherichia coli MS 200-1

BACT_233 Borrelia garinii Far04

BACT_1125 Streptomyces flavogriseus ATCC 33331

BACT_1487 Staphylococcus aureus A8117

BACT_695 Lentisphaera araneosa HTCC2155

BACT_1254 Wolbachia endosymbiont strain TRS of Brugia malayi

BACT_1322 Bacteroidetes oral taxon 274 str. F0058

BACT_183 Bacteroides plebeius DSM 17135

BACT_51 Alcanivorax sp. DG881

BACT_1643 Thermobispora bispora DSM 43833

BACT_96 Bacillus amyloliquefaciens FZB42

BACT_929 Pseudomonas putida KT2440

BACT_386 Clostridium butyricum E4 str. BoNT E BL5262

BACT_651 Kineococcus radiotolerans SRS30216

BACT_1251 Wolbachia endosymbiont of Drosophila simulans

BACT_1148 Synechococcus elongatus PCC 6301

BACT_65 Anaerococcus hydrogenalis DSM 7454

BACT_290 Campylobacter jejuni RM1221

BACT_421 Colwellia psychrerythraea 34H

BACT_865 Pedobacter sp. BAL39

BACT_1369 Escherichia coli MS 116-1

BACT_840 Opitutaceae bacterium TAV2

BACT_1612 Nitrosococcus halophilus Nc4

BACT_955 Reinekea blandensis MED297

BACT_1462 Propionibacterium acnes J139

BACT_1569 Clostridium saccharolyticum WM1

BACT_737 Marinobacter sp. ELB17

BACT_546 Faecalibacterium prausnitzii M21/2

BACT_1659 Bacillus subtilis subsp. spizizenii ATCC 6633

BACT_1085 Sphingomonas wittichii RW1

BACT_408 Clostridium ramosum DSM 1402

BACT_1356 Enterococcus faecium E1162

BACT_464 Denitrovibrio acetiphilus DSM 12809

BACT_1590 Helicobacter pylori B8

BACT_1232 Vibrio harveyi HY01

BACT_1355 Enterococcus faecium E1071

BACT_647 Janthinobacterium sp. Marseille

BACT_636 Herpetosiphon aurantiacus ATCC 23779

BACT_1238 Vibrio sp. Ex25

BACT_712 Listeria monocytogenes FSL J2-071

BACT_1239 Vibrio sp. MED222

BACT_1201 Thiomonas intermedia K12

BACT_1637 Streptococcus gallolyticus UCN34

BACT_1453 Prevotella bivia JCVIHMP010

BACT_1090 Staphylococcus aureus subsp. aureus USA300_TCH1516

BACT_1400 Fusobacterium sp. 3_1_27

BACT_1167 Syntrophomonas wolfei subsp. wolfei str. Goettingen

BACT_67 Anaerococcus prevotii DSM 20548

BACT_755 Methylocella silvestris BL2

BACT_1468 Pseudomonas syringae pv. tomato K40

BACT_974 Rhodobacterales bacterium Y4I

BACT_591 Geobacillus sp. Y4.1MC1

BACT_1066 Shewanella sp. W3-18-1

BACT_428 Corynebacterium amycolatum SK46

BACT_1223 Vibrio cholerae CT 5369-93

BACT_1538 Yersinia pestis KIM D27

BACT_1041 Salmonella enterica subsp. enterica serovar Paratyphi B str. SPB7

BACT_1499 Staphylococcus aureus subsp. aureus D139

BACT_987 Rickettsia akari str. Hartford

BACT_740 Meiothermus silvanus DSM 9946

BACT_120 Bacillus cereus E33L

BACT_965 Rhizobium leguminosarum bv. viciae 3841

BACT_680 Lactobacillus reuteri 100-23

BACT_650 Kangiella koreensis DSM 16069

BACT_907 Prochlorococcus marinus str. NATL1A

BACT_667 Lactobacillus fermentum 28-3-CHN

BACT_569 Fusobacterium sp. 2_1_31

BACT_54 Alkalilimnicola ehrlichii MLHE-1

BACT_25 Acinetobacter calcoaceticus RUH2202

BACT_129 Bacillus cereus Q1

BACT_1230 Vibrio furnissii CIP 102972

BACT_1504 Staphylococcus aureus subsp. aureus M899

BACT_1630 Sphingobium japonicum UT26S

BACT_81 Aromatoleum aromaticum EbN1

BACT_832 Oceanicola batsensis HTCC2597

BACT_350 Chlorobium chlorochromatii CaD3

BACT_1551 Bacillus pseudofirmus OF4

BACT_847 Oxalobacter formigenes OXCC13

BACT_514 Enterococcus faecalis TX0104

BACT_623 Halothiobacillus neapolitanus c2

BACT_57 Allochromatium vinosum DSM 180

BACT_1464 Propionibacterium acnes SK187

BACT_1156 Synechococcus sp. PCC 7335

BACT_903 Prochlorococcus marinus str. MIT 9303

BACT_392 Clostridium hiranonis DSM 13275

BACT_999 Rickettsiella grylli

BACT_159 Bacillus thuringiensis serovar israelensis ATCC 35646

BACT_8 Acholeplasma laidlawii PG-8A

BACT_875 Photobacterium angustum S14

BACT_1215 Verminephrobacter eiseniae EF01-2

BACT_610 Gramella forsetii KT0803

BACT_1297 Acinetobacter sp. 6013113

BACT_150 Bacillus sp. NRRL B-14911

BACT_210 Bifidobacterium angulatum DSM 20098

BACT_1406 Helicobacter pylori 35A

BACT_791 Mycoplasma hyopneumoniae 7448

BACT_390 Clostridium difficile QCD-63q42

BACT_97 Bacillus anthracis Tsiankovskii-I

BACT_287 Campylobacter curvus 525.92

BACT_562 Fulvimarina pelagi HTCC2506

BACT_127 Bacillus cereus MM3

BACT_338 Cellvibrio japonicus Ueda107

BACT_978 Rhodopirellula baltica SH 1

BACT_1136 Streptomyces sp. SPB78

BACT_205 Beggiatoa sp. SS

BACT_329 Carboxydothermus hydrogenoformans Z-2901

BACT_1131 Streptomyces roseosporus NRRL 11379

BACT_996 Rickettsia rickettsii str. Iowa

BACT_7 Acetobacter pasteurianus IFO 3283-01

BACT_915 Providencia rettgeri DSM 1131

BACT_227 Bordetella bronchiseptica RB50

BACT_771 Moritella sp. PE36

BACT_1196 Thermotoga sp. RQ2

BACT_1005 Roseobacter denitrificans OCh 114

BACT_189 Bacteroides sp. 3_1_33FAA

BACT_435 Corynebacterium matruchotii ATCC 33806

BACT_691 Leeuwenhoekiella blandensis MED217

BACT_1119 Streptococcus suis BM407

BACT_587 Gemmatimonas aurantiaca T-27

BACT_748 Methylobacterium chloromethanicum CM4

BACT_722 Lysinibacillus sphaericus C3-41

BACT_718 Listeria welshimeri serovar 6b str. SLCC5334

BACT_531 Escherichia coli str. K-12 substr. MG1655

BACT_1147 Symbiobacterium thermophilum IAM 14863

BACT_1597 Lactococcus lactis subsp. lactis KF147

BACT_475 Desulfovibrio desulfuricans subsp. desulfuricans str. ATCC 27774

BACT_1570 Conexibacter woesei DSM 14684

BACT_609 Gluconobacter oxydans 621H

BACT_852 Parabacteroides merdae ATCC 43184

BACT_705 Listeria grayi DSM 20601

BACT_683 Lactobacillus ruminis ATCC 25644

BACT_563 Fusobacterium gonidiaformans ATCC 25563

BACT_882 Planctomyces maris DSM 8797

BACT_863 Pediococcus pentosaceus ATCC 25745

BACT_1492 Staphylococcus aureus subsp. aureus 58-424

BACT_1611 Mycoplasma hominis

BACT_708 Listeria monocytogenes FSL J1-175

BACT_830 Oceanibulbus indolifex HEL-45

BACT_166 Bacillus thuringiensis serovar thuringiensis str. T01001

BACT_84 Arthrobacter sp. FB24

BACT_611 Granulibacter bethesdensis CGDNIH1

BACT_866 Pelobacter carbinolicus DSM 2380

BACT_595 Geobacillus thermodenitrificans NG80-2

BACT_248 Brevundimonas sp. BAL3

BACT_792 Mycoplasma mobile 163K

BACT_622 Halothermothrix orenii H 168

BACT_518 Enterococcus gallinarum EG2

BACT_385 Clostridium botulinum NCTC 2916

BACT_571 Fusobacterium sp. 3_1_36A2

BACT_285 Campylobacter coli RM2228

BACT_63 Anaerobaculum hydrogeniformans ATCC BAA-1850

BACT_1483 Shewanella baltica OS678

BACT_1061 Shewanella pealeana ATCC 700345

BACT_884 Polaribacter irgensii 23-P

BACT_573 Fusobacterium sp. 4_1_13

BACT_1421 Legionella longbeachae D-4968

BACT_1631 Spirochaeta smaragdinae DSM 11293

BACT_1050 Selenomonas noxia ATCC 43541

BACT_281 Burkholderia xenovorans LB400

BACT_751 Methylobacterium nodulans ORS 2060

BACT_1527 Veillonella sp. 3_1_44

BACT_1576 Dehalococcoides sp. GT

BACT_1638 Streptococcus mitis B6

BACT_466 Desulfitobacterium hafniense Y51

BACT_115 Bacillus cereus BDRD-Cer4

BACT_585 Gemella haemolysans ATCC 10379

BACT_1179 Thermoanaerobacter sp. X561

BACT_607 Gluconacetobacter diazotrophicus PAl 5

BACT_805 Neisseria gonorrhoeae NCCP11945

BACT_1649 Xanthomonas albilineans

BACT_979 Rhodopseudomonas palustris BisA53

BACT_926 Pseudomonas mendocina ymp

BACT_925 Pseudomonas fluorescens SBW25

BACT_544 Exiguobacterium sp. AT1b

BACT_806 Neisseria lactamica ATCC 23970

BACT_1305 Alicycliphilus denitrificans BC

BACT_1241 Vibrio sp. RC586

BACT_389 Clostridium cellulovorans 743B

BACT_502 Ehrlichia chaffeensis str. Sapulpa

BACT_100 Bacillus cereus 172560W

BACT_766 Mitsuokella multacida DSM 20544

BACT_1434 Mycobacterium tuberculosis KZN 4207

BACT_439 Corynebacterium urealyticum DSM 7109

BACT_1205 Treponema vincentii ATCC 35580

BACT_1624 Rothia mucilaginosa DY-18

BACT_1065 Shewanella sp. MR-7

BACT_181 Bacteroides ovatus ATCC 8483

BACT_1388 Finegoldia magna ACS-171-V-Col3

BACT_627 Helicobacter cinaedi CCUG 18818

BACT_1532 Vibrio harveyi 1DA3

BACT_912 Prosthecochloris vibrioformis DSM 265

BACT_169 Bacillus weihenstephanensis KBAB4

BACT_604 Geodermatophilus obscurus DSM 43160

BACT_1380 Escherichia coli MS 21-1

BACT_1459 Prevotella sp. oral taxon 299 str. F0039

BACT_1086 Sphingopyxis alaskensis RB2256

BACT_530 Escherichia coli SE11

BACT_1557 Brachyspira pilosicoli 95/1000

BACT_568 Fusobacterium periodonticum ATCC 33693

BACT_1127 Streptomyces griseoflavus Tu4000

BACT_1589 Helicobacter mustelae 12198

BACT_968 Rhodobacter sphaeroides 2.4.1

BACT_363 Chthoniobacter flavus Ellin428

BACT_91 Atopobium vaginae DSM 15829

BACT_763 Microcystis aeruginosa NIES-843

BACT_678 Lactobacillus paracasei subsp. paracasei 8700:2

BACT_1261 Xanthomonas campestris pv. vasculorum NCPPB702

BACT_1540 Acidaminococcus fermentans DSM 20731

BACT_182 Bacteroides pectinophilus ATCC 43243

BACT_613 Granulicatella elegans ATCC 700633

BACT_602 Geobacter sulfurreducens PCA

BACT_641 Hyphomicrobium denitrificans ATCC 51888

BACT_320 Candidatus Protochlamydia amoebophila UWE25

BACT_156 Bacillus thuringiensis serovar andalousiensis BGSC 4AW1

BACT_1566 Citrobacter rodentium ICC168

BACT_874 Phenylobacterium zucineum HLK1

BACT_933 Pseudomonas syringae pv. aesculi str. 2250

BACT_696 Leptolyngbya valderiana BDU 20041

BACT_658 Kytococcus sedentarius DSM 20547

BACT_424 Coprococcus comes ATCC 27758

BACT_1122 Streptomyces albus J1074

BACT_20 Acinetobacter baumannii AB307-0294

BACT_415 Clostridium sporogenes ATCC 15579

BACT_632 Helicobacter pylori HPKX_438_AG0C1

BACT_489 Dichelobacter nodosus VCS1703A

BACT_1328 Burkholderia sp. CCGE1003

BACT_899 Prochlorococcus marinus str. AS9601

BACT_80 Arcobacter butzleri RM4018

BACT_1361 Enterococcus faecium U0317

BACT_1303 Afipia sp. 1NLS2

BACT_32 Acinetobacter sp. RUH2624

BACT_384 Clostridium botulinum F str. Langeland

BACT_663 Lactobacillus casei BL23

BACT_90 Atopobium rimae ATCC 49626

BACT_1315 Bacteroides sp. 3_1_19

BACT_123 Bacillus cereus G9842

BACT_37 Actinomyces coleocanis DSM 15436

BACT_1471 Pyramidobacter piscolens W5455

BACT_296 Campylobacter rectus RM3267

BACT_186 Bacteroides sp. 2_1_22

BACT_1456 Prevotella buccalis ATCC 35310

BACT_626 Helicobacter canadensis MIT 98-5491

BACT_179 Bacteroides fragilis YCH46

BACT_330 Cardiobacterium hominis ATCC 15826

BACT_939 Pseudomonas syringae pv. tomato str. DC3000

BACT_1412 Lactobacillus delbrueckii subsp. bulgaricus PB2003/044-T3-4

BACT_1647 Truepera radiovictrix DSM 17093

BACT_454 Cyanothece sp. PCC 8802

BACT_1477 Rothia dentocariosa ATCC 17931

BACT_336 Caulobacter sp. K31

BACT_456 Dechloromonas aromatica RCB

BACT_671 Lactobacillus gasseri MV-22

BACT_553 Flavobacteriaceae bacterium 3519-10

BACT_41 Actinosynnema mirum DSM 43827

BACT_228 Bordetella parapertussis 12822

BACT_4 Flavobacteriales bacterium ALC-1

BACT_1212 Ureaplasma urealyticum serovar 9 str. ATCC 33175

BACT_1475 Rhodopseudomonas palustris DX-1

BACT_1165 Synechocystis sp. PCC 6803

BACT_1450 Peptoniphilus sp. oral taxon 836 str. F0141

BACT_221 Bifidobacterium pseudocatenulatum DSM 20438

BACT_353 Chlorobium phaeobacteroides BS1

BACT_606 Gloeobacter violaceus PCC 7421

BACT_190 Bacteroides sp. 3_2_5

BACT_101 Bacillus cereus 95/8201

BACT_931 Pseudomonas sp. UK4

BACT_509 Cronobacter sakazakii ATCC BAA-894

BACT_779 Mycobacterium marinum M

BACT_601 Geobacter sp. M21

BACT_1339 Clostridium thermocellum JW20

BACT_138 Bacillus cereus subsp. cytotoxis NVH 391-98

BACT_1603 Listeria monocytogenes 08-5923

BACT_522 Erwinia tasmaniensis Et1/99

BACT_855 Parvimonas micra ATCC 33270

BACT_474 Desulfovibrio desulfuricans G20

BACT_1048 Sebaldella termitidis ATCC 33386

BACT_1104 Streptococcus agalactiae CJB111

BACT_1180 Thermoanaerobacter tengcongensis MB4

BACT_1354 Enterococcus faecium E1039

BACT_809 Neisseria sicca ATCC 29256

BACT_1112 Streptococcus infantarius subsp. infantarius ATCC BAA-102

BACT_416 Clostridium tetani E88

BACT_567 Fusobacterium nucleatum subsp. vincentii ATCC 49256

BACT_1514 Streptococcus parasanguinis ATCC 15912

BACT_515 Enterococcus faecalis V583

BACT_540 Eubacterium saphenum ATCC 49989

BACT_706 Listeria innocua Clip11262

BACT_1028 Sagittula stellata E-37

BACT_603 Geobacter uraniireducens Rf4

BACT_937 Pseudomonas syringae pv. syringae FF5

BACT_222 Blastopirellula marina DSM 3645

BACT_935 Pseudomonas syringae pv. phaseolicola 1448A

BACT_709 Listeria monocytogenes FSL J1-194

BACT_497 Dyadobacter fermentans DSM 18053

BACT_1478 Rothia dentocariosa M567

BACT_1027 Saccharophagus degradans 2-40

BACT_1554 Bifidobacterium dentium Bd1

BACT_990 Rickettsia conorii str. Malish 7

BACT_906 Prochlorococcus marinus str. MIT 9515

BACT_896 Prevotella sp. oral taxon 472 str. F0295

BACT_1001 Roseburia intestinalis L1-82

BACT_1600 Leuconostoc gasicomitatum LMG 18811

BACT_361 Chromohalobacter salexigens DSM 3043

BACT_1580 Dickeya dadantii Ech586

BACT_1636 Starkeya novella DSM 506

BACT_1067 Shewanella woodyi ATCC 51908

BACT_576 Fusobacterium sp. D12

BACT_521 Erwinia pyrifoliae Ep1/96

BACT_1502 Staphylococcus aureus subsp. aureus M1015

BACT_880 Photorhabdus luminescens subsp. laumondii TTO1

BACT_79 Aquifex aeolicus VF5

BACT_829 Novosphingobium aromaticivorans DSM 12444

BACT_1063 Shewanella sediminis HAW-EB3

BACT_277 Burkholderia thailandensis Bt4

BACT_550 Flavobacteria bacterium BBFL7

BACT_1246 Victivallis vadensis ATCC BAA-548

BACT_750 Methylobacterium extorquens DM4

BACT_621 Halorhodospira halophila SL1

BACT_1395 Frankia sp. EUN1f

BACT_161 Bacillus thuringiensis serovar monterrey BGSC 4AJ1

BACT_1099 Stenotrophomonas sp. SKA14

BACT_133 Bacillus cereus Rock3-28

BACT_325 Capnocytophaga gingivalis ATCC 33624

BACT_782 Mycobacterium tuberculosis 02_1987

BACT_644 Idiomarina loihiensis L2TR

BACT_1341 Corynebacterium ammoniagenes DSM 20306

BACT_240 Brachybacterium faecium DSM 4810

BACT_229 Bordetella pertussis Tohama I

BACT_1654 Erysipelotrichaceae bacterium 5_2_54FAA

BACT_45 Aggregatibacter actinomycetemcomitans D11S-1

BACT_666 Lactobacillus delbrueckii subsp. bulgaricus ATCC 11842

BACT_672 Lactobacillus hilgardii ATCC 8290

BACT_668 Lactobacillus fermentum IFO 3956

BACT_1256 Wolinella succinogenes DSM 1740

BACT_22 Acinetobacter baumannii ATCC 19606

BACT_450 Cyanothece sp. PCC 7424

BACT_174 Bacteroides coprocola DSM 17136

BACT_356 Chloroflexus aggregans DSM 9485

BACT_959 Rhizobium etli CIAT 652

BACT_1528 Veillonella sp. 6_1_27

BACT_1009 Roseobacter sp. GAI101

BACT_75 Anaerotruncus colihominis DSM 17241

BACT_128 Bacillus cereus NVH0597-99

BACT_716 Listeria monocytogenes J0161

BACT_1096 Staphylococcus saprophyticus subsp. saprophyticus ATCC 15305

BACT_429 Corynebacterium diphtheriae NCTC 13129

BACT_478 Desulfovibrio salexigens DSM 2638

BACT_617 Haemophilus influenzae 6P18H1

BACT_1490 Staphylococcus aureus A9754

BACT_1574 Cyanobacterium UCYN-A

BACT_484 Desulfuromonas acetoxidans DSM 684

BACT_836 Octadecabacter antarcticus 238

BACT_1200 Thiomicrospira crunogena XCL-2

BACT_785 Mycobacterium vanbaalenii PYR-1

BACT_536 Eubacterium dolichum DSM 3991

BACT_35 Actinobacillus pleuropneumoniae serovar 7 str. AP76

BACT_629 Helicobacter pullorum MIT 98-5489

BACT_1213 Veillonella dispar ATCC 17748

BACT_197 Bartonella bacilliformis KC583

BACT_1376 Escherichia coli MS 187-1

BACT_149 Bacillus sp. B14905

BACT_1397 Fusobacterium nucleatum subsp. nucleatum ATCC 23726

BACT_1308 Bacillus cellulosilyticus DSM 2522

BACT_125 Bacillus cereus m1293

BACT_1539 Acetohalobium arabaticum DSM 5501

BACT_860 Pectobacterium carotovorum subsp. brasiliensis PBR1692

BACT_1192 Thermotoga maritima MSB8

BACT_188 Bacteroides sp. 2_1_7

BACT_1202 Tolumonas auensis DSM 9187

BACT_1157 Synechococcus sp. RCC307

BACT_814 Nitratiruptor sp. SB155-2

BACT_692 Legionella drancourtii LLAP12

BACT_355 Chlorobium tepidum TLS

BACT_256 Brucella pinnipedialis B2/94

BACT_1480 Scardovia inopinata F0304

BACT_469 Desulfomicrobium baculatum DSM 4028

BACT_322 Candidatus Sulcia muelleri SMDSEM

BACT_1620 Ralstonia solanacearum CFBP2957

BACT_746 Methylibium petroleiphilum PM1

BACT_1312 Bacteroides ovatus SD CMC 3f

BACT_1366 Escherichia coli FVEC1412

BACT_1022 Ruminococcus lactaris ATCC 29176

BACT_1115 Streptococcus pyogenes MGAS10750

BACT_1302 Aerococcus viridans ATCC 11563

BACT_1431 Mycobacterium parascrofulaceum ATCC BAA-614

BACT_334 Catonella morbi ATCC 51271

BACT_995 Rickettsia prowazekii str. Madrid E

BACT_589 Geobacillus sp. G11MC16

BACT_1098 Stenotrophomonas maltophilia K279a

BACT_1447 Pediococcus acidilactici

BACT_900 Prochlorococcus marinus str. MIT 9211

BACT_374 Clostridium bolteae ATCC BAA-613

BACT_1436 Mycobacterium tuberculosis KZN R506

BACT_1602 Listeria monocytogenes 08-5578

BACT_1449 Peptoniphilus sp. oral taxon 386 str. F0131

BACT_500 Ehrlichia canis str. Jake

BACT_989 Rickettsia canadensis str. McKiel

BACT_1627 Segniliparus rotundus DSM 44985

BACT_1640 Streptomyces scabiei 87.22

BACT_238 Borrelia turicatae 91E135

BACT_739 Meiothermus ruber DSM 1279

BACT_294 Campylobacter jejuni subsp. jejuni NCTC 11168

BACT_1336 Clostridium difficile NAP07

BACT_769 Mollicutes bacterium D7

BACT_1064 Shewanella sp. ANA-3

BACT_114 Bacillus cereus B4264

BACT_153 Bacillus thuringiensis Bt407

BACT_36 Actinobacillus succinogenes 130Z

BACT_328 Carboxydibrachium pacificum DSM 12653

BACT_1177 Thermoanaerobacter pseudethanolicus ATCC 33223

BACT_811 Neorickettsia risticii str. Illinois

BACT_1070 Shigella sonnei Ss046

BACT_102 Bacillus cereus AH1134

BACT_1599 Legionella pneumophila 2300/99 Alcoy

BACT_423 Congregibacter litoralis KT71

BACT_139 Bacillus cereus W

BACT_803 Neisseria flavescens NRL30031/H210

BACT_494 Dokdonia donghaensis MED134

BACT_1023 Ruminococcus obeum ATCC 29174

BACT_472 Desulfotomaculum acetoxidans DSM 771

BACT_88 Asticcacaulis excentricus CB 48

BACT_110 Bacillus cereus ATCC 10876

BACT_1116 Streptococcus salivarius SK126

BACT_1137 Streptomyces viridochromogenes DSM 40736

BACT_1430 Mobiluncus mulieris 28-1

BACT_1025 Ruminococcus torques ATCC 27756

BACT_798 Myxococcus xanthus DK 1622

BACT_1648 Waddlia chondrophila WSU 86-1044

BACT_1170 Thalassiobium sp. R2A62

BACT_675 Lactobacillus jensenii 269-3

BACT_137 Bacillus cereus Rock4-2

BACT_1656 Acinetobacter sp. DR1

BACT_461 Deinococcus geothermalis DSM 11300

BACT_1399 Fusobacterium sp. 1_1_41FAA

BACT_560 Frankia sp. CcI3

BACT_1277 Yersinia pestis biovar Orientalis str. IP275

BACT_305 Candidatus Amoebophilus asiaticus 5a2

BACT_700 Leptotrichia buccalis DSM 1135

BACT_597 Geobacter lovleyi SZ

BACT_378 Clostridium botulinum Ba4 str. 657

BACT_267 Burkholderia cenocepacia J2315

BACT_490 Dickeya zeae Ech1591

BACT_1233 Vibrio metschnikovii CIP 69.14

BACT_1598 Legionella longbeachae NSW150

BACT_1290 Acidobacterium sp. MP5ACTX8

BACT_344 Chlamydia trachomatis 70

BACT_406 Clostridium perfringens str. 13

BACT_1351 Enterococcus faecalis S613

BACT_958 Rhizobium etli Brasil 5

BACT_1451 Peptostreptococcus anaerobius 653-L

BACT_28 Acinetobacter lwoffii SH145

BACT_820 Nitrosomonas eutropha C91

BACT_640 Hydrogenobaculum sp. Y04AAS1

BACT_242 Brachyspira murdochii DSM 12563

BACT_476 Desulfovibrio magneticus RS-1

BACT_656 Kosmotoga olearia TBF 19.5.1

BACT_1364 Escherichia coli B354

BACT_1294 Acinetobacter baumannii AB058

BACT_795 Mycoplasma pneumoniae M129

BACT_1114 Streptococcus pneumoniae TCH8431/19A

BACT_1542 Amycolatopsis mediterranei U32

BACT_1264 Xanthomonas oryzae pv. oryzicola BLS256

BACT_1108 Streptococcus dysgalactiae subsp. equisimilis GGS_124

BACT_1295 Acinetobacter baumannii AB059

BACT_42 Aeromicrobium marinum DSM 15272

BACT_681 Lactobacillus reuteri JCM 1112

BACT_230 Bordetella petrii DSM 12804

BACT_620 Hahella chejuensis KCTC 2396

BACT_50 Alcanivorax borkumensis SK2

BACT_1301 Actinomyces odontolyticus F0309

BACT_1587 Gardnerella vaginalis 409-05

BACT_1111 Streptococcus gordonii str. Challis substr. CH1

BACT_1110 Streptococcus equi subsp. zooepidemicus

BACT_1044 Salmonella enterica subsp. enterica serovar Typhi str. M223

BACT_247 Brevibacterium linens BL2

BACT_73 Anaeromyxobacter sp. K

BACT_1568 Clostridium ljungdahlii ATCC 49587

BACT_1327 Burkholderia sp. CCGE1001

BACT_1166 Syntrophobacter fumaroxidans MPOB

BACT_1117 Streptococcus sanguinis SK36

BACT_938 Pseudomonas syringae pv. tabaci ATCC 11528

BACT_265 Burkholderia ambifaria MC40-6

BACT_420 Collinsella stercoris DSM 13279

BACT_1153 Synechococcus sp. JA-2-3B

BACT_202 Baumannia cicadellinicola str. Hc

BACT_58 Alpha proteobacterium BAL199

BACT_1530 Vibrio angustum S14

BACT_508 Enterobacter cancero ATCC 35316

BACT_369 Clostridiales bacterium 1_7_47FAA strain

BACT_581 Gamma proteobacterium HTCC5015

BACT_300 Candidate division TM7 genomosp. GTL1

BACT_1124 Streptomyces coelicolor A3

BACT_582 Gamma proteobacterium NOR5-3

BACT_313 Candidatus Hamiltonella defensa 5AT

BACT_254 Brucella melitensis bv. 3

BACT_86 Arthrospira platensis str.

BACT_579 Gallionella ferruginea ES-2

BACT_1467 Pseudomonas syringae pv. Syringae 642

BACT_302 Candidate division TM7 single-cell isolate TM7b

BACT_382 Clostridium botulinum E1 str. 'BoNT E

BACT_1288 Clostridium phage D-1873

BACT_910 Propionibacterium sp. oral taxon 191 str. F0233

BACT_263 Buchnera aphidicola str. Sg

BACT_506 Endoriftia persephone

BACT_731 Marine gamma proteobacterium HTCC2080

BACT_1425 marine gamma proteobacterium HTCC2207

BACT_730 Marine actinobacterium PHSC20C1

BACT_1556 Borrelia afzelii PKo clone

BACT_327 Capnocytophaga sputigena ATCC 33612 strain Capno

BACT_583 Gamma proteobacterium NOR51-B

BACT_455 Cytophaga hutchinsonii ATCC

BACT_1319 Bacteroides sp. D4

BACT_697 Leptospira biflexa serovar Patoc strain

BACT_1 Nostoc azollae

BACT_323 Candidatus Sulcia muelleri str. Hc

BACT_321 Candidatus Ruthia magnifica str. Cm

BACT_1024 Ruminococcus sp. 5_1_39BFAA

BACT_1034 Salmonella enterica subsp. enterica serovar Choleraesuis str. SC-B67

BACT_1036 Salmonella enterica subsp. enterica serovar Enteritidis str. P125109

BACT_1039 Salmonella enterica subsp. enterica serovar Javiana str. GA_MM04042433

BACT_1040 Salmonella enterica subsp. enterica serovar Paratyphi A str. ATCC 9150

BACT_1043 Salmonella enterica subsp. enterica serovar Tennessee str. CDC07-0191

BACT_1045 Salmonella enterica subsp. enterica serovar Weltevreden str. HI_N05-537

BACT_1159 Synechococcus sp. RS9917

BACT_1210 unidentified eubacterium SCB49

BACT_1252 Wolbachia endosymbiont of Drosophila willistoni TSC

BACT_1285 Salmonella enterica subsp. enterica serovar 4

BACT_1310 Bacterium S5 strain S5

BACT_1330 Campylobacter jejuni subsp. Jejuni 1336

BACT_1331 Campylobacter jejuni subsp. Jejuni 414

BACT_1344 delta proteobacterium NaphS2

BACT_1348 Enterococcus faecalis AR01/DG

BACT_1465 Pseudomonas aeruginosa Pab1

BACT_170 Bacterium Ellin514 strain Ellin514

BACT_207 Bermanella marisrubri strain RED65

BACT_208 beta proteobacterium KB13

BACT_223 Blattabacterium sp.

BACT_249 Brucella abortus bv. 1 str. 9-941 chromosome

BACT_250 Brucella abortus bv. 3 str.

BACT_260 Buchnera aphidicola str. Bp

BACT_261 Buchnera aphidicola str. Cc

BACT_262 Buchnera aphidicola str. LSR1

BACT_301 candidate division TM7 single-cell isolate TM7a

BACT_303 candidate division TM7 single-cell isolate TM7c

BACT_317 Candidatus Pelagibacter sp. HTCC7211 1105874033148

BACT_318 Candidatus Phytoplasma australiense :

BACT_366 Citrobacter sp.

BACT_447 Cyanothece sp. ATCC 51142 chromosome circular

BACT_448 Cyanothece sp. ATCC 51142 chromosome linear

BACT_463 delta proteobacterium MLMS-1

BACT_47 Agrobacterium tumefaciens str. C58 chromosome circular

BACT_48 Agrobacterium tumefaciens str. C58 chromosome linear

BACT_557 Francisella novicida FTE

BACT_580 Gamma proteobacterium HTCC2207

BACT_59 Alpha proteobacterium HIMB114

BACT_698 Leptospira borgpetersenii serovar Hardjo-bovis L550

BACT_732 Marine gamma proteobacterium HTCC2143

BACT_733 Marine gamma proteobacterium HTCC2148

BACT_770 Moorella thermoacetica ATCC

BACT_772 Mycobacterium abscessus

BACT_789 Mycoplasma conjunctivae

BACT_790 Mycoplasma gallisepticum str. R

BACT_813 Neptuniibacter caesariensis strain MED92

BACT_888 Polynucleobacter necessarius subsp. asymbioticus

BACT_948 Ralstonia eutropha H16

BACT_997 Rickettsia sibirica 246

BACT_1088 Spirosoma linguale DSM 74

BACT_1089 Stackebrandtia nassauensis DSM 44728

BACT_1082 Sphaerobacter thermophilus DSM 20745

BACT_868 Pelodictyon luteolum DSM 273

BACT_2222 Deinococcus radiodurans R1

BACT_3001 Acinetobacter radioresistens SH164

BACT_3004 Bacteroides fragilis 3_1_12

BACT_3005 Bacteroides sp. 2_2_4

BACT_3006 Bacteroides sp. 9_1_42FAA

BACT_3007 Bacteroides sp. D1

BACT_3008 Bacteroides uniformis ATCC 8492

BACT_3010 Bacteroidetes sp. F0058

BACT_3011 Bifidobacterium adolescentis L2-32

BACT_3012 Bifidobacterium dentium ATCC 27679

BACT_3013 Bifidobacterium dentium JCVIHMP022

BACT_3014 Bifidobacterium longum infantis ATCC 55813

BACT_3015 Burkholderiales bacterium 1_1_47

BACT_3016 Campylobacter coli JV20

BACT_3017 Clostridiales genomosp. BVAB3 UPII9-5

BACT_3018 Corynebacterium accolens ATCC 49726

BACT_3020 Corynebacterium efficiens

BACT_3021 Corynebacterium glucuronalyticum ATCC 51867

BACT_3022 Corynebacterium jeikeium ATCC 43734

BACT_3024 Desulfovibrio sp. 3_1_syn3

BACT_3026 Enterococcus faecalis TX 0109

BACT_3027 Enterococcus faecalis TX 0411

BACT_3028 Enterococcus faecalis TX 0855

BACT_3029 Enterococcus faecalis TX 0860

BACT_3030 Enterococcus faecalis TX 2134

BACT_3031 Enterococcus faecalis TX 4248

BACT_3032 Enterococcus faecalis TX1322

BACT_3033 Escherichia coli O150:H5 SE15

BACT_3034 Escherichia sp. 1_1_43

BACT_3035 Eubacterium yurii margaretiae ATCC 43715

BACT_3036 Finegoldia magna ATCC 29328

BACT_3037 Finegoldia magna ATCC 53516

BACT_3041 Lactobacillus acidophilus ATCC 4796

BACT_3042 Lactobacillus brevis gravesensis ATCC 27305

BACT_3043 Lactobacillus crispatus 125-2-CHN

BACT_3045 Lactobacillus crispatus JV-V01

BACT_3046 Lactobacillus crispatus MV-1A-US

BACT_3048 Lactobacillus fermentum ATCC 14931

BACT_3049 Lactobacillus helveticus DSM 20075

BACT_3055 Lactobacillus jensenii 115-3-CHN

BACT_3056 Lactobacillus jensenii JV-V16

BACT_3057 Lactobacillus johnsonii ATCC 33200

BACT_3058 Lactobacillus paracasei ATCC 25302

BACT_3059 Lactobacillus plantarum ATCC 14917

BACT_3060 Lactobacillus reuteri CF48-3A

BACT_3061 Lactobacillus reuteri MM2-3

BACT_3062 Lactobacillus reuteri MM4-1A

BACT_3063 Lactobacillus reuteri SD2112, ATCC 55730

BACT_3064 Lactobacillus rhamnosus GG, ATCC 53103

BACT_3065 Lactobacillus salivarius ATCC 11741

BACT_3066 Leuconostoc mesenteroides cremoris ATCC 19254

BACT_3068 Mobiluncus curtisii curtisii ATCC 35241

BACT_3069 Mobiluncus mulieris ATCC 35239

BACT_3072 Neisseria meningitidis ATCC 13091

BACT_3073 Paenibacillus sp. D14

BACT_3074 Pediococcus acidilactici 7_4

BACT_3075 Pediococcus acidilactici DSM 20284

BACT_3076 Peptoniphilus duerdenii ATCC BAA-1640

BACT_3080 Prevotella marshii DSM 16973

BACT_3081 Propionibacterium acnes SK137

BACT_3082 Proteus mirabilis ATCC 29906

BACT_3085 Selenomonas sp. 67H29BP F0410

BACT_3086 Sphingobacterium spiritivorum ATCC 33861

BACT_3087 Staphylococcus aureus aureus ATCC BAA-39

BACT_3088 Staphylococcus aureus aureus MN8

BACT_3089 Staphylococcus aureus aureus TCH130/ST-72

BACT_3090 Staphylococcus aureus aureus TCH60

BACT_3091 Staphylococcus aureus aureus TCH70

BACT_3092 Staphylococcus aureus aureus USA300_TCH959

BACT_3093 Staphylococcus epidermidis BCM-HMP0060

BACT_3094 Staphylococcus epidermidis M23864:W1

BACT_3095 Staphylococcus epidermidis W23144

BACT_3096 Streptococcus bovis ATCC 700338

BACT_3097 Streptococcus gallolyticus TX20005

BACT_3098 Streptococcus mitis ATCC 6249

BACT_3099 Streptococcus pyogenes ATCC 10782

BACT_3100 Streptococcus sp. 73H25AP F0408

BACT_4001 Helicobacter pylori 51

BACT_4002 Methanosphaera stadtmanae DSM 3091

BACT_4003 Vibrio cholerae O395

BACT_4004 Streptococcus pneumoniae AP200

BACT_4005 Vibrio cholerae O395

BACT_4006 Clostridium difficile CD196

BACT_4007 Helicobacter pylori 52

BACT_4008 Mycobacterium tuberculosis KZN 4207

BACT_4009 Trypanosoma brucei gambiense DAL972

BACT_4010 Clostridium difficile M68

BACT_4011 Clostridium difficile BI1

BACT_4012 Clostridium difficile BI9

BACT_4013 Chlamydia trachomatis E/11023

BACT_4014 Chlamydia trachomatis E/150

BACT_4015 Chlamydia trachomatis G/9768

BACT_4016 Chlamydia trachomatis G/11222

BACT_4017 Chlamydia trachomatis G/11074

BACT_4018 Lactobacillus fermentum CECT 5716

BACT_4019 Lactobacillus salivarius CECT 5713

BACT_4020 Mycobacterium tuberculosis SUMu001

BACT_4021 Chlamydia trachomatis G/9301

BACT_4022 Edwardsiella tarda FL6-60

BACT_4023 Mycoplasma pneumoniae FH

BACT_4024 Chlamydia trachomatis D-EC

BACT_4025 Chlamydia trachomatis D-LC

BACT_4026 Neisseria meningitidis K1207

BACT_4027 Neisseria meningitidis S0108

BACT_4028 Mycoplasma pneumoniae FH

BACT_4029 Bacteroides fragilis 638R

BACT_4030 Helicobacter pylori v225d

BACT_4031 Providencia alcalifaciens Ban1 integrating conjugative element ICEPalban1

BACT_4032 Clostridium difficile complete genome, strain CF5

BACT_4033 Shigella flexneri 2002017

BACT_4034 Clostridium difficile complete genome, strain M120

BACT_4035 Escherichia coli O26:H11 str. 11368

BACT_4036 Gordonia bronchialis DSM 43247

BACT_4037 Photorhabdus asymbiotica subsp. asymbiotica ATCC 43949

BACT_4038 Listeria grayi DSM20601

BACT_4039 Helicobacter canadensis MIT 98-5491

BACT_4040 Escherichia coli IHE3034

BACT_4041 Neisseria meningitidis 8013

BACT_4042 Bifidobacterium animalis subsp. lactis V9

BACT_4043 Haemophilus influenzae 22.4-21

BACT_4044 Clostridium difficile ATCC 43255

BACT_4045 Escherichia coli 042

BACT_4046 Borrelia burgdorferi 297

BACT_4047 Clostridium difficile R20291

BACT_5001 Megasphaera micronuciformis

BACT_5002 Selenomonas artemidis

BACT_5003 Solobacterium moorei

BACT_5004 Treponema phagedenis (biotype Kazan 5)

BACT_5005 Actinomyces sp. oral taxon 171

ARCH_1 Acidianus ambivalens Lei 10 plasmid pDL10

ARCH_2 Acidianus hospitalis plasmid pAH1

ARCH_3 Acidilobus saccharovorans 345-15

ARCH_4 Aciduliprofundum boonei T469

ARCH_5 Aeropyrum pernix K1

ARCH_6 Archaeoglobus fulgidus DSM 4304

ARCH_7 Archaeoglobus profundus DSM 5631

ARCH_8 Archaeoglobus profundus plasmid pGS5

ARCH_9 Caldivirga maquilingensis IC-167

ARCH_10 Candidatus Korarchaeum cryptofilum OPF8

ARCH_11 Candidatus Methanoregula boonei 6A8

ARCH_12 Candidatus Methanosphaerula palustris E1-9c

ARCH_13 Cenarchaeum symbiosum A

ARCH_14 Cenarchaeum symbiosum B

ARCH_15 Desulfurococcus kamchatkensis 1221n

ARCH_16 Ferroglobus placidus DSM 10642

ARCH_17 Halalkalicoccus jeotgali B3

ARCH_18 Haloarchaeal coccus LOC-1 GN101 plasmid pHGN1

ARCH_19 Haloarcula marismortui ATCC 43049

ARCH_20 Haloarcula sp. AS7094 plasmid pSCM201

ARCH_21 Halobacterium salinarum

ARCH_22 Halobacterium salinarum R1

ARCH_23 Halobacterium sp. NRC-1

ARCH_24 Haloferax volcanii DS2

ARCH_25 Halomicrobium mukohataei DSM 12286

ARCH_26 Haloquadratum walsbyi DSM 16790

ARCH_27 Halorhabdus utahensis DSM 12940

ARCH_28 Halorubrum lacusprofundi ATCC 49239

ARCH_29 Halorubrum saccharovorum plasmid pZMX101

ARCH_30 Haloterrigena thermotolerans plasmid pSN

ARCH_31 Haloterrigena turkmenica DSM 5511

ARCH_32 Hyperthermus butylicus DSM 5456

ARCH_33 Ignicoccus hospitalis KIN4/I

ARCH_34 Ignisphaera aggregans DSM 17230

ARCH_35 Metallosphaera sedula DSM 5348

ARCH_36 Methanobrevibacter ruminantium M1

ARCH_37 Methanobrevibacter smithii ATCC 35061

ARCH_38 Methanocaldococcus fervens AG86

ARCH_39 Methanocaldococcus infernus ME c

ARCH_40 Methanocaldococcus jannaschii DSM 2661

ARCH_41 Methanocaldococcus sp. FS406-22

ARCH_43 Methanocaldococcus vulcanius M7

ARCH_44 Methanocella paludicola SANAE

ARCH_45 Methanococcoides burtonii DSM 6242

ARCH_46 Methanococcus aeolicus Nankai-3

ARCH_47 Methanococcus maripaludis C5

ARCH_48 Methanococcus maripaludis C6

ARCH_49 Methanococcus maripaludis C7

ARCH_50 Methanococcus maripaludis S2

ARCH_51 Methanococcus vannielii SB

ARCH_52 Methanococcus voltae A3

ARCH_53 Methanocorpusculum labreanum Z

ARCH_54 Methanoculleus marisnigri JR1

ARCH_55 Methanohalobium evestigatum Z-7303

ARCH_56 Methanohalophilus mahii DSM 5219

ARCH_57 Methanohalophilus mahii plasmid pML

ARCH_58 Methanoplanus petrolearius DSM 11571

ARCH_59 Methanopyrus kandleri AV19

ARCH_60 Methanosaeta thermophila PT

ARCH_61 Methanosarcina acetivorans C2A

ARCH_62 Methanosarcina barkeri str. fusaro

ARCH_63 Methanosarcina mazei Go1

ARCH_64 Methanosphaera stadtmanae DSM 3091

ARCH_65 Methanospirillum hungatei JF-1

ARCH_66 Methanothermobacter marburgensis str. Marburg

ARCH_67 Methanothermobacter thermautotrophicus Marburg plasmid pME2001

ARCH_68 Methanothermobacter thermautotrophicus plasmid pME2200

ARCH_69 Methanothermobacter thermautotrophicus str. Delta H

ARCH_70 Methanothermobacter thermautotrophicus THF plasmid pFV1

ARCH_71 Methanothermobacter thermautotrophicus Z-245 plasmid pFZ1

ARCH_72 Nanoarchaeum equitans Kin4-M

ARCH_73 Natrialba magadii ATCC 43099

ARCH_74 Natrinema sp. CX2021 plasmid pZMX201

ARCH_75 Natronobacterium sp. AS-7091 plasmid pNB101

ARCH_76 Natronomonas pharaonis DSM 2160

ARCH_77 Nitrosopumilus maritimus SCM1

ARCH_78 Picrophilus torridus DSM 9790

ARCH_79 Pyrobaculum aerophilum str. IM2

ARCH_80 Pyrobaculum arsenaticum DSM 13514

ARCH_81 Pyrobaculum calidifontis JCM 11548

ARCH_82 Pyrobaculum islandicum DSM 4184

ARCH_83 Pyrococcus abyssi GE5

ARCH_84 Pyrococcus furiosus DSM 3638

ARCH_85 Pyrococcus horikoshii OT3

ARCH_86 Pyrococcus sp. 12/1 plasmid pP12-1

ARCH_87 Pyrococcus sp. JT1 plasmid pRT1

ARCH_88 Staphylothermus hellenicus DSM 12710

ARCH_89 Staphylothermus marinus F1

ARCH_90 Sulfolobus acidocaldarius DSM 639

ARCH_91 Sulfolobus islandicus HEN7H2 plasmid pHEN7

ARCH_92 Sulfolobus islandicus L.D.8.5

ARCH_93 Sulfolobus islandicus L.S.2.15

ARCH_94 Sulfolobus islandicus M.14.25

ARCH_95 Sulfolobus islandicus M.16.27

ARCH_96 Sulfolobus islandicus M.16.4

ARCH_97 Sulfolobus islandicus plasmid pARN3

ARCH_98 Sulfolobus islandicus plasmid pARN4

ARCH_99 Sulfolobus islandicus plasmid pHVE14

ARCH_100 Sulfolobus islandicus plasmid pING1

ARCH_101 Sulfolobus islandicus plasmid pKEF9

ARCH_102 Sulfolobus islandicus plasmid pSOG1

ARCH_103 Sulfolobus islandicus plasmid pSOG2

ARCH_104 Sulfolobus islandicus plasmid pSSVx

ARCH_105 Sulfolobus islandicus plasmid pXZ1

ARCH_106 Sulfolobus islandicus REN1H1 plasmid pRN1

ARCH_107 Sulfolobus islandicus REN1H1 plasmid pRN2

ARCH_108 Sulfolobus islandicus Y.G.57.14

ARCH_109 Sulfolobus islandicus Y.N.15.51

ARCH_110 Sulfolobus islandicus Y.N.15.51 plasmid pYN01

ARCH_111 Sulfolobus neozealandicus plasmid pORA1

ARCH_112 Sulfolobus solfataricus IT3 plasmid pIT3

ARCH_113 Sulfolobus solfataricus P2

ARCH_114 Sulfolobus sp. NOB8H2 plasmid pNOB8

ARCH_115 Sulfolobus tengchongensis plasmid pTC

ARCH_116 Sulfolobus tokodaii str. 7

ARCH_117 Thermococcus gammatolerans EJ3

ARCH_118 Thermococcus kodakarensis KOD1

ARCH_119 Thermococcus nautilus 30-1 plasmid pTN1

ARCH_120 Thermococcus nautilus plasmid pTN2

ARCH_121 Thermococcus onnurineus NA1

ARCH_122 Thermococcus sibiricus MM 739

ARCH_123 Thermococcus sp. 26/2 plasmid pT26-2

ARCH_124 Thermococcus sp. AMT11 plasmid pAMT11

ARCH_125 Thermofilum pendens Hrk 5

ARCH_126 Thermoplasma acidophilum DSM 1728

ARCH_127 Thermoplasma acidophilum plasmid pTA1

ARCH_128 Thermoplasma volcanium GSS1

ARCH_129 Thermoproteus neutrophilus V24Sta

ARCH_130 Thermosphaera aggregans DSM 11486

ARCH_131 Uncultured methanogenic archaeon RC-I

ARCH_136 Sulfolobus islandicus Y.G.57.14

EUKY_261 Porphyra pulchra

EUKY_250 Desmarestia viridis

EUKY_291 Colpophyllia natans

EUKY_37 Aureoumbra lagunensis

EUKY_166 Candida orthopsilosis

EUKY_146 Vaucheria litorea

EUKY_150 Stigeoclonium helveticum

EUKY_33 Lubomirskia baicalensis

EUKY_119 Montastraea annularis

EUKY_61 Zasmidium cellare

EUKY_47 Plasmodium simium

EUKY_274 Phytophthora sojae

EUKY_152 Porphyra yezoensis

EUKY_140 Dictyostelium discoideum

EUKY_14 Micromonas sp.

EUKY_169 Moniliophthora perniciosa

EUKY_271 Allomyces macrogynus

EUKY_190 Saccharomyces pastorianus

EUKY_220 Spizellomyces punctatus

EUKY_70 Friedmanniomyces simplex

EUKY_193 Arthroderma uncinatum

EUKY_115 Dictyota dichotoma

EUKY_211 Topsentia ophiraphidites

EUKY_168 Montipora cactus

EUKY_216 Tethya actinia

EUKY_143 Monomastix sp.

EUKY_71 Falciformispora lignatilis

EUKY_104 Hyaloraphidium curvatum

EUKY_179 Ectocarpus siliculosus

EUKY_4 Phakopsora meibomiae

EUKY_318 Fusarium oxysporum

EUKY_32 Candida viswanathii

EUKY_97 Theileria annulata

EUKY_72 Endosporium populi-tremuloidis

EUKY_329 Dictyostelium fasciculatum

EUKY_278 Stylophora pistillata

EUKY_267 Monoblepharella sp.

EUKY_136 Candida dubliniensis

EUKY_57 Paramecium tetraurelia

EUKY_124 Rhizophydium sp.

EUKY_225 Zygosaccharomyces bisporus

EUKY_183 Saccharina diabolica

EUKY_230 Pleurotus ostreatus

EUKY_194 Trichophyton mentagrophytes

EUKY_118 Leishmania major

EUKY_109 Phaeodactylum tricornutum

EUKY_295 Rhodactis sp.

EUKY_309 Leucocytozoon majoris

EUKY_276 Schizosaccharomyces octosporus

EUKY_273 Phaeosphaeria nodorum

EUKY_160 Candida parapsilosis

EUKY_159 Penicillium marneffei

EUKY_290 Mussa angulosa

EUKY_172 Phytophthora infestans

EUKY_82 Mytilinidion scolecosporum

EUKY_103 Chlamydomonas eugametos

EUKY_87 Ascocratera manglicola

EUKY_297 Helicosporidium sp.

EUKY_116 Fucus vesiculosus

EUKY_78 Rhytidhysteron rufulum

EUKY_59 Phoma betae

EUKY_289 Nematostella sp.

EUKY_215 Oscarella carmela

EUKY_112 Leishmania infantum

EUKY_95 Botryosphaeria dothidea

EUKY_255 Malawimonas jakobiformis

EUKY_240 Leucocytozoon sabrazesi

EUKY_213 Ephydatia muelleri

EUKY_187 Polytomella sp.

EUKY_145 Pycnococcus provasolii

EUKY_63 Teratosphaeria fibrillosa

EUKY_300 Montastraea faveolata

EUKY_257 Gracilariopsis lemaneiformis

EUKY_182 Saccharina longipedalis

EUKY_319 Chondrus crispus

EUKY_302 Anacropora matthai

EUKY_36 Dunaliella salina

EUKY_306 Cyanophora paradoxa

EUKY_201 Igernella notabilis

EUKY_184 Saccharina ochotensis

EUKY_262 Neurospora crassa

EUKY_132 Cryptosporidium parvum

EUKY_120 Mortierella verticillata

EUKY_123 Pylaiella littoralis

EUKY_282 Pocillopora eydouxi

EUKY_275 Plasmodium gallinaceum

EUKY_177 Dendronephthya gigantea

EUKY_229 Cryphonectria parasitica

EUKY_191 Cyanidioschyzon merolae

EUKY_283 Pocillopora damicornis

EUKY_130 Acanthamoeba castellanii

EUKY_243 Tetrahymena pigmentosa

EUKY_238 Plasmodium mexicanum

EUKY_144 Pyramimonas parkeae

EUKY_314 Haemoproteus sp.

EUKY_154 Porphyra purpurea

EUKY_316 Beauveria bassiana

EUKY_79 Oedohysterium sinense

EUKY_307 Metridium senile

EUKY_58 Dictyostelium citrinum

EUKY_239 Tilletia indica

EUKY_173 Chlamydomonas reinhardtii

EUKY_200 Plakortis angulospiculatus

EUKY_299 Discosoma sp.

EUKY_117 Thalassiosira pseudonana

EUKY_304 Acropora tenuis

EUKY_332 Verticillium dahliae

EUKY_263 Zygosaccharomyces bailii

EUKY_221 Heterosigma akashiwo

EUKY_280 Seriatopora caliendrum

EUKY_125 Tetrahymena thermophila

EUKY_218 Axinella corrugata

EUKY_265 Hemiselmis andersenii

EUKY_151 Ostreococcus tauri

EUKY_305 Chlorella vulgaris

EUKY_16 Kluyveromyces lactis

EUKY_137 Babesia bovis

EUKY_298 Briareum asbestinum

EUKY_139 Theileria parva

EUKY_189 Aspergillus terreus

EUKY_122 Rhizopus oryzae

EUKY_249 Paracoccidioides brasiliensis

EUKY_23 Dekkera bruxellensis

EUKY_75 Chaetosphaeronema hispidulum

EUKY_204 Halisarca dujardini

EUKY_10 Encephalitozoon intestinalis

EUKY_66 Boeremia exigua

EUKY_245 Tetrahymena malaccensis

EUKY_133 Debaryomyces hansenii

EUKY_253 Thanatephorus cucumeris

EUKY_197 Leishmania tarentolae

EUKY_226 Lachancea waltii

EUKY_17 Ashbya gossypii

EUKY_51 Guillardia theta

EUKY_25 Eimeria tenella

EUKY_228 Blumeria graminis

EUKY_147 Oedogonium cardiacum

EUKY_232 Monosiga brevicollis

EUKY_2 Cryptomonas paramecium

EUKY_233 Candida neerlandica

EUKY_26 Toxoplasma gondii

EUKY_287 Porites porites

EUKY_331 Amphimedon queenslandica

EUKY_322 Candida zemplinina

EUKY_15 Candida glabrata

EUKY_313 Plasmodium relictum

EUKY_85 Lentithecium aquaticum

EUKY_174 Penicillium chrysogenum

EUKY_180 Saccharina coriacea

EUKY_321 Vanderwaltozyma polyspora

EUKY_68 Lindgomyces ingoldianus

EUKY_111 Bigelowiella natans

EUKY_88 Aigialus grandis

EUKY_107 Zygosaccharomyces rouxii

EUKY_326 Hydra magnipapillata

EUKY_212 Aplysina fulva

EUKY_311 Parahaemoproteus vireonis

EUKY_248 Metarhizium anisopliae

EUKY_65 Hysterobrevium mori

EUKY_129 Chrysodidymus synuroideus

EUKY_155 Odontella sinensis

EUKY_38 Aureococcus anophagefferens

EUKY_222 Nakaseomyces bacillisporus

EUKY_34 Synedra acus

EUKY_86 Hysterium barrianum

EUKY_94 Capnodium salicinum

EUKY_106 Kluyveromyces thermotolerans

EUKY_308 Leucocytozoon fringillinarum

EUKY_325 Blastocladiella emersonii

EUKY_100 Aspergillus fumigatus

EUKY_156 Euglena gracilis

EUKY_84 Leptosphaerulina australis

EUKY_315 Chaetomium globosum

EUKY_196 Trichophyton rubrum

EUKY_209 Iotrochota birotulata

EUKY_195 Glomus intraradices

EUKY_157 Hanseniaspora uvarum

EUKY_18 Alveolata sp.

EUKY_320 Tilletia walkeri

EUKY_73 Dissoconium commune

EUKY_90 Spencermartinsia viticola

EUKY_294 Ricordea florida

EUKY_50 Microsporum canis

EUKY_227 Hypocrea lixii

EUKY_69 Gloniopsis arciformis

EUKY_108 Plasmodium knowlesi

EUKY_202 Hippospongia lachne

EUKY_76 Catinella olivacea

EUKY_49 Candida maltosa

EUKY_281 Seriatopora hystrix

EUKY_48 Saccharomyces cerevisiae

EUKY_284 Savalia savaglia

EUKY_8 Acanella eburnea

EUKY_114 Trypanosoma brucei

EUKY_236 Negombata magnifica

EUKY_167 Candida metapsilosis

EUKY_210 Callyspongia plicifera

EUKY_223 Candida castellii

EUKY_64 Quadricrura septentrionalis

EUKY_208 Chondrilla aff.

EUKY_55 Oltmannsiellopsis viridis

EUKY_56 Emiliania huxleyi

EUKY_40 Yarrowia lipolytica

EUKY_231 Cyanidium caldarium

EUKY_44 Cordyceps brongniartii

EUKY_242 Ustilago maydis

EUKY_21 Paramecium aurelia

EUKY_11 Micromonas pusilla

EUKY_67 Lindgomyces breviappendiculatus

EUKY_277 Hypocrea jecorina

EUKY_171 Lecanicillium muscarium

EUKY_52 Magnaporthe oryzae

EUKY_165 Pichia farinosa

EUKY_288 Pavona clavus

EUKY_105 Pedinomonas minor

EUKY_135 Pichia pastoris

EUKY_164 Chattonella marina

EUKY_185 Saccharina religiosa

EUKY_101 Plasmodium reichenowi

EUKY_312 Plasmodium fragile

EUKY_102 Tetrahymena pyriformis

EUKY_234 Aphrocallistes vastus

EUKY_126 Ochromonas danica

EUKY_310 Haemoproteus columbae

EUKY_286 Siderastrea radians

EUKY_293 Agaricia humilis

EUKY_77 Astrosphaeriella aggregata

EUKY_254 Laminaria digitata

EUKY_205 Xestospongia muta

EUKY_19 Chromera velia

EUKY_237 Plasmodium floridense

EUKY_206 Ectyoplasia ferox

EUKY_217 Geodia neptuni

EUKY_251 Aspergillus tubingensis

EUKY_170 Pseudendoclonium akinetum

EUKY_269 Schizophyllum commune

EUKY_46 Plasmodium vivax

EUKY_134 Encephalitozoon cuniculi

EUKY_91 Guignardia bidwellii

EUKY_1 Pythium ultimum

EUKY_199 Vaceletia sp.

EUKY_138 Candida albicans

EUKY_224 Nakaseomyces delphensis

EUKY_241 Aurelia aurita

EUKY_22 Plasmodium falciparum

EUKY_83 Lophiotrema brunneosporum

EUKY_131 Prototheca wickerhamii

EUKY_198 Cinachyrella kuekenthali

EUKY_6 Floydiella terrestris

EUKY_148 Leptosira terrestris

EUKY_324 Plasmodium juxtanucleare

EUKY_296 Pseudopterogorgia bipinnata

EUKY_207 Ptilocaulis walpersi

EUKY_149 Rhodomonas salina

EUKY_323 Mycosphaerella graminicola

EUKY_301 Montastraea franksi

EUKY_292 Astrangia sp.

EUKY_62 Venturia inaequalis

EUKY_141 Bryopsis hypnoides

EUKY_203 Agelas schmidti

EUKY_244 Tetrahymena paravorax

EUKY_27 Schizosaccharomyces pombe

EUKY_279 Madracis mirabilis

EUKY_30 Trametes cingulata

EUKY_12 Cryptococcus neoformans

EUKY_162 Saccharomyces castellii

EUKY_93 Dothidea insculpta

EUKY_42 Kryptoperidinium foliaceum

EUKY_330 Fusarium proliferatum

EUKY_81 Mytilinidion resinicola

EUKY_247 Nephroselmis olivacea

EUKY_127 Physarum polycephalum

EUKY_43 Paramecium caudatum

EUKY_235 Suberites domuncula

EUKY_178 Aspergillus niger

EUKY_96 Coprinus comatus

EUKY_303 Gracilaria tenuistipitata

EUKY_3 Candida subhashii

EUKY_29 Hartmannella vermiformis

EUKY_285 Chrysopathes formosa

EUKY_142 Parachlorella kessleri

EUKY_328 Pichia canadensis

EUKY_13 Ostreococcus lucimarinus

EUKY_28 Gibberella zeae

EUKY_259 Dictyostelium firmibasis

EUKY_31 Candida sojae

EUKY_74 Corynespora cassiicola

EUKY_60 Tetraplosphaeria sasicola

EUKY_268 Schizosaccharomyces japonicus

EUKY_89 Westerdykella cylindrica

EUKY_80 Oedohysterium insidens

EUKY_252 Saprolegnia ferax

EUKY_270 Cafeteria roenbergensis

EUKY_186 Saccharina japonica

EUKY_99 Trichoplax adhaerens

EUKY_9 Naegleria gruberi

EUKY_5 Phakopsora pachyrhizi

EUKY_219 Keratoisidinae sp.

EUKY_272 Polytomella capuana

EUKY_121 Smittium culisetae

EUKY_20 Reclinomonas americana

EUKY_113 Leishmania braziliensis

EUKY_246 Aspergillus oryzae

EUKY_256 Gracilaria chilensis

EUKY_24 Brettanomyces custersianus

EUKY_175 Pneumocystis carinii

EUKY_161 Saccharomyces servazzii

EUKY_188 Aspergillus nidulans

EUKY_181 Saccharina angustata

EUKY_192 Arthroderma obtusum

EUKY_39 Scheffersomyces stipitis

EUKY_110 Paulinella chromatophora

EUKY_176 Ircinia strobilina

EUKY_128 Scenedesmus obliquus

EUKY_98 Placozoan sp.

EUKY_317 Phytophthora ramorum

EUKY_7 Proteromonas lacertae

EUKY_45 Hydra oligactis

EUKY_54 Podospora anserina

EUKY_153 Euglena longa

EUKY_41 Durinskia baltica

EUKY_92 Aliquandostipite khaoyaiensis

EUKY_158 Epidermophyton floccosum

EUKY_264 Zygosaccharomyces fermentati

EUKY_260 Dictyostelium giganteum

EUKY_214 Amphimedon compressa

VIRL_134 Abaca bunchy top virus DNA-M

VIRL_136 Abaca bunchy top virus DNA-N

VIRL_133 Abaca bunchy top virus DNA-R

VIRL_135 Abaca bunchy top virus DNA-S

VIRL_2784 Abaca bunchy top virus segment 2

VIRL_2783 Abaca bunchy top virus segment 5

VIRL_2740 Abalone shriveling syndrome-associated virus

VIRL_1325 Abelson murine leukemia virus

VIRL_271 Abutilon Brazil virus DNA A

VIRL_270 Abutilon Brazil virus DNA B

VIRL_3400 Abutilon mosaic virus DNA A

VIRL_3399 Abutilon mosaic virus DNA B

VIRL_1101 Acanthamoeba polyphaga mimivirus

VIRL_1883 Acanthocystis turfacea Chlorella virus 1

VIRL_2422 Acheta domesticus densovirus

VIRL_2398 Acholeplasma phage L2

VIRL_2402 Acholeplasma phage MV-L1

VIRL_1716 Acidianus bottle-shaped virus

VIRL_640 Acidianus filamentous virus 1

VIRL_557 Acidianus filamentous virus 2

VIRL_546 Acidianus filamentous virus 3

VIRL_549 Acidianus filamentous virus 6

VIRL_548 Acidianus filamentous virus 7

VIRL_547 Acidianus filamentous virus 8

VIRL_540 Acidianus filamentous virus 9

VIRL_554 Acidianus rod-shaped virus 1

VIRL_466 Acidianus spindle-shaped virus 1

VIRL_604 Acidianus two-tailed virus

VIRL_2622 Acinetobacter phage AP205

VIRL_3505 Aconitum latent virus

VIRL_2537 Actinomyces phage Av-1

VIRL_2077 Actinoplanes phage phiAsp2

VIRL_3354 Acute bee paralysis virus

VIRL_2628 Acyrthosiphon pisum bacteriophage APSE-1

VIRL_1216 Acyrthosiphon pisum virus

VIRL_920 Adeno-associated virus - 1

VIRL_3486 Adeno-associated virus - 2

VIRL_218 Adeno-associated virus - 3

VIRL_922 Adeno-associated virus - 4

VIRL_905 Adeno-associated virus - 7

VIRL_904 Adeno-associated virus - 8

VIRL_2929 Adeno-associated virus 5

VIRL_2119 Adoxophyes honmai NPV

VIRL_1142 Adoxophyes orana granulovirus

VIRL_3533 Adoxophyes orana nucleopolyhedrovirus

VIRL_3577 Adult diarrheal rotavirus strain J19

VIRL_3575 Adult diarrheal rotavirus strain J19

VIRL_3574 Adult diarrheal rotavirus strain J19

VIRL_3576 Adult diarrheal rotavirus strain J19

VIRL_3570 Adult diarrheal rotavirus strain J19

VIRL_3571 Adult diarrheal rotavirus strain J19

VIRL_3573 Adult diarrheal rotavirus strain J19

VIRL_3578 Adult diarrheal rotavirus strain J19

VIRL_3572 Adult diarrheal rotavirus strain J19

VIRL_2883 Adult diarrheal rotavirus strain J19

VIRL_2884 Adult diarrheal rotavirus strain J19

VIRL_826 Aedes aegypti densovirus

VIRL_2155 Aedes albopictus densovirus

VIRL_125 Aedes flavivirus

VIRL_1567 Aedes pseudoscutellaris reovirus segment 1

VIRL_1566 Aedes pseudoscutellaris reovirus segment 2

VIRL_1565 Aedes pseudoscutellaris reovirus segment 3

VIRL_1564 Aedes pseudoscutellaris reovirus segment 4

VIRL_1563 Aedes pseudoscutellaris reovirus segment 5

VIRL_1562 Aedes pseudoscutellaris reovirus segment 6

VIRL_1561 Aedes pseudoscutellaris reovirus segment 7

VIRL_1560 Aedes pseudoscutellaris reovirus segment 8

VIRL_1559 Aedes pseudoscutellaris reovirus segment 9

VIRL_1927 Aedes taeniorhynchus iridescent virus

VIRL_586 Aeromonas phage 25

VIRL_617 Aeromonas phage 31

VIRL_648 Aeromonas phage 44RR2.8t

VIRL_646 Aeromonas phage Aeh1

VIRL_560 Aeromonas phage phiO18P

VIRL_2493 African cassava mosaic virus DNA A

VIRL_2492 African cassava mosaic virus DNA B

VIRL_656 African green monkey polyomavirus

VIRL_165 African horsesickness virus chromosome segment 5

VIRL_351 African horsesickness virus chromosome segment 9

VIRL_745 African horsesickness virus segment 1

VIRL_2946 African horsesickness virus segment 10

VIRL_1021 African horsesickness virus segment 2

VIRL_2938 African horsesickness virus segment 3

VIRL_2943 African horsesickness virus segment 4

VIRL_2937 African horsesickness virus segment 6

VIRL_2944 African horsesickness virus segment 7

VIRL_2939 African horsesickness virus segment 8

VIRL_2669 African oil palm ringspot virus

VIRL_1272 African swine fever virus

VIRL_3289 Ageratum enation virus

VIRL_2642 Ageratum leaf Cameroon betasatellite

VIRL_3014 Ageratum leaf curl disease associated sequence virion

VIRL_2061 Ageratum leaf curl virus - [G52]

VIRL_2722 Ageratum yellow leaf curl beta ‚Äì [Pakistan:Lahore

VIRL_3126 Ageratum yellow vein China virus

VIRL_2896 Ageratum yellow vein China virus-associated DNA beta

VIRL_853 Ageratum yellow vein Hualian virus-[Taiwan:Hsinchu:2003] DNA A

VIRL_919 Ageratum yellow vein Sri Lanka virus segment A

VIRL_3044 Ageratum yellow vein Taiwan virus

VIRL_3045 Ageratum yellow vein virus

VIRL_1507 Ageratum yellow vein virus-associated DNA 1

VIRL_3294 Ageratum yellow vein virus-associated DNA beta

VIRL_279 Aggregatibacter phage S1249

VIRL_1454 Agropyron mosaic virus

VIRL_1598 Agrotis ipsilon multiple nucleopolyhedrovirus

VIRL_975 Agrotis segetum granulovirus

VIRL_955 Agrotis segetum nucleopolyhedrovirus

VIRL_3402 Aichi virus

VIRL_1673 Akabane virus segment L

VIRL_2801 Akabane virus segment M

VIRL_2800 Akabane virus segment S

VIRL_61 Alcelaphine herpesvirus 1

VIRL_1271 Aleutian mink disease virus

VIRL_2386 Alfalfa mosaic virus RNA 1

VIRL_3462 Alfalfa mosaic virus RNA 2

VIRL_3461 Alfalfa mosaic virus RNA 3

VIRL_1539 Algerian watermelon mosaic virus

VIRL_3067 Alkhurma virus

VIRL_845 Allamanda leaf curl virus DNA-A

VIRL_1584 Allium virus X

VIRL_1660 Allpahuayo virus segment L

VIRL_2787 Allpahuayo virus segment S

VIRL_2004 Alstroemeria virus x

VIRL_1957 Alternanthera mosaic virus

VIRL_2893 Alternanthera yellow vein virus DNA-A

VIRL_1698 Alternanthera yellow vein virus satellite DNA beta

VIRL_1299 Alternaria alternata dsRNA mycovirus segment L

VIRL_1292 Alternaria alternata dsRNA mycovirus segment M1

VIRL_1291 Alternaria alternata dsRNA mycovirus segment M2

VIRL_1290 Alternaria alternata dsRNA mycovirus segment S

VIRL_2788 Amapari virus segment L

VIRL_2789 Amapari virus segment S

VIRL_2795 Amasya cherry disease associated chrysovirus segment 1

VIRL_2796 Amasya cherry disease associated chrysovirus segment 2

VIRL_2797 Amasya cherry disease associated chrysovirus segment 3

VIRL_2798 Amasya cherry disease associated chrysovirus segment 4

VIRL_2918 Amasya cherry disease-associated mycovirus

VIRL_2919 Amasya cherry disease-associated mycovirus

VIRL_2963 Ambystoma tigrinum virus

VIRL_3650 American grass carp reovirus segment 1

VIRL_3642 American grass carp reovirus segment 10

VIRL_3641 American grass carp reovirus segment 11

VIRL_3649 American grass carp reovirus segment 2

VIRL_3648 American grass carp reovirus segment 3

VIRL_3647 American grass carp reovirus segment 4

VIRL_2777 American grass carp reovirus segment 5

VIRL_3646 American grass carp reovirus segment 6

VIRL_3645 American grass carp reovirus segment 7

VIRL_3644 American grass carp reovirus segment 8

VIRL_3643 American grass carp reovirus segment 9

VIRL_1233 American plum line pattern virus RNA1

VIRL_1232 American plum line pattern virus RNA2

VIRL_1231 American plum line pattern virus RNA3

VIRL_686 Amsacta moorei entomopoxvirus 'L'

VIRL_3514 Anagyris vein yellowing virus

VIRL_1229 Andes virus segment L

VIRL_1230 Andes virus segment M

VIRL_3284 Andes virus segment S

VIRL_1332 Angelonia flower break virus

VIRL_767 Anguillid herpesvirus 1

VIRL_3562 Anopheles gambiae densonucleosis virus

VIRL_2859 Antheraea pernyi nucleopolyhedrovirus

VIRL_1900 Anticarsia gemmatalis nucleopolyhedrovirus

VIRL_1194 Aphid lethal paralysis virus

VIRL_2248 Apoi virus

VIRL_3483 Apple chlorotic leaf spot virus

VIRL_2219 Apple latent spherical virus segment 1

VIRL_2218 Apple latent spherical virus segment 2

VIRL_2308 Apple mosaic virus RNA 1

VIRL_2307 Apple mosaic virus RNA 2

VIRL_3275 Apple mosaic virus RNA 3

VIRL_3423 Apple stem grooving virus

VIRL_3285 Apple stem pitting virus

VIRL_2407 Apricot pseudo-chlorotic leaf spot virus

VIRL_1979 Aquareovirus A segment 1

VIRL_1441 Aquareovirus A segment 10

VIRL_1440 Aquareovirus A segment 11

VIRL_2875 Aquareovirus A segment 2

VIRL_1978 Aquareovirus A segment 3

VIRL_1444 Aquareovirus A segment 4

VIRL_1439 Aquareovirus A segment 6

VIRL_1443 Aquareovirus A segment 8

VIRL_1442 Aquareovirus A segment 9

VIRL_2295 Arabis mosaic virus large satellite RNA

VIRL_2931 Arabis mosaic virus RNA 1

VIRL_2932 Arabis mosaic virus RNA 2

VIRL_2384 Arabis mosaic virus small satellite RNA

VIRL_1888 Archaeal BJ1 virus

VIRL_2403 Artichoke mottled crinkle virus

VIRL_1364 Asparagus virus 2 RNA 1

VIRL_1363 Asparagus virus 2 RNA 2

VIRL_1595 Asparagus virus 2 RNA 3

VIRL_1648 Asparagus virus 3

VIRL_3549 Astrovirus MLB1

VIRL_50 Astrovirus MLB1 HK05

VIRL_1341 Astrovirus VA1

VIRL_695 Ateline herpesvirus 3

VIRL_2306 Atkinsonella hypoxylon partitivirus RNA 1

VIRL_2305 Atkinsonella hypoxylon partitivirus RNA 2

VIRL_2304 Atkinsonella hypoxylon partitivirus RNA 3

VIRL_1036 Atlantic salmon swim bladder sarcoma virus

VIRL_1493 Aura virus

VIRL_1236 Australian bat lyssavirus

VIRL_764 Autographa californica nucleopolyhedrovirus

VIRL_3021 Avian adeno-associated virus ATCC VR-865

VIRL_205 Avian adeno-associated virus strain DA-1

VIRL_2400 Avian carcinoma virus

VIRL_3136 Avian encephalomyelitis virus

VIRL_2953 Avian endogenous retrovirus EAV-HP

VIRL_2494 Avian infectious bronchitis virus

VIRL_3484 Avian leukosis virus - RSA

VIRL_2871 Avian metapneumovirus

VIRL_1264 Avian myelocytomatosis virus

VIRL_990 Avian paramyxovirus 6

VIRL_987 Avian sapelovirus

VIRL_2709 Azospirillum phage Cd

VIRL_1552 Bacillus phage 0305phi8-36

VIRL_515 Bacillus phage AP50

VIRL_2608 Bacillus phage B103

VIRL_2588 Bacillus phage Bam35c

VIRL_1569 Bacillus phage BCJA1c

VIRL_2003 Bacillus phage Cherry

VIRL_1954 Bacillus phage Fah

VIRL_2624 Bacillus phage GA-1

VIRL_2889 Bacillus phage Gamma

VIRL_2568 Bacillus phage GIL16c

VIRL_3586 Bacillus phage IEBH

VIRL_2715 Bacillus phage phi105

VIRL_536 Bacillus phage phi29

VIRL_2365 Bacillus phage SPBc2

VIRL_1343 Bacillus phage SPO1

VIRL_2424 Bacillus phage SPP1

VIRL_507 Bacillus phage TP21-L

VIRL_2560 Bacillus phage WBeta

VIRL_1395 Bacillus prophage phBC6A51

VIRL_1394 Bacillus prophage phBC6A52

VIRL_1687 Bacillus virus 1

VIRL_2413 Bacteriophage Aaphi23

VIRL_511 Bacteriophage APSE-2

VIRL_2680 Bacteriophage PSA

VIRL_3584 Bacteroides phage B40-8

VIRL_2666 Bagaza virus

VIRL_3437 Bamboo mosaic virus

VIRL_1228 Bamboo mosaic virus satellite RNA

VIRL_429 Banana bract mosaic virus

VIRL_3276 Banana bunchy top virus DNA 1

VIRL_3280 Banana bunchy top virus DNA 2

VIRL_3282 Banana bunchy top virus DNA 3

VIRL_3281 Banana bunchy top virus DNA 4

VIRL_3278 Banana bunchy top virus DNA 5

VIRL_3279 Banana bunchy top virus DNA 6

VIRL_3637 Banana mild mosaic virus

VIRL_1446 Banana streak GF virus

VIRL_2911 Banana streak Mysore virus

VIRL_2311 Banana streak OL virus

VIRL_2861 Banana streak virus

VIRL_3669 Banana streak virus strain Acuminata Vietnam

VIRL_550 Bandicoot papillomatosis carcinomatosis virus type 1

VIRL_537 Bandicoot papillomatosis carcinomatosis virus type 2

VIRL_147 Banna virus segment 1

VIRL_3106 Banna virus segment 10

VIRL_3107 Banna virus segment 11

VIRL_3108 Banna virus segment 12

VIRL_146 Banna virus segment 2

VIRL_145 Banna virus segment 3

VIRL_144 Banna virus segment 4

VIRL_143 Banna virus segment 5

VIRL_142 Banna virus segment 6

VIRL_3103 Banna virus segment 7

VIRL_3104 Banna virus segment 8

VIRL_3105 Banna virus segment 9

VIRL_1327 Barfin flounder nervous necrosis virus RNA 1

VIRL_1326 Barfin flounder nervous necrosis virus RNA 2

VIRL_2646 Barfin flounder virus BF93Hok RNA1

VIRL_2645 Barfin flounder virus BF93Hok RNA2

VIRL_855 Barley dwarf virus

VIRL_3272 Barley mild mosaic virus RNA 1

VIRL_3273 Barley mild mosaic virus RNA2

VIRL_3283 Barley stripe mosaic virus RNA 1

VIRL_3274 Barley stripe mosaic virus RNA 2

VIRL_3277 Barley stripe mosaic virus RNA 3

VIRL_2246 Barley yellow dwarf virus - MAV

VIRL_2415 Barley yellow dwarf virus - PAV

VIRL_1190 Barley yellow dwarf virus-GAV

VIRL_2352 Barley yellow dwarf virus-PAS

VIRL_3343 Barley yellow mosaic virus RNA 1

VIRL_3342 Barley yellow mosaic virus RNA 2

VIRL_2474 Barmah Forest virus

VIRL_432 Basella rugose mosaic virus

VIRL_33 Bat adeno-associated virus YNM

VIRL_3672 Bat coronavirus (BtCoV/133/2005)

VIRL_1545 Bat coronavirus 1A

VIRL_1546 Bat coronavirus 1B

VIRL_52 Bat coronavirus BM48-31/BGR/2008

VIRL_2794 Bat coronavirus HKU2

VIRL_2811 Bat coronavirus HKU3

VIRL_2834 Bat coronavirus HKU4-1

VIRL_2833 Bat coronavirus HKU5-1

VIRL_1544 Bat coronavirus HKU8

VIRL_2832 Bat coronavirus HKU9-1

VIRL_138 Bat SARS CoV Rf1/2004

VIRL_137 Bat SARS CoV Rm1/2004

VIRL_1688 Bat SARS CoV Rp3/2004

VIRL_1516 Bdellovibrio phage phiMH2K

VIRL_921 Beak and feather disease virus

VIRL_2300 Bean calico mosaic virus DNA A

VIRL_2299 Bean calico mosaic virus DNA B

VIRL_3130 Bean common mosaic necrosis virus

VIRL_3296 Bean common mosaic virus

VIRL_1531 Bean dwarf mosaic virus DNA A

VIRL_1532 Bean dwarf mosaic virus DNA B

VIRL_2176 Bean golden mosaic virus DNA A

VIRL_2175 Bean golden mosaic virus DNA B

VIRL_3476 Bean golden yellow mosaic virus DNA A

VIRL_3477 Bean golden yellow mosaic virus DNA B

VIRL_372 Bean leafroll virus

VIRL_3268 Bean pod mottle virus RNA 1

VIRL_3269 Bean pod mottle virus RNA 2

VIRL_1646 Bean yellow dis virus RNA 1

VIRL_1645 Bean yellow dis virus RNA 2

VIRL_3270 Bean yellow dwarf virus

VIRL_3271 Bean yellow mosaic virus

VIRL_2786 Bear Canyon virus segment L

VIRL_2785 Bear Canyon virus segment S

VIRL_3061 Beet black scorch virus

VIRL_1309 Beet black scorch virus satellite RNA

VIRL_3345 Beet chlorosis virus

VIRL_445 Beet cryptic virus 1 RNA 1

VIRL_444 Beet cryptic virus 1 RNA 2

VIRL_2779 Beet curly top Iran virus-[K]

VIRL_2497 Beet curly top virus - California [Logan]

VIRL_3025 Beet mild curly top virus - [Worland4]

VIRL_2436 Beet mild yellowing virus

VIRL_2973 Beet mosaic virus

VIRL_3265 Beet necrotic yellow vein virus RNA 1

VIRL_2434 Beet necrotic yellow vein virus RNA 2

VIRL_3264 Beet necrotic yellow vein virus RNA 3

VIRL_3263 Beet necrotic yellow vein virus RNA 4

VIRL_3266 Beet necrotic yellow vein virus RNA 5

VIRL_1320 Beet pseudo-yellows virus RNA 1

VIRL_1319 Beet pseudo-yellows virus RNA 2

VIRL_2244 Beet ringspot virus RNA 1

VIRL_2243 Beet ringspot virus RNA 2

VIRL_3024 Beet severe curly top virus - Cfh

VIRL_3508 Beet soil-borne mosaic virus RNA 2

VIRL_3507 Beet soil-borne mosaic virus RNA1

VIRL_1227 Beet soil-borne mosaic virus RNA3

VIRL_1226 Beet soil-borne mosaic virus RNA4

VIRL_3260 Beet soil-borne virus RNA 1

VIRL_3262 Beet soil-borne virus RNA 2

VIRL_3261 Beet soil-borne virus RNA 3

VIRL_1579 Beet virus Q RNA 1

VIRL_2297 Beet virus Q RNA 2

VIRL_2296 Beet virus Q RNA 3

VIRL_1207 Beet western yellows ST9 associated virus

VIRL_2115 Beet western yellows virus

VIRL_3444 Beet yellows virus

VIRL_2910 Begomovirus-associated DNA-II

VIRL_1447 Begomovirus-associated DNA-III

VIRL_1955 Beilong virus

VIRL_1689 Bell pepper mottle tobamovirus

VIRL_993 Beluga Whale coronavirus SW1

VIRL_265 Bettongia penicillata papillomavirus 1

VIRL_2685 Bhendi yellow vein Bhubhaneswar virus DNA-A

VIRL_1594 Bhendi yellow vein Delhi virus [2004:New Delhi] DNA-A

VIRL_3290 Bhendi yellow vein mosaic virus

VIRL_3293 Bhendi yellow vein mosaic virus-associated DNA beta

VIRL_175 Bidens mottle virus

VIRL_1035 Bitter gourd leaf curl disease-associated DNA beta

VIRL_723 BK polyomavirus

VIRL_2399 Black beetle virus

VIRL_2357 Black beetle virus RNA 2

VIRL_366 Black queen cell virus

VIRL_434 Black raspberry necrosis virus RNA1

VIRL_770 Black raspberry necrosis virus RNA2

VIRL_1677 Black raspberry virus F

VIRL_446 Blackberry chlorotic ringspot virus

VIRL_448 Blackberry chlorotic ringspot virus RNA1

VIRL_447 Blackberry chlorotic ringspot virus RNA2

VIRL_3660 Blackberry virus Y

VIRL_425 Blackberry yellow vein-associated virus RNA1

VIRL_141 Blackberry yellow vein-associated virus RNA2

VIRL_2301 Blackcurrant reversion virus RNA 2

VIRL_2298 Blackcurrant reversion virus RNA1

VIRL_1214 Blackcurrant reversion virus satellite RNA

VIRL_848 Blainvillea yellow spot virus DNA-A

VIRL_847 Blainvillea yellow spot virus DNA-B

VIRL_909 Blattella germanica densovirus

VIRL_2076 Blotched snakehead virus

VIRL_2075 Blotched snakehead virus

VIRL_760 Blueberry red ringspot virus

VIRL_2435 Blueberry scorch virus

VIRL_2935 Bluetongue virus segment 1

VIRL_2940 Bluetongue virus segment 10

VIRL_2942 Bluetongue virus segment 2

VIRL_2941 Bluetongue virus segment 3

VIRL_2934 Bluetongue virus segment 4

VIRL_2933 Bluetongue virus segment 5

VIRL_2945 Bluetongue virus segment 6

VIRL_2936 Bluetongue virus segment 7

VIRL_2948 Bluetongue virus segment 8

VIRL_2947 Bluetongue virus segment 9

VIRL_76 Bocavirus gorilla/GBoV1/2009

VIRL_407 Bombyx mandarina nucleopolyhedrovirus

VIRL_2702 Bombyx mori cypovirus 1 satellite RNA

VIRL_2154 Bombyx mori densovirus 5

VIRL_387 Bombyx mori NPV

VIRL_2162 Boolarra virus RNA 2

VIRL_1197 Boolarra virus RNA1

VIRL_3222 B disease virus X818

VIRL_2409 Bordetella phage BIP-1

VIRL_2410 Bordetella phage BMP-1

VIRL_2584 Bordetella phage BPP-1

VIRL_36 Borna disease virus

VIRL_1652 Botryotinia fuckeliana partitivirus 1 RNA1

VIRL_1651 Botryotinia fuckeliana partitivirus 1 RNA2

VIRL_1650 Botryotinia fuckeliana partitivirus 1 RNA3

VIRL_1722 Botryotinia fuckeliana totivirus 1

VIRL_3551 Botrytis cinerea debilitation-related virus

VIRL_1248 Botrytis virus F

VIRL_1134 Botrytis virus X

VIRL_3509 Bougainvillea spectabilis chlorotic vein-banding virus

VIRL_229 Bovine adeno-associated virus

VIRL_2063 Bovine adenovirus A

VIRL_1357 Bovine adenovirus B

VIRL_2366 Bovine adenovirus B

VIRL_2324 Bovine adenovirus D

VIRL_3319 Bovine coronavirus

VIRL_3411 Bovine enterovirus

VIRL_1519 Bovine ephemeral fever virus

VIRL_1266 Bovine foamy virus

VIRL_63 Bovine herpesvirus 1

VIRL_683 Bovine herpesvirus 4

VIRL_262 Bovine herpesvirus 5

VIRL_3482 Bovine immunodeficiency virus

VIRL_2149 Bovine kobuvirus

VIRL_3481 Bovine leukemia virus

VIRL_728 Bovine papillomavirus - 1

VIRL_166 Bovine papillomavirus - 11

VIRL_660 Bovine papillomavirus 3

VIRL_559 Bovine papillomavirus 8

VIRL_661 Bovine papillomavirus type 5

VIRL_544 Bovine papillomavirus-10

VIRL_545 Bovine papillomavirus-9

VIRL_643 Bovine papular stomatitis virus

VIRL_3369 Bovine parainfluenza virus 3

VIRL_1280 Bovine parvovirus

VIRL_2064 Bovine parvovirus 2

VIRL_734 Bovine polyomavirus

VIRL_118 Bovine respiratory coronavirus AH187

VIRL_117 Bovine respiratory coronavirus bovine/US/OH-440-TC/1996

VIRL_1315 Bovine respiratory syncytial virus

VIRL_981 Bovine rhinitis B virus

VIRL_3470 Bovine viral diarrhea virus 1

VIRL_963 Bovine viral diarrhea virus 3 Th/04_KhonKaen

VIRL_3414 Bovine viral diarrhea virus genotype 2

VIRL_1080 Breda virus

VIRL_1712 Brevicoryne brassicae picorna-like virus

VIRL_2183 Broad bean mottle virus RNA 1

VIRL_3134 Broad bean mottle virus RNA 2

VIRL_2184 Broad bean mottle virus RNA 3

VIRL_2148 Broad bean necrosis virus RNA 1

VIRL_2147 Broad bean necrosis virus RNA 2

VIRL_2146 Broad bean necrosis virus RNA 3

VIRL_2978 Broad bean wilt virus 1 RNA 1

VIRL_2977 Broad bean wilt virus 1 RNA 2

VIRL_3341 Broad bean wilt virus 2 RNA1

VIRL_3340 Broad bean wilt virus 2 RNA2

VIRL_3460 Brome mosaic virus RNA 1

VIRL_3459 Brome mosaic virus RNA 2

VIRL_3458 Brome mosaic virus RNA 3

VIRL_2302 Brome streak mosaic virus

VIRL_249 Broome virus segment L1

VIRL_248 Broome virus segment L2

VIRL_247 Broome virus segment L3

VIRL_246 Broome virus segment M1

VIRL_245 Broome virus segment M2

VIRL_244 Broome virus segment M3

VIRL_243 Broome virus segment S1

VIRL_242 Broome virus segment S2

VIRL_241 Broome virus segment S3

VIRL_240 Broome virus segment S4

VIRL_428 Brugmansia mild mottle virus

VIRL_11 Brugmansia suaveolens mottle virus

VIRL_655 Budgerigar fledgling disease polyomavirus

VIRL_164 Bundibugyo ebolavirus

VIRL_2363 Bunyamwera virus L segment

VIRL_3401 Bunyamwera virus M segment

VIRL_176 Bunyamwera virus segment S

VIRL_1727 Burkholderia ambifaria phage BcepF1

VIRL_2587 Burkholderia phage Bcep1

VIRL_2001 Burkholderia phage Bcep176

VIRL_2091 Burkholderia phage Bcep22

VIRL_2585 Burkholderia phage Bcep43

VIRL_2601 Burkholderia phage Bcep781

VIRL_2577 Burkholderia phage BcepB1A

VIRL_2576 Burkholderia phage BcepC6B

VIRL_562 Burkholderia phage BcepGomr

VIRL_1413 Burkholderia phage BcepIL02

VIRL_2578 Burkholderia phage BcepMu

VIRL_2098 Burkholderia phage BcepNazgul

VIRL_2538 Burkholderia phage BcepNY3

VIRL_2686 Burkholderia phage KS10

VIRL_148 Burkholderia phage KS9

VIRL_2088 Burkholderia phage phi1026b

VIRL_748 Burkholderia phage phi644-2 chromosome

VIRL_416 Burkholderia phage phiE12-2 chromosome

VIRL_2616 Burkholderia phage phiE125

VIRL_417 Burkholderia phage phiE202 chromosome

VIRL_415 Burkholderia phage phiE255 chromosome

VIRL_606 Burkholderia prophage phi52237

VIRL_1381 Bussuquara virus

VIRL_1027 Butterbur mosaic virus

VIRL_3150 Cabbage leaf curl virus DNA A

VIRL_3146 Cabbage leaf curl virus DNA B

VIRL_261 Cacao swollen shoot virus

VIRL_1365 Cactus mild mottle virus

VIRL_746 Cactus virus X

VIRL_103 Calicivirus isolate Allston 2008/US

VIRL_104 Calicivirus isolate Allston 2009/US

VIRL_2044 Calicivirus isolate TCG

VIRL_954 Calicivirus pig/AB90/CAN

VIRL_1203 Calicivirus strain NB

VIRL_827 California sea lion anellovirus

VIRL_99 California sea lion polyomavirus 1

VIRL_659 Callitrichine herpesvirus 3

VIRL_676 Camelpox virus

VIRL_1153 Campoletis sonorensis ichnovirus chromosome segment W

VIRL_2862 Campoletis sonorensis ichnovirus segment B

VIRL_785 Campoletis sonorensis ichnovirus segment C

VIRL_771 Campoletis sonorensis ichnovirus segment D

VIRL_784 Campoletis sonorensis ichnovirus segment E

VIRL_773 Campoletis sonorensis ichnovirus segment F

VIRL_783 Campoletis sonorensis ichnovirus segment G

VIRL_782 Campoletis sonorensis ichnovirus segment G2

VIRL_781 Campoletis sonorensis ichnovirus segment H

VIRL_780 Campoletis sonorensis ichnovirus segment I

VIRL_777 Campoletis sonorensis ichnovirus segment I2

VIRL_779 Campoletis sonorensis ichnovirus segment J

VIRL_778 Campoletis sonorensis ichnovirus segment L

VIRL_776 Campoletis sonorensis ichnovirus segment M

VIRL_2555 Campoletis sonorensis ichnovirus segment N

VIRL_1952 Campoletis sonorensis ichnovirus segment O1

VIRL_1951 Campoletis sonorensis ichnovirus segment P

VIRL_593 Campoletis sonorensis ichnovirus segment Q

VIRL_1950 Campoletis sonorensis ichnovirus segment T

VIRL_1949 Campoletis sonorensis ichnovirus segment U

VIRL_1154 Campoletis sonorensis ichnovirus segment V

VIRL_774 Campoletis sonorensis ichnovirus segment Z

VIRL_772 Campoletis sonorensis ichnovirus superhelical segment A

VIRL_775 Campoletis sonorensis ichnovirus superhelical segment Aprime

VIRL_3292 Canary circovirus

VIRL_645 Canarypox virus

VIRL_3425 Canine adenovirus

VIRL_3312 Canine adenovirus 1

VIRL_3301 Canine adenovirus type 2

VIRL_2141 Canine calicivirus

VIRL_1258 Canine distemper virus

VIRL_914 Canine minute virus

VIRL_710 Canine oral papillomavirus

VIRL_635 Canine papillomavirus 2

VIRL_581 Canine papillomavirus 3

VIRL_543 Canine papillomavirus 4

VIRL_470 Canine papillomavirus 5

VIRL_469 Canine papillomavirus 6

VIRL_600 Canine papillomavirus 7

VIRL_1281 Canine parvovirus

VIRL_1337 Canna Yellow Streak Virus

VIRL_591 Capra hircus papillomavirus type 1

VIRL_3469 Caprine arthritis-encephalitis virus

VIRL_1914 Capsicum chlorosis virus segment L

VIRL_2849 Capsicum chlorosis virus segment M

VIRL_2850 Capsicum chlorosis virus segment S

VIRL_220 Cardamine chlorotic fleck virus

VIRL_1656 Cardiospermum yellow leaf curl virus satellite DNA beta

VIRL_514 Caretta caretta papillomavirus 1

VIRL_3267 Carnation etched ring virus

VIRL_2303 Carnation Italian ringspot virus

VIRL_2455 Carnation mottle virus

VIRL_2293 Carnation ringspot virus RNA 1

VIRL_2292 Carnation ringspot virus RNA 2

VIRL_3614 Carrot mottle mimic virus

VIRL_1215 Carrot red leaf luteovirus associated RNA

VIRL_1108 Carrot red leaf virus

VIRL_108 Carrot yellow leaf virus

VIRL_1483 Casphalia extranea densovirus

VIRL_965 Cassava brown streak virus

VIRL_2378 Cassava common mosaic virus

VIRL_3435 Cassava vein mosaic virus

VIRL_928 Cassava virus C

VIRL_930 Cassava virus C

VIRL_929 Cassava virus C

VIRL_2040 Cassia yellow blotch virus RNA1

VIRL_2039 Cassia yellow blotch virus RNA2

VIRL_2038 Cassia yellow blotch virus RNA3

VIRL_2489 Cauliflower mosaic virus

VIRL_58 Caviid herpesvirus 2

VIRL_2382 Cell fusing agent virus

VIRL_2769 Ceratocystis polonica partitivirus

VIRL_2770 Ceratocystis polonica partitivirus segment 1

VIRL_1624 Ceratocystis resinifera partitivirus RNA 1

VIRL_1625 Ceratocystis resinifera partitivirus RNA 2

VIRL_342 Cercopithecine herpesvirus 2

VIRL_149 Cercopithecine herpesvirus 5

VIRL_377 Cercopithecine herpesvirus 9

VIRL_2350 Cereal yellow dwarf virus-RPS

VIRL_3026 Cereal yellow dwarf virus-RPV

VIRL_2290 Cereal yellow dwarf virus-RPV satellite RNA

VIRL_1333 Cestrum yellow leaf curling virus

VIRL_140 Chaetoceros salsugineum DNA virus

VIRL_1361 Chaetoceros socialis f. radians RNA virus segment 1

VIRL_206 Chalara elegans RNA Virus 1

VIRL_3652 Chapare virus segment L

VIRL_3653 Chapare virus segment S

VIRL_1582 Chayote mosaic virus

VIRL_2134 Chayote yellow mosaic virus

VIRL_513 Chelonia mydas papillomavirus 1

VIRL_388 Cherry green ring mottle virus

VIRL_1522 Cherry mottle leaf virus

VIRL_1251 Cherry necrotic rusty mottle virus

VIRL_2926 Cherry rasp leaf virus

VIRL_2925 Cherry rasp leaf virus RNA2

VIRL_2245 Cherry virus A

VIRL_2495 Chicken anemia virus

VIRL_2217 Chicken astrovirus

VIRL_865 Chickpea chlorotic dwarf Sudan virus

VIRL_840 Chickpea chlorotic dwarf virus

VIRL_1918 Chickpea chlorotic stunt virus

VIRL_2220 Chicory yellow mottle virus large satellite RNA

VIRL_278 Chicory yellow mottle virus satellite RNA

VIRL_2059 Chicory yellow mottle virus satellite RNA L1

VIRL_364 Chikungunya virus

VIRL_3012 Chilli leaf curl disease associated sequence virion

VIRL_1371 Chilli leaf curl Multan alphasatellite

VIRL_3043 Chilli leaf curl virus

VIRL_2703 Chilli veinal mottle virus

VIRL_274 Chiltepin yellow mosaic virus

VIRL_3358 Chinese wheat mosaic virus RNA1

VIRL_3359 Chinese wheat mosaic virus RNA2

VIRL_3166 Chino del tomate virus DNA A

VIRL_3165 Chino del tomate virus DNA B

VIRL_1344 Chlamydia phage 3

VIRL_603 Chlamydia phage 4

VIRL_2632 Chlamydia phage Chp1

VIRL_2453 Chlamydia phage Chp2

VIRL_383 Chlamydia phage CPAR39

VIRL_2467 Chlamydia phage PhiCPG1

VIRL_1402 Chloris striate mosaic virus

VIRL_1062 Choristoneura fumiferana DEF MNPV

VIRL_1469 Choristoneura fumiferana MNPV

VIRL_1941 Choristoneura occidentalis granulovirus

VIRL_1430 Chronic bee paralysis virus RNA 1

VIRL_1429 Chronic bee paralysis virus RNA 2

VIRL_2830 Chrysanthemum virus B

VIRL_972 Chrysodeixis chalcites nucleopolyhedrovirus

VIRL_340 Chum salmon reovirus CS segment 5

VIRL_339 Chum salmon reovirus CS segment 7

VIRL_2073 Chuzan virus segment 3

VIRL_2072 Chuzan virus segment 1

VIRL_2067 Chuzan virus segment 10

VIRL_2950 Chuzan virus segment 2

VIRL_2071 Chuzan virus segment 4

VIRL_2069 Chuzan virus segment 5

VIRL_2074 Chuzan virus segment 6

VIRL_2949 Chuzan virus segment 7

VIRL_2068 Chuzan virus segment 8

VIRL_2070 Chuzan virus segment 9

VIRL_822 Circovirus-like genome BBC-A

VIRL_815 Circovirus-like genome CB-A

VIRL_814 Circovirus-like genome CB-B

VIRL_820 Circovirus-like genome RW-A

VIRL_819 Circovirus-like genome RW-B

VIRL_818 Circovirus-like genome RW-C

VIRL_817 Circovirus-like genome RW-D

VIRL_816 Circovirus-like genome RW-E

VIRL_813 Circovirus-like genome SAR-A

VIRL_823 Circovirus-like genome SAR-B

VIRL_168 Circulifer tenellus virus 1

VIRL_3148 Citrus leaf blotch virus

VIRL_2286 Citrus leaf rugose virus RNA 1

VIRL_2287 Citrus leaf rugose virus RNA 2

VIRL_2288 Citrus leaf rugose virus RNA 3

VIRL_968 Citrus leprosis virus C RNA-1

VIRL_967 Citrus leprosis virus C RNA-2

VIRL_1105 Citrus psorosis virus RNA1

VIRL_1104 Citrus psorosis virus RNA2

VIRL_1103 Citrus psorosis virus RNA3

VIRL_1056 Citrus sudden death-associated virus

VIRL_3433 Citrus tristeza virus

VIRL_202 Citrus variegation virus chromosome RNA 3

VIRL_1708 Citrus variegation virus RNA1

VIRL_1707 Citrus variegation virus RNA2

VIRL_3297 Citrus yellow mosaic virus

VIRL_1383 Clanis bilineata nucleopolyhedrosis virus

VIRL_3349 Classical swine fever virus

VIRL_81 Clavibacter phage CMP1

VIRL_881 Clerodendron yellow mosaic virus

VIRL_837 Clerodendrum golden mosaic China virus DNA A

VIRL_836 Clerodendrum golden mosaic China virus DNA B

VIRL_9 Clerodendrum golden mosaic virus DNA-A

VIRL_8 Clerodendrum golden mosaic virus DNA-B

VIRL_3564 Clostridium phage 39-O

VIRL_2699 Clostridium phage c-st

VIRL_1953 Clostridium phage phi CD119

VIRL_2294 Clostridium phage phi3626

VIRL_1721 Clostridium phage phiC2

VIRL_3529 Clostridium phage phiCD27

VIRL_73 Clostridium phage phiCTP1

VIRL_337 Clostridium phage phiSM101

VIRL_2475 Clover yellow mosaic virus

VIRL_3259 Clover yellow vein virus

VIRL_1289 Cocksfoot mild mosaic virus

VIRL_2445 Cocksfoot mottle virus

VIRL_3203 Cocksfoot streak virus

VIRL_2396 Coconut foliar decay virus

VIRL_1680 Coleus vein necrosis virus

VIRL_2158 Colorado tick fever virus segment 1

VIRL_3621 Colorado tick fever virus segment 10

VIRL_3619 Colorado tick fever virus segment 11

VIRL_3620 Colorado tick fever virus segment 12

VIRL_3628 Colorado tick fever virus segment 2

VIRL_3627 Colorado tick fever virus segment 3

VIRL_3626 Colorado tick fever virus segment 4

VIRL_3625 Colorado tick fever virus segment 5

VIRL_3624 Colorado tick fever virus segment 6

VIRL_3623 Colorado tick fever virus segment 7

VIRL_3622 Colorado tick fever virus segment 8

VIRL_2159 Colorado tick fever virus segment 9

VIRL_3357 Columbid circovirus

VIRL_2401 Commelina yellow mottle virus

VIRL_699 Common chimpanzee papillomavirus 1

VIRL_1159 Coniothyrium minitans RNA virus

VIRL_870 Corchorus golden mosaic virus DNA-A

VIRL_10 Corchorus golden mosaic virus DNA-B

VIRL_1557 Corchorus yellow spot virus DNA A

VIRL_1556 Corchorus yellow spot virus DNA B

VIRL_903 Corchorus yellow vein virus - [Hoa Binh] DNA A

VIRL_902 Corchorus yellow vein virus - [Hoa Binh] DNA B

VIRL_3663 Corynebacterium phage BFK20

VIRL_3585 Corynebacterium phage P1201

VIRL_40 Cote d'Ivoire ebolavirus

VIRL_634 Cotesia congregata bracovirus segment Circle1

VIRL_632 Cotesia congregata bracovirus segment Circle10

VIRL_631 Cotesia congregata bracovirus segment Circle11

VIRL_630 Cotesia congregata bracovirus segment Circle12

VIRL_3541 Cotesia congregata bracovirus segment Circle13

VIRL_629 Cotesia congregata bracovirus segment Circle14

VIRL_2049 Cotesia congregata bracovirus segment Circle15

VIRL_628 Cotesia congregata bracovirus segment Circle17

VIRL_3546 Cotesia congregata bracovirus segment Circle19

VIRL_3543 Cotesia congregata bracovirus segment Circle2

VIRL_2048 Cotesia congregata bracovirus segment Circle20

VIRL_2047 Cotesia congregata bracovirus segment Circle21

VIRL_3540 Cotesia congregata bracovirus segment Circle22

VIRL_3539 Cotesia congregata bracovirus segment Circle23

VIRL_626 Cotesia congregata bracovirus segment Circle25

VIRL_625 Cotesia congregata bracovirus segment Circle26

VIRL_3542 Cotesia congregata bracovirus segment Circle3

VIRL_2046 Cotesia congregata bracovirus segment Circle30

VIRL_3531 Cotesia congregata bracovirus segment Circle31

VIRL_3538 Cotesia congregata bracovirus segment Circle32

VIRL_2045 Cotesia congregata bracovirus segment Circle33

VIRL_624 Cotesia congregata bracovirus segment Circle35

VIRL_3537 Cotesia congregata bracovirus segment Circle36

VIRL_3532 Cotesia congregata bracovirus segment Circle4

VIRL_3547 Cotesia congregata bracovirus segment Circle5

VIRL_2050 Cotesia congregata bracovirus segment Circle9

VIRL_627 Cotesia congregata virus segment Circle18

VIRL_2052 Cotesia congregata virus segment Circle6

VIRL_633 Cotesia congregata virus segment Circle7

VIRL_2051 Cotesia congregata virus segment Circle8

VIRL_3054 Cotton leaf crumple geminivirus DNA B

VIRL_3055 Cotton leaf crumple virus DNA A

VIRL_3053 Cotton leaf curl Alabad virus

VIRL_898 Cotton leaf curl Bangalore virus segment A

VIRL_1083 Cotton leaf curl Bangalore virus-associated DNA beta

VIRL_801 Cotton leaf curl Burewala alphasatellite

VIRL_796 Cotton leaf curl Burewala betasatellite

VIRL_793 Cotton leaf curl Burewala virus - [India

VIRL_440 Cotton leaf curl Gezira alphasatellite

VIRL_2810 Cotton leaf curl Gezira beta

VIRL_439 Cotton leaf curl Gezira betasatellite

VIRL_38 Cotton leaf curl Gezira betasatellite extrachromosomal

VIRL_3356 Cotton leaf curl Gezira virus

VIRL_3052 Cotton leaf curl Kokhran virus

VIRL_3051 Cotton leaf curl Multan virus

VIRL_2816 Cotton leaf curl Multan virus satellite DNA beta

VIRL_1960 Cotton leaf curl Multan virus satellite U36-1

VIRL_918 Cotton leaf curl Rajasthan virus segment A

VIRL_1328 Cotton leaf curl virus-associated DNA beta

VIRL_722 Cottontail rabbit papillomavirus

VIRL_2179 Cowpea aphid-borne mosaic virus

VIRL_3256 Cowpea chlorotic mottle virus RNA 1

VIRL_3258 Cowpea chlorotic mottle virus RNA 2

VIRL_3257 Cowpea chlorotic mottle virus RNA 3

VIRL_2285 Cowpea mosaic virus RNA 1

VIRL_2284 Cowpea mosaic virus RNA 2

VIRL_2433 Cowpea mottle virus

VIRL_1088 Cowpea severe leaf curl-associated DNA beta

VIRL_2431 Cowpea severe mosaic virus RNA 1

VIRL_2432 Cowpea severe mosaic virus RNA 2

VIRL_673 Cowpox virus

VIRL_886 Crassocephalum yellow vein virus - Jinghong

VIRL_1211 Cricket paralysis virus

VIRL_2975 Crimean-Congo hemorrhagic fever virus segment L

VIRL_2976 Crimean-Congo hemorrhagic fever virus segment M

VIRL_2974 Crimean-Congo hemorrhagic fever virus segment S

VIRL_592 Crocodilepox virus

VIRL_802 Croton yellow vein mosaic alphasatellite

VIRL_3069 Croton yellow vein mosaic virus

VIRL_2841 Croton yellow vein mosaic virus satellite DNA beta

VIRL_48 Croton yellow vein virus

VIRL_595 Crow polyomavirus

VIRL_3300 Crucifer tobamovirus

VIRL_1283 Cryphonectria hypovirus 1

VIRL_2289 Cryphonectria hypovirus 2

VIRL_1252 Cryphonectria hypovirus 3

VIRL_1102 Cryphonectria hypovirus 4 endogenous virus

VIRL_1206 Cryphonectria parasitica mitovirus 1-NB631

VIRL_1140 Cryptophlebia leucotreta granulovirus

VIRL_1188 Cucumber Bulgarian latent virus

VIRL_1246 Cucumber fruit mottle mosaic virus

VIRL_3419 Cucumber green mottle mosaic virus

VIRL_2867 Cucumber leaf spot virus

VIRL_3387 Cucumber mosaic virus RNA 1

VIRL_260 Cucumber mosaic virus RNA 2

VIRL_3475 Cucumber mosaic virus RNA 3

VIRL_3351 Cucumber mosaic virus satellite RNA

VIRL_1890 Cucumber mottle virus

VIRL_2395 Cucumber necrosis virus

VIRL_1089 Cucumber vein yellowing virus

VIRL_3220 Cucurbit aphid-borne yellows virus

VIRL_92 Cucurbit leaf crumple virus DNA A

VIRL_91 Cucurbit leaf crumple virus DNA B

VIRL_3022 Cucurbit yellow stunting dis virus RNA1

VIRL_1144 Cucurbit yellow stunting dis virus RNA2

VIRL_2079 Cucurbita yellow vein virus-associated DNA beta

VIRL_1393 Culex flavivirus

VIRL_1241 Culex nigripalpus NPV

VIRL_1417 Culex pipiens densovirus

VIRL_1658 Cupixi virus segment L

VIRL_3595 Cupixi virus segment S

VIRL_2649 Curvularia thermal tolerance virus RNA1

VIRL_2648 Curvularia thermal tolerance virus RNA2

VIRL_1295 Cyanophage PSS2

VIRL_2540 Cyanophage Syn5

VIRL_3602 Cycad leaf necrosis virus

VIRL_1573 Cycas necrotic stunt virus RNA 1

VIRL_1572 Cycas necrotic stunt virus RNA 2

VIRL_376 Cydia pomonella granulovirus

VIRL_3417 Cymbidium mosaic virus

VIRL_2291 Cymbidium ringspot virus

VIRL_2182 Cymbidium ringspot virus satellite RNA

VIRL_3339 Cypovirus 14 RNA 1

VIRL_3330 Cypovirus 14 RNA 10

VIRL_3338 Cypovirus 14 RNA 2

VIRL_3337 Cypovirus 14 RNA 3

VIRL_3336 Cypovirus 14 RNA 4

VIRL_3335 Cypovirus 14 RNA 5

VIRL_3334 Cypovirus 14 RNA 6

VIRL_3333 Cypovirus 14 RNA 7

VIRL_3332 Cypovirus 14 RNA 8

VIRL_3331 Cypovirus 14 RNA 9

VIRL_566 Cyprinid herpesvirus 3

VIRL_3498 Daphne mosaic virus

VIRL_1947 Daphne virus S

VIRL_1501 Dasheen mosaic virus

VIRL_727 Deer papillomavirus

VIRL_619 Deerpox virus W-1170-84

VIRL_620 Deerpox virus W-848-83

VIRL_3020 Deformed wing virus

VIRL_937 Deftia phage phiW-14

VIRL_225 Dendrolimus punctatus densovirus

VIRL_1123 Dendrolimus punctatus tetravirus RNA1

VIRL_1122 Dendrolimus punctatus tetravirus RNA2

VIRL_3466 Dengue virus type 1

VIRL_2721 Dengue virus type 2

VIRL_3467 Dengue virus type 3

VIRL_3350 Dengue virus type 4

VIRL_1555 Desmodium leaf distortion virus DNA A

VIRL_1904 Desmodium leaf distortion virus DNA B

VIRL_3563 Diadromus pulchellus ascovirus 4a

VIRL_2353 Diaporthe ambigua RNA virus 1

VIRL_1602 Diascia yellow mottle virus

VIRL_1260 Diatraea saccharalis densovirus

VIRL_3153 Dicliptera yellow mottle virus DNA A

VIRL_2197 Dicliptera yellow mottle virus DNA B

VIRL_2394 Digitaria streak virus

VIRL_2835 Dioscorea bacilliform virus

VIRL_956 Diplodia scrobiculata RNA virus 1

VIRL_1513 Discula destructiva virus 1 RNA 4

VIRL_1243 Discula destructiva virus 1 segment 1

VIRL_1242 Discula destructiva virus 1 segment 2

VIRL_1514 Discula destructiva virus 1 segment 3

VIRL_1219 Discula destructiva virus 2 segment 1

VIRL_1218 Discula destructiva virus 2 segment 2

VIRL_2986 Dobrava virus segment M

VIRL_2987 Dobrava virus segment S

VIRL_2985 Dobrava-Belgrade virus strain DOBV/Ano-Poroia/Afl9/1999

VIRL_89 Dolichos yellow mosaic virus

VIRL_2089 Dolphin morbillivirus

VIRL_1558 Dracaena mottle virus

VIRL_790 Drosophila A virus

VIRL_390 Drosophila C virus

VIRL_1366 Drosophila melanogaster sigma virus AP30

VIRL_809 Drosophila melanogaster totivirus SW-2009a

VIRL_3629 Drosophila x virus segment A

VIRL_3631 Drosophila x virus segment B

VIRL_2372 Duck adenovirus A

VIRL_1356 Duck adenovirus A

VIRL_2674 Duck astrovirus C-NGB

VIRL_899 Duck circovirus

VIRL_476 Duck enteritis virus

VIRL_984 Duck hepatitis A virus

VIRL_3492 Duck hepatitis B virus

VIRL_2809 Duck hepatitis virus AP

VIRL_2160 Dugbe virus segment L

VIRL_2161 Dugbe virus segment M

VIRL_3113 Dugbe virus segment S

VIRL_970 Dulcamara mottle virus

VIRL_3046 East African cassava mosaic Cameroon virus DNA A

VIRL_3042 East African cassava mosaic Cameroon virus DNA B

VIRL_2745 East African cassava mosaic Kenya virus DNA A

VIRL_2744 East African cassava mosaic Kenya virus DNA B

VIRL_3030 East African cassava mosaic virus DNA A

VIRL_3029 East African cassava mosaic virus DNA B

VIRL_3033 East African cassava mosaic Zanzibar virus DNA B

VIRL_912 East African cassava mosaic Zanzibar virus DNA-A

VIRL_1958 East Asian Passiflora virus

VIRL_3140 Eastern equine encephalitis virus

VIRL_1892 Ecotropis obliqua NPV

VIRL_2441 Ectocarpus siliculosus virus 1

VIRL_663 Ectromelia virus

VIRL_750 Ectropis obliqua picorna-like virus

VIRL_2393 Eggplant mosaic virus

VIRL_1244 Eimeria brunetti RNA virus 1

VIRL_2281 Elm mottle virus RNA 1

VIRL_2282 Elm mottle virus RNA 2

VIRL_3242 Elm mottle virus RNA 3

VIRL_863 Emilia yellow vein virus-[Fz1]

VIRL_1418 Emilia yellow vein virus-associated DNA beta

VIRL_3545 Emiliania huxleyi virus 86

VIRL_222 Encephalomyocarditis virus

VIRL_2838 Entebbe bat virus

VIRL_2511 Enterobacteria phage 13a

VIRL_2629 Enterobacteria phage 933W

VIRL_2640 Enterobacteria phage alpha3

VIRL_2515 Enterobacteria phage BA14

VIRL_2594 Enterobacteria phage BP-4795

VIRL_2513 Enterobacteria phage EcoDS1

VIRL_414 Enterobacteria phage EPS7

VIRL_2595 Enterobacteria phage epsilon15

VIRL_1448 Enterobacteria phage ES18

VIRL_2586 Enterobacteria phage Felix 01

VIRL_2521 Enterobacteria phage Fels-2

VIRL_2604 Enterobacteria phage FI

VIRL_2637 Enterobacteria phage G4

VIRL_2634 Enterobacteria phage GA

VIRL_1528 Enterobacteria phage HK022

VIRL_1515 Enterobacteria phage HK620

VIRL_1527 Enterobacteria phage HK97

VIRL_743 Enterobacteria phage I2-2

VIRL_2557 Enterobacteria phage ID18

VIRL_2559 Enterobacteria phage ID2 Moscow/ID/2001

VIRL_697 Enterobacteria phage If1

VIRL_736 Enterobacteria phage Ike

VIRL_198 Enterobacteria phage IME08

VIRL_2018 Enterobacteria phage JK06

VIRL_488 Enterobacteria phage JS10

VIRL_551 Enterobacteria phage JS98

VIRL_489 Enterobacteria phage JSE

VIRL_2554 Enterobacteria phage K1-5

VIRL_2562 Enterobacteria phage K1E

VIRL_2563 Enterobacteria phage K1F

VIRL_2496 Enterobacteria phage lambda

VIRL_2619 Enterobacteria phage M13

VIRL_460 Enterobacteria phage Min27

VIRL_1530 Enterobacteria phage Mu

VIRL_1533 Enterobacteria phage N15

VIRL_2542 Enterobacteria phage N4

VIRL_2960 Enterobacteria phage P1

VIRL_698 Enterobacteria phage P2

VIRL_2625 Enterobacteria phage P22 virus

VIRL_2481 Enterobacteria phage P4

VIRL_558 Enterobacteria phage Phi1

VIRL_3616 Enterobacteria phage phiEco32

VIRL_3661 Enterobacteria phage phiEcoM-GJ1

VIRL_2314 Enterobacteria phage phiP27

VIRL_2635 Enterobacteria phage phiX174

VIRL_2636 Enterobacteria phage PRD1

VIRL_642 Enterobacteria phage PsP3

VIRL_2630 Enterobacteria phage Qbeta

VIRL_495 Enterobacteria phage RB14

VIRL_65 Enterobacteria phage RB16

VIRL_579 Enterobacteria phage RB32

VIRL_616 Enterobacteria phage RB43

VIRL_651 Enterobacteria phage RB49

VIRL_496 Enterobacteria phage RB51

VIRL_652 Enterobacteria phage RB69

VIRL_1977 Enterobacteria phage RTP

VIRL_1462 Enterobacteria phage Sf6

VIRL_2612 Enterobacteria phage SfV

VIRL_2592 Enterobacteria phage SP6

VIRL_2676 Enterobacteria phage SSL-2009a

VIRL_478 Enterobacteria phage St-1

VIRL_2581 Enterobacteria phage ST104

VIRL_1481 Enterobacteria phage ST64T

VIRL_1458 Enterobacteria phage T1

VIRL_2618 Enterobacteria phage T3

VIRL_693 Enterobacteria phage T4

VIRL_2959 Enterobacteria phage T5

VIRL_713 Enterobacteria phage T7

VIRL_2815 Enterobacteria phage TLS

VIRL_2458 Enterobacteria phage VT2-Sakai

VIRL_2558 Enterobacteria phage WA13

VIRL_481 Enterobacteria phage WV8

VIRL_517 Enterobacteria phage YYZ-2008

VIRL_2638 Enterobacterio phage MS2

VIRL_2675 Enterococcus phage EFAP-1

VIRL_938 Enterococcus phage phiEf11

VIRL_1432 Enterococcus phage phiEF24C

VIRL_798 Enterococcus phage phiFL1A

VIRL_800 Enterococcus phage phiFL2A

VIRL_797 Enterococcus phage phiFL3A

VIRL_799 Enterococcus phage phiFL4A

VIRL_1143 Enzootic nasal tumour virus of goats

VIRL_3118 Epinephelus tauvina nervous necrosis virus RNA 1

VIRL_3119 Epinephelus tauvina nervous necrosis virus RNA 2

VIRL_761 Epiphyas postvittana NPV

VIRL_936 Epirus cherry virus

VIRL_934 Epirus cherry virus

VIRL_935 Epirus cherry virus

VIRL_190 Epizootic hemorrhagic disease virus (serotype 1 / strain New Jersey) segment 1

VIRL_181 Epizootic hemorrhagic disease virus (serotype 1 / strain New Jersey) segment 10

VIRL_189 Epizootic hemorrhagic disease virus (serotype 1 / strain New Jersey) segment 2

VIRL_188 Epizootic hemorrhagic disease virus (serotype 1 / strain New Jersey) segment 3

VIRL_187 Epizootic hemorrhagic disease virus (serotype 1 / strain New Jersey) segment 4

VIRL_186 Epizootic hemorrhagic disease virus (serotype 1 / strain New Jersey) segment 5

VIRL_185 Epizootic hemorrhagic disease virus (serotype 1 / strain New Jersey) segment 6

VIRL_184 Epizootic hemorrhagic disease virus (serotype 1 / strain New Jersey) segment 7

VIRL_183 Epizootic hemorrhagic disease virus (serotype 1 / strain New Jersey) segment 8

VIRL_182 Epizootic hemorrhagic disease virus (serotype 1 / strain New Jersey) segment 9

VIRL_395 Equid herpesvirus 1

VIRL_709 Equid herpesvirus 2

VIRL_389 Equid herpesvirus 4

VIRL_334 Equid herpesvirus 9

VIRL_3355 Equine arteritis virus

VIRL_1654 Equine coronavirus

VIRL_2349 Equine foamy virus

VIRL_765 Equine infectious anemia virus

VIRL_499 Equine papillomavirus 2

VIRL_3138 Equine rhinitis A virus

VIRL_2186 Equine rhinitis B virus 1

VIRL_373 Equine rhinitis B virus 2

VIRL_662 Equinus papillomavirus

VIRL_672 Equus caballus papillomavirus - 1

VIRL_825 Eragrostis curvula streak virus

VIRL_862 Eragrostis streak virus

VIRL_875 Erectites yellow mosaic virus DNA-A

VIRL_1700 Erectites yellow mosaic virus satellite DNA beta

VIRL_223 Erethizon dorsatum papillomavirus type 1

VIRL_505 Erinaceus europaeus papillomavirus

VIRL_2541 Erwinia amylovora phage Era103

VIRL_503 Erwinia phage phiEa21-4

VIRL_386 Erysimum latent virus

VIRL_197 Escherichia phage D108

VIRL_598 Escherichia phage phiV10

VIRL_2514 Escherichia phage rv5

VIRL_1629 Eupatorium vein clearing virus

VIRL_3254 Eupatorium yellow vein virus

VIRL_3060 Eupatorium yellow vein virus-associated DNA beta

VIRL_2972 Euphorbia leaf curl virus DNA A

VIRL_2848 Euphorbia mosaic virus-Yucatan Peninsula DNA A

VIRL_2847 Euphorbia mosaic virus-Yucatan Peninsula DNA B

VIRL_2644 Euphorbia yellow mosaic virus DNA A

VIRL_2643 Euphorbia yellow mosaic virus DNA B

VIRL_1538 Euproctis pseudoconspersa nucleopolyhedrovirus

VIRL_1234 Euprosterna elaeasa virus

VIRL_2818 European bat lyssavirus 1

VIRL_2817 European bat lyssavirus 2

VIRL_1517 European brown hare syndrome virus

VIRL_726 European elk papillomavirus

VIRL_1010 European mountain ash ringspot-associated virus RNA 1

VIRL_1006 European mountain ash ringspot-associated virus RNA 2

VIRL_1004 European mountain ash ringspot-associated virus RNA 3

VIRL_1005 European mountain ash ringspot-associated virus RNA 4

VIRL_1221 Eyach virus segment 1

VIRL_2235 Eyach virus segment 10

VIRL_2234 Eyach virus segment 11

VIRL_2233 Eyach virus segment 12

VIRL_2242 Eyach virus segment 2

VIRL_2241 Eyach virus segment 3

VIRL_2240 Eyach virus segment 4

VIRL_2239 Eyach virus segment 5

VIRL_2238 Eyach virus segment 6

VIRL_2237 Eyach virus segment 7

VIRL_2236 Eyach virus segment 8

VIRL_1220 Eyach virus segment 9

VIRL_1378 Faba bean necrotic stunt virus DNA C

VIRL_1377 Faba bean necrotic stunt virus DNA M

VIRL_1376 Faba bean necrotic stunt virus DNA N

VIRL_1380 Faba bean necrotic stunt virus DNA R

VIRL_1379 Faba bean necrotic stunt virus DNA S

VIRL_1375 Faba bean necrotic stunt virus DNA U1

VIRL_1374 Faba bean necrotic stunt virus DNA U2

VIRL_1373 Faba bean necrotic stunt virus DNA U4

VIRL_3249 Faba bean necrotic yellows virus

VIRL_3246 Faba bean necrotic yellows virus

VIRL_3252 Faba bean necrotic yellows virus DNA 1

VIRL_3251 Faba bean necrotic yellows virus DNA 10

VIRL_3250 Faba bean necrotic yellows virus DNA 2

VIRL_3248 Faba bean necrotic yellows virus DNA 4

VIRL_3247 Faba bean necrotic yellows virus DNA 5

VIRL_3245 Faba bean necrotic yellows virus DNA 7

VIRL_3244 Faba bean necrotic yellows virus DNA 8

VIRL_3243 Faba bean necrotic yellows virus DNA 9

VIRL_1288 Feldmannia species virus

VIRL_1000 Felid herpesvirus 1

VIRL_3465 Feline calicivirus

VIRL_1054 Feline coronavirus

VIRL_123 Feline coronavirus RM

VIRL_112 Feline coronavirus UU10

VIRL_115 Feline coronavirus UU11

VIRL_114 Feline coronavirus UU15

VIRL_113 Feline coronavirus UU16

VIRL_120 Feline coronavirus UU2

VIRL_806 Feline coronavirus UU22

VIRL_805 Feline coronavirus UU23

VIRL_119 Feline coronavirus UU3

VIRL_122 Feline coronavirus UU4

VIRL_121 Feline coronavirus UU5

VIRL_94 Feline coronavirus UU7

VIRL_93 Feline coronavirus UU8

VIRL_111 Feline coronavirus UU9

VIRL_2741 Feline foamy virus

VIRL_1401 Feline immunodeficiency virus

VIRL_3392 Feline leukemia virus

VIRL_1187 Felis domesticus papillomavirus type 1

VIRL_170 Fenneropenaeus chinensis hepatopancreatic densovirus

VIRL_2099 Fer-de-lance virus

VIRL_2283 Figwort mosaic virus

VIRL_1165 Fiji disease virus chromosome segment 4

VIRL_1162 Fiji disease virus segment 1

VIRL_1160 Fiji disease virus segment 10

VIRL_1166 Fiji disease virus segment 2

VIRL_1163 Fiji disease virus segment 3

VIRL_1161 Fiji disease virus segment 5

VIRL_1164 Fiji disease virus segment 6

VIRL_1025 Fiji disease virus segment 7

VIRL_971 Fiji disease virus segment 8

VIRL_2025 Fiji disease virus segment 9

VIRL_3667 Finch circovirus

VIRL_594 Finch polyomavirus

VIRL_14 Flavobacterium phage 11b

VIRL_3594 Flexal virus segment L

VIRL_2764 Flexal virus segment S

VIRL_3116 Flock house virus RNA 1

VIRL_3117 Flock house virus RNA 2

VIRL_1127 Foot-and-mouth disease virus - type A

VIRL_1185 Foot-and-mouth disease virus - type Asia 1

VIRL_1250 Foot-and-mouth disease virus - type C

VIRL_1209 Foot-and-mouth disease virus - type O

VIRL_1126 Foot-and-mouth disease virus - type SAT 1

VIRL_1145 Foot-and-mouth disease virus - type SAT 2

VIRL_1061 Foot-and-mouth disease virus - type SAT 3

VIRL_1026 Fort Morgan virus

VIRL_1351 Fowl adenovirus A

VIRL_2477 Fowl adenovirus A

VIRL_2459 Fowl adenovirus D

VIRL_1352 Fowl adenovirus D

VIRL_688 Fowlpox virus

VIRL_3464 Foxtail mosaic virus

VIRL_982 Fragaria chiloensis cryptic virus RNA 1

VIRL_3658 Fragaria chiloensis cryptic virus RNA 2

VIRL_3659 Fragaria chiloensis cryptic virus RNA 3

VIRL_1096 Fragaria chiloensis latent virus RNA 1

VIRL_1095 Fragaria chiloensis latent virus RNA 2

VIRL_2054 Fragaria chiloensis latent virus RNA 3

VIRL_472 Francolinus leucoscepus papillomavirus 1

VIRL_237 Freesia mosaic virus

VIRL_3488 Friend murine leukemia virus

VIRL_667 Fringilla coelebs papillomavirus

VIRL_1614 Fritillary virus Y

VIRL_329 Frog adenovirus 1

VIRL_2954 Frog virus 3

VIRL_1286 Fujinami sarcoma virus

VIRL_1386 Fusarium graminearum dsRNA mycovirus-1

VIRL_943 Fusarium graminearum dsRNA mycovirus-3

VIRL_942 Fusarium graminearum dsRNA mycovirus-4 dsRNA1

VIRL_941 Fusarium graminearum dsRNA mycovirus-4 dsRNA2

VIRL_792 Fusarium poae virus 1 RNA 1

VIRL_791 Fusarium poae virus 1 RNA 2

VIRL_2371 Galinsoga mosaic virus

VIRL_2423 Galleria mellonella densovirus

VIRL_341 Gallid herpesvirus 1

VIRL_382 Gallid herpesvirus 2

VIRL_379 Gallid herpesvirus 3

VIRL_53 Gammapapillomavirus HPV127

VIRL_3253 Garlic latent virus

VIRL_2725 Garlic virus A

VIRL_2724 Garlic virus C

VIRL_2180 Garlic virus E

VIRL_3420 Garlic virus X

VIRL_1589 Gayfeather mild mottle virus RNA 1

VIRL_1588 Gayfeather mild mottle virus RNA 2

VIRL_1587 Gayfeather mild mottle virus RNA 3

VIRL_3429 GB virus C/Hepatitis G virus

VIRL_1382 Geobacillus phage GBSV1

VIRL_3590 Geobacillus virus E2

VIRL_1097 Getah virus

VIRL_3255 Giardia lamblia virus

VIRL_3408 Gibbon ape leukemia virus

VIRL_756 Gill-associated virus

VIRL_1649 Glossina pallidipes salivary gland hypertrophy virus

VIRL_1871 Glypta fumiferanae ichnovirus segment A1

VIRL_1862 Glypta fumiferanae ichnovirus segment A10

VIRL_1870 Glypta fumiferanae ichnovirus segment A2

VIRL_1869 Glypta fumiferanae ichnovirus segment A3

VIRL_1868 Glypta fumiferanae ichnovirus segment A4

VIRL_1867 Glypta fumiferanae ichnovirus segment A5

VIRL_1866 Glypta fumiferanae ichnovirus segment A6

VIRL_1865 Glypta fumiferanae ichnovirus segment A7

VIRL_1864 Glypta fumiferanae ichnovirus segment A8

VIRL_1863 Glypta fumiferanae ichnovirus segment A9

VIRL_1861 Glypta fumiferanae ichnovirus segment B1

VIRL_1853 Glypta fumiferanae ichnovirus segment B10

VIRL_1852 Glypta fumiferanae ichnovirus segment B11

VIRL_1851 Glypta fumiferanae ichnovirus segment B12

VIRL_1850 Glypta fumiferanae ichnovirus segment B13

VIRL_1849 Glypta fumiferanae ichnovirus segment B14

VIRL_1848 Glypta fumiferanae ichnovirus segment B15

VIRL_1847 Glypta fumiferanae ichnovirus segment B16

VIRL_1846 Glypta fumiferanae ichnovirus segment B17

VIRL_1776 Glypta fumiferanae ichnovirus segment B18

VIRL_1845 Glypta fumiferanae ichnovirus segment B19

VIRL_1777 Glypta fumiferanae ichnovirus segment B2

VIRL_1844 Glypta fumiferanae ichnovirus segment B20

VIRL_1843 Glypta fumiferanae ichnovirus segment B21

VIRL_1842 Glypta fumiferanae ichnovirus segment B22

VIRL_1841 Glypta fumiferanae ichnovirus segment B23

VIRL_1840 Glypta fumiferanae ichnovirus segment B24

VIRL_1839 Glypta fumiferanae ichnovirus segment B25

VIRL_1838 Glypta fumiferanae ichnovirus segment B26

VIRL_1837 Glypta fumiferanae ichnovirus segment B27

VIRL_1836 Glypta fumiferanae ichnovirus segment B28

VIRL_1835 Glypta fumiferanae ichnovirus segment B29

VIRL_1860 Glypta fumiferanae ichnovirus segment B3

VIRL_1775 Glypta fumiferanae ichnovirus segment B30

VIRL_1834 Glypta fumiferanae ichnovirus segment B31

VIRL_1833 Glypta fumiferanae ichnovirus segment B32

VIRL_1832 Glypta fumiferanae ichnovirus segment B33

VIRL_1831 Glypta fumiferanae ichnovirus segment B34

VIRL_1830 Glypta fumiferanae ichnovirus segment B35

VIRL_1774 Glypta fumiferanae ichnovirus segment B36

VIRL_1829 Glypta fumiferanae ichnovirus segment B37

VIRL_1828 Glypta fumiferanae ichnovirus segment B38

VIRL_1827 Glypta fumiferanae ichnovirus segment B39

VIRL_1859 Glypta fumiferanae ichnovirus segment B4

VIRL_1826 Glypta fumiferanae ichnovirus segment B40

VIRL_1825 Glypta fumiferanae ichnovirus segment B41

VIRL_1824 Glypta fumiferanae ichnovirus segment B42

VIRL_1823 Glypta fumiferanae ichnovirus segment B43

VIRL_1822 Glypta fumiferanae ichnovirus segment B44

VIRL_1821 Glypta fumiferanae ichnovirus segment B45

VIRL_1820 Glypta fumiferanae ichnovirus segment B46

VIRL_1819 Glypta fumiferanae ichnovirus segment B47

VIRL_1773 Glypta fumiferanae ichnovirus segment B48

VIRL_1818 Glypta fumiferanae ichnovirus segment B49

VIRL_1858 Glypta fumiferanae ichnovirus segment B5

VIRL_1817 Glypta fumiferanae ichnovirus segment B50

VIRL_1816 Glypta fumiferanae ichnovirus segment B51

VIRL_1815 Glypta fumiferanae ichnovirus segment B52

VIRL_1814 Glypta fumiferanae ichnovirus segment B53

VIRL_1772 Glypta fumiferanae ichnovirus segment B54

VIRL_2836 Glypta fumiferanae ichnovirus segment B55

VIRL_1813 Glypta fumiferanae ichnovirus segment B56

VIRL_1812 Glypta fumiferanae ichnovirus segment B57

VIRL_1811 Glypta fumiferanae ichnovirus segment B58

VIRL_1810 Glypta fumiferanae ichnovirus segment B59

VIRL_1857 Glypta fumiferanae ichnovirus segment B6

VIRL_1809 Glypta fumiferanae ichnovirus segment B60

VIRL_1808 Glypta fumiferanae ichnovirus segment B61

VIRL_1807 Glypta fumiferanae ichnovirus segment B62

VIRL_1806 Glypta fumiferanae ichnovirus segment B63

VIRL_1805 Glypta fumiferanae ichnovirus segment B64

VIRL_1804 Glypta fumiferanae ichnovirus segment B65

VIRL_1856 Glypta fumiferanae ichnovirus segment B7

VIRL_1855 Glypta fumiferanae ichnovirus segment B8

VIRL_1854 Glypta fumiferanae ichnovirus segment B9

VIRL_1771 Glypta fumiferanae ichnovirus segment C1

VIRL_1797 Glypta fumiferanae ichnovirus segment C10

VIRL_1796 Glypta fumiferanae ichnovirus segment C11

VIRL_1795 Glypta fumiferanae ichnovirus segment C12

VIRL_1794 Glypta fumiferanae ichnovirus segment C13

VIRL_1793 Glypta fumiferanae ichnovirus segment C14

VIRL_1792 Glypta fumiferanae ichnovirus segment C15

VIRL_1791 Glypta fumiferanae ichnovirus segment C16

VIRL_1790 Glypta fumiferanae ichnovirus segment C17

VIRL_1789 Glypta fumiferanae ichnovirus segment C18

VIRL_1788 Glypta fumiferanae ichnovirus segment C19

VIRL_1803 Glypta fumiferanae ichnovirus segment C2

VIRL_1787 Glypta fumiferanae ichnovirus segment C20

VIRL_1786 Glypta fumiferanae ichnovirus segment C21

VIRL_1785 Glypta fumiferanae ichnovirus segment C22

VIRL_1802 Glypta fumiferanae ichnovirus segment C3

VIRL_1801 Glypta fumiferanae ichnovirus segment C4

VIRL_1800 Glypta fumiferanae ichnovirus segment C5

VIRL_1799 Glypta fumiferanae ichnovirus segment C6

VIRL_575 Glypta fumiferanae ichnovirus segment C7

VIRL_1770 Glypta fumiferanae ichnovirus segment C8

VIRL_1798 Glypta fumiferanae ichnovirus segment C9

VIRL_1784 Glypta fumiferanae ichnovirus segment D1

VIRL_1783 Glypta fumiferanae ichnovirus segment D2

VIRL_1782 Glypta fumiferanae ichnovirus segment D3

VIRL_1769 Glypta fumiferanae ichnovirus segment D4

VIRL_1781 Glypta fumiferanae ichnovirus segment D5

VIRL_1780 Glypta fumiferanae ichnovirus segment D6

VIRL_1779 Glypta fumiferanae ichnovirus segment D7

VIRL_1778 Glypta fumiferanae ichnovirus segment E1

VIRL_669 Goatpox virus Pellor

VIRL_3002 Golden shiner reovirus segment 1

VIRL_2993 Golden shiner reovirus segment 10

VIRL_2992 Golden shiner reovirus segment 11

VIRL_3001 Golden shiner reovirus segment 2

VIRL_3000 Golden shiner reovirus segment 3

VIRL_2999 Golden shiner reovirus segment 4

VIRL_2998 Golden shiner reovirus segment 5

VIRL_2997 Golden shiner reovirus segment 6

VIRL_2996 Golden shiner reovirus segment 7

VIRL_2995 Golden shiner reovirus segment 8

VIRL_2994 Golden shiner reovirus segment 9

VIRL_3317 Goose circovirus

VIRL_653 Goose hemorrhagic polyomavirus

VIRL_1184 Goose paramyxovirus SF02

VIRL_923 Goose parvovirus

VIRL_959 Gossypium darwinii symptomless alphasatellite DNA-alpha

VIRL_1596 Gossypium darwinii symptomless virus DNA-A

VIRL_961 Gossypium davidsonii symptomless alphasatellite DNA-alpha-B

VIRL_960 Gossypium mustilinum symptomless alphasatellite DNA-alpha-B

VIRL_830 Gossypium punctatum mild leaf curl virus DNA A

VIRL_1590 Gossypium punctatum mild leaf curl virus DNA B

VIRL_3521 Grapevine Algerian latent virus

VIRL_2275 Grapevine chrome mosaic virus RNA 1

VIRL_2276 Grapevine chrome mosaic virus RNA 2

VIRL_2277 Grapevine fanleaf virus RNA 1

VIRL_3232 Grapevine fanleaf virus RNA 2

VIRL_2318 Grapevine fanleaf virus satellite RNA

VIRL_2315 Grapevine fleck virus

VIRL_2730 Grapevine leafroll-associated virus 10

VIRL_1041 Grapevine leafroll-associated virus 2

VIRL_3031 Grapevine leafroll-associated virus 3

VIRL_2116 Grapevine rootstock stem lesion associated virus

VIRL_2671 Grapevine Syrah Virus-1

VIRL_3240 Grapevine virus A

VIRL_3241 Grapevine virus B

VIRL_3534 Grapevine virus E

VIRL_1109 Gremmeniella abietina mitochondrial RNA virus S2

VIRL_3632 Gremmeniella abietina RNA virus L1

VIRL_1120 Gremmeniella abietina RNA virus L2

VIRL_1490 Gremmeniella abietina RNA virus MS1 RNA 1

VIRL_1489 Gremmeniella abietina RNA virus MS1 RNA 2

VIRL_1488 Gremmeniella abietina RNA virus MS1 RNA 3

VIRL_1452 Gremmeniella abietina RNA virus MS2 RNA 1

VIRL_1451 Gremmeniella abietina RNA virus MS2 RNA 2

VIRL_1450 Gremmeniella abietina RNA virus MS2 RNA 3

VIRL_969 Gremmeniella abietina type B RNA virus XL1

VIRL_1285 Ground squirrel hepatitis virus

VIRL_2278 Groundnut bud necrosis virus segment L

VIRL_3233 Groundnut bud necrosis virus segment M

VIRL_3234 Groundnut bud necrosis virus segment S

VIRL_2280 Groundnut rosette virus

VIRL_3347 Groundnut rosette virus satellite RNA

VIRL_3670 Gryllus bimaculatus nudivirus

VIRL_1136 Guanarito virus segment L

VIRL_1182 Guanarito virus segment S

VIRL_1899 Gull circovirus

VIRL_2478 Haemophilus phage HP1

VIRL_2438 Haemophilus phage HP2

VIRL_924 Haloarcula hispanica pleomorphic virus 1

VIRL_2019 Haloarcula phage SH1

VIRL_2523 Halomonas phage phiHAP-1

VIRL_1314 Halorubrum phage HF2

VIRL_2641 Halorubrum pleomorphic virus 1

VIRL_2104 Halovirus HF1

VIRL_708 Hamster polyomavirus

VIRL_1177 Hantaan virus

VIRL_209 Hantaan virus

VIRL_2991 Hantaan virus segment L

VIRL_263 Hantavirus Z10 chromosome L

VIRL_264 Hantavirus Z10 chromosome S segment

VIRL_1171 Hantavirus Z10 segment M

VIRL_101 Helicobasidium mompa endornavirus 1

VIRL_2101 Helicobasidium mompa No.17 dsRNA virus segment L

VIRL_2100 Helicobasidium mompa No.17 dsRNA virus segment S

VIRL_1662 Helicoverpa armigera granulovirus

VIRL_3506 Helicoverpa armigera multiple nucleopolyhedrovirus

VIRL_1146 Helicoverpa armigera NPV

VIRL_795 Helicoverpa armigera NPV NNg1

VIRL_991 Helicoverpa armigera nucleopolyhedrovirus G4

VIRL_2360 Helicoverpa armigera stunt virus RNA 1

VIRL_2359 Helicoverpa armigera stunt virus RNA 2

VIRL_1508 Helicoverpa zea SNPV

VIRL_1634 Heliothis armigera cypovirus 5 segment 1

VIRL_2774 Heliothis armigera cypovirus 5 segment 10

VIRL_1635 Heliothis armigera cypovirus 5 segment 2

VIRL_1636 Heliothis armigera cypovirus 5 segment 3

VIRL_1637 Heliothis armigera cypovirus 5 segment 4

VIRL_1638 Heliothis armigera cypovirus 5 segment 5

VIRL_1639 Heliothis armigera cypovirus 5 segment 6

VIRL_1642 Heliothis armigera cypovirus 5 segment 7

VIRL_1640 Heliothis armigera cypovirus 5 segment 8

VIRL_1641 Heliothis armigera cypovirus 5 segment 9

VIRL_3587 Heliothis virescens ascovirus 3e

VIRL_1287 Heliothis zea virus 1

VIRL_2683 Helleborus net necrosis virus

VIRL_454 Helminthosporium victoriae 145S virus

VIRL_453 Helminthosporium victoriae 145S virus

VIRL_455 Helminthosporium victoriae 145S virus

VIRL_452 Helminthosporium victoriae 145S virus

VIRL_215 Helminthosporium victoriae virus 190S

VIRL_1259 Hendra virus

VIRL_3463 Hepatitis A virus

VIRL_2427 Hepatitis B virus

VIRL_1403 Hepatitis C virus genotype 1

VIRL_2807 Hepatitis C virus genotype 2

VIRL_2806 Hepatitis C virus genotype 3

VIRL_2805 Hepatitis C virus genotype 4

VIRL_2804 Hepatitis C virus genotype 5

VIRL_2803 Hepatitis C virus genotype 6

VIRL_2479 Hepatitis delta virus

VIRL_51 Hepatitis E virus

VIRL_3412 Hepatitis GB virus A

VIRL_3434 Hepatitis GB virus B

VIRL_2670 Heron hepatitis B virus

VIRL_2888 Heterocapsa circularisquama RNA virus

VIRL_2090 Heterosigma akashiwo RNA virus SOG263

VIRL_3238 Hibiscus chlorotic ringspot virus

VIRL_1031 Hibiscus latent Singapore virus

VIRL_1342 Highlands J virus

VIRL_3180 Himetobi P virus

VIRL_3526 Hippeastrum latent virus

VIRL_232 Hirame rhabdovirus

VIRL_597 His1 virus

VIRL_596 His2 virus

VIRL_102 HMO Astrovirus A

VIRL_3127 Hollyhock leaf crumple virus

VIRL_3124 Hollyhock leaf crumple virus satellite DNA

VIRL_3674 Homalodisca coagulata virus-1

VIRL_2665 Homalodisca vitripennis reovirus segment S1

VIRL_2656 Homalodisca vitripennis reovirus segment S10

VIRL_2655 Homalodisca vitripennis reovirus segment S11

VIRL_2654 Homalodisca vitripennis reovirus segment S12

VIRL_2664 Homalodisca vitripennis reovirus segment S2

VIRL_2663 Homalodisca vitripennis reovirus segment S3

VIRL_2662 Homalodisca vitripennis reovirus segment S4

VIRL_2661 Homalodisca vitripennis reovirus segment S5

VIRL_2660 Homalodisca vitripennis reovirus segment S6

VIRL_2659 Homalodisca vitripennis reovirus segment S7

VIRL_2658 Homalodisca vitripennis reovirus segment S8

VIRL_2657 Homalodisca vitripennis reovirus segment S9

VIRL_253 Honeysuckle yellow vein beta-[Japan:Fukui:2001]

VIRL_252 Honeysuckle yellow vein mosaic beta-[Japan:Miyizaki:2001]

VIRL_2813 Honeysuckle yellow vein mosaic disease associated satellite DNA beta-[Ibaraki]

VIRL_3237 Honeysuckle yellow vein mosaic virus

VIRL_3009 Honeysuckle yellow vein mosaic virus satellite DNA beta

VIRL_887 Honeysuckle yellow vein mosaic virus-[Kagoshima]

VIRL_2964 Honeysuckle yellow vein virus-[UK1]

VIRL_2342 Hop latent virus

VIRL_3601 Hop mosaic virus

VIRL_1453 Hordeum mosaic virus

VIRL_2085 Horsegram yellow mosaic virus

VIRL_1428 Horsegram yellow mosaic virus DNA B

VIRL_381 Horseradish curly top virus

VIRL_3523 Hosta virus X

VIRL_3310 Human adenovirus 2

VIRL_3309 Human adenovirus 5

VIRL_109 Human adenovirus 54

VIRL_2397 Human adenovirus A

VIRL_1355 Human adenovirus A

VIRL_2757 Human adenovirus B1

VIRL_2758 Human adenovirus B2

VIRL_1537 Human adenovirus C

VIRL_3311 Human adenovirus D

VIRL_2759 Human adenovirus D

VIRL_1510 Human adenovirus E

VIRL_1536 Human adenovirus F

VIRL_3304 Human adenovirus type 1

VIRL_3306 Human adenovirus type 11

VIRL_3302 Human adenovirus type 35

VIRL_3303 Human adenovirus type 7

VIRL_3391 Human astrovirus

VIRL_897 Human bocavirus

VIRL_2505 Human bocavirus 2

VIRL_2504 Human bocavirus 3

VIRL_15 Human bocavirus 4

VIRL_328 Human coronavirus 229E

VIRL_2916 Human coronavirus HKU1

VIRL_1129 Human coronavirus NL63

VIRL_1133 Human coronavirus OC43

VIRL_1406 Human cosavirus A1

VIRL_1405 Human cosavirus B1

VIRL_1404 Human cosavirus D1

VIRL_1407 Human cosavirus E1

VIRL_116 Human enteric coronavirus strain 4408

VIRL_2802 Human enterovirus 100

VIRL_441 Human enterovirus 107

VIRL_172 Human enterovirus 109

VIRL_473 Human enterovirus 98

VIRL_3442 Human enterovirus A

VIRL_3468 Human enterovirus B

VIRL_3480 Human enterovirus C

VIRL_3479 Human enterovirus D

VIRL_2151 Human erythrovirus V9

VIRL_64 Human herpesvirus 1

VIRL_392 Human herpesvirus 2

VIRL_396 Human herpesvirus 3

VIRL_564 Human herpesvirus 4

VIRL_602 Human herpesvirus 4 type 1

VIRL_348 Human herpesvirus 5

VIRL_394 Human herpesvirus 6A

VIRL_96 Human herpesvirus 6B

VIRL_393 Human herpesvirus 7

VIRL_336 Human herpesvirus 8

VIRL_3418 Human immunodeficiency virus 1

VIRL_2476 Human immunodeficiency virus 2

VIRL_950 Human klassevirus 1

VIRL_3114 Human metapneumovirus

VIRL_738 Human papillomavirus - 1

VIRL_737 Human papillomavirus - 18

VIRL_741 Human papillomavirus - 2

VIRL_707 Human papillomavirus 54

VIRL_519 Human papillomavirus FA75/KI88-03

VIRL_211 Human papillomavirus RTRX7

VIRL_1022 Human papillomavirus SIBX-3a

VIRL_721 Human papillomavirus type 10

VIRL_484 Human papillomavirus type 100

VIRL_588 Human papillomavirus type 101

VIRL_589 Human papillomavirus type 103

VIRL_480 Human papillomavirus type 104

VIRL_483 Human papillomavirus type 105

VIRL_498 Human papillomavirus type 108

VIRL_443 Human papillomavirus type 109

VIRL_442 Human papillomavirus type 112

VIRL_482 Human papillomavirus type 113

VIRL_437 Human papillomavirus type 114

VIRL_1007 Human papillomavirus type 115

VIRL_477 Human papillomavirus type 116

VIRL_257 Human papillomavirus type 121

VIRL_725 Human papillomavirus type 16

VIRL_705 Human papillomavirus type 24

VIRL_720 Human papillomavirus type 26

VIRL_719 Human papillomavirus type 32

VIRL_718 Human papillomavirus type 34

VIRL_733 Human papillomavirus type 4

VIRL_740 Human papillomavirus type 41

VIRL_704 Human papillomavirus type 48

VIRL_717 Human papillomavirus type 49

VIRL_724 Human papillomavirus type 5

VIRL_703 Human papillomavirus type 50

VIRL_716 Human papillomavirus type 53

VIRL_702 Human papillomavirus type 60

VIRL_1270 Human papillomavirus type 61

VIRL_732 Human papillomavirus type 63

VIRL_739 Human papillomavirus type 6b

VIRL_715 Human papillomavirus type 7

VIRL_684 Human papillomavirus type 71

VIRL_541 Human papillomavirus type 88

VIRL_714 Human papillomavirus type 9

VIRL_664 Human papillomavirus type 90

VIRL_658 Human papillomavirus type 92

VIRL_649 Human papillomavirus type 96

VIRL_486 Human papillomavirus type 98

VIRL_485 Human papillomavirus type 99

VIRL_1502 Human parainfluenza virus 1

VIRL_2437 Human parainfluenza virus 2

VIRL_2473 Human parainfluenza virus 3

VIRL_3406 Human parechovirus

VIRL_1086 Human parvovirus 4

VIRL_1255 Human parvovirus B19

VIRL_2034 Human picobirnavirus RNA segment 1

VIRL_2033 Human picobirnavirus RNA segment 2

VIRL_1268 Human respiratory syncytial virus

VIRL_1284 Human rhinovirus 14

VIRL_3440 Human rhinovirus 89

VIRL_2793 Human rhinovirus C

VIRL_753 Human T-lymphotropic virus 1

VIRL_2490 Human T-lymphotropic virus 2

VIRL_1597 Human T-lymphotropic virus 4

VIRL_3555 Human TMEV-like cardiovirus

VIRL_1111 Humulus japonicus latent virus

VIRL_1113 Humulus japonicus latent virus

VIRL_1112 Humulus japonicus latent virus

VIRL_927 Hydrangea chlorotic mottle virus

VIRL_3676 Hydrangea ringspot virus

VIRL_179 Hyperthermophilic Archaeal Virus 1

VIRL_180 Hyperthermophilic Archaeal Virus 2

VIRL_2723 Hyphantria cunea nucleopolyhedrovirus

VIRL_1768 Hyposoter fugitivus ichnovirus segment A1

VIRL_1764 Hyposoter fugitivus ichnovirus segment A2

VIRL_1763 Hyposoter fugitivus ichnovirus segment A3

VIRL_193 Hyposoter fugitivus ichnovirus segment B1

VIRL_570 Hyposoter fugitivus ichnovirus segment B10

VIRL_1767 Hyposoter fugitivus ichnovirus segment B11

VIRL_1760 Hyposoter fugitivus ichnovirus segment B12

VIRL_1759 Hyposoter fugitivus ichnovirus segment B13

VIRL_1732 Hyposoter fugitivus ichnovirus segment B14

VIRL_422 Hyposoter fugitivus ichnovirus segment B15

VIRL_1758 Hyposoter fugitivus ichnovirus segment B16

VIRL_572 Hyposoter fugitivus ichnovirus segment B17

VIRL_1757 Hyposoter fugitivus ichnovirus segment B18

VIRL_424 Hyposoter fugitivus ichnovirus segment B2

VIRL_423 Hyposoter fugitivus ichnovirus segment B3

VIRL_1762 Hyposoter fugitivus ichnovirus segment B4

VIRL_571 Hyposoter fugitivus ichnovirus segment B5

VIRL_1733 Hyposoter fugitivus ichnovirus segment B6

VIRL_195 Hyposoter fugitivus ichnovirus segment B7

VIRL_1765 Hyposoter fugitivus ichnovirus segment B8

VIRL_1761 Hyposoter fugitivus ichnovirus segment B9

VIRL_1756 Hyposoter fugitivus ichnovirus segment C1

VIRL_194 Hyposoter fugitivus ichnovirus segment C10

VIRL_1749 Hyposoter fugitivus ichnovirus segment C11

VIRL_196 Hyposoter fugitivus ichnovirus segment C12

VIRL_1731 Hyposoter fugitivus ichnovirus segment C13

VIRL_1748 Hyposoter fugitivus ichnovirus segment C14

VIRL_1747 Hyposoter fugitivus ichnovirus segment C15

VIRL_574 Hyposoter fugitivus ichnovirus segment C16

VIRL_1746 Hyposoter fugitivus ichnovirus segment C17

VIRL_1745 Hyposoter fugitivus ichnovirus segment C18

VIRL_1730 Hyposoter fugitivus ichnovirus segment C19

VIRL_1766 Hyposoter fugitivus ichnovirus segment C2

VIRL_1744 Hyposoter fugitivus ichnovirus segment C20

VIRL_569 Hyposoter fugitivus ichnovirus segment C3

VIRL_1755 Hyposoter fugitivus ichnovirus segment C4

VIRL_1754 Hyposoter fugitivus ichnovirus segment C5

VIRL_1753 Hyposoter fugitivus ichnovirus segment C6

VIRL_1752 Hyposoter fugitivus ichnovirus segment C7

VIRL_1751 Hyposoter fugitivus ichnovirus segment C8

VIRL_1750 Hyposoter fugitivus ichnovirus segment C9

VIRL_1743 Hyposoter fugitivus ichnovirus segment D1

VIRL_1737 Hyposoter fugitivus ichnovirus segment D10

VIRL_1736 Hyposoter fugitivus ichnovirus segment D11

VIRL_1735 Hyposoter fugitivus ichnovirus segment D12

VIRL_433 Hyposoter fugitivus ichnovirus segment D2

VIRL_1742 Hyposoter fugitivus ichnovirus segment D3

VIRL_1741 Hyposoter fugitivus ichnovirus segment D4

VIRL_573 Hyposoter fugitivus ichnovirus segment D5

VIRL_1740 Hyposoter fugitivus ichnovirus segment D6

VIRL_1739 Hyposoter fugitivus ichnovirus segment D7

VIRL_421 Hyposoter fugitivus ichnovirus segment D8

VIRL_1738 Hyposoter fugitivus ichnovirus segment D9

VIRL_1729 Hyposoter fugitivus ichnovirus segment E1

VIRL_1734 Hyposoter fugitivus ichnovirus segment E2

VIRL_568 Hyposoter fugitivus ichnovirus segment G1

VIRL_731 Ictalurid herpesvirus 1

VIRL_419 Ilheus virus

VIRL_3230 Impatiens necrotic spot virus segment L

VIRL_3235 Impatiens necrotic spot virus segment M

VIRL_3231 Impatiens necrotic spot virus segment S

VIRL_3520 Imperata yellow mottle virus

VIRL_3398 Indian cassava mosaic virus DNA A

VIRL_3397 Indian cassava mosaic virus DNA B

VIRL_1240 Indian citrus ringspot virus

VIRL_2417 Indian peanut clump virus RNA 1

VIRL_238 Indian peanut clump virus RNA 2

VIRL_3110 Infectious bursal disease virus segment A

VIRL_3109 Infectious bursal disease virus segment B

VIRL_3181 Infectious flacherie virus

VIRL_1273 Infectious hematopoietic necrosis virus

VIRL_457 Infectious hypodermal and hematopoietic necrosis virus

VIRL_3404 Infectious pancreatic necrosis virus segment A

VIRL_3403 Infectious pancreatic necrosis virus segment B

VIRL_344 Infectious salmon anemia virus

VIRL_343 Infectious salmon anemia virus

VIRL_345 Infectious salmon anemia virus

VIRL_346 Infectious salmon anemia virus

VIRL_449 Infectious salmon anemia virus segment 1

VIRL_347 Infectious salmon anemia virus segment 6

VIRL_318 Infectious salmon anemia virus segment 7

VIRL_319 Infectious salmon anemia virus segment 8

VIRL_1313 Infectious spleen and kidney necrosis virus

VIRL_1044 Influenza A virus (A/Goose/Guangdong/1/96(H5N1))

VIRL_1023 Influenza A virus (A/Goose/Guangdong/1/96(H5N1))

VIRL_1045 Influenza A virus (A/Goose/Guangdong/1/96(H5N1))

VIRL_1046 Influenza A virus (A/Goose/Guangdong/1/96(H5N1))

VIRL_2017 Influenza A virus (A/Goose/Guangdong/1/96(H5N1)) segment 2

VIRL_2016 Influenza A virus (A/Goose/Guangdong/1/96(H5N1)) segment 4

VIRL_1024 Influenza A virus (A/Goose/Guangdong/1/96(H5N1)) strain A/Goose/Guangdong/1/96(H5N1)

VIRL_1043 Influenza A virus (A/Goose/Guangdong/1/96(H5N1)) strain A/Goose/Guangdong/1/96(H5N1)

VIRL_2112 Influenza A virus (A/Hong Kong/1073/99(H9N2))

VIRL_2107 Influenza A virus (A/Hong Kong/1073/99(H9N2)) segment 1

VIRL_2106 Influenza A virus (A/Hong Kong/1073/99(H9N2)) segment 2

VIRL_2105 Influenza A virus (A/Hong Kong/1073/99(H9N2)) segment 3

VIRL_2109 Influenza A virus (A/Hong Kong/1073/99(H9N2)) segment 4

VIRL_2108 Influenza A virus (A/Hong Kong/1073/99(H9N2)) segment 6

VIRL_2110 Influenza A virus (A/Hong Kong/1073/99(H9N2)) segment 7

VIRL_2111 Influenza A virus (A/Hong Kong/1073/99(H9N2)) segment 8

VIRL_1042 Influenza A virus (A/Korea/426/1968(H2N2))

VIRL_2406 Influenza A virus (A/Korea/426/68(H2N2)) segment 2

VIRL_2006 Influenza A virus (A/Korea/426/68(H2N2)) segment 3

VIRL_2007 Influenza A virus (A/Korea/426/68(H2N2)) segment 4

VIRL_3681 Influenza A virus (A/Korea/426/68(H2N2)) segment 5

VIRL_3680 Influenza A virus (A/Korea/426/68(H2N2)) segment 6

VIRL_2005 Influenza A virus (A/Korea/426/68(H2N2)) segment 7

VIRL_3682 Influenza A virus (A/Korea/426/68(H2N2)) segment 8

VIRL_2008 Influenza A virus (A/New York/392/2004(H3N2)) segment 1

VIRL_2009 Influenza A virus (A/New York/392/2004(H3N2)) segment 2

VIRL_2010 Influenza A virus (A/New York/392/2004(H3N2)) segment 3

VIRL_2015 Influenza A virus (A/New York/392/2004(H3N2)) segment 4

VIRL_2012 Influenza A virus (A/New York/392/2004(H3N2)) segment 5

VIRL_2013 Influenza A virus (A/New York/392/2004(H3N2)) segment 6

VIRL_2014 Influenza A virus (A/New York/392/2004(H3N2)) segment 7

VIRL_2011 Influenza A virus (A/New York/392/2004(H3N2)) segment 8

VIRL_2387 Influenza A virus (A/Puerto Rico/8/34(H1N1)) segment 1

VIRL_2491 Influenza A virus (A/Puerto Rico/8/34(H1N1)) segment 2

VIRL_2388 Influenza A virus (A/Puerto Rico/8/34(H1N1)) segment 3

VIRL_2391 Influenza A virus (A/Puerto Rico/8/34(H1N1)) segment 4

VIRL_3615 Influenza A virus (A/Puerto Rico/8/34(H1N1)) segment 5

VIRL_2390 Influenza A virus (A/Puerto Rico/8/34(H1N1)) segment 6

VIRL_2392 Influenza A virus (A/Puerto Rico/8/34(H1N1)) segment 7

VIRL_2389 Influenza A virus (A/Puerto Rico/8/34(H1N1)) segment 8

VIRL_2348 Influenza B virus RNA 1

VIRL_3611 Influenza B virus RNA 4

VIRL_2345 Influenza B virus RNA 5

VIRL_2344 Influenza B virus RNA 6

VIRL_2452 Influenza B virus RNA 7

VIRL_2343 Influenza B virus RNA 8

VIRL_2347 Influenza B virus RNA-2

VIRL_2346 Influenza B virus RNA-3

VIRL_87 Influenza C virus (C/Ann Arbor/1/50) segment 1

VIRL_86 Influenza C virus (C/Ann Arbor/1/50) segment 2

VIRL_85 Influenza C virus (C/Ann Arbor/1/50) segment 3

VIRL_84 Influenza C virus (C/Ann Arbor/1/50) segment 4

VIRL_83 Influenza C virus (C/Ann Arbor/1/50) segment 5

VIRL_82 Influenza C virus (C/Ann Arbor/1/50) segment 6

VIRL_88 Influenza C virus (C/Ann Arbor/1/50) segment 7

VIRL_2319 Invertebrate iridescent virus 6

VIRL_3512 Iodobacteriophage phiPLPE

VIRL_821 Ipomoea yellow vein virus

VIRL_3596 Ippy virus segment L

VIRL_3597 Ippy virus segment S

VIRL_3525 Iranian maize mosaic nucleorhabdovirus

VIRL_2831 Israel acute paralysis virus of bees

VIRL_1040 J-virus

VIRL_1282 Jaagsiekte sheep retrovirus

VIRL_3478 Japanese encephalitis virus

VIRL_789 Japanese holly fern mottle virus RNA1

VIRL_788 Japanese holly fern mottle virus RNA2

VIRL_2351 Japanese iris necrotic ring virus

VIRL_3371 Japanese yam mosaic virus

VIRL_838 Jatropha leaf curl virus DNA A

VIRL_3565 Jatropha yellow mosaic India virus DNA-A

VIRL_701 JC polyomavirus

VIRL_2087 Johnsongrass chlorotic stripe mosaic virus

VIRL_2279 Johnsongrass mosaic virus

VIRL_1138 Junin virus segment L

VIRL_1137 Junin virus segment S

VIRL_233 Junonia coenia densovirus

VIRL_1196 Kadipiro virus chromosome segment 1

VIRL_362 Kadipiro virus chromosome segment 10

VIRL_363 Kadipiro virus chromosome segment 12

VIRL_359 Kadipiro virus chromosome segment 2

VIRL_358 Kadipiro virus chromosome segment 3

VIRL_357 Kadipiro virus chromosome segment 4

VIRL_356 Kadipiro virus chromosome segment 5

VIRL_355 Kadipiro virus chromosome segment 6

VIRL_2156 Kadipiro virus chromosome segment 7

VIRL_360 Kadipiro virus chromosome segment 8

VIRL_361 Kadipiro virus chromosome segment 9

VIRL_2157 Kadipiro virus segment 11

VIRL_3499 Kakugo virus

VIRL_962 Kalanchoe latent virus

VIRL_759 Kalanchoe top-spotting virus

VIRL_1183 Kamiti River virus

VIRL_1058 Karshi virus

VIRL_1186 Kashmir bee virus

VIRL_2667 Kedougou virus

VIRL_757 Kelp fly virus

VIRL_861 Kenaf leaf curl virus DNA A

VIRL_3613 Kennedya yellow mosaic virus

VIRL_565 KI polyomavirus Stockholm 60

VIRL_2512 Klebsiella phage K11

VIRL_426 Klebsiella phage KP15

VIRL_258 Klebsiella phage KP32

VIRL_427 Klebsiella phage KP34

VIRL_2580 Klebsiella phage phiKO2

VIRL_512 Kluyvera phage Kvp1

VIRL_926 Kobuvirus pig/JY-2010a/CHN

VIRL_418 Kokobera virus

VIRL_2698 Konjac mosaic virus

VIRL_869 Kudzu mosaic virus DNA-A

VIRL_868 Kudzu mosaic virus DNA-B

VIRL_3236 Kyuri green mottle mosaic virus

VIRL_1200 La Crosse virus segment L

VIRL_1199 La Crosse virus segment M

VIRL_1198 La Crosse virus segment S

VIRL_3438 Lactate dehydrogenase-elevating virus

VIRL_1664 Lactobacillus johnsonii prophage Lj771

VIRL_2167 Lactobacillus phage A2

VIRL_1033 Lactobacillus phage KC5a

VIRL_1296 Lactobacillus phage Lb338-1

VIRL_1039 Lactobacillus phage Lc-Nu

VIRL_747 Lactobacillus phage LL-H

VIRL_2055 Lactobacillus phage LP65

VIRL_3600 Lactobacillus phage Lrm1

VIRL_2717 Lactobacillus phage Lv-1

VIRL_2574 Lactobacillus phage phiAT3

VIRL_1359 Lactobacillus phage phig1e

VIRL_2569 Lactobacillus phage phiJL-1

VIRL_1461 Lactobacillus prophage Lj928

VIRL_1460 Lactobacillus prophage Lj965

VIRL_2460 Lactobacillus prophage phiadh

VIRL_3651 Lactococcus phage 1706

VIRL_2596 Lactococcus phage 4268

VIRL_2711 Lactococcus phage 712

VIRL_2522 Lactococcus phage asccphi28

VIRL_1605 Lactococcus phage bIBB29

VIRL_1583 Lactococcus phage bIL170

VIRL_3550 Lactococcus phage bIL67

VIRL_2620 Lactococcus phage BK5-T

VIRL_2376 Lactococcus phage c2

VIRL_2710 Lactococcus phage jj50

VIRL_2533 Lactococcus phage KSY1

VIRL_1913 Lactococcus phage P008

VIRL_1421 Lactococcus phage P087

VIRL_2582 Lactococcus phage phiLC3

VIRL_1912 Lactococcus phage Q54

VIRL_2603 Lactococcus phage r1t

VIRL_1534 Lactococcus phage sk1

VIRL_2621 Lactococcus phage TP901-1

VIRL_2322 Lactococcus phage Tuc2009

VIRL_2609 Lactococcus phage ul36

VIRL_2327 Lactococcus prophage bIL285

VIRL_2326 Lactococcus prophage bIL286

VIRL_2623 Lactococcus prophage bIL309

VIRL_2443 Lactococcus prophage bIL310

VIRL_2442 Lactococcus prophage bIL311

VIRL_2325 Lactococcus prophage bIL312

VIRL_3443 Lake Victoria marburgvirus - Musoke

VIRL_1630 Lamium leaf distortion associated virus

VIRL_1224 Langat virus

VIRL_3070 Lassa virus segment L

VIRL_3071 Lassa virus segment S

VIRL_3593 Latino virus segment L

VIRL_2763 Latino virus segment S

VIRL_2370 Leek white stripe virus

VIRL_2181 Leek yellow stripe virus

VIRL_221 Leishmania RNA virus 1 - 1

VIRL_1225 Leishmania RNA virus 1 - 4

VIRL_219 Leishmania RNA virus 2 - 1

VIRL_3515 Lettuce big-vein associated virus RNA 1

VIRL_3513 Lettuce big-vein associated virus segment 2

VIRL_128 Lettuce chlorosis virus RNA1

VIRL_127 Lettuce chlorosis virus RNA2

VIRL_1500 Lettuce infectious yellows virus RNA 1

VIRL_1499 Lettuce infectious yellows virus RNA 2

VIRL_3239 Lettuce mosaic virus

VIRL_1975 Lettuce necrotic yellows virus

VIRL_1119 Lettuce ring necrosis virus RNA 1

VIRL_1118 Lettuce ring necrosis virus RNA 2

VIRL_1117 Lettuce ring necrosis virus RNA 3

VIRL_1116 Lettuce ring necrosis virus RNA 4

VIRL_3530 Lettuce virus X

VIRL_3522 Lettuce yellow mottle virus

VIRL_3665 Leucania separata nuclear polyhedrosis virus

VIRL_944 Leucas zeylanica yellow vein virus satellite DNA beta

VIRL_1709 Leuconostoc phage L5

VIRL_1078 Liao ning virus segment 1

VIRL_1069 Liao ning virus segment 10

VIRL_1068 Liao ning virus segment 11

VIRL_1067 Liao ning virus segment 12

VIRL_1077 Liao ning virus segment 2

VIRL_1076 Liao ning virus segment 3

VIRL_1075 Liao ning virus segment 4

VIRL_1074 Liao ning virus segment 5

VIRL_1073 Liao ning virus segment 6

VIRL_1072 Liao ning virus segment 7

VIRL_1071 Liao ning virus segment 8

VIRL_1070 Liao ning virus segment 9

VIRL_2650 Ligustrum necrotic ringspot virus

VIRL_2979 Lily mottle virus

VIRL_3003 Lily symptomless virus

VIRL_2023 Lily virus X

VIRL_874 Lindernia anagallis yellow vein virus DNA-A

VIRL_1699 Lindernia anagallis yellow vein virus satellite DNA beta

VIRL_2863 Lisianthus necrosis virus

VIRL_1302 Listeria phage A006

VIRL_2317 Listeria phage A118

VIRL_1306 Listeria phage A500

VIRL_2534 Listeria phage A511

VIRL_1305 Listeria phage B025

VIRL_1304 Listeria phage B054

VIRL_1303 Listeria phage P35

VIRL_1297 Listeria phage P40

VIRL_2712 Listonella phage phiHSIC

VIRL_2368 Little cherry virus 1

VIRL_2102 Little cherry virus 2

VIRL_3139 Ljungan virus

VIRL_3639 Lolium latent virus

VIRL_2114 Loofa yellow mosaic virus DNA A

VIRL_2113 Loofa yellow mosaic virus DNA B

VIRL_2373 Louping ill virus

VIRL_2377 Lucerne transient streak virus

VIRL_3178 Lucerne transient streak virus satellite RNA

VIRL_1695 Lucky bamboo bacilliform virus

VIRL_2778 Ludwigia leaf distortion betasatellite [India

VIRL_2894 Ludwigia yellow vein virus DNA-A

VIRL_2022 Ludwigia yellow vein virus-associated DNA beta

VIRL_1946 Luffa begomovirus associated DNA beta

VIRL_1079 Luffa puckering and leaf distortion-associated DNA beta

VIRL_2418 LuIII virus

VIRL_952 Lujo virus segment L

VIRL_953 Lujo virus segment S

VIRL_682 Lumpy skin disease virus NI-2490

VIRL_3329 Lymantria dispar cypovirus 1 segment 1

VIRL_3320 Lymantria dispar cypovirus 1 segment 10

VIRL_3328 Lymantria dispar cypovirus 1 segment 2

VIRL_3327 Lymantria dispar cypovirus 1 segment 3

VIRL_3326 Lymantria dispar cypovirus 1 segment 4

VIRL_3325 Lymantria dispar cypovirus 1 segment 5

VIRL_3324 Lymantria dispar cypovirus 1 segment 6

VIRL_3323 Lymantria dispar cypovirus 1 segment 7

VIRL_3322 Lymantria dispar cypovirus 1 segment 8

VIRL_3321 Lymantria dispar cypovirus 1 segment 9

VIRL_752 Lymantria dispar MNPV

VIRL_465 Lymantria xylina MNPV

VIRL_1455 Lymphocystis disease virus - isolate China

VIRL_2472 Lymphocystis disease virus 1

VIRL_3073 Lymphocytic choriomeningitis virus segment L

VIRL_3072 Lymphocytic choriomeningitis virus segment S

VIRL_492 Macaca fascicularis papillomavirus type 10

VIRL_494 Macaca fascicularis papillomavirus type 7

VIRL_493 Macaca fascicularis papillomavirus type 9

VIRL_354 Macacine herpesvirus 1

VIRL_59 Macacine herpesvirus 3

VIRL_638 Macacine herpesvirus 4

VIRL_675 Macacine herpesvirus 5

VIRL_1621 Macaque simian foamy virus

VIRL_1139 Machupo virus segment L

VIRL_1181 Machupo virus segment S

VIRL_1135 Macrobrachium rosenbergii nodavirus RNA-1

VIRL_3004 Macrobrachium rosenbergii nodavirus RNA-2

VIRL_2760 Macroptilium golden mosaic virus-[Jamaica

VIRL_1615 Macroptilium golden mosaic virus-[Jamaica

VIRL_3122 Macroptilium mosaic Puerto Rico virus DNA A

VIRL_3121 Macroptilium mosaic Puerto Rico virus DNA B

VIRL_2707 Macroptilium yellow mosaic Florida virus DNA A

VIRL_2169 Macroptilium yellow mosaic Florida virus DNA B

VIRL_2776 Macroptilium yellow mosaic virus DNA A

VIRL_2775 Macroptilium yellow mosaic virus DNA B

VIRL_69 Magnaporthe oryzae chrysovirus 1 segment 1

VIRL_66 Magnaporthe oryzae chrysovirus 1 segment 2

VIRL_68 Magnaporthe oryzae chrysovirus 1 segment 3

VIRL_67 Magnaporthe oryzae chrysovirus 1 segment 4

VIRL_2062 Magnaporthe oryzae virus 1

VIRL_2697 Magnaporthe oryzae virus 2

VIRL_3229 Maize chlorotic dwarf virus

VIRL_3228 Maize chlorotic mottle virus

VIRL_3299 Maize dwarf mosaic virus

VIRL_1311 Maize fine streak virus

VIRL_227 Maize mosaic virus

VIRL_1307 Maize necrotic streak virus

VIRL_2321 Maize rayado fino virus

VIRL_3491 Maize streak virus - A[South Africa]

VIRL_1710 Maize white line mosaic virus

VIRL_1875 Mal de Rio Cuarto virus segment 9

VIRL_1877 Mal de Rio Cuarto virus segment S1

VIRL_1066 Mal de Rio Cuarto virus segment S10

VIRL_1880 Mal de Rio Cuarto virus segment S2

VIRL_1878 Mal de Rio Cuarto virus segment S3

VIRL_1881 Mal de Rio Cuarto virus segment S4

VIRL_1065 Mal de Rio Cuarto virus segment S5

VIRL_1879 Mal de Rio Cuarto virus segment S6

VIRL_1876 Mal de Rio Cuarto virus segment S7

VIRL_1882 Mal de Rio Cuarto virus segment S8

VIRL_1653 Malachra yellow vein mosaic virus-associated satellite DNA beta

VIRL_995 Malva mosaic virus

VIRL_891 Malvastrum leaf curl Guangdong virus

VIRL_2869 Malvastrum leaf curl virus - [G87]

VIRL_1959 Malvastrum leaf curl virus-associated defective DNA beta

VIRL_1961 Malvastrum leaf curl virus-associated DNA beta

VIRL_2844 Malvastrum yellow mosaic virus DNA-A

VIRL_2843 Malvastrum yellow mosaic virus satellite DNA beta

VIRL_2842 Malvastrum yellow mosaic virus-associated DNA 1

VIRL_1419 Malvastrum yellow vein Baoshan virus DNA-A

VIRL_3041 Malvastrum yellow vein virus

VIRL_3027 Malvastrum yellow vein virus satellite DNA beta

VIRL_2053 Malvastrum yellow vein Yunnan virus

VIRL_2915 Malvastrum yellow vein Yunnan virus satellite DNA beta

VIRL_368 Mamestra configurata NPV-A

VIRL_751 Mamestra configurata NPV-B

VIRL_3085 Mammalian orthoreovirus 1 segment L1

VIRL_3097 Mammalian orthoreovirus 1 segment L2

VIRL_3101 Mammalian orthoreovirus 1 segment L3

VIRL_3095 Mammalian orthoreovirus 1 segment M1

VIRL_3094 Mammalian orthoreovirus 1 segment M2

VIRL_3099 Mammalian orthoreovirus 1 segment M3

VIRL_3089 Mammalian orthoreovirus 1 segment S1

VIRL_3088 Mammalian orthoreovirus 1 segment S2

VIRL_3090 Mammalian orthoreovirus 1 segment S3

VIRL_3091 Mammalian orthoreovirus 1 segment S4

VIRL_3084 Mammalian orthoreovirus 2 segment L1

VIRL_3096 Mammalian orthoreovirus 2 segment L2

VIRL_3100 Mammalian orthoreovirus 2 segment L3

VIRL_3102 Mammalian orthoreovirus 2 segment M1

VIRL_3086 Mammalian orthoreovirus 2 segment M2

VIRL_3098 Mammalian orthoreovirus 2 segment M3

VIRL_3092 Mammalian orthoreovirus 2 segment S1

VIRL_3093 Mammalian orthoreovirus 2 segment S2

VIRL_3087 Mammalian orthoreovirus 2 segment S3

VIRL_3083 Mammalian orthoreovirus 2 segment S4

VIRL_408 Mammalian orthoreovirus 3 segment L1

VIRL_406 Mammalian orthoreovirus 3 segment L1

VIRL_405 Mammalian orthoreovirus 3 segment L2

VIRL_3081 Mammalian orthoreovirus 3 segment L2

VIRL_402 Mammalian orthoreovirus 3 segment L3

VIRL_3082 Mammalian orthoreovirus 3 segment L3

VIRL_404 Mammalian orthoreovirus 3 segment M1

VIRL_3076 Mammalian orthoreovirus 3 segment M1

VIRL_3078 Mammalian orthoreovirus 3 segment M2

VIRL_403 Mammalian orthoreovirus 3 segment M2

VIRL_3075 Mammalian orthoreovirus 3 segment M3

VIRL_401 Mammalian orthoreovirus 3 segment M3

VIRL_3079 Mammalian orthoreovirus 3 segment S1

VIRL_400 Mammalian orthoreovirus 3 segment S1

VIRL_399 Mammalian orthoreovirus 3 segment S2

VIRL_3077 Mammalian orthoreovirus 3 segment S2

VIRL_3074 Mammalian orthoreovirus 3 segment S3

VIRL_398 Mammalian orthoreovirus 3 segment S3

VIRL_3080 Mammalian orthoreovirus 3 segment S4

VIRL_397 Mammalian orthoreovirus 3 segment S4

VIRL_587 Mannheimia phage phiMHaA1

VIRL_3668 Mapuera virus

VIRL_3679 Maracuja mosaic virus

VIRL_1152 Marine birnavirus segment A

VIRL_2860 Marine birnavirus segment B

VIRL_1685 Marine RNA virus JP-A

VIRL_1684 Marine RNA virus JP-B

VIRL_1686 Marine RNA virus SOG

VIRL_925 Marseillevirus sp

VIRL_3583 Maruca vitrata MNPV

VIRL_3449 Mason-Pfizer monkey virus

VIRL_578 Mastomys coucha papillomavirus 2

VIRL_712 Mastomys natalensis papillomavirus

VIRL_3291 Mayaro virus

VIRL_39 Measles virus

VIRL_1340 Melandrium yellow fleck virus RNA1

VIRL_1339 Melandrium yellow fleck virus RNA2

VIRL_1338 Melandrium yellow fleck virus RNA3

VIRL_694 Melanoplus sanguinipes entomopoxvirus

VIRL_378 Meleagrid herpesvirus 1

VIRL_1622 Melon aphid-borne yellows virus

VIRL_90 Melon chlorotic leaf curl virus DNA A

VIRL_158 Melon chlorotic mosaic virus DNA-A

VIRL_157 Melon chlorotic mosaic virus DNA-B

VIRL_159 Melon chlorotic mosaic virus-associated alphasatellite

VIRL_3455 Melon necrotic spot virus

VIRL_2688 Melon yellow spot virus segment L

VIRL_2687 Melon yellow spot virus segment M

VIRL_2851 Melon yellow spot virus segment S

VIRL_1037 Menangle virus

VIRL_542 Merkel cell polyomavirus

VIRL_2865 Merremia mosaic virus DNA A

VIRL_2864 Merremia mosaic virus DNA B

VIRL_2761 Mesta yellow vein mosaic Bahraich virus-[India

VIRL_883 Mesta yellow vein mosaic virus DNA-A

VIRL_2652 Mesta yellow vein mosaic virus-associated DNA beta

VIRL_2469 Methanobacterium phage psiM2

VIRL_1581 Methanothermobacter prophage psiM100

VIRL_2812 Microbacterium phage Min1

VIRL_1895 Microcystis phage Ma-LMM01

VIRL_1940 Micromonas pusilla reovirus segment 1

VIRL_1931 Micromonas pusilla reovirus segment 10

VIRL_1930 Micromonas pusilla reovirus segment 11

VIRL_1939 Micromonas pusilla reovirus segment 2

VIRL_1938 Micromonas pusilla reovirus segment 3

VIRL_1937 Micromonas pusilla reovirus segment 4

VIRL_1936 Micromonas pusilla reovirus segment 5

VIRL_1935 Micromonas pusilla reovirus segment 6

VIRL_1934 Micromonas pusilla reovirus segment 7

VIRL_1933 Micromonas pusilla reovirus segment 8

VIRL_1932 Micromonas pusilla reovirus segment 9

VIRL_1085 Microplitis demolitor bracovirus segment A

VIRL_615 Microplitis demolitor bracovirus segment B

VIRL_614 Microplitis demolitor bracovirus segment C

VIRL_1053 Microplitis demolitor bracovirus segment D

VIRL_3588 Microplitis demolitor bracovirus segment E

VIRL_613 Microplitis demolitor bracovirus segment F

VIRL_612 Microplitis demolitor bracovirus segment G

VIRL_611 Microplitis demolitor bracovirus segment H

VIRL_607 Microplitis demolitor bracovirus segment I

VIRL_610 Microplitis demolitor bracovirus segment J

VIRL_1052 Microplitis demolitor bracovirus segment K

VIRL_1051 Microplitis demolitor bracovirus segment L

VIRL_609 Microplitis demolitor bracovirus segment M

VIRL_608 Microplitis demolitor bracovirus segment N

VIRL_1050 Microplitis demolitor bracovirus segment O

VIRL_1415 Midway virus

VIRL_131 Mikania micrantha mosaic virus RNA1

VIRL_132 Mikania micrantha mosaic virus RNA2

VIRL_1578 Milk vetch dwarf virus segment 1

VIRL_3493 Milk vetch dwarf virus segment 10

VIRL_2266 Milk vetch dwarf virus segment 11

VIRL_1577 Milk vetch dwarf virus segment 2

VIRL_1576 Milk vetch dwarf virus segment 3

VIRL_1575 Milk vetch dwarf virus segment 4

VIRL_2270 Milk vetch dwarf virus segment 5

VIRL_2269 Milk vetch dwarf virus segment 6

VIRL_2268 Milk vetch dwarf virus segment 7

VIRL_2267 Milk vetch dwarf virus segment 8

VIRL_3225 Milk vetch dwarf virus segment 9

VIRL_878 Mimosa yellow leaf curl virus DNA-A

VIRL_1703 Mimosa yellow leaf curl virus satellite DNA beta

VIRL_1696 Mimosa yellow leaf curl virus-associated DNA 1

VIRL_2421 Mink astrovirus

VIRL_256 Mint virus 1

VIRL_1057 Mint virus X

VIRL_977 Minute virus of mice

VIRL_1208 Mirabilis mosaic virus

VIRL_3503 Mirafiore lettuce virus RNA 1

VIRL_3501 Mirafiore lettuce virus RNA 2

VIRL_3023 Mirafiore lettuce virus RNA 3

VIRL_3502 Mirafiore lettuce virus RNA 4

VIRL_3298 Miscanthus streak virus - [91]

VIRL_3598 Mobala virus segment L

VIRL_3599 Mobala virus segment S

VIRL_1498 Modoc virus

VIRL_2920 Mokola virus

VIRL_1269 Molluscum contagiosum virus subtype 1

VIRL_3457 Moloney murine leukemia virus

VIRL_2487 Moloney murine sarcoma virus

VIRL_679 Monkeypox virus Zaire-96-I-16

VIRL_1486 Montana myotis leukoencephalitis virus

VIRL_1094 Mopeia Lassa reassortant 29 segment L

VIRL_1093 Mopeia Lassa reassortant 29 segment S

VIRL_1092 Mopeia virus AN20410 segment L

VIRL_1091 Mopeia virus AN20410 segment S

VIRL_534 Morganella phage MmP1

VIRL_769 Moroccan watermelon mosaic virus

VIRL_1028 Morogoro virus segment L

VIRL_1029 Morogoro virus segment S

VIRL_251 Mossman virus

VIRL_3456 Mouse mammary tumor virus

VIRL_235 Mouse parvovirus 1

VIRL_1928 Mouse parvovirus 2

VIRL_1929 Mouse parvovirus 3

VIRL_831 Mouse parvovirus 4

VIRL_832 Mouse parvovirus 5

VIRL_1063 Mulard duck circovirus

VIRL_80 Mumps virus

VIRL_3050 Mungbean yellow mosaic India virus DNA A

VIRL_3049 Mungbean yellow mosaic India virus DNA B

VIRL_2465 Mungbean yellow mosaic virus DNA A

VIRL_2466 Mungbean yellow mosaic virus DNA B

VIRL_2718 Munia coronavirus HKU13-3514

VIRL_668 Murid herpesvirus 1

VIRL_62 Murid herpesvirus 2

VIRL_391 Murid herpesvirus 4

VIRL_2405 Murine adenovirus 3

VIRL_2456 Murine adenovirus A

VIRL_1353 Murine adenovirus A

VIRL_2471 Murine hepatitis virus strain A59

VIRL_2914 Murine hepatitis virus strain JHM

VIRL_2846 Murine norovirus 1

VIRL_2486 Murine osteosarcoma virus

VIRL_730 Murine pneumotropic virus

VIRL_729 Murine polyomavirus

VIRL_3430 Murine type C retrovirus

VIRL_3372 Murray Valley encephalitis virus

VIRL_174 Mus musculus papillomavirus type 1

VIRL_1540 Musca domestica salivary gland hypertrophy virus

VIRL_901 Muscovy duck circovirus

VIRL_226 Muscovy duck parvovirus

VIRL_2379 Mushroom bacilliform virus

VIRL_2552 Mycobacterium phage 244

VIRL_2520 Mycobacterium phage Adjutor

VIRL_951 Mycobacterium phage Angel

VIRL_72 Mycobacterium phage Angelica

VIRL_766 Mycobacterium phage Ardmore

VIRL_1471 Mycobacterium phage Barnyard

VIRL_2528 Mycobacterium phage Bethlehem

VIRL_2510 Mycobacterium phage Boomer

VIRL_1300 Mycobacterium phage BPs

VIRL_3567 Mycobacterium phage Brujita

VIRL_524 Mycobacterium phage Butterscotch

VIRL_3561 Mycobacterium phage Bxb1

VIRL_3558 Mycobacterium phage Bxz1

VIRL_3559 Mycobacterium phage Bxz2

VIRL_3569 Mycobacterium phage Cali

VIRL_1919 Mycobacterium phage Catera

VIRL_3568 Mycobacterium phage Chah

VIRL_1922 Mycobacterium phage Che12

VIRL_1475 Mycobacterium phage Che8

VIRL_2120 Mycobacterium phage Che9c

VIRL_1472 Mycobacterium phage Che9d

VIRL_2598 Mycobacterium phage Cjw1

VIRL_1926 Mycobacterium phage Cooper

VIRL_1473 Mycobacterium phage Corndog

VIRL_71 Mycobacterium phage CrimD

VIRL_2470 Mycobacterium phage D29

VIRL_2517 Mycobacterium phage DD5

VIRL_999 Mycobacterium phage ET08

VIRL_522 Mycobacterium phage Fruitloop

VIRL_1293 Mycobacterium phage Giles

VIRL_520 Mycobacterium phage Gumball

VIRL_1923 Mycobacterium phage Halo

VIRL_1609 Mycobacterium phage Jasper

VIRL_1610 Mycobacterium phage KBG

VIRL_3566 Mycobacterium phage Konstantine

VIRL_2508 Mycobacterium phage Kostya

VIRL_2404 Mycobacterium phage L5

VIRL_70 Mycobacterium phage LeBron

VIRL_2551 Mycobacterium phage Llij

VIRL_2518 Mycobacterium phage Lockley

VIRL_527 Mycobacterium phage Myrna

VIRL_1606 Mycobacterium phage Nigel

VIRL_2597 Mycobacterium phage Omega

VIRL_1925 Mycobacterium phage Orion

VIRL_523 Mycobacterium phage Pacc40

VIRL_2550 Mycobacterium phage PBI1

VIRL_939 Mycobacterium phage Peaches

VIRL_1464 Mycobacterium phage PG1

VIRL_1604 Mycobacterium phage Phaedrus

VIRL_2693 Mycobacterium phage Phlyer

VIRL_1924 Mycobacterium phage Pipefish

VIRL_2549 Mycobacterium phage PLot

VIRL_2548 Mycobacterium phage PMC

VIRL_2509 Mycobacterium phage Porky

VIRL_1607 Mycobacterium phage Predator

VIRL_1608 Mycobacterium phage Pukovnik

VIRL_1921 Mycobacterium phage Qyrzula

VIRL_521 Mycobacterium phage Ramsey

VIRL_3579 Mycobacterium phage Rizal

VIRL_1474 Mycobacterium phage Rosebush

VIRL_3581 Mycobacterium phage ScottMcG

VIRL_3582 Mycobacterium phage Solon

VIRL_3580 Mycobacterium phage Spud

VIRL_3560 Mycobacterium phage TM4

VIRL_525 Mycobacterium phage Troll4

VIRL_2530 Mycobacterium phage Tweety

VIRL_1679 Mycobacterium phage U2

VIRL_1920 Mycobacterium phage Wildcat

VIRL_2362 Mycoplasma phage MAV1

VIRL_1521 Mycoplasma phage P1

VIRL_3654 Mycoplasma phage phiMFV1

VIRL_1628 Mycoreovirus 1 segment 1

VIRL_2733 Mycoreovirus 1 segment 10

VIRL_2732 Mycoreovirus 1 segment 11

VIRL_1627 Mycoreovirus 1 segment 2

VIRL_1626 Mycoreovirus 1 segment 3

VIRL_2739 Mycoreovirus 1 segment 4

VIRL_2738 Mycoreovirus 1 segment 5

VIRL_2737 Mycoreovirus 1 segment 6

VIRL_2736 Mycoreovirus 1 segment 7

VIRL_2735 Mycoreovirus 1 segment 8

VIRL_2734 Mycoreovirus 1 segment 9

VIRL_1995 Mycoreovirus 3 segment 10

VIRL_1994 Mycoreovirus 3 segment 11

VIRL_1993 Mycoreovirus 3 segment 12

VIRL_1999 Mycoreovirus 3 segment 6

VIRL_1998 Mycoreovirus 3 segment 7

VIRL_1997 Mycoreovirus 3 segment 8

VIRL_1996 Mycoreovirus 3 segment 9

VIRL_1990 Mycoreovirus 3 segment S1

VIRL_1992 Mycoreovirus 3 segment S2

VIRL_1989 Mycoreovirus 3 segment S3

VIRL_1991 Mycoreovirus 3 segment S5

VIRL_2000 Mycoreovirus 3 segment X

VIRL_2193 Mycovirus FusoV RNA 1

VIRL_2192 Mycovirus FusoV RNA 2

VIRL_518 Myotis polyomavirus VM-2008

VIRL_162 Mythimna loreyi densovirus

VIRL_2681 Myxococcus phage Mx8

VIRL_691 Myxoma virus

VIRL_910 Myzus persicae densovirus

VIRL_2951 Nanovirus-like particle

VIRL_1917 Narcissus common latent virus

VIRL_1872 Narcissus degeneration virus

VIRL_3474 Narcissus mosaic virus

VIRL_1896 Narcissus symptomless virus

VIRL_3516 Narcissus yellow stripe virus

VIRL_2170 Natrialba phage PhiCh1

VIRL_3518 Nemesia ring necrosis virus

VIRL_983 Neodiprion abietis NPV

VIRL_1121 Neodiprion lecontii NPV

VIRL_1423 Neodiprion sertifer NPV

VIRL_1963 Nerine virus X

VIRL_3677 Newbury agent 1 virus

VIRL_1247 Newcastle disease virus B1

VIRL_468 Ngaingan virus

VIRL_2263 Nilaparvata lugens reovirus segment 1

VIRL_2265 Nilaparvata lugens reovirus segment 10

VIRL_2262 Nilaparvata lugens reovirus segment 2

VIRL_2261 Nilaparvata lugens reovirus segment 3

VIRL_2260 Nilaparvata lugens reovirus segment 4

VIRL_2259 Nilaparvata lugens reovirus segment 5

VIRL_2258 Nilaparvata lugens reovirus segment 6

VIRL_2257 Nilaparvata lugens reovirus segment 7

VIRL_2264 Nilaparvata lugens reovirus segment 8

VIRL_2256 Nilaparvata lugens reovirus segment 9

VIRL_3348 Nipah virus

VIRL_2323 Nodamura virus RNA1

VIRL_1245 Nodamura virus RNA2

VIRL_1726 Nootka lupine vein-clearing virus

VIRL_1384 Nora virus

VIRL_2450 Northern cereal mosaic virus

VIRL_3388 Norwalk virus

VIRL_2361 Nudaurelia capensis beta virus

VIRL_1414 Nyamanini virus

VIRL_1317 O'nyong-nyong virus

VIRL_2374 Oat blue dwarf virus

VIRL_2272 Oat chlorotic stunt virus

VIRL_854 Oat dwarf virus

VIRL_1525 Oat golden stripe virus RNA 1

VIRL_1526 Oat golden stripe virus RNA 2

VIRL_1491 Oat mosaic virus RNA 1

VIRL_3535 Oat mosaic virus RNA 2

VIRL_1466 Oat necrotic mottle virus

VIRL_3156 Obuda pepper virus

VIRL_3426 Odontoglossum ringspot virus

VIRL_3123 OkLCV satDNA 10

VIRL_1301 Okra leaf curl disease associated DNA 1

VIRL_2678 Okra leaf curl Mali virus satellite DNA beta

VIRL_1391 Okra leaf curl virus-[Cameroon]

VIRL_1711 Okra mosaic virus

VIRL_1600 Okra mottle virus - [Brazil:okra] DNA A

VIRL_1599 Okra mottle virus - [Brazil:okra] DNA B

VIRL_2845 Okra yellow crinkle virus segment A

VIRL_277 Okra yellow mosaic Mexico virus DNA A

VIRL_276 Okra yellow mosaic Mexico virus DNA B

VIRL_3010 Okra yellow vein disease associated sequence virion

VIRL_3606 Okra yellow vein mosaic virus

VIRL_577 Old World harvest mouse papillomavirus

VIRL_3427 Olive latent virus 1

VIRL_2250 Olive latent virus 2 RNA 1

VIRL_2249 Olive latent virus 2 RNA 2

VIRL_2252 Olive latent virus 2 RNA 3

VIRL_160 Olive latent virus 3

VIRL_1090 Olive mild mosaic virus

VIRL_1659 Oliveros virus segment L

VIRL_1661 Oliveros virus segment S

VIRL_1141 Omsk hemorrhagic fever virus

VIRL_3017 Onion yellow dwarf virus

VIRL_2385 Ononis yellow mosaic virus

VIRL_1038 Operophtera brumata reovirus segment 1

VIRL_1980 Operophtera brumata reovirus segment 10

VIRL_1988 Operophtera brumata reovirus segment 2

VIRL_1987 Operophtera brumata reovirus segment 3

VIRL_1986 Operophtera brumata reovirus segment 4

VIRL_1985 Operophtera brumata reovirus segment 5

VIRL_1984 Operophtera brumata reovirus segment 6

VIRL_1983 Operophtera brumata reovirus segment 7

VIRL_1982 Operophtera brumata reovirus segment 8

VIRL_1981 Operophtera brumata reovirus segment 9

VIRL_2174 Ophiostoma mitovirus 3a

VIRL_2173 Ophiostoma mitovirus 4

VIRL_2172 Ophiostoma mitovirus 5

VIRL_2171 Ophiostoma novo-ulmi mitovirus 6-Ld

VIRL_1114 Opuntia virus X

VIRL_1334 Orangutan polyomavirus

VIRL_1693 Orchid fleck virus RNA 1

VIRL_1692 Orchid fleck virus RNA 2

VIRL_644 Orf virus

VIRL_1657 Orgyia leucostigma NPV

VIRL_1261 Orgyia pseudotsugata MNPV

VIRL_1175 Oropouche virus segment L

VIRL_2965 Oropouche virus segment M

VIRL_1130 Oropouche virus segment S

VIRL_3510 Oryctes rhinoceros virus

VIRL_2689 Oryza rufipogon endornavirus

VIRL_3617 Oryza sativa endornavirus

VIRL_3556 Ostreid herpesvirus 1

VIRL_786 Ostreococcus tauri virus 1

VIRL_1551 Ostreococcus virus OsV5

VIRL_933 Ourmia melon virus

VIRL_932 Ourmia melon virus

VIRL_931 Ourmia melon virus

VIRL_1358 Ovine adenovirus A

VIRL_2446 Ovine adenovirus A

VIRL_2425 Ovine adenovirus D

VIRL_1524 Ovine astrovirus

VIRL_2037 Ovine enzootic nasal tumour virus

VIRL_599 Ovine herpesvirus 2

VIRL_2483 Ovine lentivirus

VIRL_700 Ovine papillomavirus - 1

VIRL_411 Oyster mushroom spherical virus

VIRL_236 Panax virus Y

VIRL_2708 Panicum mosaic satellite virus

VIRL_2328 Panicum mosaic virus

VIRL_3436 Panicum streak virus - Karino

VIRL_369 Panine herpesvirus 2

VIRL_2970 Papaya leaf curl China virus - [G8]

VIRL_1704 Papaya leaf curl China virus satellite DNA beta

VIRL_2962 Papaya leaf curl Guandong virus - [GD2] DNA A

VIRL_3115 Papaya leaf curl virus

VIRL_2118 Papaya leaf curl virus-associated DNA beta

VIRL_3018 Papaya leaf-distortion mosaic potyvirus

VIRL_3612 Papaya mosaic virus

VIRL_3421 Papaya ringspot virus

VIRL_338 Papiine herpesvirus 2

VIRL_2168 Paprika mild mottle virus

VIRL_758 Parainfluenza virus 5

VIRL_1671 Paralichthys olivaceus birnavirus segment A

VIRL_1670 Paralichthys olivaceus birnavirus segment B

VIRL_1294 Paramecium bursaria Chlorella virus 1

VIRL_1672 Paramecium bursaria Chlorella virus AR158

VIRL_2839 Paramecium bursaria Chlorella virus FR483

VIRL_335 Paramecium bursaria Chlorella virus NY2A

VIRL_3592 Parana virus segment L

VIRL_1148 Parana virus segment S (small)

VIRL_1223 Pariacato virus chromosome RNA1

VIRL_1222 Pariacato virus chromosome RNA2

VIRL_2081 Parietaria mottle virus RNA 1

VIRL_207 Parietaria mottle virus RNA 2

VIRL_1570 Parietaria mottle virus RNA 3

VIRL_3227 Parsnip yellow fleck virus

VIRL_2502 Parvovirus H1

VIRL_1331 Passiflora latent carlavirus

VIRL_1410 Passionfruit severe leaf distortion virus DNA-A

VIRL_1409 Passionfruit severe leaf distortion virus DNA-B

VIRL_2553 Pasteurella phage F108

VIRL_2690 Patchouli mild mosaic virus RNA 1

VIRL_2187 Patchouli mild mosaic virus RNA 2

VIRL_2358 Pea early browning virus RNA 1

VIRL_3487 Pea early browning virus RNA 2

VIRL_3610 Pea enation mosaic virus satellite RNA

VIRL_2274 Pea enation mosaic virus-1

VIRL_3155 Pea enation mosaic virus-2

VIRL_3432 Pea seed-borne mosaic virus

VIRL_3500 Pea stem necrosis virus

VIRL_1675 Peach chlorotic mottle virus

VIRL_2704 Peach mosaic virus

VIRL_1275 Peanut chlorotic streak virus

VIRL_2251 Peanut clump virus RNA 1

VIRL_2255 Peanut clump virus RNA 2

VIRL_1518 Peanut mottle virus

VIRL_3386 Peanut stunt virus RNA 1

VIRL_3385 Peanut stunt virus RNA 2

VIRL_3384 Peanut stunt virus RNA 3

VIRL_3154 Peanut stunt virus satellite RNA

VIRL_1189 Pear latent virus

VIRL_1591 Pedilanthus leaf curl virus-Pedilanthus [Pakistan

VIRL_1422 Pelargonium chlorotic ring pattern virus

VIRL_2980 Pelargonium flower break virus

VIRL_2908 Pelargonium line pattern virus

VIRL_1392 Pelargonium necrotic spot virus

VIRL_105 Pelargonium vein banding virus

VIRL_1497 Pelargonium zonate spot virus RNA 1

VIRL_1496 Pelargonium zonate spot virus RNA 2

VIRL_1495 Pelargonium zonate spot virus RNA 3

VIRL_2866 Penaeid shrimp infectious myonecrosis virus

VIRL_900 Penaeus merguiensis densovirus

VIRL_833 Penaeus monodon hepatopancreatic parvovirus

VIRL_1158 Penicillium chrysogenum virus segment 1

VIRL_1157 Penicillium chrysogenum virus segment 2

VIRL_1156 Penicillium chrysogenum virus segment 3

VIRL_1155 Penicillium chrysogenum virus segment 4

VIRL_1082 Penicillium stoloniferum virus F segment 1

VIRL_1081 Penicillium stoloniferum virus F segment 2

VIRL_3683 Penicillium stoloniferum virus F segment 3

VIRL_435 Penicillium stoloniferum virus S segment 1

VIRL_1060 Penicillium stoloniferum virus S segment 2

VIRL_1084 Pennisetum mosaic virus

VIRL_1915 Penstemon ringspot virus

VIRL_412 Pepino mosaic virus

VIRL_880 Pepper curly top virus

VIRL_3120 Pepper golden mosaic virus DNA A

VIRL_1388 Pepper golden mosaic virus DNA B

VIRL_3490 Pepper huasteco yellow vein virus DNA A

VIRL_2499 Pepper huasteco yellow vein virus DNA B

VIRL_916 Pepper leaf curl Bangladesh virus segment A component

VIRL_3374 Pepper leaf curl virus DNA-A

VIRL_1663 Pepper leaf curl virus satellite DNA beta

VIRL_1643 Pepper leaf curl Yunnan virus satellite DNA beta

VIRL_1644 Pepper leaf curl Yunnan virus-[YN323]

VIRL_3226 Pepper mild mottle virus

VIRL_3454 Pepper mottle virus

VIRL_2254 Pepper ringspot virus RNA 1

VIRL_2253 Pepper ringspot virus RNA 2

VIRL_1905 Pepper severe mosaic virus

VIRL_2695 Pepper veinal mottle virus

VIRL_1298 Pepper yellow dwarf virus - New Mexico

VIRL_894 Pepper yellow leaf curl Indonesia virus DNA-A

VIRL_893 Pepper yellow leaf curl Indonesia virus DNA-B

VIRL_173 Pepper yellow mosaic virus

VIRL_2967 Pepper yellow vein Mali virus

VIRL_1238 Perina nuda virus

VIRL_2457 Periplaneta fuliginosa densovirus

VIRL_3056 Peru tomato mosaic virus

VIRL_1020 Peruvian horse sickness virus segment 1

VIRL_1011 Peruvian horse sickness virus segment 10

VIRL_1019 Peruvian horse sickness virus segment 2

VIRL_1018 Peruvian horse sickness virus segment 3

VIRL_1017 Peruvian horse sickness virus segment 4

VIRL_1013 Peruvian horse sickness virus segment 5

VIRL_1016 Peruvian horse sickness virus segment 6

VIRL_1012 Peruvian horse sickness virus segment 7

VIRL_1014 Peruvian horse sickness virus segment 8

VIRL_1015 Peruvian horse sickness virus segment 9

VIRL_2921 Peste-des-petits-ruminants virus

VIRL_2247 Pestivirus Giraffe-1

VIRL_1265 Petunia vein clearing virus

VIRL_1713 Phage cdtI

VIRL_3553 Phage Gifsy-1

VIRL_3552 Phage Gifsy-2

VIRL_2043 Phage phiJL001

VIRL_2696 Phaius virus X

VIRL_464 Phlebiopsis gigantea mycovirus dsRNA 1

VIRL_1668 Phlox Virus B

VIRL_1718 Phlox virus S

VIRL_678 Phocoena spinipinnis papillomavirus

VIRL_3528 Phormidium phage Pf-WMP3

VIRL_1909 Phormidium phage Pf-WMP4

VIRL_1204 Phthorimaea operculella granulovirus

VIRL_2271 Physalis mottle virus

VIRL_2026 Phytophthora endornavirus 1

VIRL_949 Phytophthora infestans RNA virus 1 RNA1

VIRL_948 Phytophthora infestans RNA virus 1 RNA2A

VIRL_1169 Pichinde virus

VIRL_1170 Pichinde virus L RNA

VIRL_804 Pieris rapae granulovirus

VIRL_1330 Pineapple mealybug wilt-associated virus 1

VIRL_1124 Pirital virus segment L

VIRL_2955 Pirital virus segment S

VIRL_1511 Planaria asexual strain-specific virus-like element type 1 large DNA segment

VIRL_1512 Planaria asexual strain-specific virus-like element type 1 small DNA segment

VIRL_915 Planococcus citri densovirus

VIRL_3158 Plantago asiatica mosaic virus

VIRL_3517 Plantago mottle virus

VIRL_3504 Plautia stali intestine virus

VIRL_986 Pleurotus ostreatus virus 1 RNA-1

VIRL_1087 Pleurotus ostreatus virus 1 RNA-2

VIRL_1667 Plum bark necrosis and stem pitting-associated virus

VIRL_3473 Plum pox virus

VIRL_1249 Plutella xylostella granulovirus

VIRL_3664 Plutella xylostella multiple nucleopolyhedrovirus

VIRL_973 Pneumonia virus of mice J3666

VIRL_3524 Poinsettia cryptic virus

VIRL_1529 Poinsettia mosaic virus

VIRL_3453 Poliovirus sp

VIRL_98 Polyomavirus HPyV6

VIRL_97 Polyomavirus HPyV7

VIRL_1131 Poplar mosaic virus

VIRL_2080 Porcine adenovirus A

VIRL_2440 Porcine adenovirus C

VIRL_1354 Porcine adenovirus C

VIRL_56 Porcine circovirus 1

VIRL_210 Porcine circovirus 2

VIRL_41 Porcine circovirus Canada-2010a

VIRL_374 Porcine endogenous retrovirus E

VIRL_1253 Porcine enteric sapovirus

VIRL_1210 Porcine enterovirus 8

VIRL_3062 Porcine enterovirus B

VIRL_3288 Porcine epidemic diarrhea virus

VIRL_1034 Porcine hemagglutinating encephalomyelitis virus

VIRL_829 Porcine kobuvirus swine/S-1-HUN/2007/Hungary

VIRL_3428 Porcine parvovirus

VIRL_313 Porcine respiratory and reproductive syndrome virus

VIRL_1690 Porcine rubulavirus

VIRL_3137 Porcine teschovirus 1

VIRL_1887 Possum enterovirus W1

VIRL_1886 Possum enterovirus W6

VIRL_1891 Potato apical leaf curl disease-associated satellite DNA beta

VIRL_3609 Potato aucuba mosaic virus

VIRL_1318 Potato latent virus

VIRL_1345 Potato leafroll virus

VIRL_2430 Potato mop-top virus RNA 1

VIRL_214 Potato mop-top virus RNA 2

VIRL_2429 Potato mop-top virus RNA 3

VIRL_2808 Potato rough dwarf virus

VIRL_3132 Potato virus A

VIRL_3489 Potato virus M

VIRL_2891 Potato virus S

VIRL_1603 Potato virus T

VIRL_1492 Potato virus V

VIRL_2743 Potato virus X

VIRL_3441 Potato virus Y

VIRL_2355 Potato yellow mosaic Panama virus DNA A

VIRL_2354 Potato yellow mosaic Panama virus DNA B

VIRL_2131 Potato yellow mosaic Trinidad virus DNA A

VIRL_2128 Potato yellow mosaic Trinidad virus DNA B

VIRL_3396 Potato yellow mosaic virus DNA A

VIRL_3395 Potato yellow mosaic virus DNA B

VIRL_2065 Potato yellow vein virus

VIRL_2930 Potato yellow vein virus

VIRL_2066 Potato yellow vein virus segment 3

VIRL_3373 Pothos latent virus

VIRL_3221 Powassan virus

VIRL_1368 Primula malacoides virus China/Mar2007 RNA 1

VIRL_1367 Primula malacoides virus China/Mar2007 RNA 2

VIRL_622 Prochlorococcus phage P-SSM2

VIRL_621 Prochlorococcus phage P-SSM4

VIRL_2570 Prochlorococcus phage P-SSP7

VIRL_605 Procyon lotor papillomavirus type 1

VIRL_1503 Propionibacterium phage B5

VIRL_1553 Propionibacterium phage PA6

VIRL_275 Providence virus

VIRL_1945 Prune dwarf virus

VIRL_1150 Prune dwarf virus chromosome RNA1

VIRL_1151 Prune dwarf virus RNA2

VIRL_1480 Prunus necrotic ringspot virus RNA 1

VIRL_1479 Prunus necrotic ringspot virus RNA 2

VIRL_3065 Prunus necrotic ringspot virus RNA 3

VIRL_808 Pseudaletia unipuncta granulovirus

VIRL_692 Pseudoalteromonas phage PM2

VIRL_438 Pseudocowpox virus

VIRL_1436 Pseudomonas phage 119X

VIRL_2729 Pseudomonas phage 14-1

VIRL_3638 Pseudomonas phage 201phi2-1

VIRL_1437 Pseudomonas phage 73

VIRL_2056 Pseudomonas phage B3

VIRL_1523 Pseudomonas phage D3

VIRL_2589 Pseudomonas phage D3112

VIRL_2543 Pseudomonas phage DMS3

VIRL_1976 Pseudomonas phage EL

VIRL_1438 Pseudomonas phage F10

VIRL_1449 Pseudomonas phage F116

VIRL_1433 Pseudomonas phage F8

VIRL_1477 Pseudomonas phage gh-1

VIRL_531 Pseudomonas phage LBL3

VIRL_979 Pseudomonas phage LIT1

VIRL_2526 Pseudomonas phage LKA1

VIRL_2527 Pseudomonas phage LKD16

VIRL_530 Pseudomonas phage LMA2

VIRL_2524 Pseudomonas phage LUZ19

VIRL_1655 Pseudomonas phage LUZ24

VIRL_980 Pseudomonas phage LUZ7

VIRL_1434 Pseudomonas phage M6

VIRL_2532 Pseudomonas phage MP22

VIRL_508 Pseudomonas phage MP29

VIRL_509 Pseudomonas phage MP38

VIRL_1435 Pseudomonas phage PA11

VIRL_2682 Pseudomonas phage PAJU2

VIRL_2078 Pseudomonas phage PaP2

VIRL_2142 Pseudomonas phage PaP3

VIRL_2716 Pseudomonas phage PB1

VIRL_2639 Pseudomonas phage Pf1

VIRL_735 Pseudomonas phage Pf3

VIRL_2606 Pseudomonas phage phi-12 segment L

VIRL_2605 Pseudomonas phage phi-12 segment M

VIRL_1571 Pseudomonas phage phi-12 segment S

VIRL_2607 Pseudomonas phage phi-13 segment M

VIRL_3630 Pseudomonas phage phi-13 segment S

VIRL_12 Pseudomonas phage phi-2

VIRL_2610 Pseudomonas phage phi-6 segment L

VIRL_3216 Pseudomonas phage phi-6 segment M

VIRL_3217 Pseudomonas phage phi-6 segment S

VIRL_2316 Pseudomonas phage phi-8 segment M

VIRL_1484 Pseudomonas phage phi13 segment L

VIRL_501 Pseudomonas phage phi2954 segment L

VIRL_1593 Pseudomonas phage phi2954 segment M

VIRL_1592 Pseudomonas phage phi2954 segment S

VIRL_2617 Pseudomonas phage phi8 segment L

VIRL_3591 Pseudomonas phage phi8 segment S

VIRL_680 Pseudomonas phage phiCTX

VIRL_497 Pseudomonas phage phikF77

VIRL_2590 Pseudomonas phage phiKMV

VIRL_2133 Pseudomonas phage phiKZ

VIRL_2633 Pseudomonas phage PP7

VIRL_2547 Pseudomonas phage PRR1

VIRL_2506 Pseudomonas phage PT2

VIRL_2507 Pseudomonas phage PT5

VIRL_506 Pseudomonas phage SN

VIRL_13 Pseudomonas phage YuA

VIRL_353 Psittacid herpesvirus 1

VIRL_671 Psittacus erithacus timneh papillomavirus

VIRL_846 Pumpkin yellow mosaic Malaysia virus DNA A

VIRL_1465 Puumala virus segment L

VIRL_2990 Puumala virus segment M

VIRL_2989 Puumala virus segment S

VIRL_639 Pyrobaculum spherical virus

VIRL_1694 Pyrococcus abyssi virus 1

VIRL_1420 Quang Binh virus

VIRL_2728 Rabbit calicivirus Australia 1 MIC-07

VIRL_690 Rabbit fibroma virus

VIRL_3451 Rabbit hemorrhagic disease virus-FRG

VIRL_687 Rabbit oral papillomavirus

VIRL_1893 Rabbit vesivirus

VIRL_3452 Rabies virus

VIRL_1195 Rachiplusia ou MNPV

VIRL_37 Radish leaf curl virus

VIRL_2790 Radish leaf curl virus satellite DNA beta

VIRL_866 Radish leaf curl virus segment A

VIRL_2766 Radish mosaic virus RNA1

VIRL_2765 Radish mosaic virus RNA2

VIRL_2094 Ralstonia phage p12J

VIRL_563 Ralstonia phage phiRSA1

VIRL_529 Ralstonia phage RSB1

VIRL_538 Ralstonia phage RSL1

VIRL_1894 Ralstonia phage RSM1

VIRL_835 Ralstonia phage RSM3

VIRL_2545 Ralstonia phage RSS1

VIRL_858 Ramie mosaic virus DNA-A

VIRL_857 Ramie mosaic virus DNA-B

VIRL_584 Ranid herpesvirus 1

VIRL_585 Ranid herpesvirus 2

VIRL_3662 Raphanus sativus cryptic virus 1 dsRNA 1

VIRL_2854 Raphanus sativus cryptic virus 1 dsRNA 2

VIRL_3657 Raphanus sativus cryptic virus 2 segment 1

VIRL_3656 Raphanus sativus cryptic virus 2 segment 2

VIRL_3655 Raphanus sativus cryptic virus 2 segment 3

VIRL_2727 Raphanus sativus cryptic virus 3 segment 1

VIRL_2726 Raphanus sativus cryptic virus 3 segment 2

VIRL_3608 Raspberry bushy dwarf virus RNA 1

VIRL_3204 Raspberry bushy dwarf virus RNA 2

VIRL_994 Raspberry leaf mottle virus

VIRL_2981 Raspberry ringspot virus RNA 2

VIRL_1132 Raspberry ringspot virus RNA1

VIRL_124 Rat coronavirus Parker

VIRL_471 Rattus norvegicus papillomavirus 1 EES-2009

VIRL_3413 Rauscher murine leukemia virus

VIRL_1906 Raven circovirus

VIRL_1678 RD114 retrovirus

VIRL_2231 Red clover mottle virus RNA 1

VIRL_2232 Red clover mottle virus RNA 2

VIRL_3196 Red clover necrotic mosaic virus RNA 1

VIRL_3183 Red clover necrotic mosaic virus RNA 2

VIRL_1362 Red clover vein mosaic virus

VIRL_2858 Redspotted grouper nervous necrosis virus RNA 1

VIRL_2857 Redspotted grouper nervous necrosis virus RNA 2

VIRL_1723 Rehmannia mosaic virus

VIRL_1316 Respiratory syncytial virus

VIRL_239 Reston ebolavirus

VIRL_1059 Reticuloendotheliosis virus

VIRL_706 Rhesus monkey papillomavirus 1

VIRL_787 Rhizobium phage 16-3

VIRL_365 Rhizoctonia solani virus 717 RNA1

VIRL_2213 Rhizoctonia solani virus RNA2

VIRL_45 Rhododendron virus A

VIRL_2416 Rhodothermus phage RM378

VIRL_1262 Rhopalosiphum padi virus

VIRL_864 Rhynchosia golden mosaic virus DNA A

VIRL_2651 Rhynchosia golden mosaic virus DNA B

VIRL_2673 Rhynchosia golden mosaic Yucatan virus DNA A

VIRL_2672 Rhynchosia golden mosaic Yucatan virus DNA B

VIRL_3344 Ribgrass mosaic virus

VIRL_3213 Rice black streaked dwarf virus segment 1

VIRL_3209 Rice black streaked dwarf virus segment 10

VIRL_3208 Rice black streaked dwarf virus segment 2

VIRL_3214 Rice black streaked dwarf virus segment 3

VIRL_3207 Rice black streaked dwarf virus segment 4

VIRL_3206 Rice black streaked dwarf virus segment 5

VIRL_3205 Rice black streaked dwarf virus segment 6

VIRL_3212 Rice black streaked dwarf virus segment 7

VIRL_3210 Rice black streaked dwarf virus segment 8

VIRL_3211 Rice black streaked dwarf virus segment 9

VIRL_3185 Rice dwarf virus segment 1

VIRL_3188 Rice dwarf virus segment 10

VIRL_3187 Rice dwarf virus segment 11

VIRL_311 Rice dwarf virus segment 12

VIRL_3184 Rice dwarf virus segment 2

VIRL_3186 Rice dwarf virus segment 3

VIRL_3193 Rice dwarf virus segment 4

VIRL_3192 Rice dwarf virus segment 5

VIRL_3191 Rice dwarf virus segment 6

VIRL_3194 Rice dwarf virus segment 7

VIRL_3190 Rice dwarf virus segment 8

VIRL_3189 Rice dwarf virus segment 9

VIRL_2823 Rice gall dwarf virus segment S1

VIRL_2828 Rice gall dwarf virus segment S10

VIRL_2825 Rice gall dwarf virus segment S11

VIRL_2819 Rice gall dwarf virus segment S12

VIRL_2826 Rice gall dwarf virus segment S2

VIRL_2827 Rice gall dwarf virus segment S3

VIRL_2822 Rice gall dwarf virus segment S4

VIRL_1719 Rice gall dwarf virus segment S5

VIRL_2821 Rice gall dwarf virus segment S6

VIRL_2820 Rice gall dwarf virus segment S7

VIRL_2829 Rice gall dwarf virus segment S8

VIRL_2824 Rice gall dwarf virus segment S9

VIRL_3368 Rice grassy stunt virus RNA 1

VIRL_3367 Rice grassy stunt virus RNA 2

VIRL_3366 Rice grassy stunt virus RNA 3

VIRL_3365 Rice grassy stunt virus RNA 4

VIRL_3364 Rice grassy stunt virus RNA 5

VIRL_3363 Rice grassy stunt virus RNA 6

VIRL_2228 Rice ragged stunt virus segment 1

VIRL_2223 Rice ragged stunt virus segment 10

VIRL_2227 Rice ragged stunt virus segment 2

VIRL_3200 Rice ragged stunt virus segment 3

VIRL_2221 Rice ragged stunt virus segment 4

VIRL_2224 Rice ragged stunt virus segment 5

VIRL_2226 Rice ragged stunt virus segment 6

VIRL_2222 Rice ragged stunt virus segment 7

VIRL_2225 Rice ragged stunt virus segment 8

VIRL_3195 Rice ragged stunt virus segment 9

VIRL_3197 Rice stripe virus RNA 1

VIRL_3198 Rice stripe virus RNA 2

VIRL_3182 Rice stripe virus RNA 3

VIRL_3199 Rice stripe virus RNA 4

VIRL_3405 Rice tungro bacilliform virus

VIRL_3439 Rice tungro spherical virus

VIRL_3445 Rice yellow mottle virus

VIRL_2312 Rice yellow mottle virus satellite

VIRL_2229 Rice yellow stunt virus

VIRL_152 Rift Valley fever virus segment L

VIRL_153 Rift Valley fever virus segment M

VIRL_154 Rift Valley fever virus segment S

VIRL_2924 Rinderpest virus (strain Kabete O)

VIRL_1574 Rio Bravo virus

VIRL_2782 Rose cryptic virus 1 RNA 1

VIRL_2781 Rose cryptic virus 1 RNA 2

VIRL_2780 Rose cryptic virus 1 RNA 3

VIRL_1623 Rose spring dwarf-associated virus

VIRL_1009 Rosellinia necatrix megabirnavirus 1/W779 segment L1

VIRL_1008 Rosellinia necatrix megabirnavirus 1/W779 segment L2

VIRL_2701 Rosellinia necatrix partitivirus 1-W8 segment 1

VIRL_2700 Rosellinia necatrix partitivirus 1-W8 segment 2

VIRL_1520 Roseobacter phage SIO1

VIRL_490 Roseophage DSS3P2

VIRL_491 Roseophage EE36P1

VIRL_1279 Ross River virus

VIRL_2956 Ross's goose hepatitis B virus

VIRL_2749 Rotavirus A segment 1

VIRL_2752 Rotavirus A segment 10

VIRL_2751 Rotavirus A segment 11

VIRL_2750 Rotavirus A segment 2

VIRL_2748 Rotavirus A segment 3

VIRL_2746 Rotavirus A segment 4

VIRL_2756 Rotavirus A segment 5

VIRL_2747 Rotavirus A segment 6

VIRL_2754 Rotavirus A segment 7

VIRL_2755 Rotavirus A segment 8

VIRL_2753 Rotavirus A segment 9

VIRL_2885 Rotavirus C segment 1

VIRL_2882 Rotavirus C segment 10

VIRL_2878 Rotavirus C segment 11

VIRL_1399 Rotavirus C segment 2

VIRL_2879 Rotavirus C segment 3

VIRL_2877 Rotavirus C segment 4

VIRL_2881 Rotavirus C segment 5

VIRL_1400 Rotavirus C segment 6

VIRL_2887 Rotavirus C segment 7

VIRL_2880 Rotavirus C segment 8

VIRL_2886 Rotavirus C segment 9

VIRL_26 Rotavirus D chicken/05V0049/DEU/2005 segment 1

VIRL_17 Rotavirus D chicken/05V0049/DEU/2005 segment 10

VIRL_16 Rotavirus D chicken/05V0049/DEU/2005 segment 11

VIRL_25 Rotavirus D chicken/05V0049/DEU/2005 segment 2

VIRL_24 Rotavirus D chicken/05V0049/DEU/2005 segment 3

VIRL_23 Rotavirus D chicken/05V0049/DEU/2005 segment 4

VIRL_22 Rotavirus D chicken/05V0049/DEU/2005 segment 5

VIRL_21 Rotavirus D chicken/05V0049/DEU/2005 segment 6

VIRL_20 Rotavirus D chicken/05V0049/DEU/2005 segment 7

VIRL_19 Rotavirus D chicken/05V0049/DEU/2005 segment 8

VIRL_18 Rotavirus D chicken/05V0049/DEU/2005 segment 9

VIRL_3485 Rous sarcoma virus

VIRL_580 Rousettus aegyptiacus papillomavirus type 1

VIRL_3450 Rubella virus

VIRL_2720 Rubus chlorotic mottle virus

VIRL_2694 Rudbeckia flower distortion virus

VIRL_3390 Rupestris stem pitting associated virus-1

VIRL_3416 Ryegrass mosaic virus

VIRL_3201 Ryegrass mottle virus

VIRL_1172 Sabia virus

VIRL_1106 Sabia virus segment L

VIRL_385 Sacbrood virus

VIRL_1487 Saccharomyces 20S RNA narnavirus

VIRL_1205 Saccharomyces 23S RNA narnavirus

VIRL_1267 Saccharomyces cerevisiae killer virus M1

VIRL_1217 Saccharomyces cerevisiae virus L-A (L1)

VIRL_1274 Saccharomyces cerevisiae virus L-BC (La)

VIRL_812 Saccharum streak virus

VIRL_1717 Saffold virus

VIRL_1535 Saguaro cactus virus

VIRL_742 Saimiriine herpesvirus 2

VIRL_110 Salivirus NG-J1

VIRL_2188 Salmon pancreas disease virus

VIRL_504 Salmonella enterica bacteriophage SE1

VIRL_475 Salmonella phage c341

VIRL_3544 Salmonella phage E1

VIRL_502 Salmonella phage epsilon34

VIRL_3640 Salmonella phage Fels-1

VIRL_1385 Salmonella phage KS5

VIRL_2519 Salmonella phage phiSG-JL2

VIRL_1720 Salmonella phage SETP3

VIRL_2714 Salmonella phage ST64B

VIRL_2917 Sapovirus C12 strain C12

VIRL_2927 Sapovirus Hu/Dresden/pJG-Sap01/DE

VIRL_2957 Sapovirus Mc10

VIRL_1003 SARS coronavirus

VIRL_768 SARS coronavirus Rs_672/2006

VIRL_2273 Satellite virus of maize white line mosaic virus

VIRL_1312 Satellites of Trichomonas vaginalis T1 virus

VIRL_3607 Satsuma dwarf virus RNA 1

VIRL_3636 Satsuma dwarf virus RNA 2

VIRL_2310 Scallion mosaic virus

VIRL_2309 Scallion virus X

VIRL_3603 Schizochytrium single-stranded RNA virus

VIRL_2731 Schlumbergera virus X

VIRL_2084 Sclerophthora macrospora virus A RNA 1

VIRL_2083 Sclerophthora macrospora virus A RNA 2

VIRL_2082 Sclerophthora macrospora virus A RNA 3

VIRL_2117 Sclerophthora macrospora virus B

VIRL_3678 Sclerotinia sclerotiorum debilitation-associated RNA virus

VIRL_259 Sclerotinia sclerotiorum hypovirulence associated DNA virus 1

VIRL_946 Sclerotinia sclerotiorum partitivirus S segment 1

VIRL_945 Sclerotinia sclerotiorum partitivirus S segment 2

VIRL_1030 Scotophilus bat coronavirus 512

VIRL_3519 Scrophularia mottle virus

VIRL_828 Sea turtle tornovirus 1

VIRL_1676 Seal picornavirus type 1

VIRL_3315 Semliki forest virus

VIRL_315 Sendai virus

VIRL_3557 Seneca valley virus

VIRL_2041 Senecio yellow mosaic virus

VIRL_2983 Seoul virus segment M

VIRL_2984 Seoul virus strain 80-39 segment S

VIRL_2982 Seoul virus strain Seoul 80-39 clone 1

VIRL_2837 Sepik virus

VIRL_2329 Sesbania mosaic virus

VIRL_2215 Shallot virus X

VIRL_2890 Shallot yellow stripe virus

VIRL_670 Sheeppox virus 17077-99

VIRL_1125 Sheldgoose hepatitis B virus

VIRL_978 Shigella phage phiSboM-AG3

VIRL_1237 Shrimp white spot syndrome virus

VIRL_2420 Sida golden mosaic Costa Rica virus DNA A

VIRL_2419 Sida golden mosaic Costa Rica virus DNA B

VIRL_2132 Sida golden mosaic Florida virus

VIRL_75 Sida golden mosaic Florida virus-Malvastrum DNA-A

VIRL_74 Sida golden mosaic Florida virus-Malvastrum DNA-B

VIRL_2124 Sida golden mosaic Honduras virus DNA A

VIRL_2123 Sida golden mosaic Honduras virus DNA B

VIRL_2356 Sida golden mosaic virus DNA-A

VIRL_3381 Sida golden mosaic virus DNA-B

VIRL_272 Sida golden mottle virus DNA-A

VIRL_273 Sida golden mottle virus DNA-B

VIRL_3040 Sida golden yellow vein virus

VIRL_2874 Sida leaf curl virus

VIRL_1702 Sida leaf curl virus satellite DNA beta

VIRL_2872 Sida leaf curl virus-associated DNA 1

VIRL_2873 Sida leaf curl virus-associated DNA beta

VIRL_2969 Sida micrantha mosaic virus segment A

VIRL_2968 Sida micrantha mosaic virus segment B

VIRL_1942 Sida mosaic Sinaloa virus DNA A

VIRL_1944 Sida mosaic Sinaloa virus DNA B

VIRL_1478 Sida mottle virus

VIRL_3039 Sida yellow mosaic virus

VIRL_2928 Sida yellow mosaic virus-[China]-associated DNA beta DNA beta

VIRL_1874 Sida yellow mosaic Yucatan virus DNA A

VIRL_1873 Sida yellow mosaic Yucatan virus DNA B

VIRL_332 Sida yellow vein disease associated DNA 1

VIRL_882 Sida yellow vein Madurai virus

VIRL_877 Sida yellow vein Vietnam virus DNA-A

VIRL_1701 Sida yellow vein Vietnam virus satellite DNA beta

VIRL_1697 Sida yellow vein Vietnam virus-associated DNA 1

VIRL_2122 Sida yellow vein virus DNA A

VIRL_3032 Sida yellow vein virus DNA B

VIRL_2021 Sida yellow vein virus-associated DNA beta

VIRL_890 Siegesbeckia yellow vein Guangxi virus

VIRL_2853 Siegesbeckia yellow vein virus-[GD13]

VIRL_2852 Siegesbeckia yellow vein virus-[GD13]-associated DNA beta

VIRL_150 Simian adenovirus 1

VIRL_3308 Simian adenovirus 21

VIRL_3307 Simian adenovirus 25

VIRL_1310 Simian adenovirus 3

VIRL_601 Simian agent 12

VIRL_2185 Simian enterovirus A

VIRL_1549 Simian enterovirus SV19

VIRL_1548 Simian enterovirus SV43

VIRL_1547 Simian enterovirus SV6

VIRL_2501 Simian foamy virus

VIRL_1620 Simian foamy virus 3

VIRL_2439 Simian hemorrhagic fever virus

VIRL_2482 Simian immunodeficiency virus

VIRL_1191 Simian immunodeficiency virus SIV-mnd 2

VIRL_1192 Simian picornavirus 1

VIRL_1431 Simian picornavirus 17

VIRL_755 Simian picornavirus strain N125

VIRL_754 Simian picornavirus strain N203

VIRL_47 Simian retrovirus 4

VIRL_130 Simian T-cell lymphotropic virus 6

VIRL_763 Simian T-lymphotropic virus 1

VIRL_3415 Simian T-lymphotropic virus 2

VIRL_1235 Simian T-lymphotropic virus 3

VIRL_500 Simian virus 12

VIRL_255 Simian virus 40

VIRL_2060 Simian virus 41

VIRL_216 Simian-Human immunodeficiency virus

VIRL_1178 Sin Nombre virus

VIRL_1180 Sin Nombre virus chromosome M segment

VIRL_1179 Sin Nombre virus chromosome S segment

VIRL_1278 Sindbis virus

VIRL_1099 Singapore grouper iridovirus

VIRL_1903 Siniperca chuatsi rhabdovirus

VIRL_1509 Sinorhizobium phage PBC5

VIRL_1506 Sleeping disease virus

VIRL_32 Slow bee paralysis virus

VIRL_3675 Small anellovirus 1

VIRL_2653 Small anellovirus 2

VIRL_1669 Snake adenovirus

VIRL_350 Snake parvovirus 1

VIRL_1147 Snakehead retrovirus

VIRL_1254 Snakehead rhabdovirus

VIRL_2952 Snow goose hepatitis B virus

VIRL_2556 Sodalis phage phiSG1

VIRL_100 Sodalis phage SO-1

VIRL_1416 Soft-shelled turtle iridovirus

VIRL_2449 Soil-borne cereal mosaic virus RNA1

VIRL_3362 Soil-borne cereal mosaic virus RNA2

VIRL_3383 Soil-borne wheat mosaic virus RNA1

VIRL_3382 Soil-borne wheat mosaic virus RNA2

VIRL_2198 Solanum nodiflorum mottle virus satellite RNA

VIRL_3604 Solenopsis invicta virus 1

VIRL_1706 Solenopsis invicta virus 2

VIRL_1360 Solenopsis invicta virus 3

VIRL_2381 Sonchus yellow net virus

VIRL_2178 Sorghum chlorotic spot virus RNA 1

VIRL_2426 Sorghum chlorotic spot virus RNA 2

VIRL_3133 Sorghum mosaic virus

VIRL_3175 South African cassava mosaic virus DNA A

VIRL_2212 South African cassava mosaic virus DNA B

VIRL_3129 Southern bean mosaic virus

VIRL_2380 Southern cowpea mosaic virus

VIRL_794 Southern tomato virus

VIRL_267 Soybean chlorotic blotch virus DNA A

VIRL_266 Soybean chlorotic blotch virus DNA B

VIRL_3424 Soybean chlorotic mottle virus

VIRL_2313 Soybean crinkle leaf virus

VIRL_3316 Soybean dwarf virus

VIRL_268 Soybean mild mottle virus

VIRL_312 Soybean mosaic virus

VIRL_1321 Soybean yellow mottle mosaic virus

VIRL_1257 Sphaeropsis sapinea RNA virus 1

VIRL_1256 Sphaeropsis sapinea RNA virus 2

VIRL_879 Spilanthes yellow vein virus DNA-A

VIRL_230 Spinach curly top virus

VIRL_2210 Spinach latent virus RNA 1

VIRL_213 Spinach latent virus RNA 2

VIRL_2209 Spinach latent virus RNA 3

VIRL_867 Spiroplasma kunkelii virus SkV1_CR2-3x

VIRL_2216 Spiroplasma phage 1-C74

VIRL_3536 Spiroplasma phage 1-R8A2B

VIRL_674 Spiroplasma phage 4

VIRL_3370 Spiroplasma phage SVTS2

VIRL_169 Spissistilus festinus virus 1

VIRL_2488 Spleen focus-forming virus

VIRL_384 Spodoptera exigua MNPV

VIRL_1322 Spodoptera frugiperda ascovirus 1a

VIRL_420 Spodoptera frugiperda MNPV virus

VIRL_966 Spodoptera litura granulovirus

VIRL_1239 Spodoptera litura NPV

VIRL_3494 Spodoptera litura nucleopolyhedrovirus II

VIRL_2166 Spring beauty latent virus RNA 1

VIRL_2165 Spring beauty latent virus RNA 2

VIRL_2164 Spring beauty latent virus RNA 3

VIRL_234 Spring viraemia of carp virus

VIRL_1601 Sputnik virophage

VIRL_139 Squash leaf curl China virus - [B] DNA B

VIRL_171 Squash leaf curl China virus - [B] DNA-A

VIRL_907 Squash leaf curl Philippines virus segment A

VIRL_906 Squash leaf curl Philippines virus segment B

VIRL_35 Squash leaf curl virus A component DNA

VIRL_34 Squash leaf curl virus B component DNA

VIRL_2125 Squash leaf curl Yunnan virus

VIRL_3037 Squash mild leaf curl virus-[Imperial Valley] DNA A

VIRL_3036 Squash mild leaf curl virus-[Imperial Valley] DNA B

VIRL_3177 Squash mosaic virus RNA 1

VIRL_3176 Squash mosaic virus RNA 2

VIRL_1541 Squash vein yellowing virus

VIRL_2196 Squash yellow mild mottle virus DNA B

VIRL_555 Squirrel monkey polyomavirus

VIRL_57 Squirrel monkey retrovirus - HLB

VIRL_3152 Sri Lankan cassava mosaic virus DNA A

VIRL_3151 Sri Lankan cassava mosaic virus DNA B

VIRL_320 St Croix River virus chromosome segment 10

VIRL_988 St Croix River virus chromosome segment 2

VIRL_352 St Croix River virus chromosome segment 3

VIRL_326 St Croix River virus chromosome segment 4

VIRL_325 St Croix River virus chromosome segment 5

VIRL_324 St Croix River virus chromosome segment 6

VIRL_323 St Croix River virus chromosome segment 7

VIRL_322 St Croix River virus chromosome segment 8

VIRL_321 St Croix River virus chromosome segment 9

VIRL_1173 St Croix River virus segment 1

VIRL_2095 St. Augustine decline satellite virus

VIRL_2876 St. Louis encephalitis virus

VIRL_3125 Stachytarpheta leaf curl virus

VIRL_456 Staphylococcus phage 11

VIRL_985 Staphylococcus phage 187

VIRL_2032 Staphylococcus phage 2638A

VIRL_2901 Staphylococcus phage 29

VIRL_3666 Staphylococcus phage 37

VIRL_2030 Staphylococcus phage 3A

VIRL_2031 Staphylococcus phage 42e

VIRL_2600 Staphylococcus phage 44AHJD

VIRL_2904 Staphylococcus phage 47

VIRL_2900 Staphylococcus phage 52A

VIRL_2906 Staphylococcus phage 53

VIRL_2027 Staphylococcus phage 55

VIRL_2566 Staphylococcus phage 66

VIRL_2907 Staphylococcus phage 69

VIRL_2902 Staphylococcus phage 71

VIRL_1459 Staphylococcus phage 77

VIRL_1554 Staphylococcus phage 80alpha

VIRL_2905 Staphylococcus phage 85

VIRL_2899 Staphylococcus phage 88

VIRL_2898 Staphylococcus phage 92

VIRL_2903 Staphylococcus phage 96

VIRL_1885 Staphylococcus phage CNPH82

VIRL_2029 Staphylococcus phage EW

VIRL_2565 Staphylococcus phage G1

VIRL_1456 Staphylococcus phage K

VIRL_807 Staphylococcus phage P954

VIRL_1884 Staphylococcus phage PH15

VIRL_2677 Staphylococcus phage phi2958PVL

VIRL_2691 Staphylococcus phage phiETA

VIRL_3497 Staphylococcus phage phiETA2

VIRL_3496 Staphylococcus phage phiETA3

VIRL_3495 Staphylococcus phage phiMR11

VIRL_459 Staphylococcus phage phiMR25

VIRL_2840 Staphylococcus phage phiNM1

VIRL_749 Staphylococcus phage phiNM3

VIRL_2599 Staphylococcus phage phiP68

VIRL_824 Staphylococcus phage phiPVL-CN125

VIRL_1889 Staphylococcus phage phiPVL108

VIRL_1002 Staphylococcus phage phiSauS-IPLA35

VIRL_1001 Staphylococcus phage phiSauS-IPLA88

VIRL_2444 Staphylococcus phage phiSLT

VIRL_1568 Staphylococcus phage PT1028

VIRL_2028 Staphylococcus phage ROSA

VIRL_2529 Staphylococcus phage SAP-2

VIRL_54 Staphylococcus phage SAP-26

VIRL_2567 Staphylococcus phage Twort

VIRL_2897 Staphylococcus phage X2

VIRL_2136 Staphylococcus prophage phi 12

VIRL_2135 Staphylococcus prophage phi 13

VIRL_2679 Staphylococcus prophage phiN315

VIRL_2447 Staphylococcus prophage phiPV83

VIRL_2692 Staphylococcus prophage PVL

VIRL_1683 Staphylococcus prophage tp310-1

VIRL_1682 Staphylococcus prophage tp310-2

VIRL_1681 Staphylococcus prophage tp310-3

VIRL_3673 Starling circovirus

VIRL_2647 Steller sea lion vesivirus

VIRL_2024 Stenotrophomonas phage phiSMA9

VIRL_510 Stenotrophomonas phage S1

VIRL_1910 Strawberry chlorotic fleck associated virus

VIRL_2042 Strawberry latent ringspot virus RNA1

VIRL_2909 Strawberry latent ringspot virus RNA2

VIRL_3159 Strawberry latent ringspot virus satellite RNA

VIRL_3179 Strawberry mild yellow edge virus

VIRL_1505 Strawberry mottle virus RNA 1

VIRL_1504 Strawberry mottle virus RNA 2

VIRL_461 Strawberry necrotic shock virus RNA 1

VIRL_462 Strawberry necrotic shock virus RNA 2

VIRL_463 Strawberry necrotic shock virus RNA 3

VIRL_1323 Strawberry pallidosis-associated virus RNA 1

VIRL_947 Strawberry pallidosis-associated virus RNA 2

VIRL_2375 Strawberry vein banding virus

VIRL_1911 Streptocarpus flower break virus

VIRL_2036 Streptococcus phage 2972

VIRL_1412 Streptococcus phage 5093

VIRL_2454 Streptococcus phage 7201

VIRL_1550 Streptococcus phage 858

VIRL_957 Streptococcus phage Abc2

VIRL_958 Streptococcus phage ALQ13.2

VIRL_2593 Streptococcus phage C1

VIRL_2631 Streptococcus phage Cp-1

VIRL_2463 Streptococcus phage DT1

VIRL_1397 Streptococcus phage M102

VIRL_1482 Streptococcus phage O1205

VIRL_2531 Streptococcus phage P9

VIRL_1411 Streptococcus phage PH10

VIRL_3554 Streptococcus phage PH15

VIRL_1725 Streptococcus phage phi3396

VIRL_2451 Streptococcus phage Sfi11

VIRL_2462 Streptococcus phage Sfi19

VIRL_2461 Streptococcus phage Sfi21

VIRL_1467 Streptococcus phage SM1

VIRL_992 Streptococcus phage SMP

VIRL_1396 Streptococcus prophage 315.1

VIRL_1349 Streptococcus prophage 315.2

VIRL_1348 Streptococcus prophage 315.4

VIRL_1347 Streptococcus prophage 315.5

VIRL_1346 Streptococcus prophage 315.6

VIRL_2086 Streptococcus prophage EJ-1

VIRL_3318 Streptococcus prophage MM1

VIRL_436 Streptococcus pyogenes phage 315.3

VIRL_1032 Streptomyces phage mu1/6

VIRL_2121 Streptomyces phage phiBT1

VIRL_2468 Streptomyces phage phiC31

VIRL_250 Streptomyces phage phiSASD1

VIRL_2713 Streptomyces phage VWB

VIRL_1370 Stretch Lagoon orbivirus segment 1

VIRL_1369 Stretch Lagoon orbivirus segment 2

VIRL_3286 Striped Jack nervous necrosis virus

VIRL_3287 Striped Jack nervous necrosis virus RNA1

VIRL_151 Stx1 converting phage

VIRL_2611 Stx2 converting phage I

VIRL_163 Stx2 converting phage II

VIRL_516 Stx2-converting phage 1717

VIRL_2546 Stx2-converting phage 86

VIRL_3068 Subterranean clover mottle virus

VIRL_3157 Subterranean clover mottle virus satellite RNA

VIRL_2207 Subterranean clover stunt virus DNA 2

VIRL_2208 Subterranean clover stunt virus DNA 1

VIRL_2206 Subterranean clover stunt virus DNA 3

VIRL_2205 Subterranean clover stunt virus DNA 4

VIRL_3171 Subterranean clover stunt virus DNA 5

VIRL_2204 Subterranean clover stunt virus DNA 6

VIRL_2203 Subterranean clover stunt virus DNA 7

VIRL_1494 Subterranean clover stunt virus DNA 8

VIRL_2684 Sudan ebolavirus

VIRL_2320 Sugarcane bacilliform IM virus

VIRL_1948 Sugarcane bacilliform Mor virus

VIRL_155 Sugarcane bacilliform virus

VIRL_3295 Sugarcane mosaic virus

VIRL_3409 Sugarcane streak Egypt virus - [Giza]

VIRL_409 Sugarcane streak mosaic virus

VIRL_911 Sugarcane streak Reunion virus

VIRL_3202 Sugarcane streak virus - [Natal]

VIRL_989 Sugarcane striate mosaic-associated virus

VIRL_3375 Sugarcane yellow leaf virus

VIRL_349 Suid herpesvirus 1

VIRL_681 Sulfolobus islandicus filamentous virus

VIRL_665 Sulfolobus islandicus rod-shaped virus 1

VIRL_666 Sulfolobus islandicus rod-shaped virus 2

VIRL_553 Sulfolobus spindle-shaped virus 4

VIRL_528 Sulfolobus spindle-shaped virus 5

VIRL_178 Sulfolobus spindle-shaped virus 6

VIRL_177 Sulfolobus spindle-shaped virus 7

VIRL_228 Sulfolobus turreted icosahedral virus

VIRL_280 Sulfolobus turreted icosahedral virus 2

VIRL_2503 Sulfolobus virus 1

VIRL_1463 Sulfolobus virus 2

VIRL_2583 Sulfolobus virus Kamchatka 1

VIRL_641 Sulfolobus virus Ragged Hills

VIRL_637 Sulfolobus virus STSV1

VIRL_199 Sunflower chlorotic mottle virus

VIRL_1390 Sunn hemp leaf distortion virus DNA-A

VIRL_526 Sus scrofa papillomavirus type 1

VIRL_2211 Sweet clover necrotic mosaic virus RNA 1

VIRL_3173 Sweet clover necrotic mosaic virus RNA 2

VIRL_974 Sweet potato chlorotic fleck virus

VIRL_1485 Sweet potato chlorotic stunt virus RNA 1

VIRL_2163 Sweet potato chlorotic stunt virus RNA 2

VIRL_2367 Sweet potato feathery mottle virus

VIRL_458 Sweet potato leaf curl Bengal virus - [India

VIRL_811 Sweet potato leaf curl Canary virus

VIRL_2130 Sweet potato leaf curl Georgia virus

VIRL_810 Sweet potato leaf curl Lanzarote virus

VIRL_841 Sweet potato leaf curl Spain virus

VIRL_3035 Sweet potato leaf curl virus

VIRL_2214 Sweet potato mild mottle virus

VIRL_1398 Sweetpotato badnavirus B

VIRL_677 Swinepox virus

VIRL_2613 Synechococcus phage P60

VIRL_623 Synechococcus phage S-PM2

VIRL_474 Synechococcus phage S-RSM4

VIRL_582 Synechococcus phage syn9

VIRL_2153 Tacaribe virus segment L

VIRL_2152 Tacaribe virus segment S

VIRL_3135 Tamana bat virus

VIRL_2772 Tamiami virus segment L

VIRL_1149 Tamiami virus segment S (small)

VIRL_556 Tanapox virus

VIRL_2706 Taro bacilliform virus

VIRL_1308 Taro vein chlorosis virus

VIRL_583 Taterapox virus

VIRL_327 Taura syndrome virus

VIRL_431 Telosma mosaic virus

VIRL_1580 Temperate phage phiNIH1.1

VIRL_552 Thalassomonas phage BA3

VIRL_316 Theilovirus sp

VIRL_636 Thermoproteus tenax spherical virus 1

VIRL_1350 Thermus phage IN93

VIRL_2536 Thermus phage P23-45

VIRL_744 Thermus phage P23-77

VIRL_2535 Thermus phage P74-26

VIRL_576 Thermus phage phiYS40

VIRL_201 Thielaviopsis basicola mitovirus

VIRL_1167 Thogoto virus

VIRL_451 Thogoto virus chromosome segment 2

VIRL_450 Thogoto virus chromosome segment 3

VIRL_2057 Thogoto virus segment 1

VIRL_2058 Thogoto virus segment 5

VIRL_1168 Thogoto virus segment 6

VIRL_2768 Thottapalayam virus segment L

VIRL_2767 Thottapalayam virus segment M

VIRL_1633 Thottapalayam virus segment S

VIRL_2719 Thrush coronavirus HKU12-600

VIRL_2895 Thunberg fritillary virus

VIRL_3431 Tick-borne encephalitis virus

VIRL_410 Tiger puffer nervous necrosis virus RNA 1

VIRL_1329 Tiger puffer nervous necrosis virus RNA2

VIRL_1202 Tioman virus

VIRL_1193 Tobacco bushy top virus

VIRL_269 Tobacco bushy top virus satellite-like RNA

VIRL_3215 Tobacco curly shoot virus

VIRL_3008 Tobacco curly shoot virus associated DNA 1

VIRL_3058 Tobacco curly shoot virus-associated DNA beta

VIRL_3448 Tobacco etch virus

VIRL_3011 Tobacco leaf curl disease associated sequence virion

VIRL_3034 Tobacco leaf curl Japan virus

VIRL_2129 Tobacco leaf curl Kochi virus

VIRL_1705 Tobacco leaf curl Thailand virus

VIRL_44 Tobacco leaf curl virus

VIRL_803 Tobacco leaf curl virus-associated DNA beta

VIRL_3066 Tobacco leaf curl Yunnan virus - [Y136]

VIRL_3005 Tobacco leaf curl Yunnan virus associated DNA 1

VIRL_3016 Tobacco leaf curl Yunnan virus satellite DNA beta

VIRL_375 Tobacco leaf curl Zimbabwe virus

VIRL_1276 Tobacco mild green mosaic virus

VIRL_2500 Tobacco mosaic virus

VIRL_2383 Tobacco necrosis satellite virus

VIRL_217 Tobacco necrosis virus A

VIRL_370 Tobacco necrosis virus D

VIRL_3174 Tobacco rattle virus RNA 1

VIRL_3172 Tobacco rattle virus RNA 2

VIRL_2096 Tobacco ringspot virus RNA 1

VIRL_2097 Tobacco ringspot virus RNA 2

VIRL_2191 Tobacco ringspot virus satellite RNA

VIRL_3161 Tobacco streak virus RNA 1

VIRL_3162 Tobacco streak virus RNA 2

VIRL_3160 Tobacco streak virus RNA 3

VIRL_1666 Tobacco vein banding mosaic virus

VIRL_371 Tobacco vein clearing virus

VIRL_1632 Tobacco vein distorting virus

VIRL_3422 Tobacco vein mottling virus

VIRL_834 Tobacco yellow crinkle virus-[Capsicum

VIRL_2202 Tobacco yellow dwarf virus

VIRL_3163 Tomato aspermy virus RNA 1

VIRL_2428 Tomato aspermy virus RNA 2

VIRL_3164 Tomato aspermy virus RNA 3

VIRL_1468 Tomato begomovirus satellite DNA beta

VIRL_2144 Tomato black ring virus RNA 1

VIRL_3063 Tomato black ring virus RNA 2

VIRL_3145 Tomato black ring virus satellite RNA

VIRL_1277 Tomato bushy stunt virus

VIRL_3168 Tomato bushy stunt virus satellite RNA B1

VIRL_1128 Tomato chino La Paz virus segment A

VIRL_1048 Tomato chlorosis virus RNA 1

VIRL_1047 Tomato chlorosis virus RNA 2

VIRL_3224 Tomato chlorotic mottle virus DNA A

VIRL_3223 Tomato chlorotic mottle virus DNA B

VIRL_43 Tomato chocolate spot virus RNA1

VIRL_42 Tomato chocolate spot virus RNA2

VIRL_850 Tomato common mosaic virus DNA-A

VIRL_849 Tomato common mosaic virus DNA-B

VIRL_1476 Tomato curly stunt virus

VIRL_2485 Tomato golden mosaic virus DNA A

VIRL_2484 Tomato golden mosaic virus DNA B

VIRL_2856 Tomato golden mottle virus DNA A

VIRL_1943 Tomato golden mottle virus DNA B

VIRL_192 Tomato infectious chlorosis virus RNA 1

VIRL_191 Tomato infectious chlorosis virus RNA 2

VIRL_885 Tomato leaf curl Arusha virus DNA-A

VIRL_3144 Tomato leaf curl Bangalore virus

VIRL_1665 Tomato leaf curl Bangalore virus-[Ban5] satellite DNA beta

VIRL_2137 Tomato leaf curl Bangladesh virus

VIRL_997 Tomato leaf curl Cameroon virus - [Cameroon

VIRL_860 Tomato leaf curl Cebu virus DNA-A

VIRL_2971 Tomato leaf curl China virus - [G32]

VIRL_2958 Tomato leaf curl China virus satellite DNA beta

VIRL_95 Tomato leaf curl Cotabato virus DNA-A

VIRL_55 Tomato leaf curl Ghana virus segment A

VIRL_1908 Tomato leaf curl Guangdong virus DNA-A

VIRL_889 Tomato leaf curl Guangxi virus

VIRL_3057 Tomato leaf curl Gujarat virus - [Varanasi] segment A

VIRL_2139 Tomato leaf curl Gujarat virus - [Varanasi] segment B

VIRL_1372 Tomato leaf curl Hainan virus

VIRL_888 Tomato leaf curl Hsinchu virus - [Taiwan

VIRL_908 Tomato leaf curl Iran virus

VIRL_1387 Tomato leaf curl Java virus

VIRL_3605 Tomato leaf curl Java virus-[Ageratum] satellite DNA

VIRL_2791 Tomato leaf curl Joydebpur beta virus

VIRL_2870 Tomato leaf curl Joydebpur virus DNA-A

VIRL_3142 Tomato leaf curl Karnataka virus

VIRL_1898 Tomato leaf curl Karnataka virus-associated DNA beta DNA-A

VIRL_839 Tomato leaf curl Kerala virus

VIRL_5 Tomato leaf curl Kumasi virus segment A

VIRL_2138 Tomato leaf curl Laos virus

VIRL_2913 Tomato leaf curl Madagascar virus

VIRL_2126 Tomato leaf curl Malaysia virus

VIRL_231 Tomato leaf curl Mali virus

VIRL_2912 Tomato leaf curl Mayotte virus

VIRL_859 Tomato leaf curl Mindanao virus DNA-A

VIRL_3048 Tomato leaf curl New Delhi virus DNA A

VIRL_3047 Tomato leaf curl New Delhi virus DNA B

VIRL_2966 Tomato leaf curl New Delhi virus-associated DNA beta

VIRL_1585 Tomato leaf curl Nigeria virus-[Nigeria

VIRL_1 Tomato leaf curl Oman virus

VIRL_1408 Tomato leaf curl Pakistan virus associated DNA 1

VIRL_892 Tomato leaf curl Pakistan virus segment A

VIRL_1619 Tomato leaf curl Palampur virus

VIRL_1618 Tomato leaf curl Palampur virus

VIRL_4 Tomato leaf curl Patna virus DNA-A

VIRL_3 Tomato leaf curl Patna virus satellite DNA beta

VIRL_2705 Tomato leaf curl Philippine virus satellite DNA beta

VIRL_3015 Tomato leaf curl Philippines virus

VIRL_1901 Tomato leaf curl Pune virus

VIRL_884 Tomato leaf curl Seychelles virus

VIRL_872 Tomato leaf curl Sinaloa virus DNA A

VIRL_873 Tomato leaf curl Sinaloa virus DNA B

VIRL_2127 Tomato leaf curl Sri Lanka virus

VIRL_2961 Tomato leaf curl Sudan virus - [Gezira]

VIRL_2 Tomato leaf curl Sulawesi virus DNA-A

VIRL_3141 Tomato leaf curl Taiwan virus

VIRL_1586 Tomato leaf curl Togo virus-[Togo

VIRL_917 Tomato leaf curl Vietnam virus DNA A

VIRL_3143 Tomato leaf curl virus

VIRL_3346 Tomato leaf curl virus satellite DNA

VIRL_3028 Tomato leaf curl virus-associated DNA beta

VIRL_1897 Tomato leaf curl virus-Pune-associated DNA beta DNA-A

VIRL_27 Tomato leaf deformation virus DNA-A

VIRL_1612 Tomato marchitez virus RNA 1

VIRL_1611 Tomato marchitez virus RNA 2

VIRL_852 Tomato mild mosaic virus DNA-A

VIRL_851 Tomato mild mosaic virus DNA-B

VIRL_1715 Tomato mild yellow leaf curl Aragua virus DNA A

VIRL_1714 Tomato mild yellow leaf curl Aragua virus DNA B

VIRL_3149 Tomato mosaic Havana virus DNA A

VIRL_2195 Tomato mosaic Havana virus DNA B

VIRL_1427 Tomato mosaic leaf curl virus DNA A

VIRL_1426 Tomato mosaic leaf curl virus DNA B

VIRL_413 Tomato mosaic virus

VIRL_2369 Tomato mottle Taino virus DNA A

VIRL_2364 Tomato mottle Taino virus DNA B

VIRL_3394 Tomato mottle virus DNA A

VIRL_3393 Tomato mottle virus DNA B

VIRL_2201 Tomato pseudo-curly top virus

VIRL_2199 Tomato ringspot virus RNA 1

VIRL_2200 Tomato ringspot virus RNA 2

VIRL_2341 Tomato rugose mosaic virus DNA A

VIRL_2340 Tomato rugose mosaic virus DNA B

VIRL_3038 Tomato severe leaf curl virus

VIRL_871 Tomato severe rugose virus DNA A

VIRL_1691 Tomato severe rugose virus DNA B

VIRL_3380 Tomato spotted wilt virus genomic RNA

VIRL_3379 Tomato spotted wilt virus RNA L

VIRL_2464 Tomato spotted wilt virus RNA M

VIRL_1728 Tomato torrado virus RNA1

VIRL_1724 Tomato torrado virus RNA2

VIRL_1674 Tomato yellow dwarf disease associated satellite DNA beta-[Kochi] virus

VIRL_3131 Tomato yellow leaf curl China virus

VIRL_3007 Tomato yellow leaf curl China virus associated DNA 1

VIRL_3059 Tomato yellow leaf curl China virus satellite DNA beta

VIRL_1907 Tomato yellow leaf curl Guangdong virus DNA-A

VIRL_1916 Tomato yellow leaf curl Indonesia virus-[Lembang]

VIRL_6 Tomato yellow leaf curl Kanchanaburi virus DNA A

VIRL_7 Tomato yellow leaf curl Kanchanaburi virus DNA B

VIRL_913 Tomato yellow leaf curl Malaga virus

VIRL_2002 Tomato yellow leaf curl Mali virus-associated DNA beta

VIRL_3167 Tomato yellow leaf curl Sardinia virus

VIRL_996 Tomato yellow leaf curl Thailand betasatellite - [India

VIRL_3006 Tomato yellow leaf curl Thailand virus associated DNA 1

VIRL_3377 Tomato yellow leaf curl Thailand virus DNA A

VIRL_3376 Tomato yellow leaf curl Thailand virus DNA B

VIRL_3019 Tomato yellow leaf curl Thailand virus satellite DNA beta

VIRL_876 Tomato yellow leaf curl Vietnam virus DNA-A

VIRL_2814 Tomato yellow leaf curl Vietnam virus satellite DNA beta

VIRL_1389 Tomato yellow leaf curl virus

VIRL_2792 Tomato yellow leaf curl virus-associated DNA beta

VIRL_1425 Tomato yellow margin leaf curl virus DNA A

VIRL_1424 Tomato yellow margin leaf curl virus DNA B

VIRL_896 Tomato yellow spot virus DNA-A

VIRL_895 Tomato yellow spot virus DNA-B

VIRL_844 Tomato yellow vein streak virus DNA-A

VIRL_843 Tomato yellow vein streak virus DNA-B

VIRL_1542 Tomato zonate spot virus segment L

VIRL_1647 Tomato zonate spot virus segment M

VIRL_1543 Tomato zonate spot virus segment S

VIRL_307 Torque teno canis virus

VIRL_291 Torque teno douroucouli virus

VIRL_306 Torque teno felis virus

VIRL_317 Torque teno midi virus 1

VIRL_285 Torque teno midi virus 2

VIRL_281 Torque teno mini virus 1

VIRL_292 Torque teno mini virus 2

VIRL_290 Torque teno mini virus 3

VIRL_288 Torque teno mini virus 4

VIRL_289 Torque teno mini virus 5

VIRL_283 Torque teno mini virus 6

VIRL_296 Torque teno mini virus 7

VIRL_310 Torque teno mini virus 8

VIRL_330 Torque teno mini virus 9

VIRL_308 Torque teno sus virus 1

VIRL_286 Torque teno sus virus 2

VIRL_293 Torque teno tamarin virus

VIRL_331 Torque teno virus 1

VIRL_302 Torque teno virus 10

VIRL_303 Torque teno virus 12

VIRL_301 Torque teno virus 14

VIRL_282 Torque teno virus 15

VIRL_287 Torque teno virus 16

VIRL_300 Torque teno virus 19

VIRL_46 Torque teno virus 2

VIRL_295 Torque teno virus 25

VIRL_299 Torque teno virus 26

VIRL_304 Torque teno virus 27

VIRL_305 Torque teno virus 28

VIRL_297 Torque teno virus 3

VIRL_309 Torque teno virus 4

VIRL_284 Torque teno virus 6

VIRL_298 Torque teno virus 7

VIRL_294 Torque teno virus 8

VIRL_2922 Toscana virus segment L

VIRL_2408 Toscana virus segment M

VIRL_2923 Toscana virus segment S

VIRL_1324 Transmissible gastroenteritis virus

VIRL_2143 Tree shrew adenovirus

VIRL_367 Triatoma virus

VIRL_224 Trichechus manatus latirostris papillomavirus type 1

VIRL_167 Trichodysplasia spinulosa-associated polyomavirus

VIRL_3169 Trichomonas vaginalis virus

VIRL_976 Trichomonas vaginalis virus 3

VIRL_2194 Trichomonas vaginalis virus II

VIRL_3671 Trichoplusia ni ascovirus 2c

VIRL_2339 Trichoplusia ni cytoplasmic polyhedrosis virus 15 segment 1

VIRL_3352 Trichoplusia ni cytoplasmic polyhedrosis virus 15 segment 10

VIRL_2331 Trichoplusia ni cytoplasmic polyhedrosis virus 15 segment 11

VIRL_2338 Trichoplusia ni cytoplasmic polyhedrosis virus 15 segment 2

VIRL_2337 Trichoplusia ni cytoplasmic polyhedrosis virus 15 segment 3

VIRL_2330 Trichoplusia ni cytoplasmic polyhedrosis virus 15 segment 4

VIRL_2336 Trichoplusia ni cytoplasmic polyhedrosis virus 15 segment 5

VIRL_2335 Trichoplusia ni cytoplasmic polyhedrosis virus 15 segment 6

VIRL_2334 Trichoplusia ni cytoplasmic polyhedrosis virus 15 segment 7

VIRL_2333 Trichoplusia ni cytoplasmic polyhedrosis virus 15 segment 8

VIRL_2332 Trichoplusia ni cytoplasmic polyhedrosis virus 15 segment 9

VIRL_1445 Trichoplusia ni SNPV

VIRL_129 Triticum mosaic virus

VIRL_2092 Tula virus segment L

VIRL_2411 Tula virus segment M

VIRL_2988 Tula virus segment S

VIRL_3635 Tulare apple mosaic virus RNA1

VIRL_3634 Tulare apple mosaic virus RNA2

VIRL_3633 Tulare apple mosaic virus RNA3

VIRL_2150 Tulip virus X

VIRL_254 Tupaia paramyxovirus

VIRL_1055 Tupaia virus

VIRL_60 Tupaiid herpesvirus 1

VIRL_31 Turdivirus 1

VIRL_30 Turdivirus 2

VIRL_29 Turdivirus 3

VIRL_3389 Turkey adenovirus A

VIRL_3305 Turkey adenovirus A

VIRL_2448 Turkey astrovirus

VIRL_1174 Turkey astrovirus 2

VIRL_2762 Turkey coronavirus

VIRL_1064 Turnip crinkle virus

VIRL_2177 Turnip crinkle virus satellite RNA

VIRL_3548 Turnip crinkle virus virulent satellite RNA C

VIRL_49 Turnip curly top virus

VIRL_156 Turnip mosaic virus

VIRL_107 Turnip ringspot virus RNA 1

VIRL_106 Turnip ringspot virus RNA 2

VIRL_2140 Turnip rosette virus

VIRL_1263 Turnip vein-clearing virus

VIRL_3128 Turnip yellow mosaic virus

VIRL_2230 Turnip yellows virus

VIRL_533 Tursiops truncatus papillomavirus type 1

VIRL_590 Tursiops truncatus papillomavirus type 2

VIRL_532 Tursiops truncatus papillomavirus type 3

VIRL_842 TYLCAxV-Sic1-[IT

VIRL_1956 TYLCCNV-[Y322] satellite DNA beta virus

VIRL_2480 UR2 sarcoma virus

VIRL_856 Urochloa streak virus

VIRL_539 Ursus maritimus papillomavirus type 1

VIRL_212 Ustilago maydis virus H1

VIRL_1098 Usutu virus

VIRL_208 Uukuniemi virus

VIRL_1176 Uukuniemi virus chromosome segment M

VIRL_2093 Uukuniemi virus segment L

VIRL_618 Vaccinia virus

VIRL_711 Variola virus

VIRL_1100 Varroa destructor virus 1

VIRL_1336 Velvet bean severe mosaic virus DNA A

VIRL_1335 Velvet bean severe mosaic virus DNA B

VIRL_28 Velvet tobacco mottle virus

VIRL_2190 Velvet tobacco mottle virus Satellite RNA

VIRL_3472 Venezuelan equine encephalitis virus

VIRL_1631 Verbena virus Y

VIRL_200 Vernonia yellow vein betasatellite

VIRL_203 Vernonia yellow vein virus DNA-A

VIRL_3353 Vesicular exanthema of swine virus

VIRL_314 Vesicular stomatitis Indiana virus

VIRL_2602 Vibrio phage fs1

VIRL_696 Vibrio phage fs2

VIRL_2615 Vibrio phage K139

VIRL_2525 Vibrio phage kappa

VIRL_2571 Vibrio phage KSF-1phi

VIRL_650 Vibrio phage KVP40

VIRL_998 Vibrio phage N4

VIRL_479 Vibrio phage VEJphi

VIRL_2572 Vibrio phage Vf12

VIRL_2573 Vibrio phage Vf33

VIRL_2627 Vibrio phage VfO3K6

VIRL_2626 Vibrio phage VfO4K68

VIRL_1470 Vibrio phage VGJphi

VIRL_467 Vibrio phage VHML

VIRL_2579 Vibrio phage VP2

VIRL_2575 Vibrio phage VP5

VIRL_567 Vibrio phage VP882

VIRL_333 Vibrio phage VP93

VIRL_1212 Vibrio phage VpV262

VIRL_2614 Vibrio phage VSK

VIRL_2564 Vibriophage VP4

VIRL_2892 Vicia cryptic virus RNA1

VIRL_1049 Vicia cryptic virus RNA2

VIRL_1974 Vicia faba endornavirus

VIRL_3378 Viral hemorrhagic septicemia virus

VIRL_3471 Visna/Maedi virus

VIRL_3410 Walleye dermal sarcoma virus

VIRL_3618 Walrus calicivirus

VIRL_3219 Watermelon chlorotic stunt virus DNA A

VIRL_3218 Watermelon chlorotic stunt virus DNA B

VIRL_1110 Watermelon mosaic virus

VIRL_79 Watermelon silver mottle virus segment L

VIRL_78 Watermelon silver mottle virus segment M

VIRL_77 Watermelon silver mottle virus segment S

VIRL_964 Wesselsbron virus

VIRL_2799 West Nile virus (lineage I strain NY99)

VIRL_3447 West Nile virus (lineage II strain 956)

VIRL_2189 Western equine encephalomyelitis virus

VIRL_535 Western roedeer papillomavirus 1

VIRL_3313 Wheat dwarf virus

VIRL_3589 Wheat eqlid mosaic virus

VIRL_3407 Wheat streak mosaic virus

VIRL_126 Wheat yellow dwarf virus-RPV

VIRL_3360 Wheat yellow mosaic virus RNA 1

VIRL_3361 Wheat yellow mosaic virus RNA 2

VIRL_3527 White ash mosaic virus

VIRL_1902 White bream virus

VIRL_204 White clover cryptic virus 1 RNA1

VIRL_1107 White clover cryptic virus 1 RNA2

VIRL_3170 White clover mosaic virus

VIRL_2771 Whitewater Arroyo virus segment L

VIRL_2773 Whitewater Arroyo virus segment S

VIRL_2145 Wild potato mosaic virus

VIRL_430 Wild tomato mosaic virus

VIRL_1617 Wissadula golden mosaic St Thomas Virus DNA A

VIRL_1616 Wissadula golden mosaic St Thomas Virus DNA B

VIRL_2020 Wisteria vein mosaic virus

VIRL_2742 Wongabel virus

VIRL_1201 Woodchuck hepatitis virus

VIRL_161 Woolly monkey sarcoma virus

VIRL_561 WU Polyomavirus

VIRL_2498 Xanthomonas phage Cf1c

VIRL_2561 Xanthomonas phage OP1

VIRL_1962 Xanthomonas phage OP2

VIRL_487 Xanthomonas phage phiL7

VIRL_2539 Xanthomonas phage Xop411

VIRL_2591 Xanthomonas phage Xp10

VIRL_2035 Xanthomonas phage Xp15

VIRL_1613 Xenopus laevis endogenous retrovirus Xen1

VIRL_2868 Xenotropic MuLV-related virus VP62

VIRL_762 Xestia c-nigrum granulovirus

VIRL_940 Xylella phage Xfas53

VIRL_2855 Y73 sarcoma virus

VIRL_647 Yaba monkey tumor virus

VIRL_685 Yaba-like disease virus

VIRL_2414 Yam mosaic virus

VIRL_3446 Yellow fever virus

VIRL_3112 Yellowtail ascites virus segment A

VIRL_3111 Yellowtail ascites virus segment B

VIRL_654 Yersinia pestis phage phiA1122

VIRL_2544 Yersinia phage Berlin

VIRL_657 Yersinia phage L-413C

VIRL_689 Yersinia phage phiYeO3-12

VIRL_2412 Yersinia phage PY54

VIRL_2516 Yersinia phage Yepe2

VIRL_2103 Yokose virus

VIRL_3064 Youcai mosaic virus

VIRL_1973 Yunnan orbivirus segment 1

VIRL_1964 Yunnan orbivirus segment 10

VIRL_1972 Yunnan orbivirus segment 2

VIRL_1971 Yunnan orbivirus segment 3

VIRL_1970 Yunnan orbivirus segment 4

VIRL_1969 Yunnan orbivirus segment 5

VIRL_1968 Yunnan orbivirus segment 6

VIRL_1967 Yunnan orbivirus segment 7

VIRL_1966 Yunnan orbivirus segment 8

VIRL_1965 Yunnan orbivirus segment 9

VIRL_380 Zaire ebolavirus

VIRL_3511 Zantedeschia mild mosaic virus

VIRL_2668 Zika virus

VIRL_3013 Zinnia leaf curl disease associated sequence virion

VIRL_1457 Zinnia leaf curl virus-associated DNA beta

VIRL_3147 Zucchini green mottle mosaic virus

VIRL_3314 Zucchini yellow mosaic virus

VIRL_1115 Zygocactus virus X

VIRL_1213 Zygosaccharomyces bailii virus Z

BACT_6001 Enterococcus faecalis OG1RF

BACT_6002 Helicobacter pylori 26695

BACT_6003 Lactobacillus gasseri ATCC 33323

BACT_6004 Listeria monocytogenes EGD-e

BACT_6005 Propionibacterium acnes KPA171202

BACT_6006 Pseudomonas aeruginosa PAO1

BACT_6007 Streptococcus agalactiae 2603V/R

BACT_6008 Streptococcus pneumoniae TIGR4

BACT_6009 Staphylococcus epidermidis ATCC 12228
