# Supplementary figures and images for: Optimizing Read Mapping to Reference Genomes to Determine Composition and Species Prevalence in Microbial Communities
Source: PLoS One. 2012 Jun 13;7(6):e36427. doi: 10.1371/journal.pone.0036427 (PMC3374613; doi:10.1371/journal.pone.0036427)

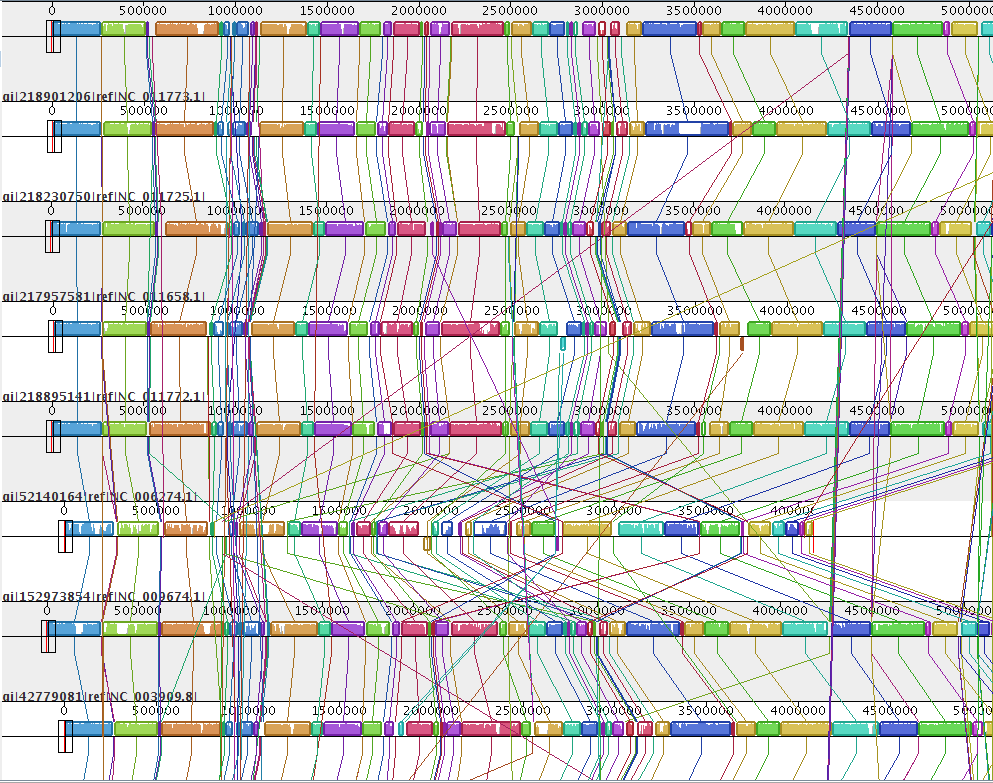

Supplement: Figure S1 — Example Mauve alignment. This picture shows a screenshot of an example Mauve alignment of 8 similar strains of B. cereus. Colored blocks show regions of homology between organisms with the amplitude shown within each box showing the strength of the similarity. Lines between strains show smaller regions of homology between sequences. (TIF) [file pone.0036427.s005.tif]

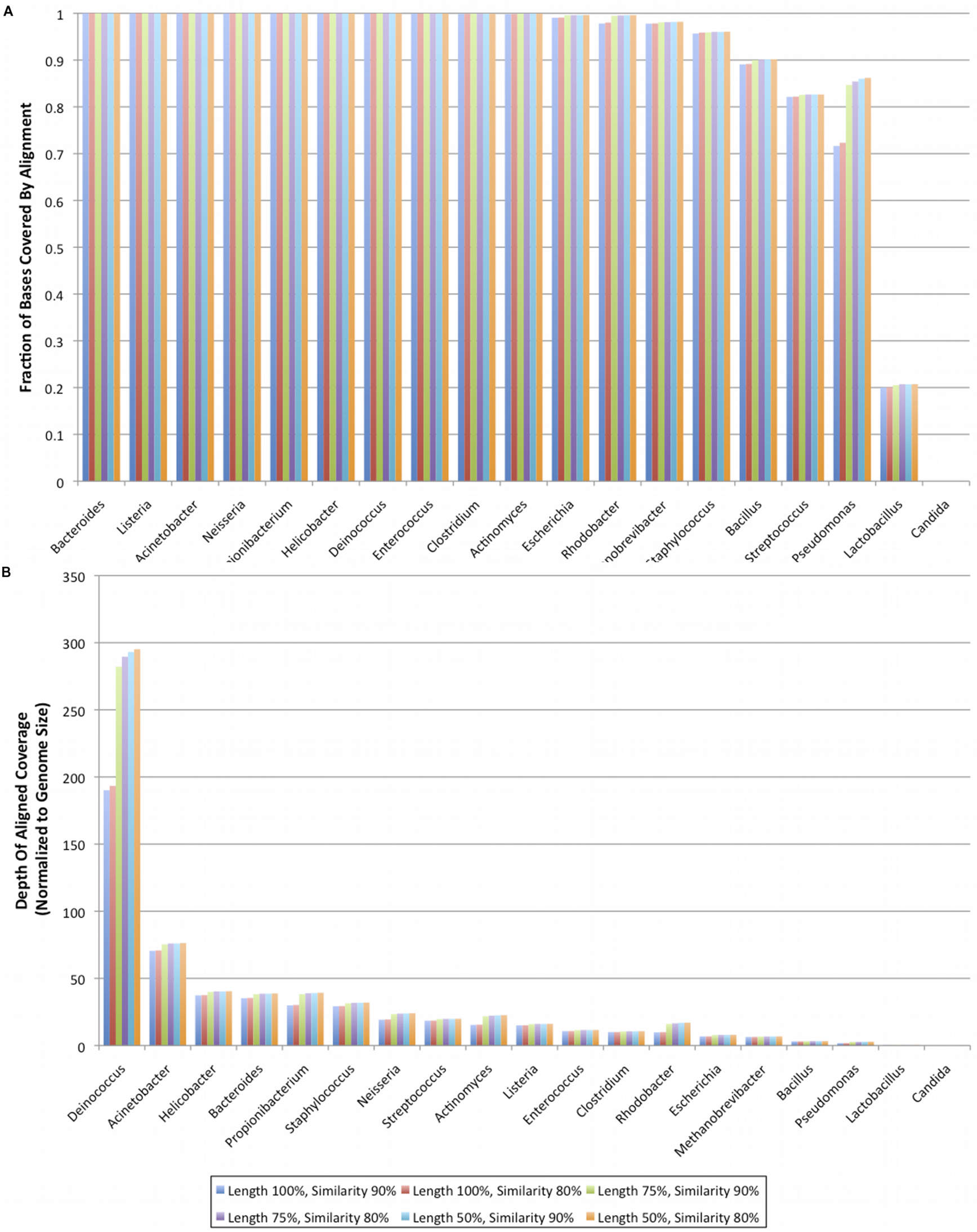

Supplement: Figure S2 — Alignment parameter effects on breadth and depth of coverage of original MMD strains. (A). Parameter effects on genome coverage of the MMD at the genus level. This chart shows the effect of varying the parameters on the coverage of the mock genomes on the genus level. For the genera Streptococcus and Staphylococcus, which are represented by more than a single strain in the mock community pool, the values are averaged across each member strain. The genus Pseudomonas, represented in the mock community by the strain P. aeruginosa PAO1, displays a marked decrease in coverage when using the stringent 100% length cutoff. (B). Parameter effects on genome depth of coverage of the MMD at the genus level. This chart shows the effect of varying the parameters on the depth of coverage found for the mock genomes on the genus level. For the genera Streptococcus and Staphylococcus, which are represented by more than a single strain in our mock community pool, the values are averaged across each member strain. The genus Deinococcus, which in the mock community is represented by the strain D. radiodurans R1, shows a marked decrease in estimated depth of coverage for parameter combinations that require 100% length to align. (TIF) [file pone.0036427.s006.tif]

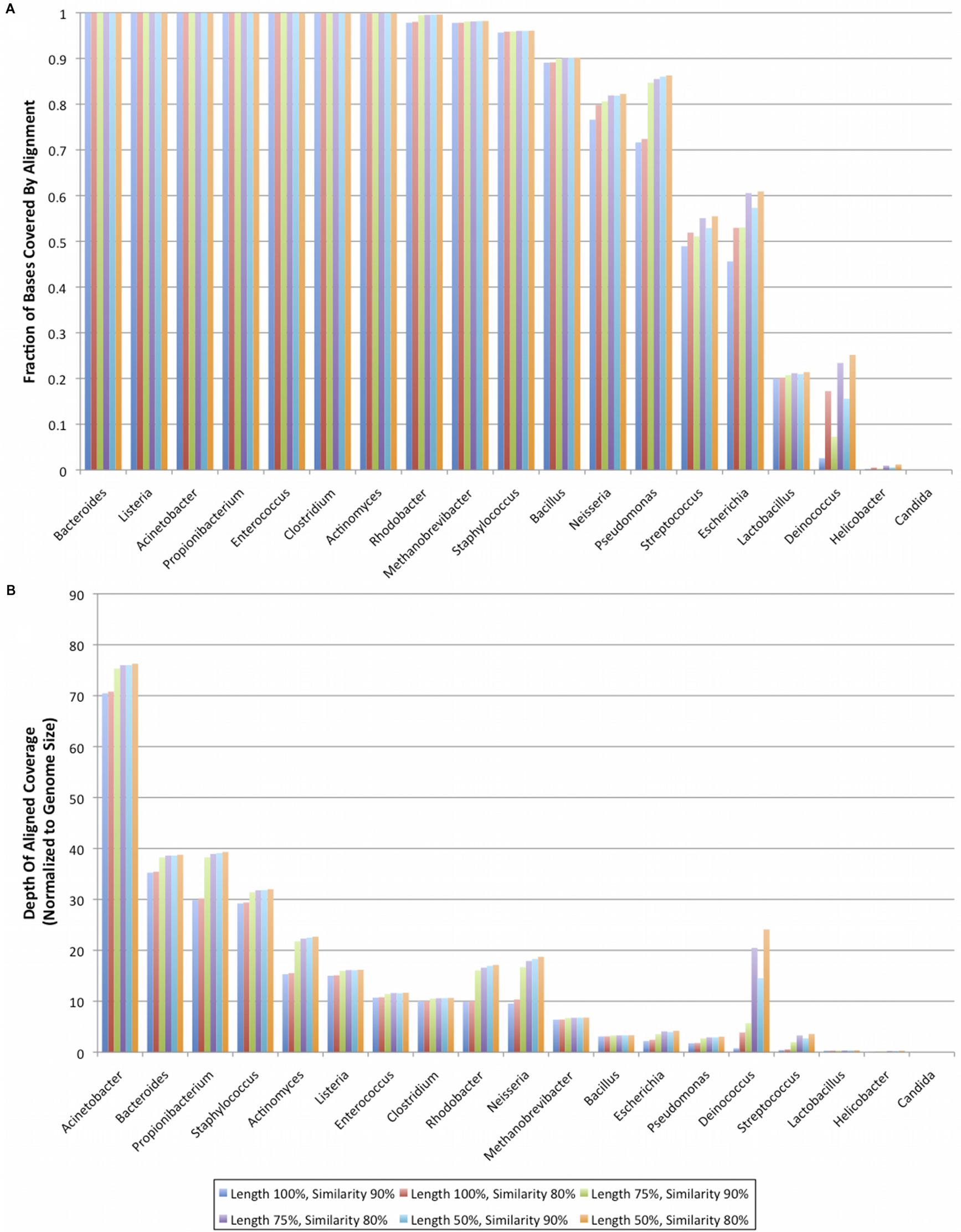

Supplement: Figure S3 — Alignment parameter effects on breadth and depth of coverage of amended MMD strains. (A). Parameter effects on genome coverage of the amended MMD at the genus level. This chart shows the effect of varying the parameters on the breadth of coverage of the mock genomes on the genus level. The most affected genera are the ones where the strain membership was modified before running the alignments. For Deinococcus, Escherichia, Helicobacter and Neisseria, the member strain was removed, and a non-mock strain from the same genus was put in its place, and for Streptococcus, two of the three strains were removed, leaving the original S.mutans UA159 strain intact. This figure illustrates that for those genera not having strains present in the mock community, the similarity value begins to have more of an effect on the numbers able to align. (B). Parameter effects on genome depth of coverage of the amended MMD at the genus level. This chart shows the effect of varying the parameters on the depth of coverage of the mock genomes on the genus level. The most affected genera are the ones where the strain membership was modified before running the alignments. For Deinococcus, Escherichia, Helicobacter and Neisseria, the member strain was removed, and a non-mock strain from the same genus was put in its place, and for Streptococcus, two of the three strains were removed, leaving the original S.mutans UA159 strain intact. This figure illustrates that for those genera not having strains present in the mock community, the similarity value begins to have more of an effect on the numbers able to align. (TIF) [file pone.0036427.s007.tif]
